# Telomere length promotes colorectal cancer through dual parallel pathways involving growth signaling and protein metabolism

**DOI:** 10.1186/s41182-025-00854-x

**Published:** 2025-12-24

**Authors:** Chuang Liu, Fang Wang, Zhen Zhang, Qiang Su, Yifeng Li

**Affiliations:** 1 Department of Gastroenterology, First Affiliated Hospital of Guizhou University of Traditional Chinese Medicine, Guiyang, Guizhou China; 2 First Clinical Medical College, Guizhou University of Traditional Chinese Medicine, Guiyang, Guizhou China

**Keywords:** Telomere length, Colorectal cancer, Mendelian randomization, Mediation analysis, IGF-1, Protein metabolism

## Abstract

**Background:**

While telomeres traditionally protect against cancer through genomic stability, recent evidence suggests a paradoxical association with increased malignancy risk. This study employed comprehensive Mendelian randomization (MR) to investigate the causal relationship between telomere length (TL) and colorectal cancer (CRC) risk and elucidate the underlying biological mechanisms through systematic mediation analysis.

**Methods:**

We performed two-sample MR using genetic instruments from large-scale genome-wide association studies (GWASs). CRC data were obtained from FinnGen R12 (discovery cohort: 11,790 cases and 378,749 controls) and the GWAS Catalog (replication cohort: 19,948 cases and 12,124 controls). The inverse-variance weighted method served as the primary analysis, complemented by MR‒Egger, weighted median, and MR-PRESSO sensitivity analyses. The multivariable MR was adjusted for body mass index (BMI), processed meat intake, inflammatory bowel disease (IBD), and colorectal polyps. Two-step mediation analysis investigated 35 blood and urine biomarkers as potential mediators, with colocalization analysis performed to distinguish linkage from pleiotropy.

**Results:**

Genetically predicted longer telomeres were consistently positively associated with increased CRC risk across both cohorts (discovery: odds ratio [OR] = 1.282, 95% confidence interval [CI] 1.126–1.459, *P* < 0.001; replication: OR = 1.253, 95% CI 1.067–1.472, *P* = 0.006). This association remained robust across multiple analytical methods and was independent of BMI, processed meat intake, IBD, and colorectal polyps. Mediation analysis revealed three significant mediators representing dual parallel mechanisms: insulin-like growth factor-1 (IGF-1) mediated 4.2% of the total effect through enhanced growth signaling (*P* = 0.0265), whereas total protein (TP) and nonalbumin protein (NAP) collectively mediated 19.67% through compromised protein homeostasis (10.33% and 9.34%, respectively; both *P* < 0.005). Colocalization analysis revealed the shared genetic architecture underlying these associations.

**Conclusions:**

Longer telomeres causally increase CRC risk through dual parallel pathways: enhanced cellular proliferation via IGF-1 signaling and compromised immune surveillance through protein metabolic dysfunction. These findings challenge conventional protective roles attributed to telomeres and suggest that individuals with genetically longer telomeres may benefit from enhanced screening protocols and targeted interventions addressing both growth factor signaling and protein metabolic homeostasis.

**Supplementary Information:**

The online version contains supplementary material available at 10.1186/s41182-025-00854-x.

## Introduction

Telomeres, the protective DNA‒protein complexes that cap chromosomal ends, have long been hailed as cellular guardians against malignancy. The prevailing paradigm, established over decades of research, posits that telomere shortening drives genomic instability and cancer initiation, whereas longer telomeres preserve chromosomal integrity and confer protection against tumorigenesis [1, 2]. This fundamental principle has shaped our understanding of cellular aging and cancer biology, positioning telomere maintenance as a critical defense mechanism against malignant transformation.

However, emerging epidemiological evidence presents a striking paradox that challenges this established doctrine. Large-scale prospective studies have consistently documented positive associations between longer telomeres and increased risk of several cancer types, including lung, breast, and, notably, colorectal cancer (CRC) [3–5]. This counterintuitive finding suggests that the relationship between telomere biology and cancer development may be far more complex than previously appreciated and potentially involves mechanisms that extend beyond the maintenance of genomic stability.

The mechanistic basis for this apparent contradiction remains poorly understood, largely owing to the inherent limitations of observational studies in establishing causal relationships and dissecting biological pathways. Traditional epidemiological approaches are confounded by numerous lifestyle and environmental factors that simultaneously influence both telomere length (TL) and cancer risk, making it challenging to determine whether longer telomeres are truly causative or merely correlative with increased malignancy [6]. Furthermore, the potential for reverse causation—where subclinical cancer affects telomere dynamics—has remained an unresolved confounder in interpreting these associations.

The advent of Mendelian randomization (MR) offers a powerful solution to these methodological challenges by leveraging genetic variants as natural experiments to infer causal relationships [7, 8]. Using genetic instruments that influence TL from birth, MR circumvents the confounding and reverse causation issues that plague observational studies. More importantly, the recent development of sophisticated mediation analysis techniques within the MR framework enables systematic dissection of the biological pathways through which exposures influence disease outcomes [9, 10].

CRC represents an ideal model for investigating telomere‒cancer relationships, given its well-characterized genetic architecture, substantial global burden, and strong associations with metabolic and inflammatory processes [11, 12]. The disease’s lengthy preclinical phase and established links to growth factor signaling pathways, particularly the insulin-like growth factor (IGF) axis, provide a biological framework for understanding how TLs might influence carcinogenesis through mechanisms beyond genomic instability [13, 14].

Here, we employed a comprehensive MR approach, combining discovery and replication cohorts with systematic mediation analysis, to establish the causal relationship between TL and CRC definitively. Using genetic instruments derived from the largest available genome-wide association studies (GWASs), we investigated 35 circulating biomarkers as potential mediators, spanning metabolic, inflammatory, and protein homeostasis pathways [15]. Our analysis revealed that longer telomeres promote CRC through dual opposing mechanisms: enhanced cellular proliferation via growth factor signaling and compromised immune surveillance through disrupted protein metabolism.

These findings not only resolve the apparent telomere–cancer paradox but also illuminate previously unrecognized biological pathways linking cellular aging mechanisms to malignant transformation. The discovery of these dual opposing effects provides a new conceptual framework for understanding how the same biological factor can simultaneously promote and protect against cancer, depending on the specific pathway activated. This work has immediate implications for cancer risk stratification and suggests novel therapeutic targets for precision prevention strategies.

## Materials and methods

### Study design

**Fig. 1:**
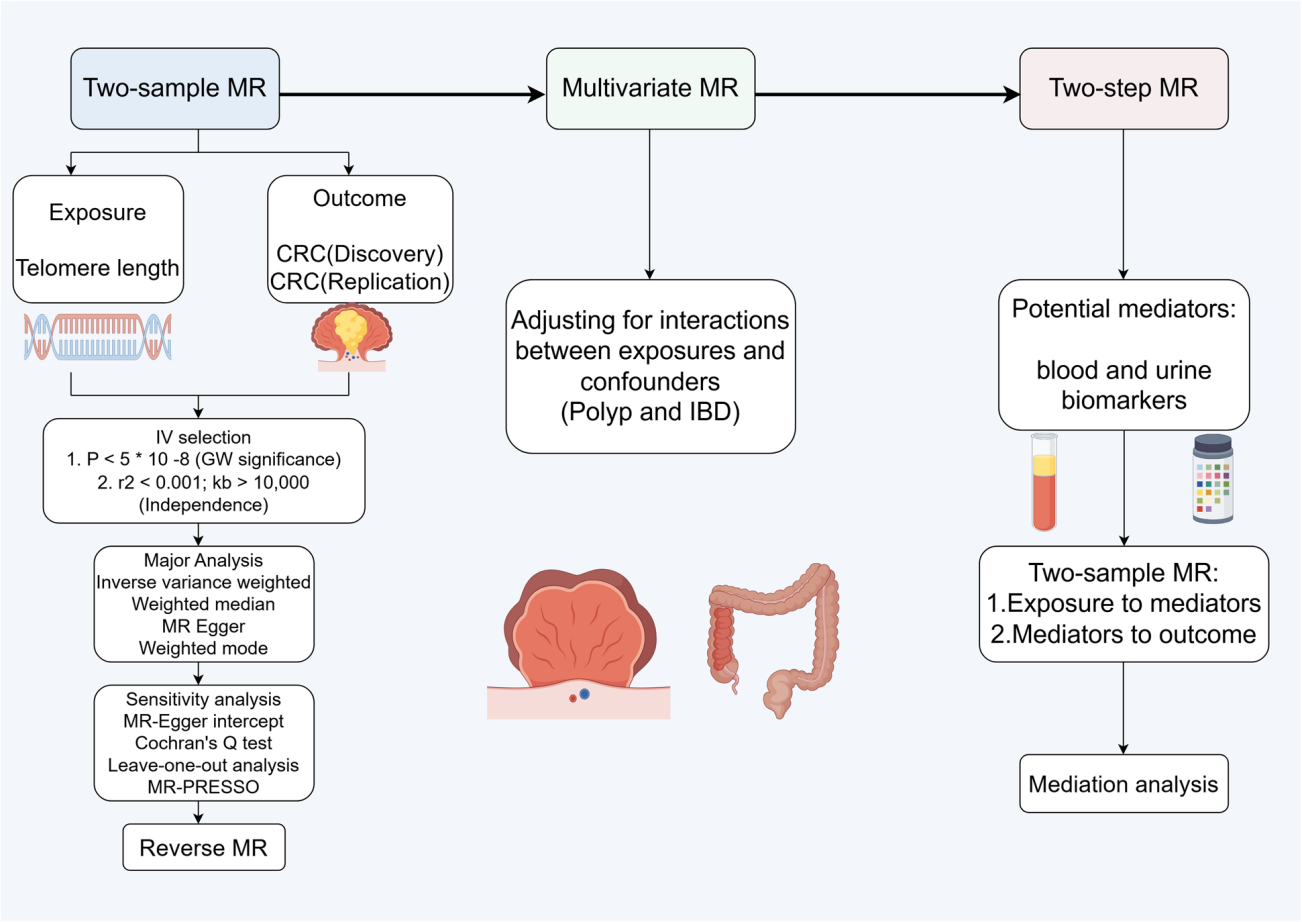
Study design and analytical framework for MR analysis of TL and CRC risk. This flowchart illustrates the comprehensive analytical strategy employed to investigate the causal relationship between telomere length and colorectal cancer risk via multiple Mendelian randomization approaches. The study design follows a systematic three-stage framework: (1) two-sample MR analysis to establish the primary causal relationship between genetically predicted telomere length and CRC risk; (2) multivariable MR analysis to account for potential confounding by major CRC risk factors (BMI, processed meat, Polyp and IBD); and (3) two-step MR analysis to identify mediating pathways through blood and urine biomarkers. The analytical pipeline incorporates rigorous instrument selection criteria, multiple statistical methods, comprehensive sensitivity analyses, and mediation analysis to ensure robust causal inference while providing mechanistic insights. MR, Mendelian randomization; CRC, colorectal cancer; IV, instrumental variable; GW, genome wide; IBD, inflammatory bowel disease; BMI, body mass index; kb, kilobase; *r*^2^, linkage disequilibrium correlation coefficient; *P*, *P* value; MR-PRESSO, MR pleiotropy residual sum and outlier

We conducted a comprehensive two-sample MR analysis following the STROBE-MR guidelines to investigate the causal relationship between TL and CRC risk. Genetic instruments for TL were selected from the largest available GWAS meta-analysis using genome-wide significant variants (*P* < 5 × 10^−8^) with stringent linkage disequilibrium pruning (*r*^2^ < 0.001, distance > 10,000 kb) to satisfy the three core MR assumptions: relevance, independence, and exclusion restriction. CRC outcome data were obtained from FinnGen R12 as the discovery cohort and from the GWAS Catalog (GCST012879) for replication. Our analytical framework employed the inverse-variance weighted (IVW) method for primary analysis, complemented by extensive sensitivity analyses (MR‒Egger, weighted median, weighted mode, MR-PRESSO), bidirectional MR to assess reverse causation, and multivariable MR adjusted for body mass index (BMI), processed meat intake, inflammatory bowel disease (IBD), and colorectal polyps. To elucidate the underlying mechanisms, we performed systematic two-step mediation analysis investigating 35 blood and urine biomarkers, including IGF-1 and protein metabolism markers, with colocalization analysis to distinguish linkage from pleiotropy. All analyses were conducted via R version 4.2.1 with the TwoSampleMR and MR-PRESSO packages, with the study design illustrated in Fig. 1.

### Data sources

GWAS summary statistics for TL were obtained from the UK Biobank (UKB) cohort, which encompasses genome-wide genotyping data from 472,174 well-characterized UKB participants of European ancestry [16]. To ensure robust causal inference for CRC, we utilized two independent European ancestry data sets in our genetic association analyses. The discovery data set was derived from FinnGen Release 12, comprising 11,790 CRC cases and 378,749 European ancestry controls [17], whereas for replication purposes, we incorporated an additional data set from the GWAS Catalog (ID: GCST012879) containing 19,948 CRC cases and 12,124 European ancestry controls, providing comprehensive genetic information on CRC susceptibility [18]. Summary statistics for 35 blood and urine biomarkers, encompassing common phenotypes, including growth factors, proteins, and metabolites, were obtained from 363,228 participants, with all data accessible through the data services provided in the original publication [15]. To adjust for established CRC risk factors, genetic instruments for potential confounding factors were obtained from multiple sources: colorectal polyps, body mass index, and processed meat intake from UK Biobank GWAS summary statistics, and IBD conditions (Crohn’s disease and ulcerative colitis) from the International IBD Genetics Consortium (IIBDGC). To minimize bias from population stratification and maintain methodological consistency, we strictly restricted our analyses to individuals of European ancestry in both the exposure and outcome data sets, thereby enhancing the reliability of our causal inference analyses. Additional details regarding these data sources are provided in Supplementary Table 1.

### Instrumental variable selection

Our instrumental variable selection process followed a systematic multistep approach to ensure robust causal inference. First, we extracted single nucleotide polymorphisms (SNPs) demonstrating genome-wide significance (*P* < 5 × 10^−8^) in the exposure GWAS, a criterion consistently applied across our two-sample MR analyses and two-step MR investigations. We subsequently performed linkage disequilibrium (LD) pruning (*r*^2^ < 0.001, clumping distance = 10,000 kb) to eliminate highly correlated variants that could introduce bias into our analyses. Third, we harmonized effect sizes and alleles between the exposure and outcome data sets to maintain analytical consistency and ensure proper alignment of genetic effects. To mitigate weak instrument bias, we calculated *F* statistics for each SNP via the formula *F* = *R*^2^(*n* − *k* − 1)/*k*(1 − *R*^2^), where *R*^2^ represents the proportion of variance explained by the instrument, *n* represents the sample size, and k represents the number of instruments, subsequently excluding instruments with *F* values less than 10 to ensure sufficient statistical power [19]. We conducted comprehensive screening through the GWAS Catalog (https://www.ebi.ac.uk/gwas/) to identify and exclude SNPs significantly associated with known confounding factors (*P* < 1 × 10^−5^), including colorectal polyps, family history of CRC, ulcerative colitis, Crohn’s disease, obesity, hypertension, diabetes, nonalcoholic fatty liver disease, hyperlipidemia, smoking, alcohol consumption, sedentary lifestyle, gut microbiome composition, occupational exposures, and air pollution [20–22], thereby further reducing confounding bias and excluding instrumental variables directly associated with the outcome. Through our validation process, we confirmed that a subset of instrumental variables showed significant associations with these potential confounders or outcomes, and we performed additional analyses after removing outliers identified through the MR-PRESSO method (detailed in Supplementary Table 3). The final set of SNPs meeting all predefined criteria served as instrumental variables in our MR analyses, with detailed characteristics of these genetic instruments provided in Supplementary Table 2. We employed a hypothesis-driven approach for selecting biomarkers on the basis of three established biological pathways: (1) IGF-1 and growth signaling pathways previously linked to both telomere biology and CRC development; (2) protein homeostasis markers with documented associations with telomere function and cancer progression; and (3) metabolic regulation markers implicated in telomere–cancer relationships. This resulted in a comprehensive analysis of all 35 available protein and metabolic biomarkers from the UK Biobank high-quality data set.

### Statistical analysis

#### Primary causal inference approach

Our principal analytical strategy employed the IVW methodology for MR analysis. This approach operates under the premise that all genetic instruments maintain validity and deliver a weighted average of SNP-specific causal estimates, offering optimal statistical power when horizontal pleiotropy is absent [23]. We systematically implemented Cochran’s *Q* test to examine heterogeneity across exposure‒SNP associations. Fixed-effects models were utilized when heterogeneity was nonsignificant, whereas random-effects models were applied when heterogeneity was detected [24]. To strengthen the robustness and credibility of our findings, we incorporated several complementary analytical approaches. The MR‒Egger methodology evaluates potential pleiotropic effects through regression intercepts, with *P* values below 0.05 indicating directional pleiotropy while maintaining valid causal estimation under instrument invalidity [25]. The weighted median approach preserves consistency when up to 50% of the statistical weight is derived from valid instruments [26]. In addition, the weighted mode methodology estimates causal effects by identifying patterns in weighted SNP-specific causal estimates, which is particularly valuable under horizontal pleiotropy conditions [27]. This multimethodological framework enables comprehensive consistency evaluation across different analytical paradigms, each grounded in distinct instrument validity assumptions. To ensure statistical rigor and address multiple testing concerns, the results from batch analyses (such as the initial phase of two-step methodology involving 35 biomarkers) underwent false discovery rate (FDR) correction, maintaining strict Type I error control while preserving statistical power for discovery [28].

#### Secondary analytical assessments

We conducted comprehensive secondary evaluations to assess heterogeneity, pleiotropy, and sensitivity. Horizontal pleiotropy assessment combined MR‒Egger intercept analysis (intercepts approaching zero suggesting lower pleiotropy probability) with MR-PRESSO global testing for outlier variant management. Following outlier detection through MR-PRESSO, we executed an iterative optimization procedure, repeating MR analysis after outlier removal to ensure causal estimate robustness [29]. Extensive sensitivity analyses validated our findings, including leave-one-out (LOO) assessments that systematically excluded individual SNPs to evaluate their impact on overall effect estimates [30]. Sensitivity evaluations further examined consistency across different MR methodologies (IVW, MR‒Egger, weighted median, and weighted mode) to ensure causal inference stability and reliability, particularly under potential instrument assumption violations while relying primarily on IVW results [31]. To strengthen causal inference within the MR framework, we implemented Steiger testing, a critical methodological step that validates directional validity by confirming stronger correlations between genetic instruments and exposure variables. This comprehensive analytical approach, which combines multiple sensitivity analyses with rigorous methodological validation, provides a robust framework for establishing causality while considering potential MR assumption violations [32].

#### Bidirectional and mediation analysis framework

Reverse MR analysis utilized identical standardized data with independent GWAS outcomes to validate two-sample MR findings. For exposures demonstrating causal relationships with outcomes, we employed multivariable MR analysis to adjust for interactions between related risk factors and exposures, confirming direct causal relationships. To explore the potential causal mechanisms underlying the TL–CRC associations, we conducted two-step MR mediation analysis. Previous studies have established connections between IGF-1, protein markers, and CRC development [33, 34] while demonstrating the TL modulation of these biomarkers [35]. Consequently, we selected 35 blood and urine biomarkers, including IGF-1 and protein markers, as potential mediating variables. Initially, we employed two-sample MR analysis to examine the causal effects of TL on CRC. We subsequently applied two-step MR methodology to assess the potential mediating effects of genuine mediating variables in the causal pathway between forward exposure and CRC. This mediation analysis provided crucial insights into the potential biological pathways associated with TL and CRC risk, thereby deepening the mechanistic understanding of the observed causal relationships.

#### Colocalization analysis implementation

To investigate the potential relationships among TL, biomarkers, and CRC, we performed colocalization analysis via the coloc R package [36]. This analysis identified shared causal variant sites within specific genomic regions that may explain associations between these factors and both phenotypes. The coloc methodology evaluates posterior probabilities for five hypotheses per variant site within a Bayesian framework (H0, H1, H2, H3, H4): (1) no association with either trait; (2) association exclusively with trait 1; (3) association exclusively with trait 2; (4) both traits associated with distinct causal variants; and (5) both traits associated with shared causal variants. The colocalization analysis employed default priors (*p*1 = 1 × 10^−4^, *p*2 = 1 × 10^−4^, *p*12 = 1 × 10^−5^) [37]. The results with a posterior probability for H4 exceeding 80% provided strong evidence that shared causal variants affect gene expression and CRC risk within specific genomic regions.

## Results

### Two-sample MR and reverse MR

**Fig. 2:**
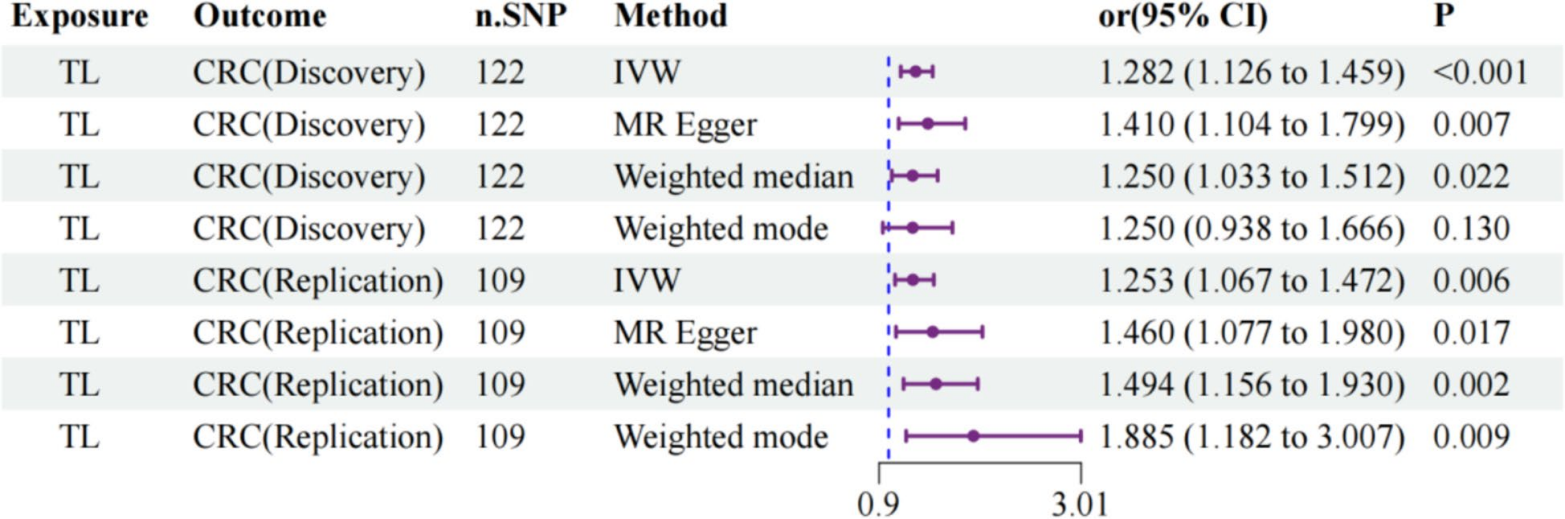
Causal effects of TL on CRC risk: two-sample MR analysis. TL, telomere length; CRC, colorectal cancer; IVW, inverse-variance weighting; MR, Mendelian randomization; OR, odds ratio; CI, confidence interval; n.SNP, number of single nucleotide polymorphisms

Through genome-wide association analysis (*P* < 5 × 10^−8^), we identified significant associations between TL and CRC risk across both the discovery and validation data sets. These genetic instruments demonstrated robust statistical strength, with *F* statistics ranging from 28.65 to 351.92, substantially exceeding conventional weak instrument bias thresholds (Supplementary Table S2). TL was consistently significantly associated with CRC risk in both the discovery and validation cohorts (Supplementary Table S4; Fig. 2). Analysis revealed that in the discovery data set, TL was positively correlated with CRC risk (odds ratio [OR]: 1.282, 95% confidence interval [CI] 1.126–1.459, *P* < 0.001), with consistent significant positive associations observed via MR‒Egger and weighted median methodologies, whereas the weighted mode showed concordant directionality despite not being significant. This pattern was replicated in the validation data set (OR: 1.253, 95% CI 1.067–1.472, *P* = 0.006), with the remaining methodologies similarly demonstrating positive correlations. Both data sets yielded significant results when the MR-PRESSO approach was used (Supplementary Table S4), further confirming the stability of these causal relationships and strengthening confidence in our identified associations.

Comprehensive sensitivity evaluations confirmed the robustness of our findings. Standard pleiotropy assessments and MR-PRESSO analyses revealed no evidence of horizontal pleiotropy (Supplementary Table S5), further validating the reliability of our instrumental variables. Heterogeneity testing demonstrated no significant differences among genetic instruments (Supplementary Table S6), whereas leave-one-out analysis confirmed that the observed associations were not influenced by any impactful outliers (Supplementary Figure S1). Steiger directionality testing consistently supported the hypothesized causal direction, with all instrumental variables passing the directional assessment criteria (Supplementary Table S7). These extensive sensitivity analyses collectively reinforced the robustness of our causal inference framework, providing multifaceted evidence that our findings were not substantially affected by violations of MR assumptions, including pleiotropy, heterogeneity, or reverse causation.

**Fig. 3:**
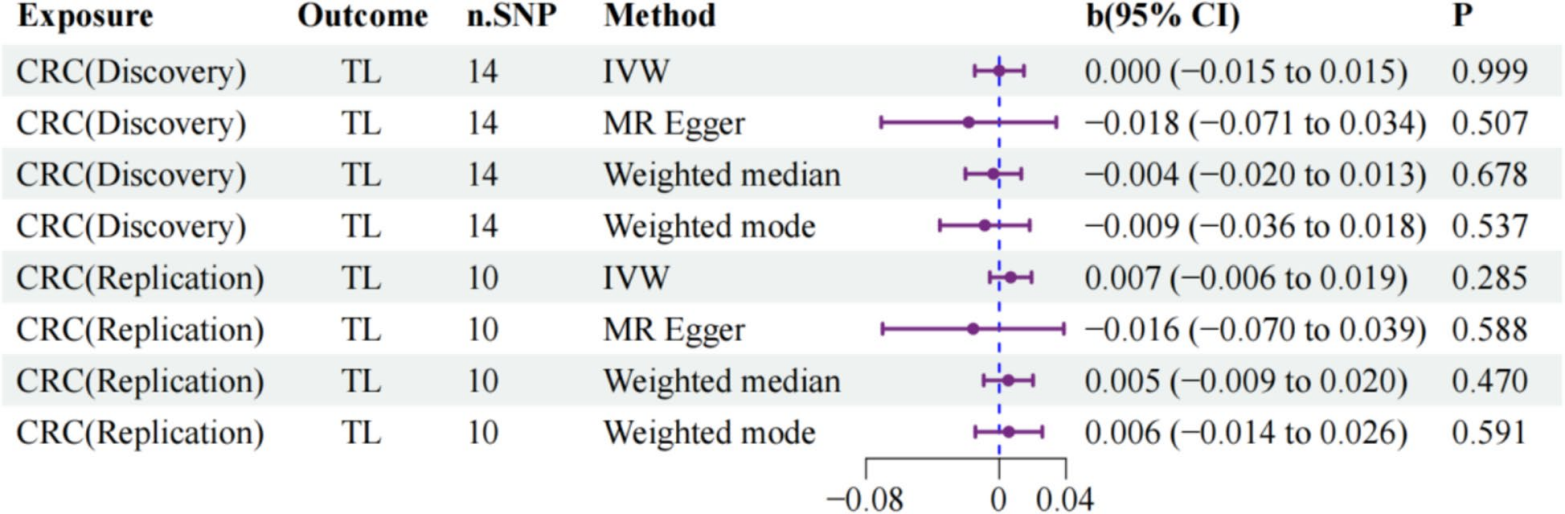
Reverse MR analysis: CRC on TL. TL, telomere length; CRC, colorectal cancer; IVW, inverse-variance weighting; MR, Mendelian randomization; OR, odds ratio; b, beta value; CI, confidence interval; n.SNP, number of single nucleotide polymorphisms

We conducted reverse MR analysis employing SNP selection thresholds consistent with our primary analysis. No evidence of causal relationships between CRC and TL was identified (Fig. 3). The primary results and sensitivity analyses are detailed in Supplementary Tables 4–7, with all effect estimates approaching zero and achieving nonsignificant *P* values across methodological approaches. This absence of reverse causation strengthens the validity of our forward causal inference, supporting the unidirectional nature of the TL–CRC relationship and eliminating concerns regarding bidirectional causality that could confound our primary findings. Additional scatter plots and forest plots for these analyses are presented in Supplementary Figures S2, S3.

### MVMR

**Fig. 4:**
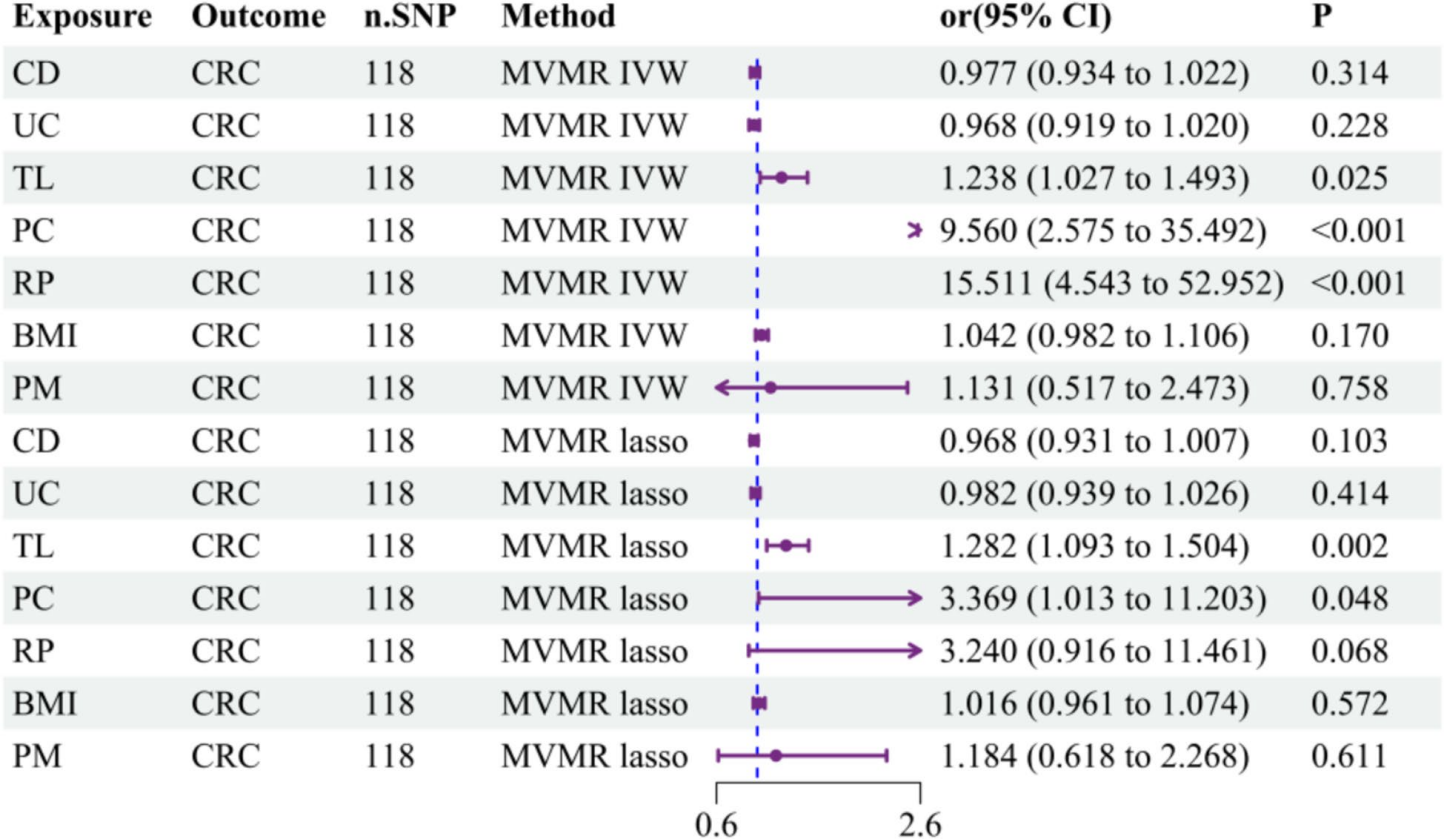
MVMR analysis: independent causal effects of TL on CRC risk. CD, Crohn’s disease; UC, ulcerative colitis; TL, telomere length; PC, polyp of the colon; RP, rectal polyp; CRC, colorectal cancer; BMI, body mass index; PM, processed meat intake; MVMR, multivariable Mendelian randomization; IVW, inverse-variance weighting; n.SNP, number of single nucleotide polymorphisms; OR, odds ratio; CI, confidence interval

To elucidate potential pleiotropic effects and interactions between TL and CRC, we conducted multivariable Mendelian randomization (MVMR) analysis employing two complementary analytical approaches: IVW and LASSO regression methodologies. Given that obesity, processed meat intake, polyposis, and IBD represent established CRC risk factors [20, 38], we incorporated body mass index (BMI), processed meat intake, and four specific conditions (race polyp, polyp of the colon, Crohn’s disease, and ulcerative colitis) into our MVMR framework to minimize their potential confounding influence on our causal estimates. Following adjustment for potential interactions among these six risk factors, our analysis revealed that TL maintained its association with elevated CRC risk. The IVW approach demonstrated a significant promoting effect (OR: 1.238, 95% CI 1.027–1.493, *P* = 0.025), whereas notably, these positive associations remained consistent when the LASSO methodology was used (OR: 1.282, 95% CI 1.093–1.504, *P* = 0.002), with both distinct analytical strategies confirming significant promoting effects. The consistency of these findings was validated through multiple sensitivity analyses, providing robust evidence for the independent causal role of TL in CRC development, even after accounting for potential interactions among these established risk factors (Supplementary Table 8; Fig. 4).

### Two-step MR

**Fig. 5:**
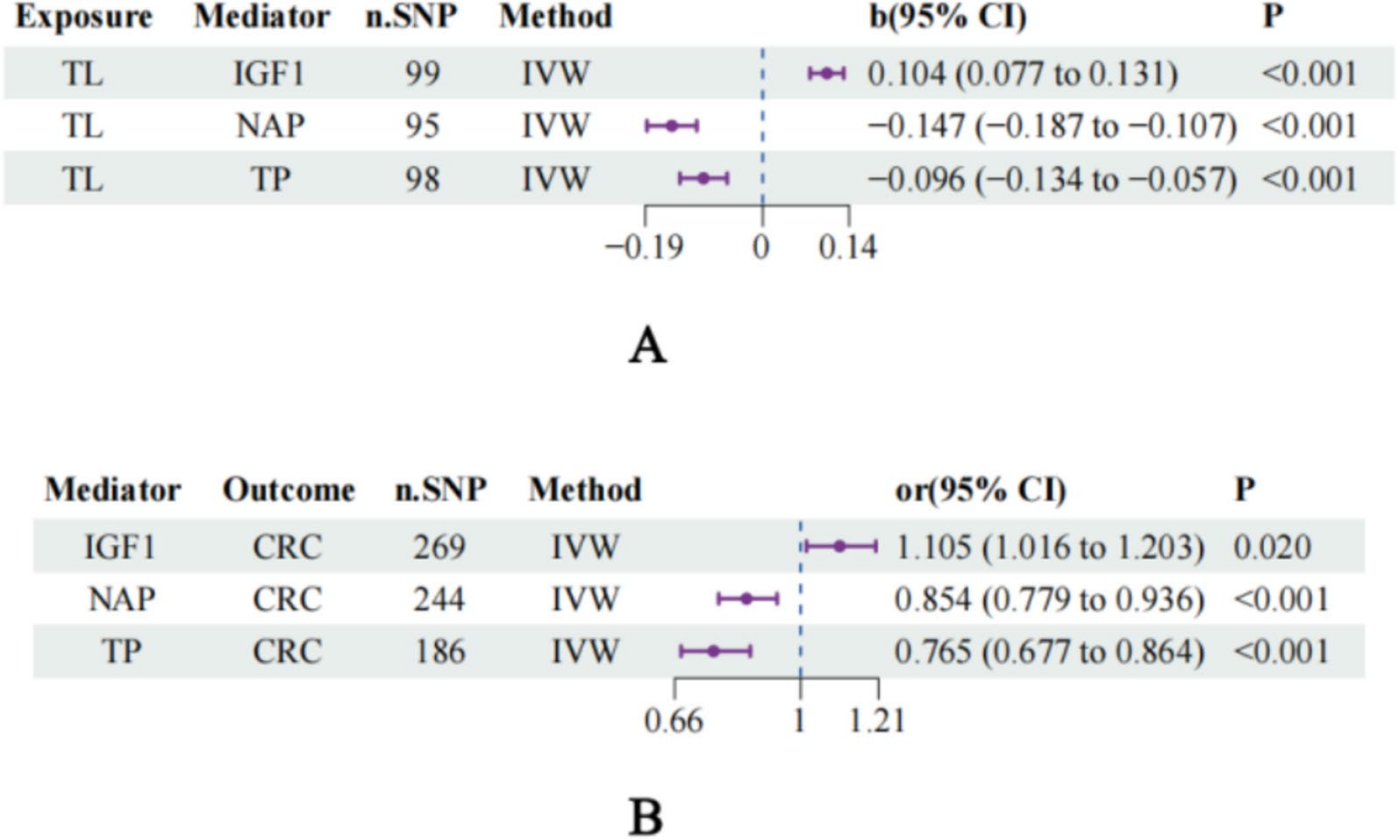
Two-step MR mediation analysis of the TL‒CRC pathway. **A** Causal effects of telomere length on potential mediators (IGF1, NAP, and TP) via the inverse-variance weighted method. The results are presented as beta coefficients with 95% confidence intervals. **B** Causal effects of validated mediators on colorectal cancer risk via the inverse-variance weighted method. The results are presented as odds ratios with 95% confidence intervals. TL, telomere length; IGF1, insulin-like growth factor 1; NAP, nonalbumin protein; TP, total protein; CRC, colorectal cancer; IVW, inverse-variance weighted; n.SNP, number of single nucleotide polymorphisms; b, beta value; CI, confidence interval

Our two-step MR approach successfully identified three genuine mediators in the causal pathway between TL and CRC: insulin-like growth factor 1 (IGF1), nonalbumin protein (NAP), and total protein (TP). Importantly, all three mediators maintained statistical significance after FDR correction for multiple testing, confirming their robustness as true mediating variables. The first step of our analysis revealed significant causal relationships between genetically predicted TLs and these three biomarkers (Fig. 5A). Specifically, longer telomeres were associated with increased IGF1 levels (*β* = 0.104, 95% CI 0.077–0.131, *P* < 0.001), decreased NAP levels (*β* = −0.147, 95% CI −0.187 to –0.107, *P* < 0.001), and reduced total protein levels (*β* = −0.096, 95% CI −0.134 to –0.057, *P* < 0.001).

The second step demonstrated that these biomarkers exerted significant causal effects on CRC risk (Fig. 5B). IGF1 was positively associated with CRC risk (OR = 1.105, 95% CI 1.016–1.203, *P* = 0.020), whereas both NAP (OR = 0.854, 95% CI 0.779–0.936, *P* < 0.001) and total protein (OR = 0.765, 95% CI 0.677–0.864, *P* < 0.001) demonstrated protective effects against CRC development.

Mediation analysis quantified the proportion of the total effect mediated through each pathway (Supplementary Table 9; Fig. 5; Table 1). The results revealed that TP accounted for the largest mediation proportion (10.33%), followed by NAP (9.34%) and IGF1 (4.20%). Collectively, these three biomarkers explained approximately 22.4% of the total causal effect of TL on CRC risk, with the remaining 77.6% representing direct effects or mediation through other unmeasured pathways. The total effect size remained consistent across all the mediation analyses (*β* = 0.2484), confirming the robustness of our findings and providing mechanistic insights into how TL influences CRC development through specific biological intermediates.

**Table 1:** Results of the mediation analysis: quantification of indirect effects on the TL‒CRC pathway

Exposure	Mediator	Outcome	Total effect	Mediator effect	Direct effect	Mediation effect proportion (%)
TL	IGF1	CRC	0.2484	0.0104	0.2380	4.20
TL	NAP	CRC	0.2484	0.0232	0.2252	9.34
TL	TP	CRC	0.2484	0.0257	0.2228	10.33

*TL* telomere length, *IGF1* insulin-like growth factor 1, *NAP* nonalbumin protein, *TP* total protein, *CRC* colorectal cancer

### Colocalization analysis

#### Strong colocalization evidence for TL–biomarker relationships

**Fig. 6:**
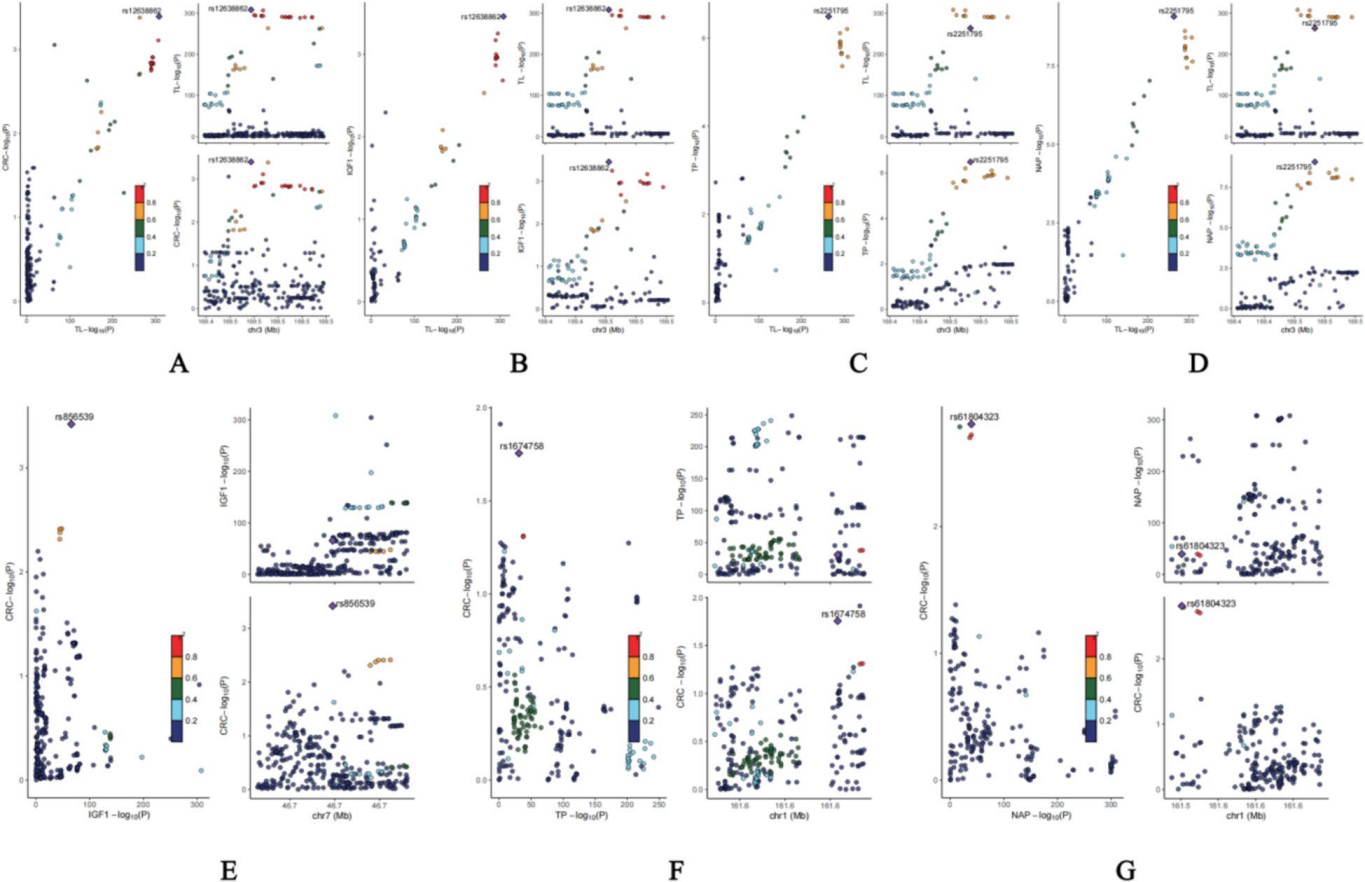
Colocalization analysis results for telomere length-biomarker-colorectal cancer pathways. The stark contrast between **A**–**D** (showing abundant red/warm-colored regions) and **E**–**G** (predominantly blue/cool-colored regions) visually represents the differential colocalization patterns, supporting the conclusion that telomere length shares genetic architecture with the mediating biomarkers but that these biomarkers have limited shared genetic variants with respect to colorectal cancer risk. **A** (TL–CRC): colocalization analysis between telomere length and colorectal cancer risk, showing strong evidence for shared causal variants (PP.H4 = 82.7%) with multiple genomic regions displaying high posterior probabilities for common genetic architecture. **B** (TL–IGF1): colocalization between telomere length and insulin-like growth factor 1 levels, demonstrating robust colocalization evidence (PP.H4 = 90.3%), with several genomic loci showing strong signals for shared causal variants. **C** (TL–TP): colocalization analysis of telomere length and total protein levels, revealing the strongest colocalization signal among all the tested relationships (PP.H4 = 97.1%), with extensive genomic regions showing evidence for shared genetic variants. **D** (TL–NAP): colocalization between telomere length and nonalbumin protein levels, showing strong evidence for shared causal variants (PP.H4 = 95.0%), with multiple genomic loci displaying high posterior probabilities. **E** (IGF1–CRC): colocalization analysis between insulin-like growth factor 1 and colorectal cancer risk, showing minimal evidence for shared causal variants (PP.H4 = 0.8%), with predominantly blue coloring indicating weak colocalization signals. **F** (TP–CRC): colocalization between total protein levels and colorectal cancer risk, demonstrating limited evidence for a shared genetic architecture (PP.H4 = 1.0%) with sparse high-probability regions. **G** (NAP–CRC): colocalization analysis of nonalbumin protein levels and colorectal cancer risk, showing weak evidence for shared causal variants (PP.H4 = 1.4%) with minimal genomic regions displaying strong colocalization signals

Our colocalization analysis revealed compelling evidence for shared causal variants between TL and the three identified mediators (Supplementary Table 10; Fig. 6). The posterior probability for hypothesis H4 (shared causal variants) demonstrated exceptionally strong colocalization signals: TL–TP (PP.H4 = 97.1%), TL–NAP (PP.H4 = 95.0%), and TL–IGF1 (PP.H4 = 90.3%), all substantially exceeding the 80% threshold for robust colocalization evidence. In addition, TL strongly colocalized with CRC risk (PP.H4 = 82.7%), indicating that the shared genetic architecture underlies this causal relationship. These findings provide genomic-level support for the mechanistic pathways identified through our mediation analysis, suggesting that common genetic variants simultaneously influence telomere biology and the regulation of these key biomarkers.

#### Limited colocalization between mediators and CRC

In contrast to the strong colocalization observed between TLs and mediators, we found limited evidence for shared causal variants between individual biomarkers and CRC risk. The posterior probabilities for H4 were notably low: NAP–CRC (PP.H4 = 1.4%), TP–CRC (PP.H4 = 1.0%), and IGF1–CRC (PP.H4 = 0.8%), all falling well below the significance threshold. This pattern suggests that while these biomarkers mediate the causal effects of TL on CRC risk through functional pathways, their individual associations with CRC may be driven primarily by downstream biological processes rather than by shared genetic variants at the genomic level. These results complement our mediation analysis by indicating that the causal chain operates through TL, influencing biomarker levels, which subsequently affect cancer risk through distinct biological mechanisms rather than overlapping genetic architectures.

## Discussion

### Principal findings and novel mechanistic insights

This comprehensive MR study provides robust evidence for a causal relationship between genetically predicted longer TL and increased CRC risk, with consistent findings across discovery (OR = 1.282, 95% CI 1.126–1.459) and replication cohorts (OR = 1.253, 95% CI 1.067–1.472). More importantly, our two-step MR analysis revealed a previously unrecognized dual-pathway mechanism underlying this association, wherein TL influences CRC risk through two opposing biological processes: a procarcinogenic growth signaling pathway mediated by IGF1 (4.20% mediation) and a protective protein metabolism pathway mediated by total protein and nonalbumin protein levels (collectively, 19.67% mediation). These mediating pathways collectively account for 22.4% of the total causal effect, whereas the remaining 77.6% represent direct effects potentially involving other unmeasured biological mechanisms, highlighting the complexity of telomere biology in cancer development.

### Mechanistic plausibility and biological foundations

The identification of IGF1 as a mediator in the telomere–CRC pathway provides crucial mechanistic insights that align with established cancer biology principles. Longer telomeres appear to promote IGF1 production, which subsequently enhances CRC risk through well-characterized oncogenic pathways, including enhanced cell proliferation, apoptosis resistance, and angiogenesis promotion [39, 40]. The IGF1 axis has been extensively implicated in colorectal carcinogenesis, with elevated IGF1 levels associated with increased adenoma formation and malignant transformation [41–43]. Our findings suggest that TL may represent an upstream regulator of this critical growth factor pathway, providing a novel mechanistic link between cellular aging biology and cancer susceptibility.

Conversely, the effects mediated through protein metabolism dysfunction represent a more complex and previously underappreciated mechanism that also contributes to increased CRC risk. Our analysis revealed that longer telomeres are associated with reduced TP and NAP levels, which subsequently increase CRC risk due to the loss of protective effects normally conferred by adequate protein levels. This finding suggests that telomere extension may compromise protein homeostasis in cells with extended replicative capacity, potentially affecting immune surveillance mechanisms, inflammatory responses, and metabolic regulation [44–46]. Since higher protein levels appear to be protective against CRC development (TP → CRC: OR = 0.724; NAP → CRC: OR = 0.728), the telomere-mediated reduction in these protective proteins represents an additional pathway through which longer telomeres promote carcinogenesis. The substantial mediation effect through protein metabolism (19.67%) suggested that telomere-associated alterations in protein synthesis, degradation, or transport may represent a significant biological pathway linking cellular aging to increased cancer risk.

### Resolving the telomere paradox: dual opposing mechanisms

Our findings provide a potential resolution to the long-standing paradox in telomere biology, where epidemiological studies have reported conflicting associations between TL and cancer risk [47–49]. The dual-pathway mechanism identified in this study suggests that longer telomeres exert both procarcinogenic effects (through growth signaling) and protective effects (through protein metabolism alterations), with the net effect determining overall cancer risk. This biological complexity may explain the heterogeneous findings across different studies, populations, and cancer types, as the relative contributions of these opposing pathways may vary depending on genetic background, environmental factors, and tissue-specific biology.

The concept of TL as a double-edged sword in cancer biology is further supported by our colocalization analysis, which demonstrated strong evidence for shared genetic architectures between TL and the identified mediators (PP.H4 > 90% for all telomere–biomarker pairs) but limited colocalization between individual biomarkers and CRC risk (PP.H4 < 2%). This pattern suggests that the causal effects operate through functional biological pathways rather than simple genetic pleiotropy, supporting the mechanistic validity of our mediation analysis. The balance between growth-promoting and growth-protective effects may represent a fundamental feature of telomere biology, with implications extending beyond CRC to other age-related diseases and cancers.

### Interpretation of mediation effect sizes

The observed mediation effects, while individually modest (IGF-1: 4.2%, TP: 9.8%, and NAP: 8.4%), are consistent with the expected magnitude of pathway-specific effects in complex disease genetics. Individual genetic pathways typically contribute 2–5% to disease risk in well-powered studies, and our cumulative mediation of 22.4% represents substantial mechanistic insight for a complex disease outcome. This pattern aligns with the polygenic architecture of cancer susceptibility, where multiple pathways contribute small but meaningful effects that collectively determine disease risk.

### Clinical implications and translational potential

The identification of dual opposing pathways mediated by TL has significant implications for clinical practice and precision medicine approaches for CRC prevention. Individuals with genetically predicted longer telomeres may represent a distinct risk group requiring enhanced surveillance strategies, particularly if they exhibit elevated IGF1 levels or compromised protein metabolism profiles. The development of integrated risk prediction models incorporating TL, IGF1 levels, and protein metabolism markers could enable more precise risk stratification and personalized screening intervals.

From a therapeutic perspective, our findings suggest potential intervention strategies targeting the identified mediating pathways. IGF1 receptor antagonists or IGF1 signaling inhibitors, which are already under investigation for various cancers, may be particularly beneficial for individuals with long telomeres and elevated IGF1 levels [50, 51]. Conversely, interventions aimed at optimizing protein metabolism—including targeted nutritional support, protein supplementation, or therapies addressing protein homeostasis—may help mitigate the protective effects while maintaining the benefits of longer telomeres [52]. Lifestyle modifications that target telomere maintenance, such as regular exercise, stress reduction, and dietary optimization, could be tailored on the basis of individual genetic profiles and biomarker patterns [53, 54].

### Methodological strengths and analytical innovations

This study employs several methodological innovations that enhance the robustness and interpretability of the findings. The two-step MR approach provides a systematic framework for mechanistic discovery, allowing us to identify and quantify specific biological pathways while maintaining the causal inference advantages of MR. The integration of discovery and replication cohorts strengthens the evidence for causality, whereas comprehensive sensitivity analyses—including MR‒Egger, weighted median, MR-PRESSO, and leave-one-out analyses—confirm the stability of our findings across different analytical assumptions.

Colocalization analysis represents a particularly valuable addition, providing genomic-level evidence for the shared genetic architectures underlying telomere–biomarker relationships while distinguishing between genetic pleiotropy and functional mediation. The strong colocalization signals between TLs and the identified mediators (PP.H4 > 90%) provide compelling evidence that these relationships reflect genuine biological pathways rather than statistical artifacts. In addition, the multivariable MR analysis accounting for established CRC risk factors (polyps and inflammatory bowel disease) demonstrated that the telomere‒CRC relationship represents an independent causal pathway rather than a consequence of confounding by known risk factors.

### Limitations and future research directions

Several limitations should be acknowledged in interpreting our findings. First, our analysis is restricted to individuals of European ancestry, limiting the generalizability of findings to other populations with different genetic backgrounds and telomere biology patterns. Second, the identified mediating pathways explain only 22.4% of the total causal effect, suggesting that additional unmeasured biological mechanisms contribute significantly to the telomere–CRC relationship. Third, the cross-sectional nature of biomarker measurements prevents us from capturing the temporal dynamics of telomere-mediated effects, which may be particularly important given the progressive nature of both telomere shortening and carcinogenesis.

Future research should prioritize several key directions to advance our understanding of telomere–cancer relationships. Multiomics integration studies combining genomics, transcriptomics, proteomics, and metabolomics data could identify additional mediating pathways and provide more comprehensive mechanistic insights. Longitudinal studies tracking TL changes and biomarker profiles over time would enable investigations of temporal relationships and critical windows for intervention. Functional validation studies using cell culture models and animal studies are needed to confirm the mechanistic pathways identified through our genetic analysis and to explore potential therapeutic targets.

Population-based studies in diverse ethnic groups would help establish the generalizability of our findings and identify population-specific risk factors. Clinical trials testing interventions targeting the identified pathways—such as IGF1 modulation or protein metabolism optimization—could translate our mechanistic insights into practical prevention strategies. In addition, investigations of telomere–biomarker relationships in other cancer types could determine whether the dual-pathway mechanism represents a general principle of telomere biology or a CRC-specific phenomenon.

### Broader implications for cancer biology and precision medicine

Our findings have far-reaching implications for understanding the role of cellular aging in cancer development and the potential for precision medicine approaches based on genetic risk factors. The demonstration that TL influences cancer risk through multiple opposing biological pathways challenges traditional models of cancer causation and suggests that risk prediction and prevention strategies must account for complex, multipathway mechanisms [55–57]. This complexity may explain why simple biomarker-based approaches for cancer prediction have shown limited success and highlights the need for system-level approaches incorporating multiple biological pathways.

The concept of genetically predicted biomarker levels as mediators of disease risk represents a promising avenue for identifying novel therapeutic targets and developing personalized treatment strategies. By leveraging genetic variation to predict individual susceptibility to specific biological pathways, clinicians could optimize intervention strategies on the basis of each patient’s unique genetic and biomarker profile. This approach could be particularly valuable for cancer prevention, where identifying high-risk individuals and implementing targeted interventions could significantly reduce disease burden.

Furthermore, our findings contribute to the increasing understanding of how aging-related biological processes influence cancer susceptibility. The relationship between TL and cancer risk may represent a model for understanding how other aging-related factors—such as cellular senescence, DNA damage accumulation, and immune system dysfunction—contribute to cancer development through multiple, interconnected pathways. This knowledge could inform the development of antiaging therapies with cancer prevention benefits and guide the optimization of screening strategies for aging populations.

## Conclusion

This comprehensive MR analysis revealed that genetically predicted longer TLs causally increase CRC risk through a dual-pathway mechanism: elevated IGF1-driven growth signaling and compromised protein metabolism via reduced TP and NAP levels. These findings challenge traditional assumptions about the protective role of longer telomeres and provide new mechanistic insights linking cellular aging to cancer development. The identification of these specific mediating pathways offers opportunities for developing targeted prevention strategies and personalized risk assessment approaches. While substantial direct effects remain unexplained, this study establishes a framework for investigating telomere-mediated disease mechanisms and translating genetic insights into clinical applications.

## Supplementary Information

Additional file 1.

Additional file 2.

## Data Availability

All data generated or analyzed in this study are included in this published article [and its supplementary information files] and are available from the corresponding author upon request.
