# Supplementary material for: Telomere length promotes colorectal cancer through dual parallel pathways involving growth signaling and protein metabolism
Source: Trop Med Health. 2025 Dec 24;53:192. doi: 10.1186/s41182-025-00854-x (PMC12729187; doi:10.1186/s41182-025-00854-x)
Supplement: Supplementary file 1 — Additional file 1. [file 41182_2025_854_MOESM1_ESM.pdf]

Supplementary Figure 1. Visualization results of all leave_one_out sensitivity analyses.

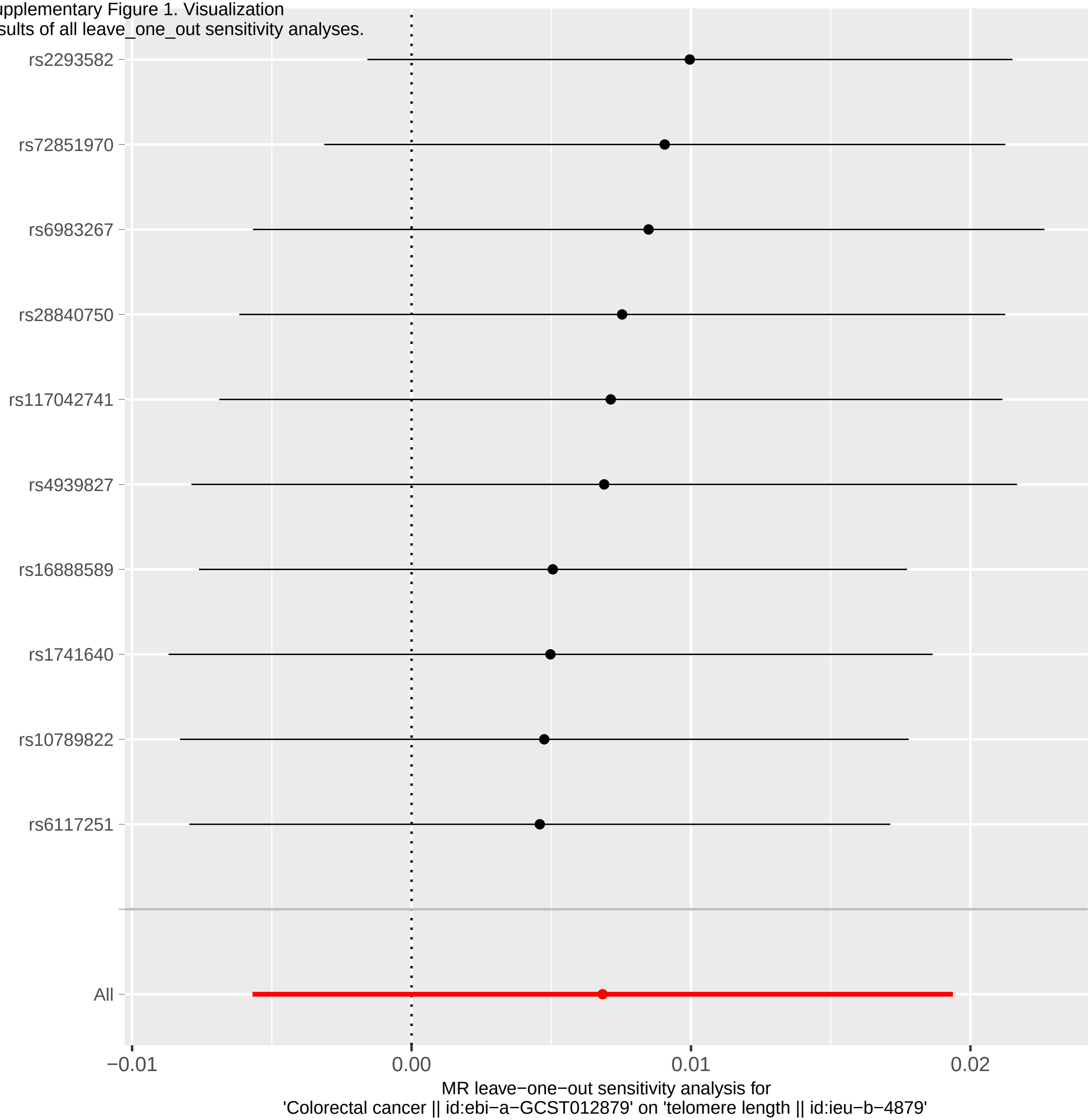

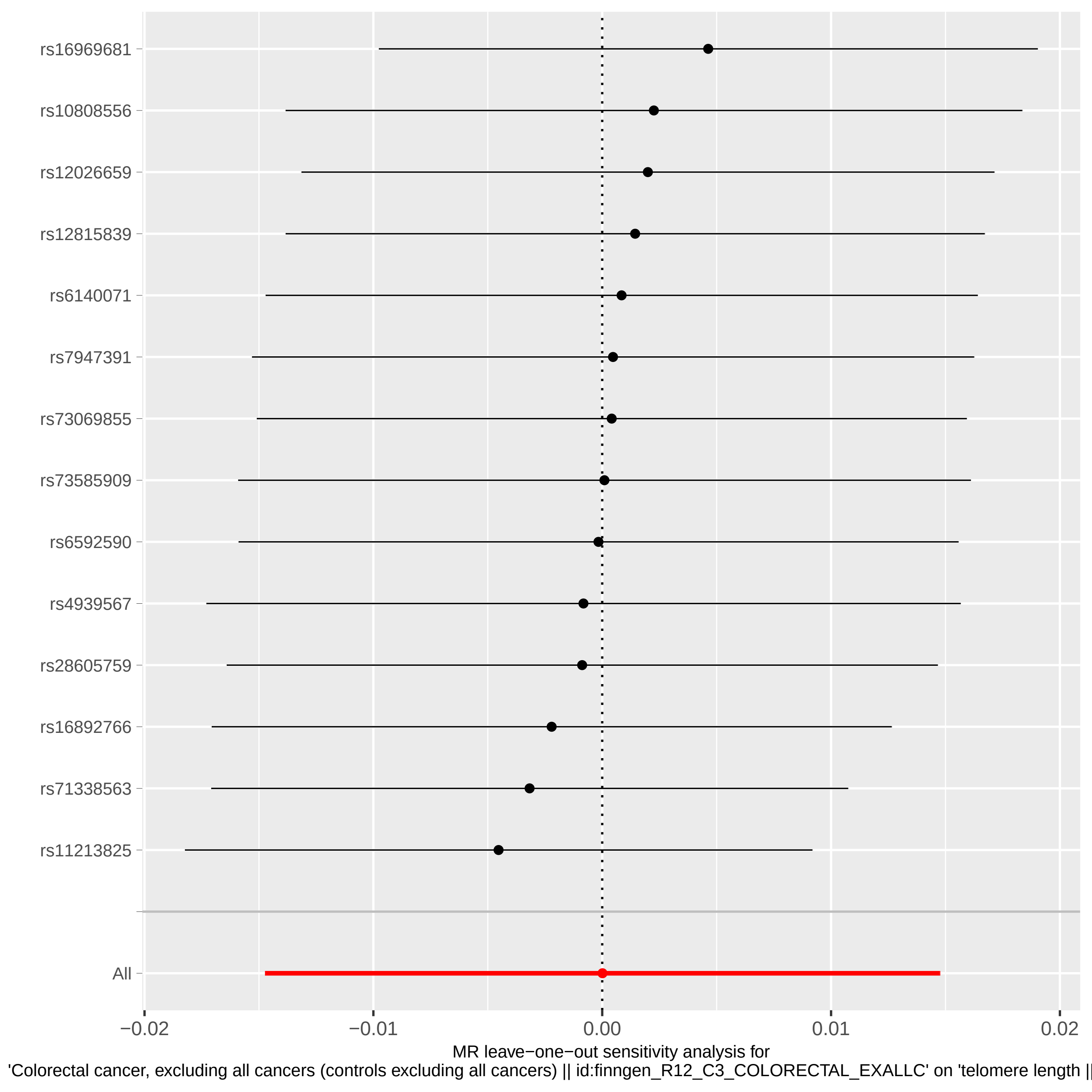
rs16969681
rs10808556
rs12026659
rs12815839
rs6140071
rs7947391
rs73069855
rs73585909
rs6592590
rs4939567
rs28605759
rs16892766
rs71338563
rs11213825
All
−0.02
−0.01
0.00
0.01
0.02
MR leave−one−out sensitivity analysis for
'Colorectal cancer, excluding all cancers (controls excluding all cancers) || id:finngen_R12_C3_COLORECTAL_EXALLC' on 'telomere length ||

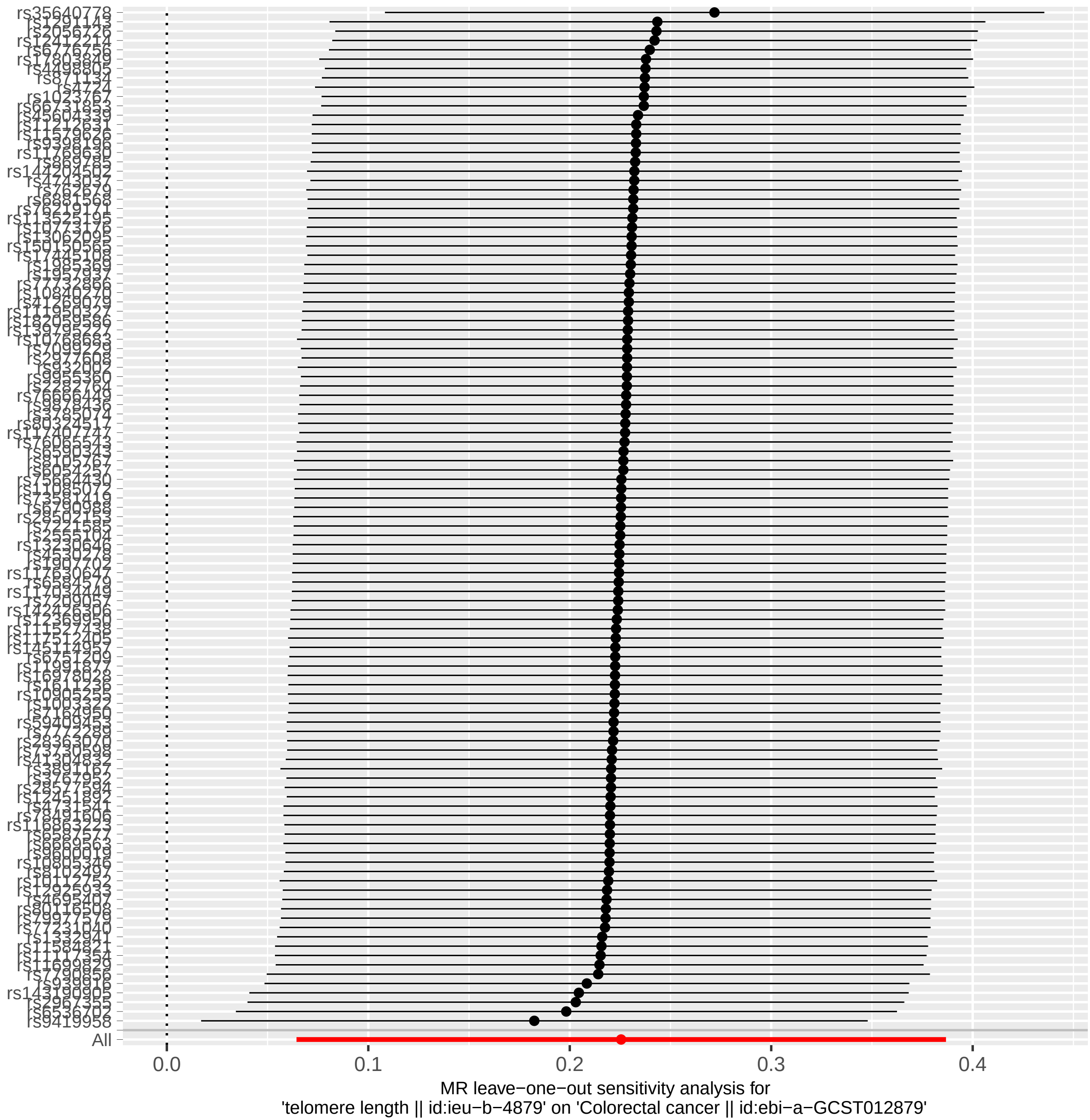

rs35640778
rs9419958
All
0.0
0.1
0.2
0.3
0.4
MR leave−one−out sensitivity analysis for
'telomere length || id:ieu−b−4879' on 'Colorectal cancer || id:ebi−a−GCST012879'

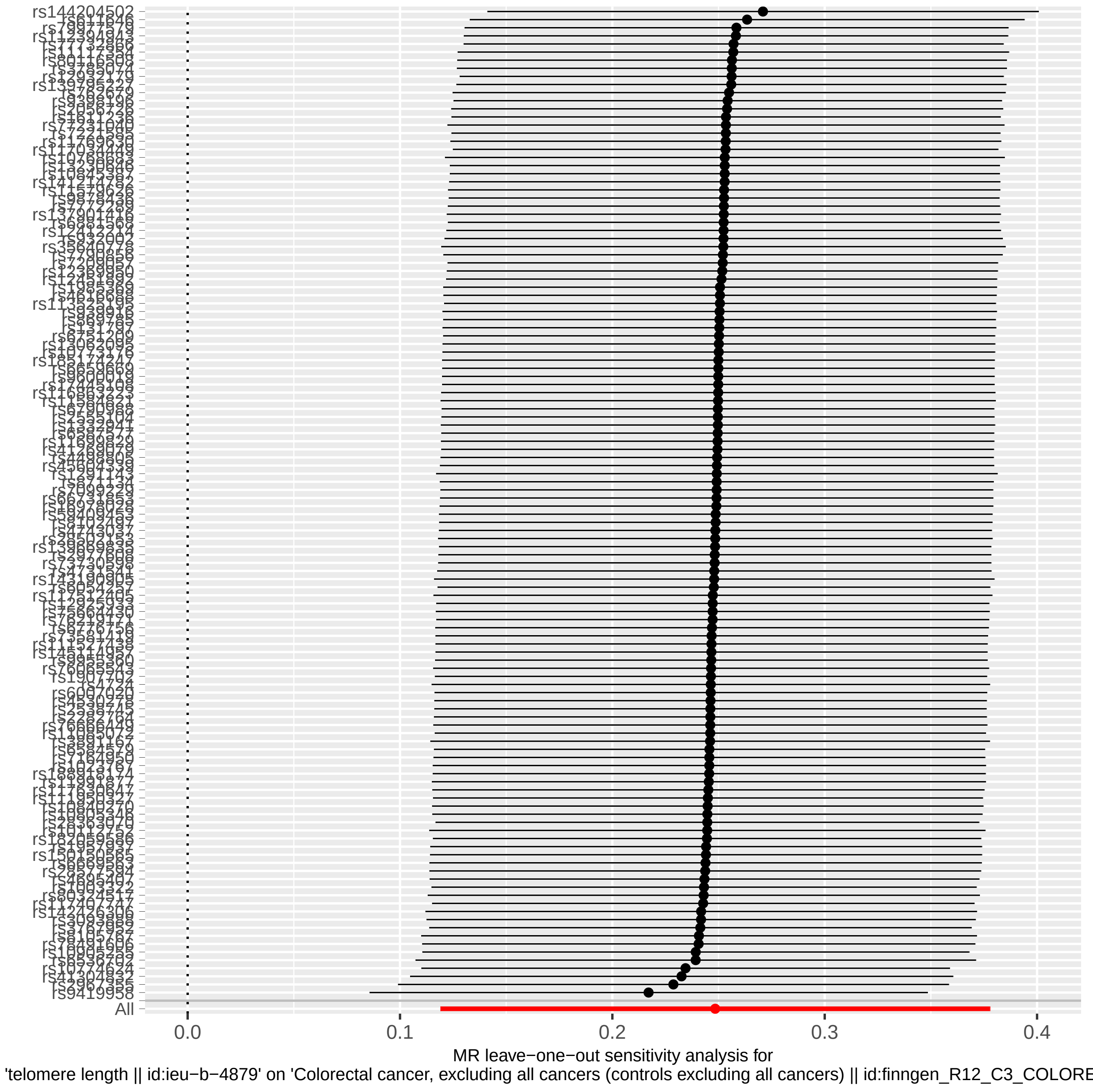

rs144204502
rs9419958
All
0.0
0.1
0.2
0.3
0.4
MR leave−one−out sensitivity analysis for
'telomere length || id:ieu−b−4879' on 'Colorectal cancer, excluding all cancers (controls excluding all cancers) || id:finngen_R12_C3_COLORE

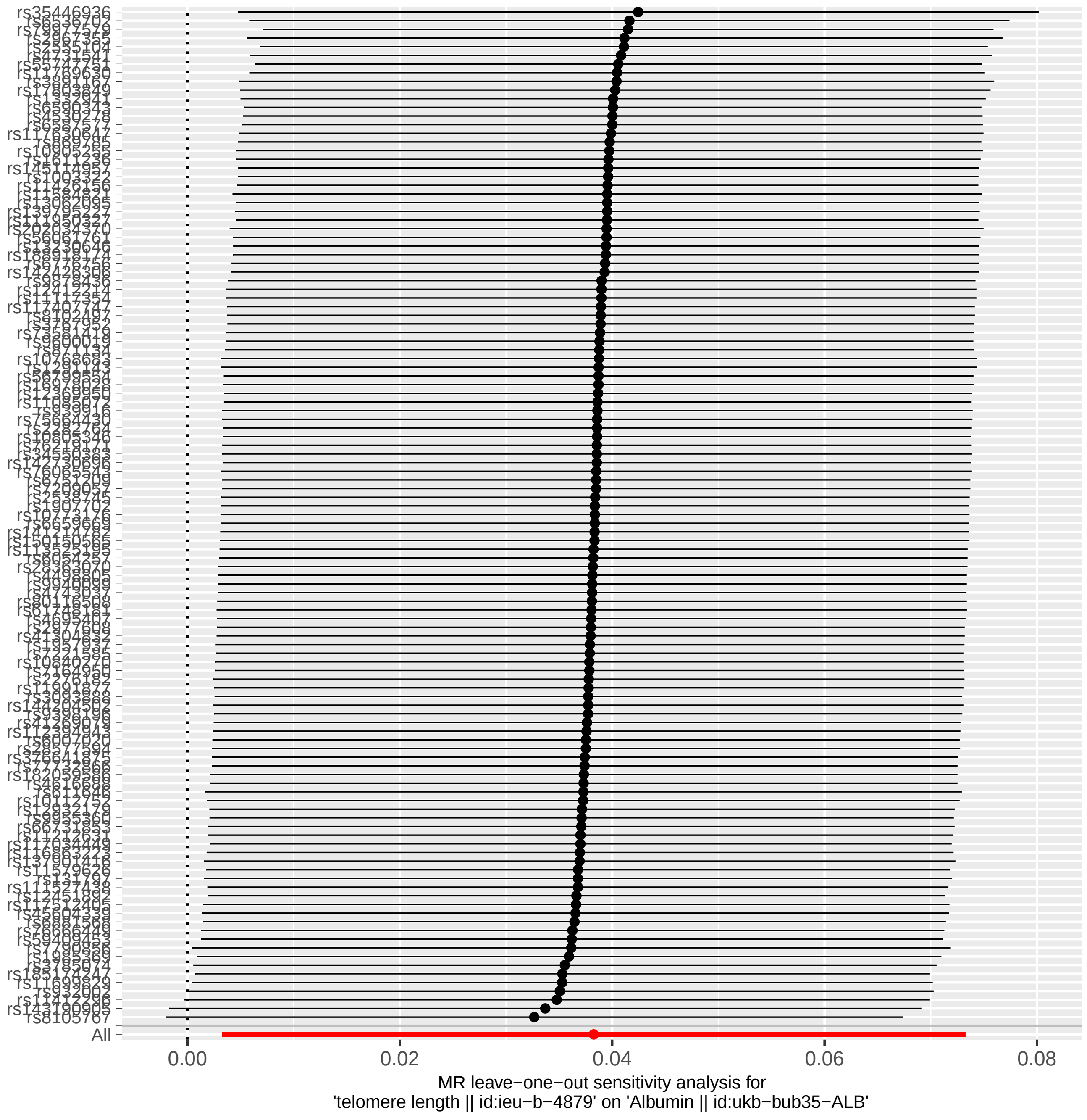
rs35446936
rs8105767
All
0.00
0.02
0.04
0.06
0.08
MR leave−one−out sensitivity analysis for
'telomere length || id:ieu−b−4879' on 'Albumin || id:ukb−bub35−ALB'

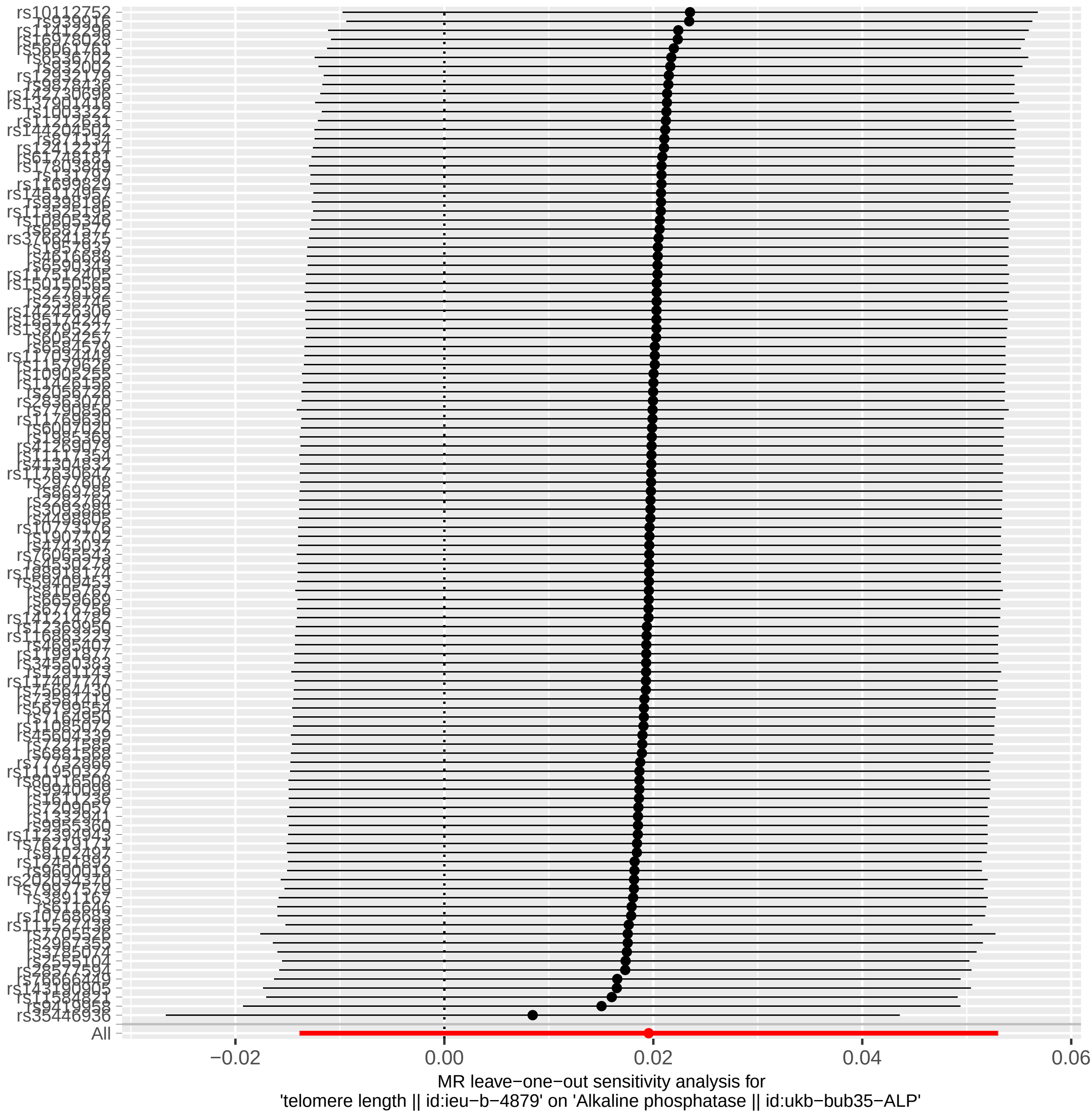
rs10112752
rs35446936
All
−0.02
0.00
0.02
0.04
0.06
MR leave−one−out sensitivity analysis for
'telomere length || id:ieu−b−4879' on 'Alkaline phosphatase || id:ukb−bub35−ALP'

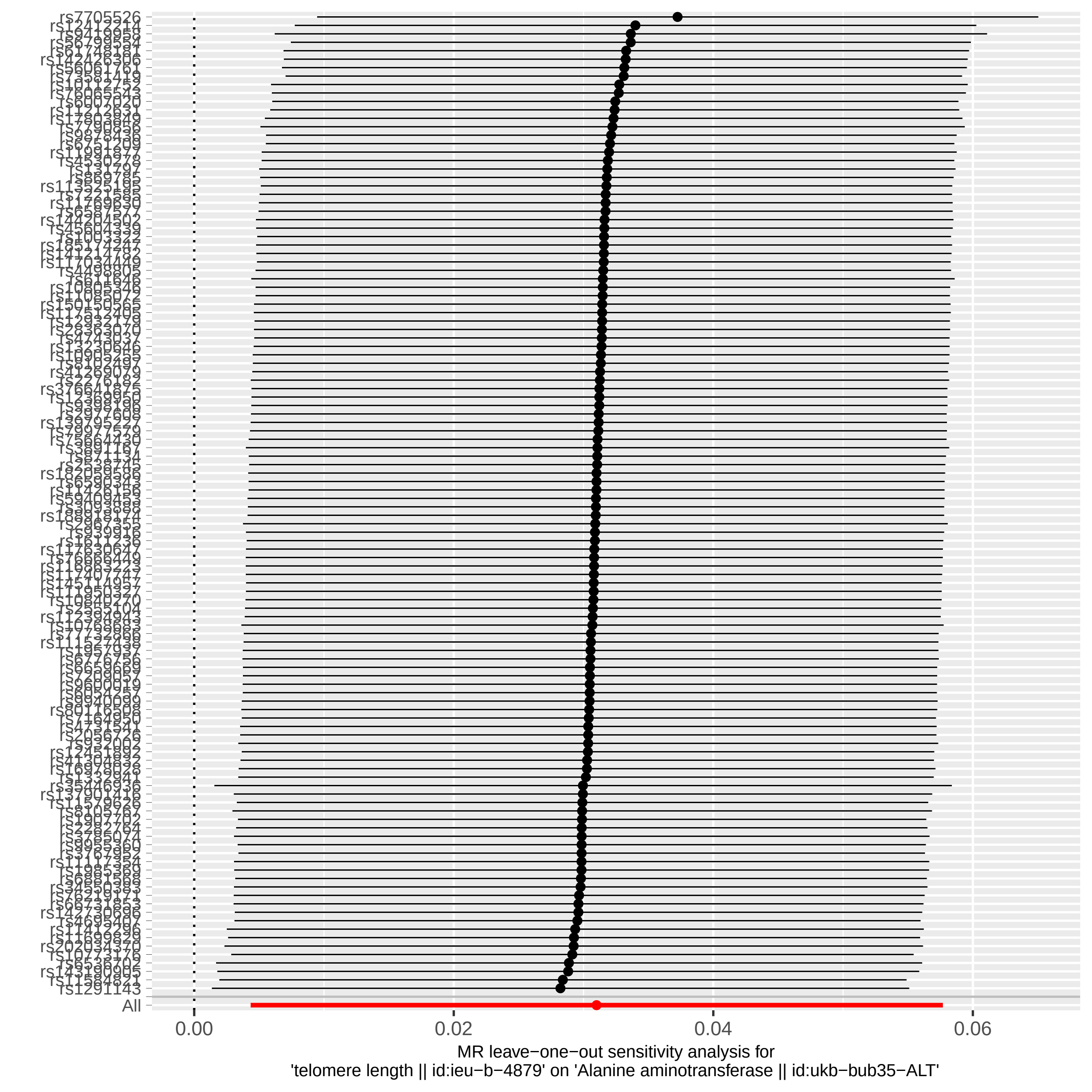

rs7705526
rs1291143
All
0.00
0.02
0.04
0.06
MR leave−one−out sensitivity analysis for
'telomere length || id:ieu−b−4879' on 'Alanine aminotransferase || id:ukb−bub35−ALT'

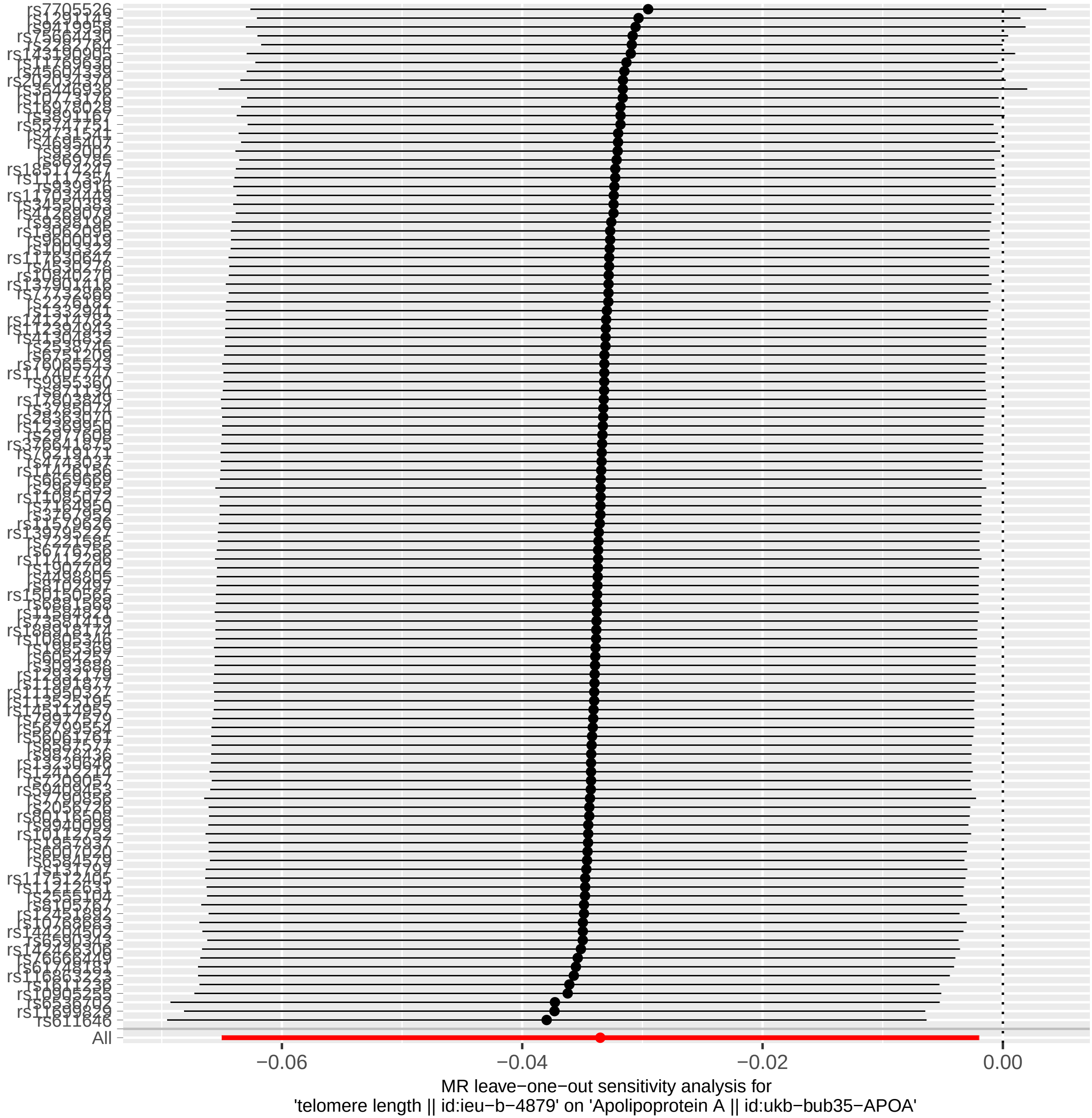

rs7705526
rs611646
All
−0.06
−0.04
−0.02
0.00
MR leave−one−out sensitivity analysis for
'telomere length || id:ieu−b−4879' on 'Apolipoprotein A || id:ukb−bub35−APOA'

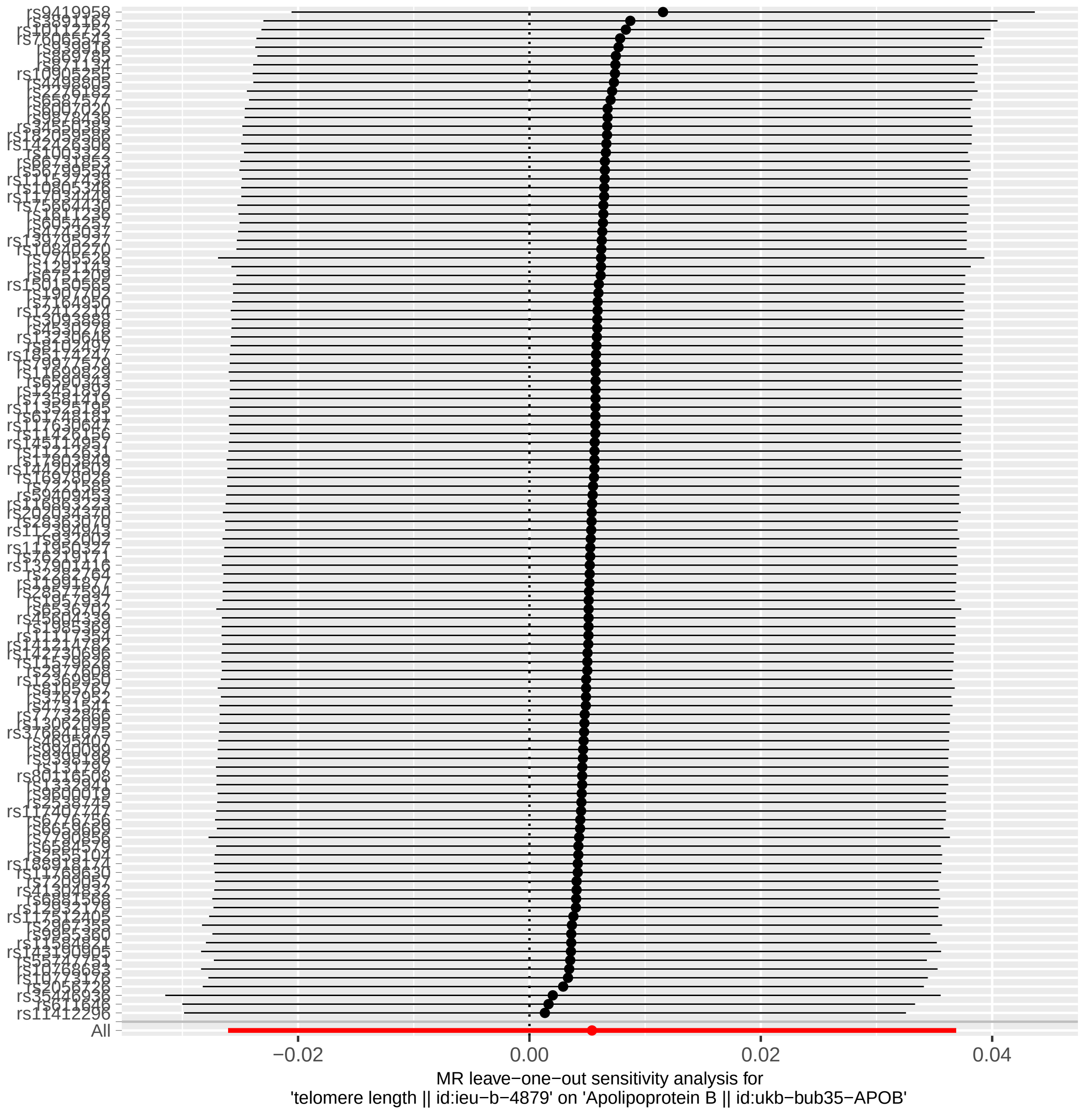
rs9419958
rs11412296
All
−0.02
0.00
0.02
0.04
MR leave−one−out sensitivity analysis for
'telomere length || id:ieu−b−4879' on 'Apolipoprotein B || id:ukb−bub35−APOB'

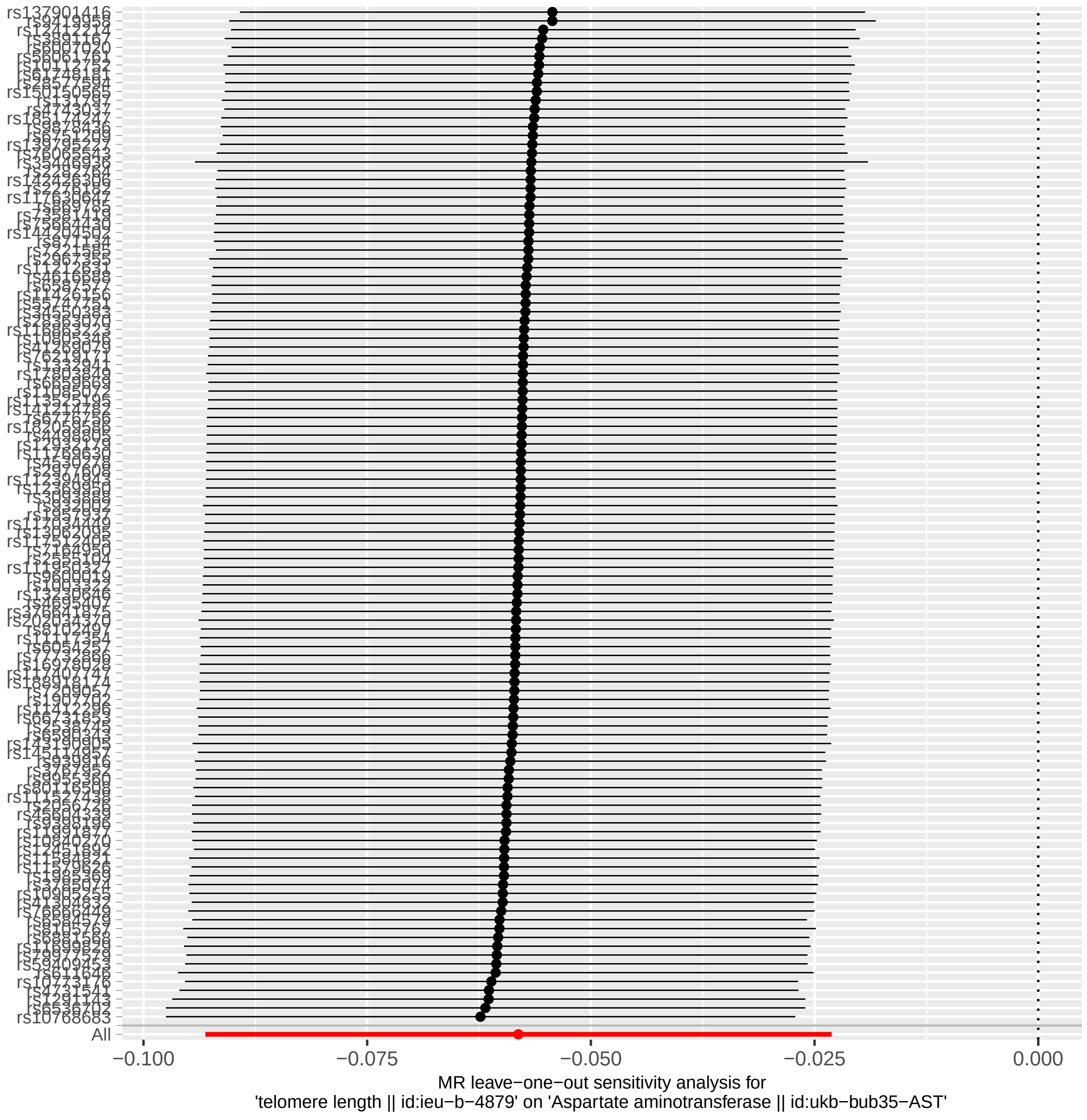

rs137901416
rs10768683
All
−0.100
−0.075
−0.050
−0.025
0.000
MR leave−one−out sensitivity analysis for
'telomere length || id:ieu−b−4879' on 'Aspartate aminotransferase || id:ukb−bub35−AST'

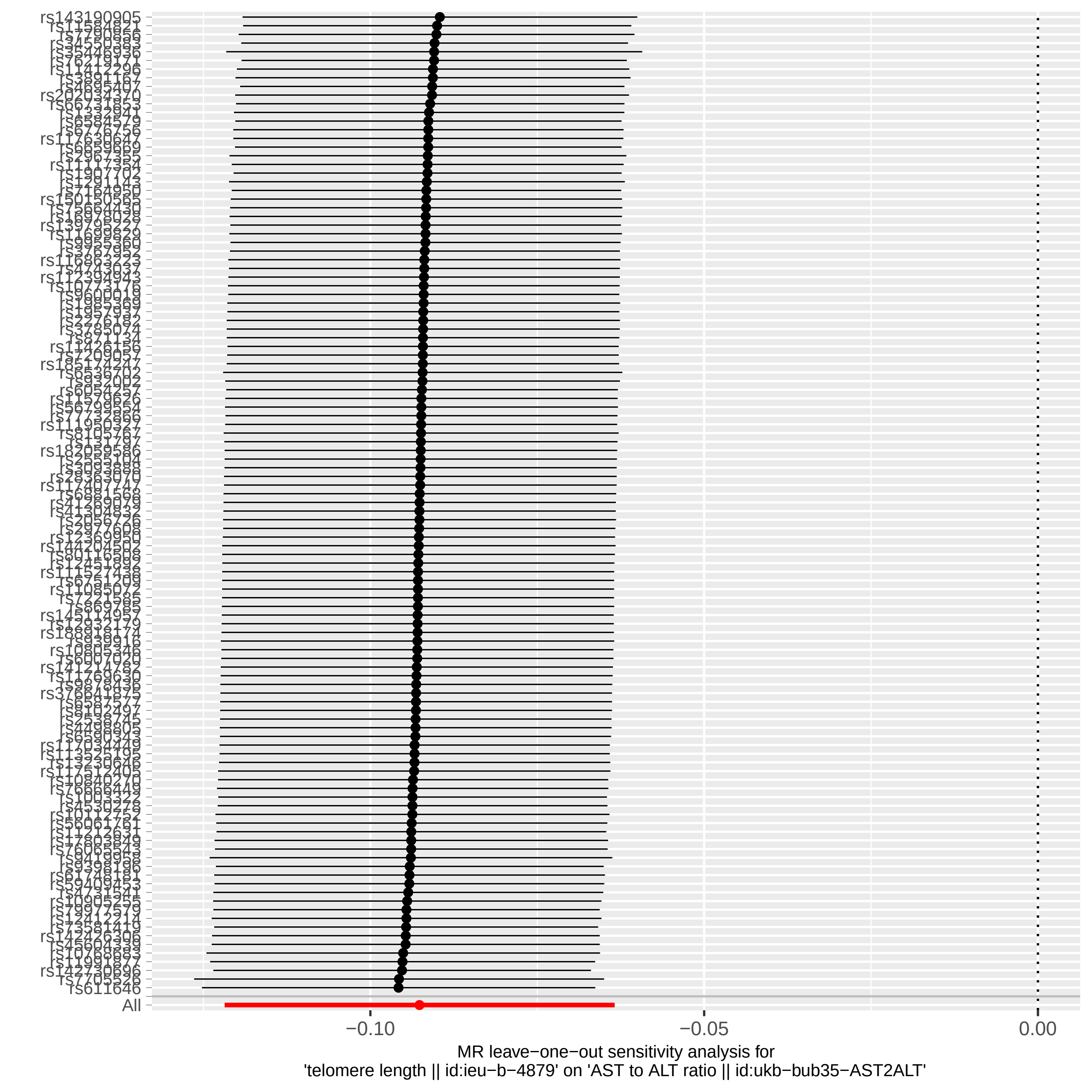

rs143190905
rs611646
All
−0.10
−0.05
0.00
MR leave−one−out sensitivity analysis for
'telomere length || id:ieu−b−4879' on 'AST to ALT ratio || id:ukb−bub35−AST2ALT'

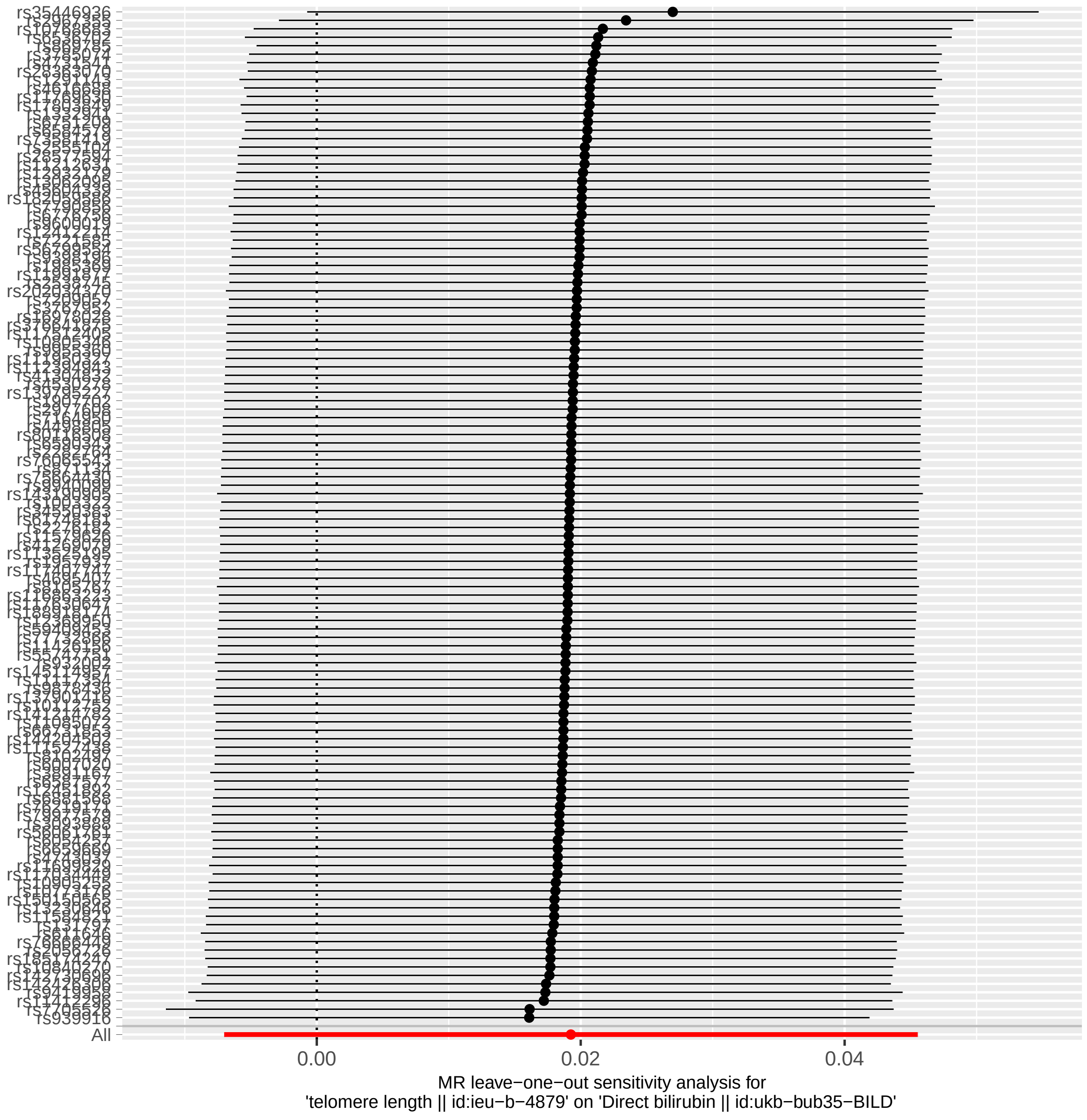
rs35446936
rs939916
All
0.00
0.02
0.04
MR leave−one−out sensitivity analysis for
'telomere length || id:ieu−b−4879' on 'Direct bilirubin || id:ukb−bub35−BILD'

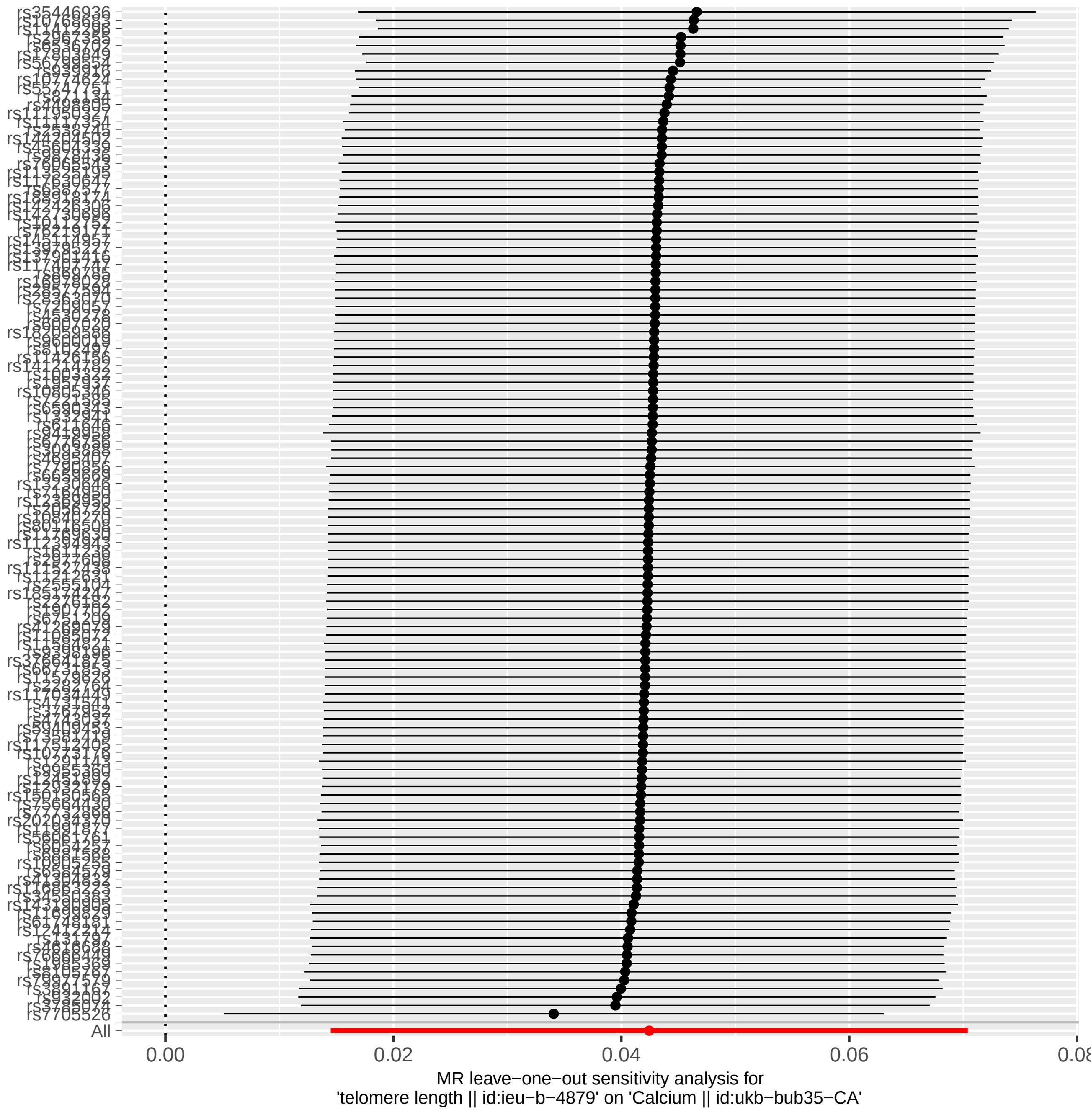

rs35446936
rs7705526
All
0.00
0.02
0.04
0.06
0.08
MR leave−one−out sensitivity analysis for
'telomere length || id:ieu−b−4879' on 'Calcium || id:ukb−bub35−CA'

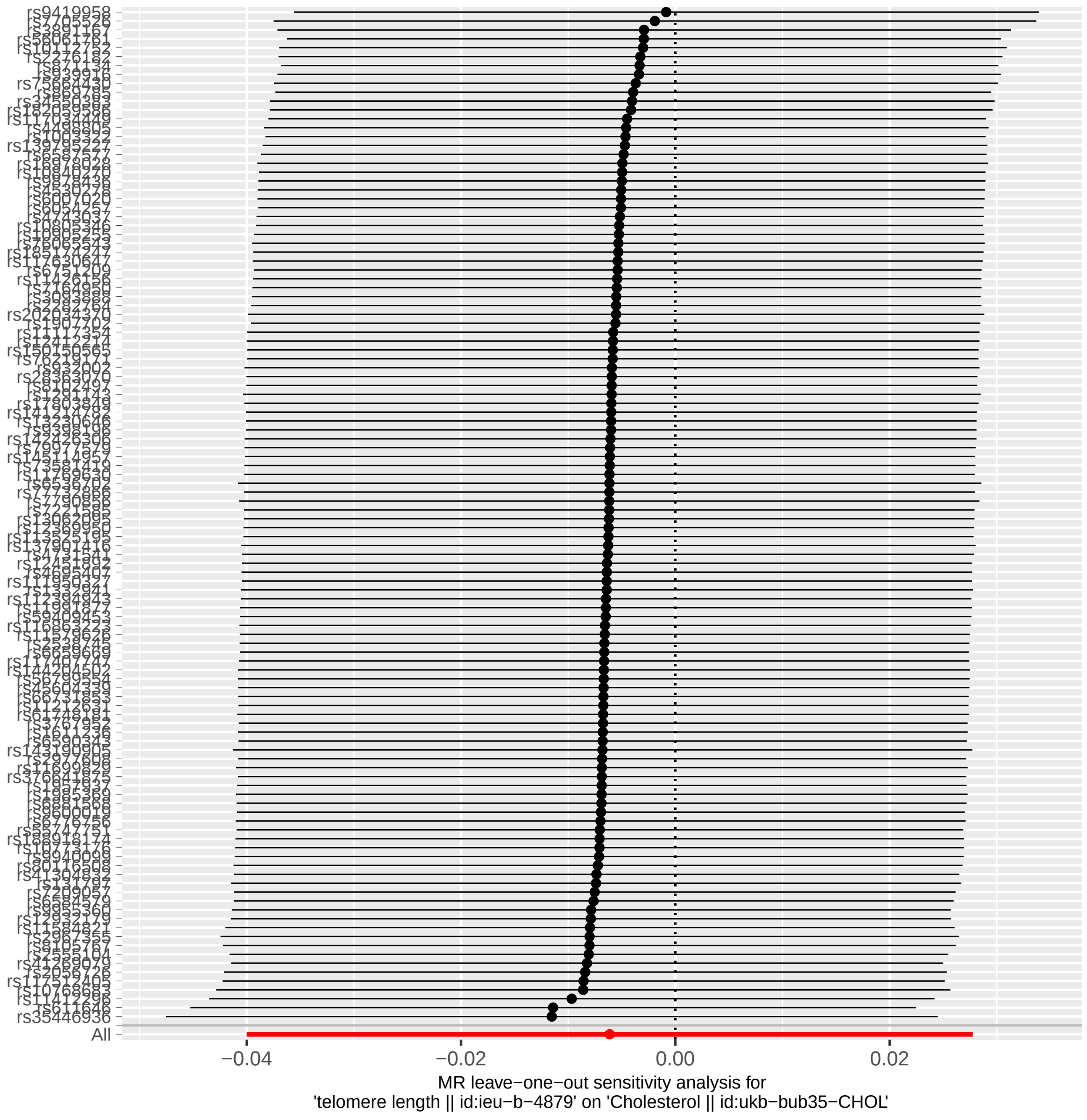
rs9419958
rs35446936
All
−0.04
−0.02
0.00
0.02
MR leave−one−out sensitivity analysis for
'telomere length || id:ieu−b−4879' on 'Cholesterol || id:ukb−bub35−CHOL'

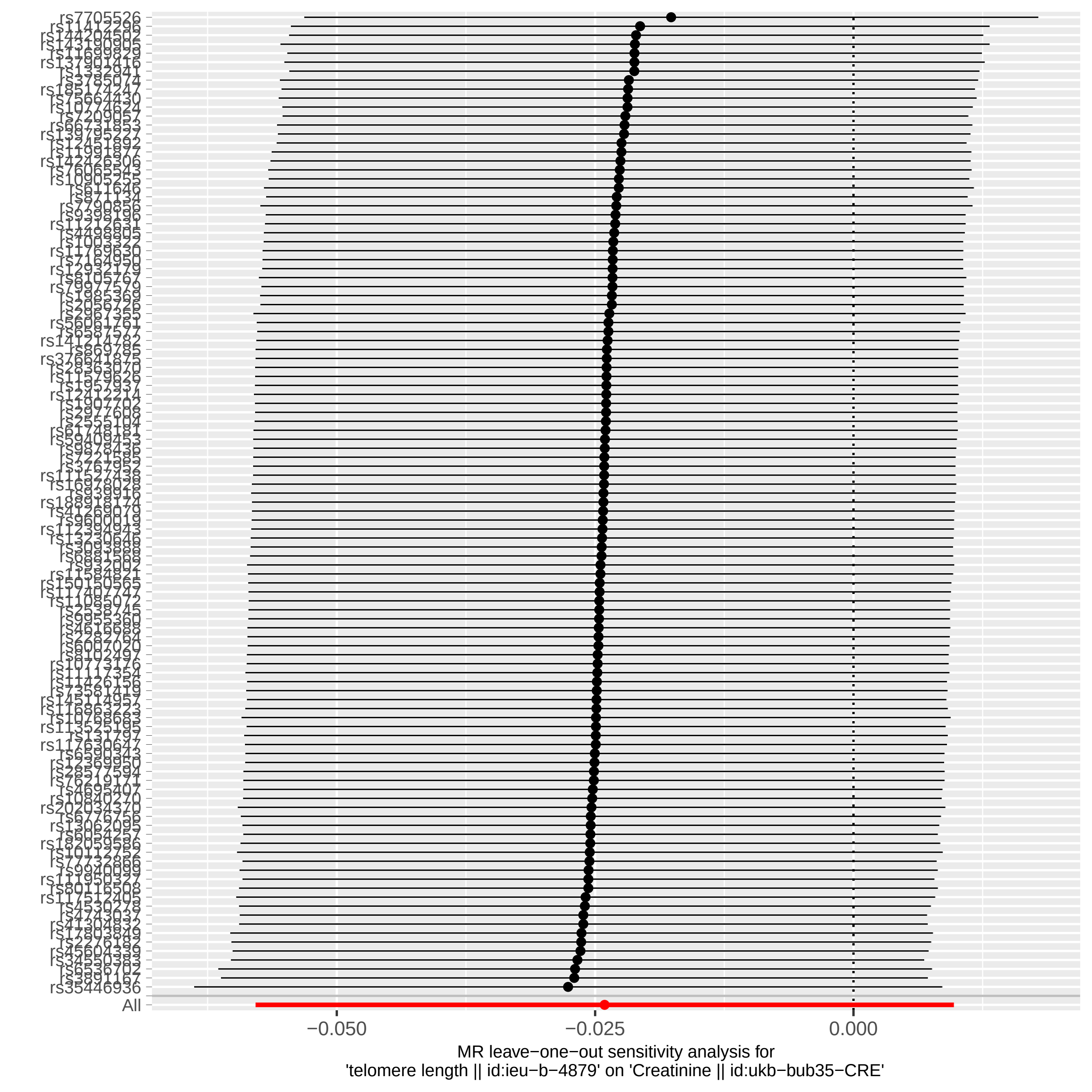
rs7705526
rs35446936
All
−0.050
−0.025
0.000
MR leave−one−out sensitivity analysis for
'telomere length || id:ieu−b−4879' on 'Creatinine || id:ukb−bub35−CRE'

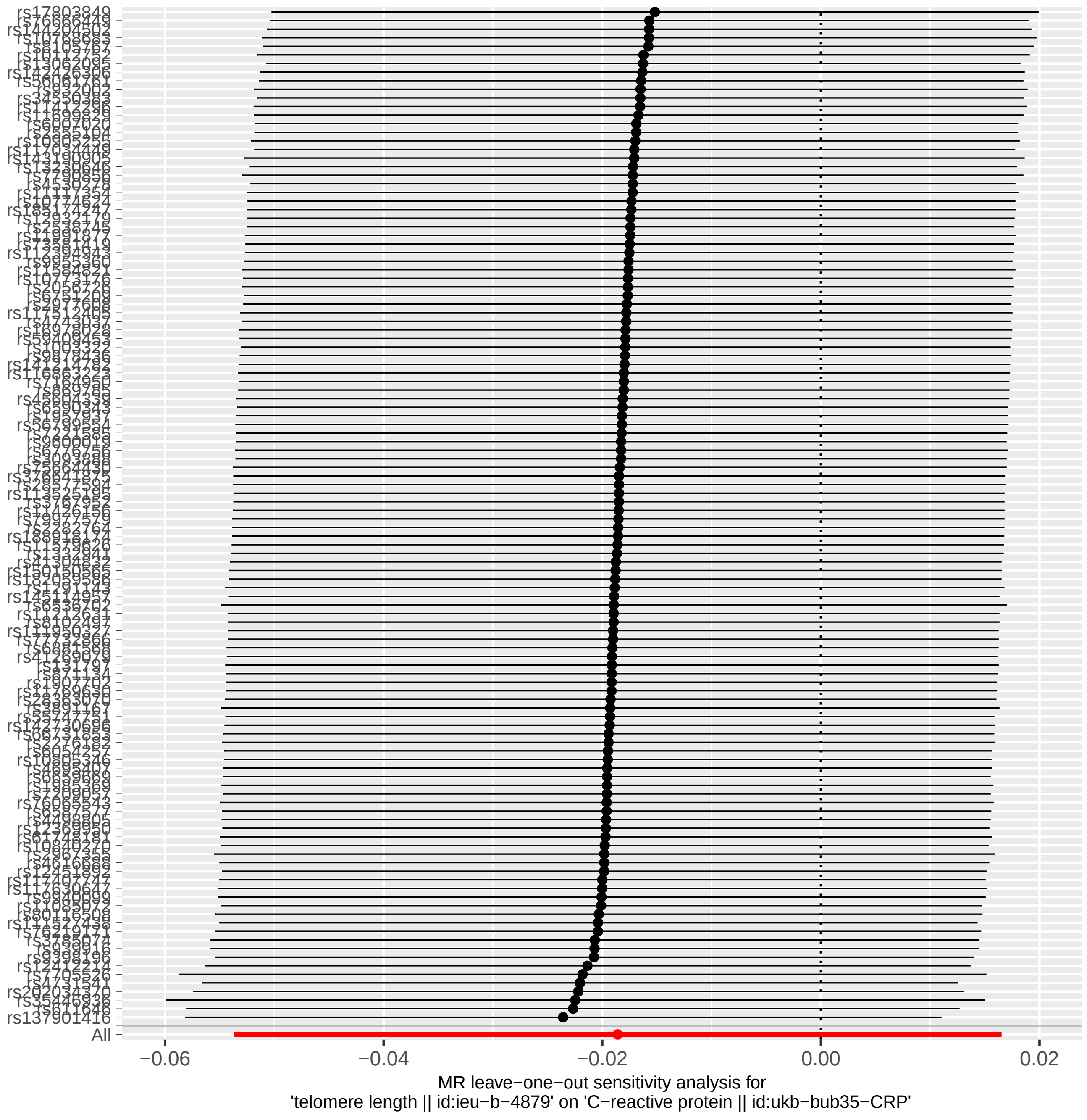

rs17803849
rs137901416
All
−0.06
−0.04
−0.02
0.00
0.02
MR leave−one−out sensitivity analysis for
'telomere length || id:ieu−b−4879' on 'C−reactive protein || id:ukb−bub35−CRP'

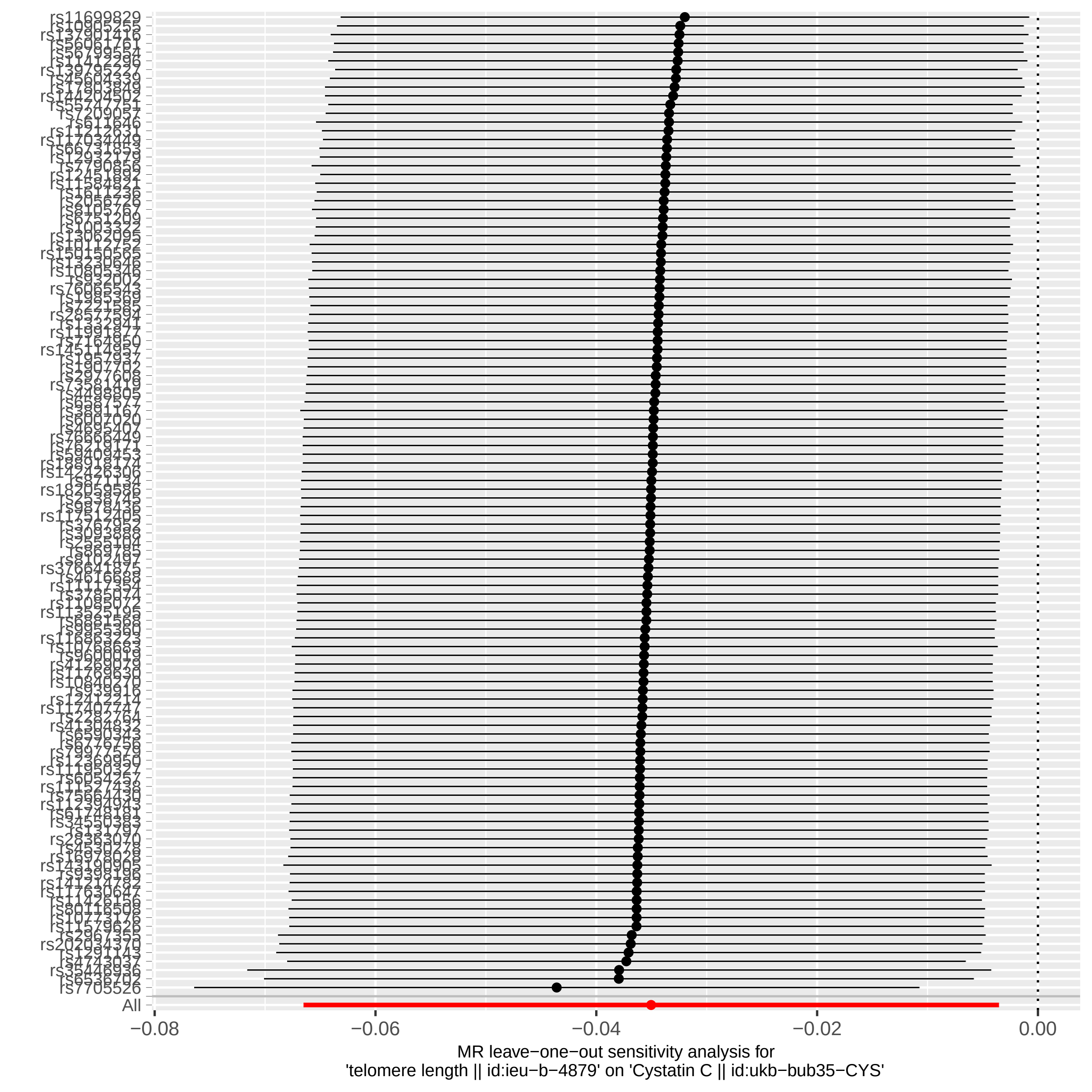
All
−0.08
−0.06
−0.04
−0.02
0.00
MR leave−one−out sensitivity analysis for
'telomere length || id:ieu−b−4879' on 'Cystatin C || id:ukb−bub35−CYS'

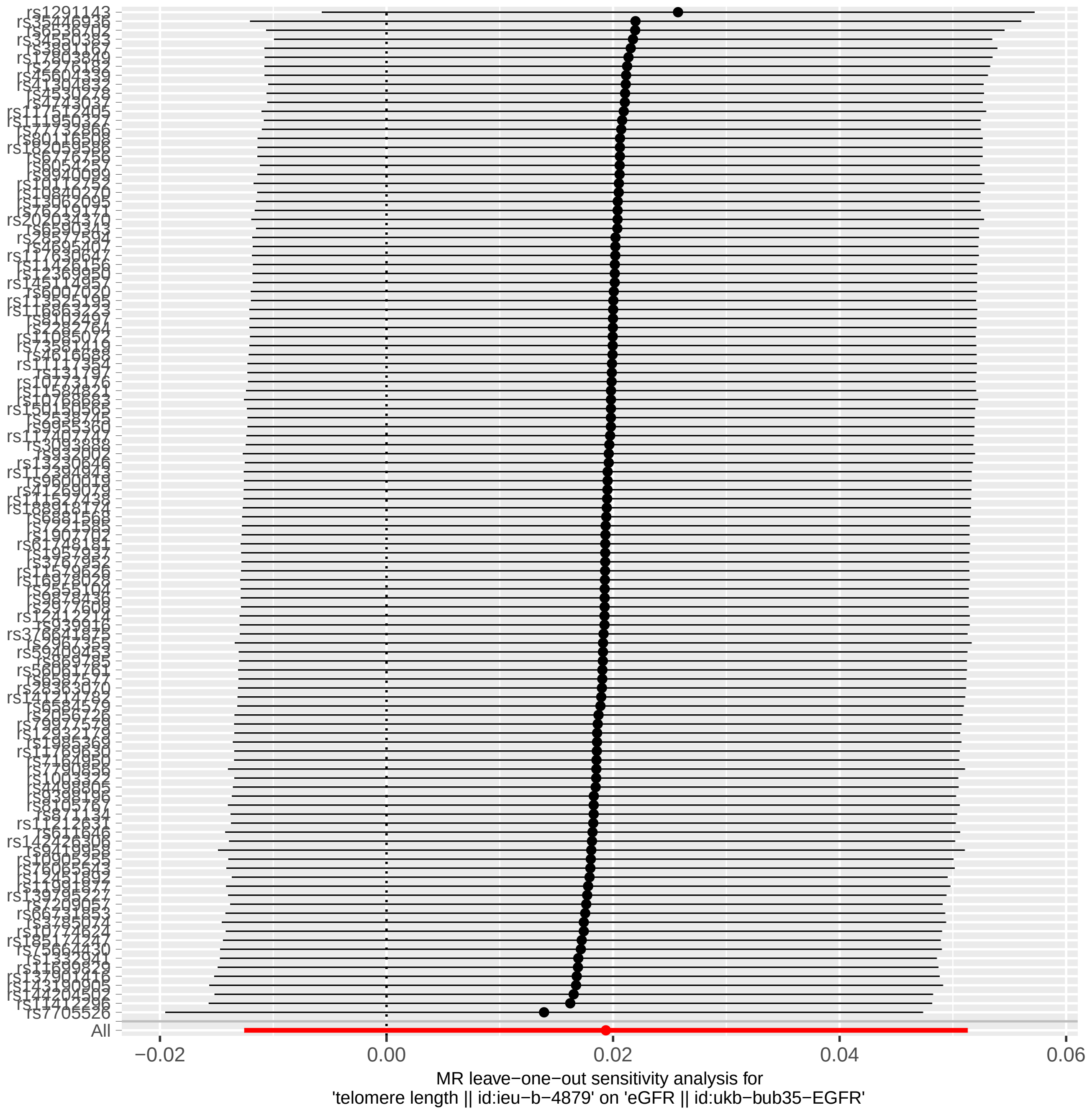
rs1291143
rs7705526
All
−0.02
0.00
0.02
0.04
0.06
MR leave−one−out sensitivity analysis for
'telomere length || id:ieu−b−4879' on 'eGFR || id:ukb−bub35−EGFR'

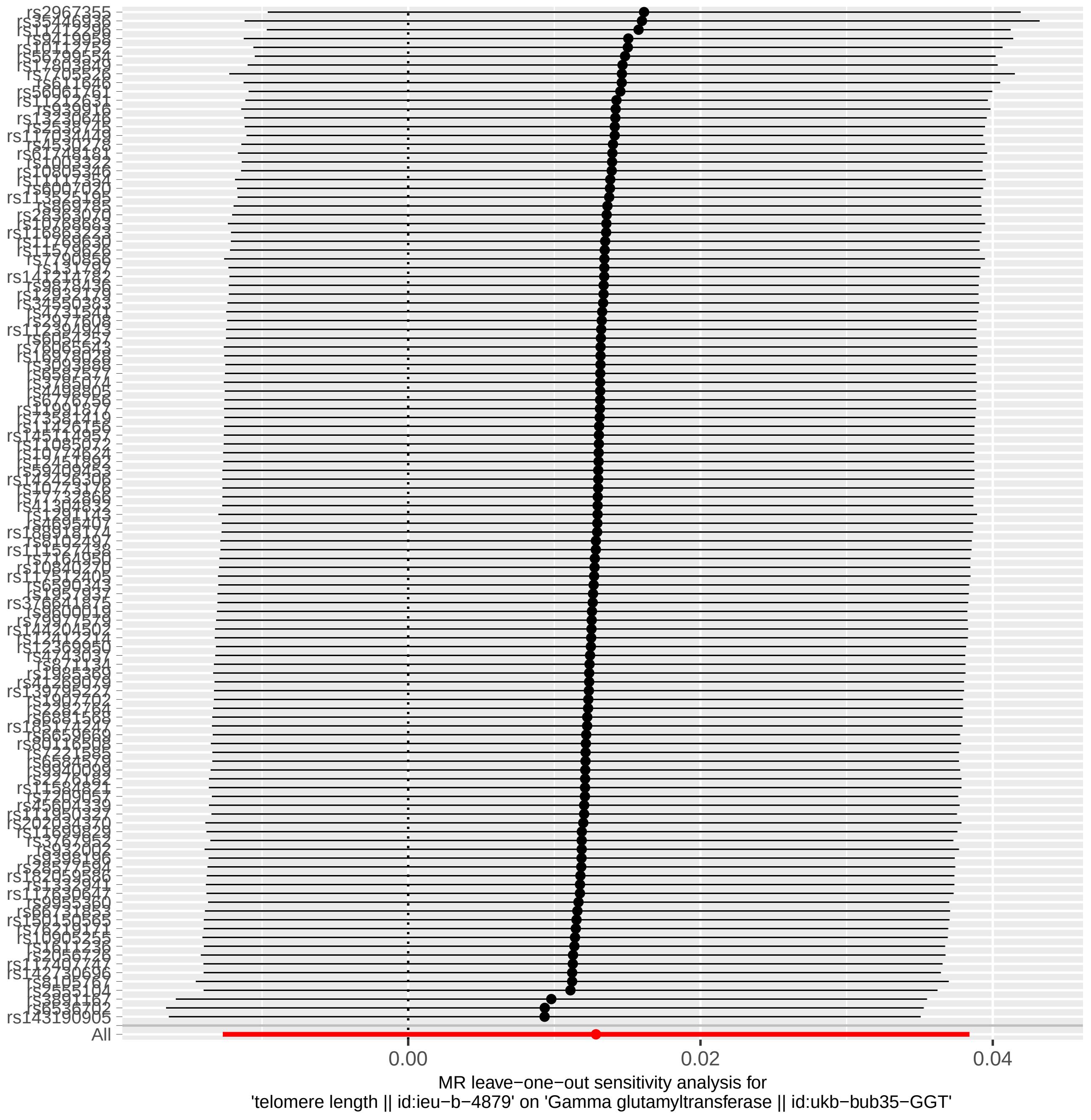
rs2967355
rs143190905
All
0.00
0.02
0.04
MR leave−one−out sensitivity analysis for
'telomere length || id:ieu-b-4879' on 'Gamma glutamyltransferase || id:ukb-bub35-GGT'

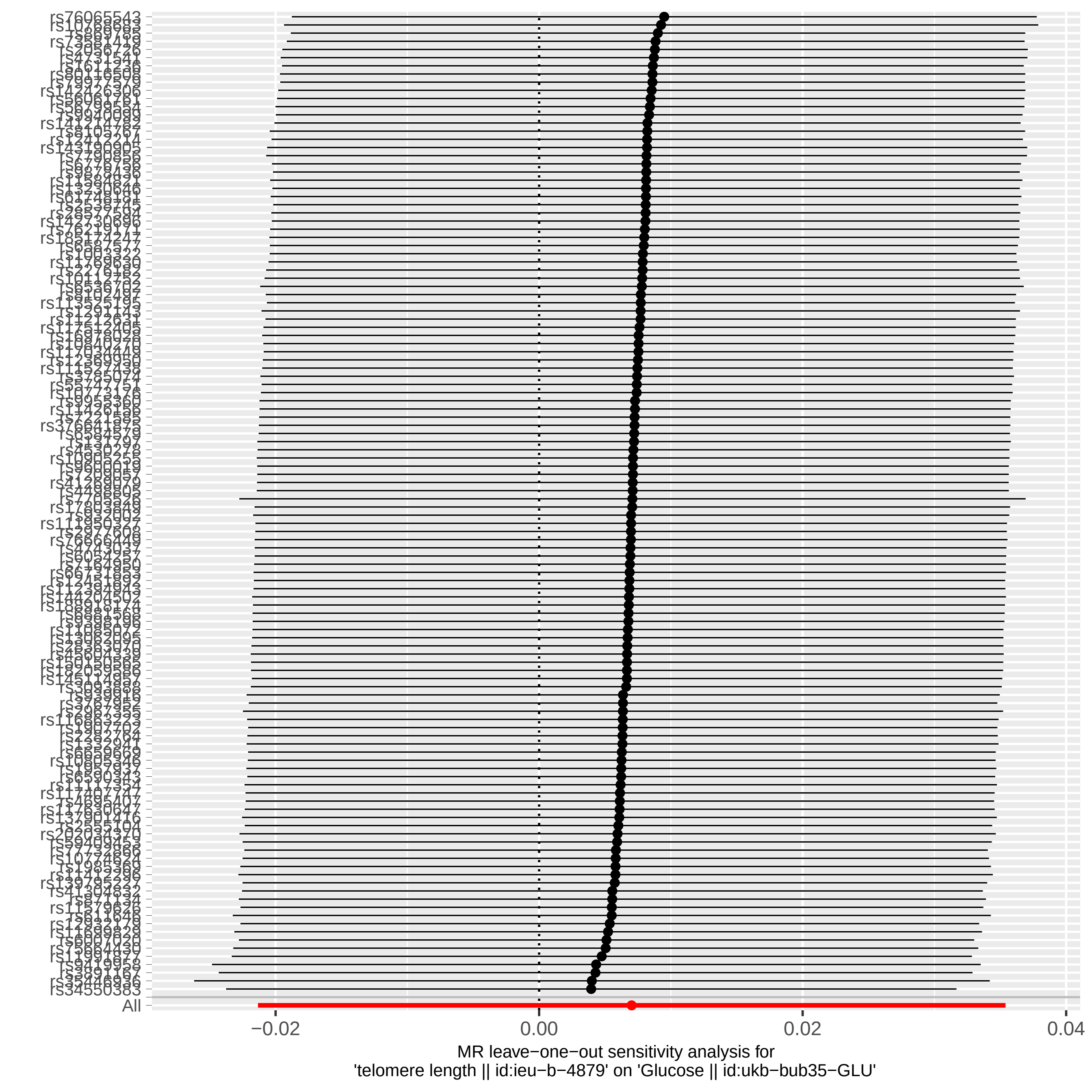
rs76065543
rs34550383
All
−0.02
0.00
0.02
0.04
MR leave−one−out sensitivity analysis for
'telomere length || id:ieu−b−4879' on 'Glucose || id:ukb−bub35−GLU'

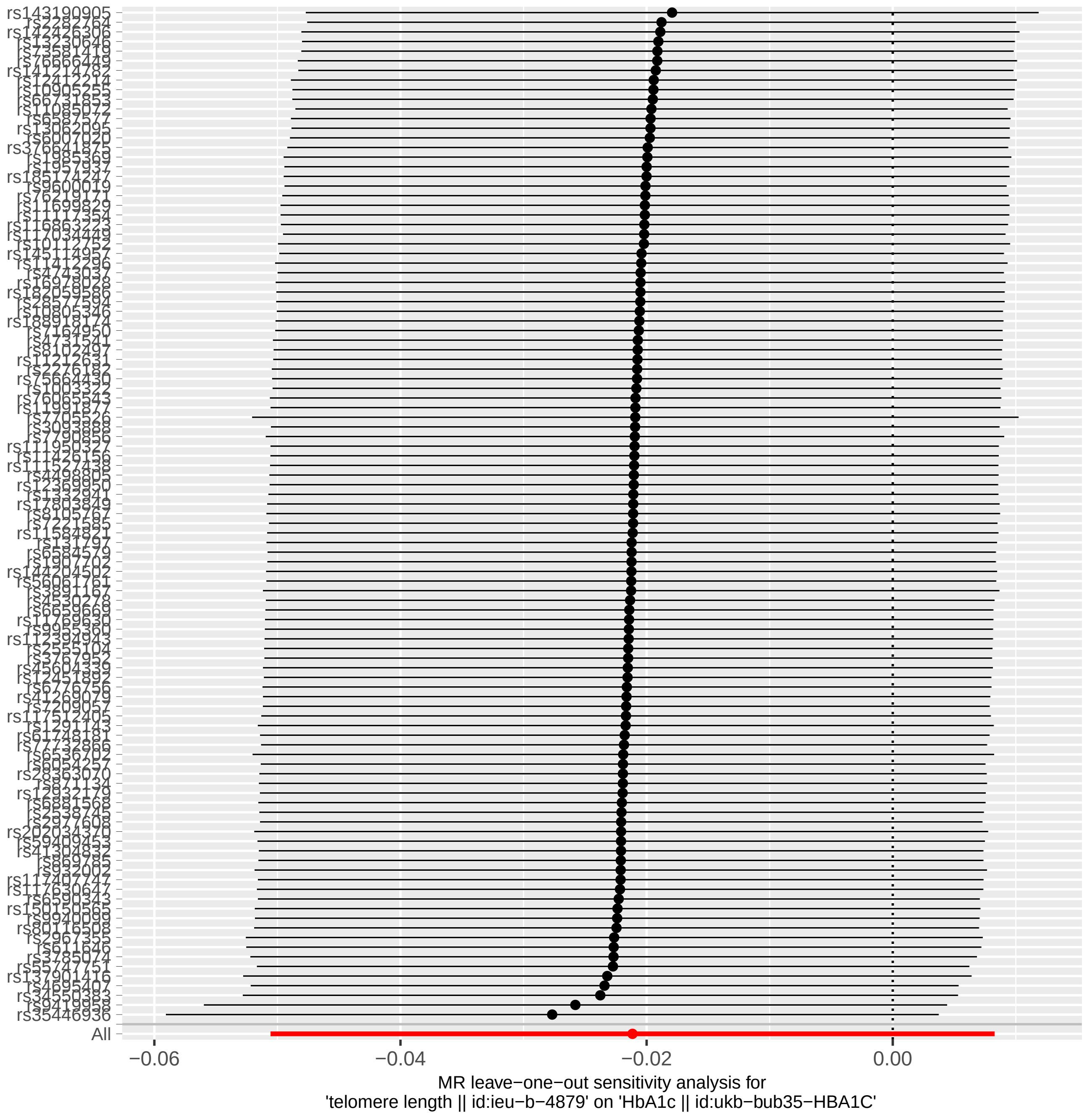
rs143190905
rs35446936
All
−0.06
−0.04
−0.02
0.00
MR leave−one−out sensitivity analysis for
'telomere length || id:ieu−b−4879' on 'HbA1c || id:ukb−bub35−HBA1C'

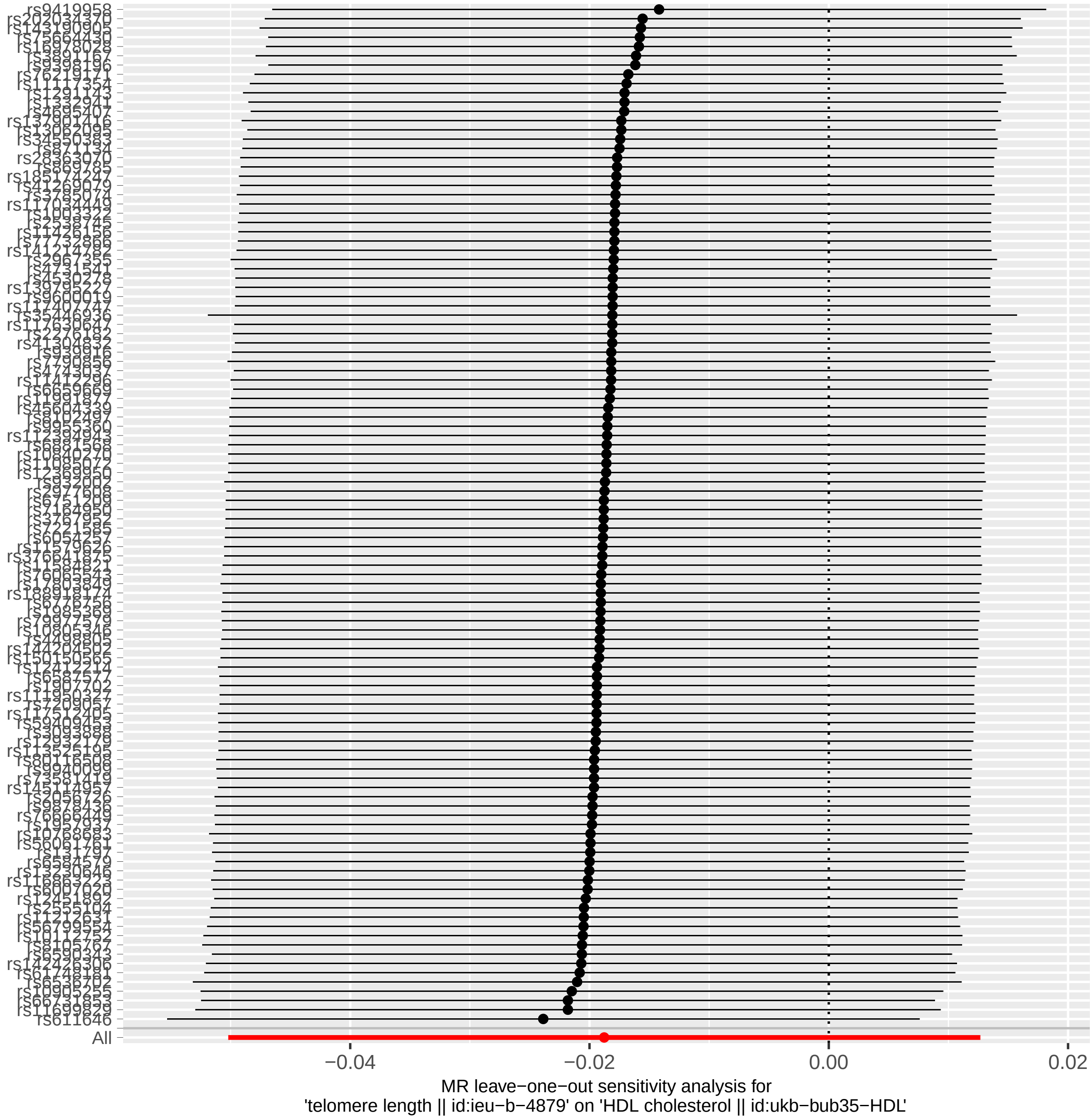

rs9419958
rs611646
All
−0.04
−0.02
0.00
0.02
MR leave−one−out sensitivity analysis for
'telomere length || id:ieu−b−4879' on 'HDL cholesterol || id:ukb−bub35−HDL'

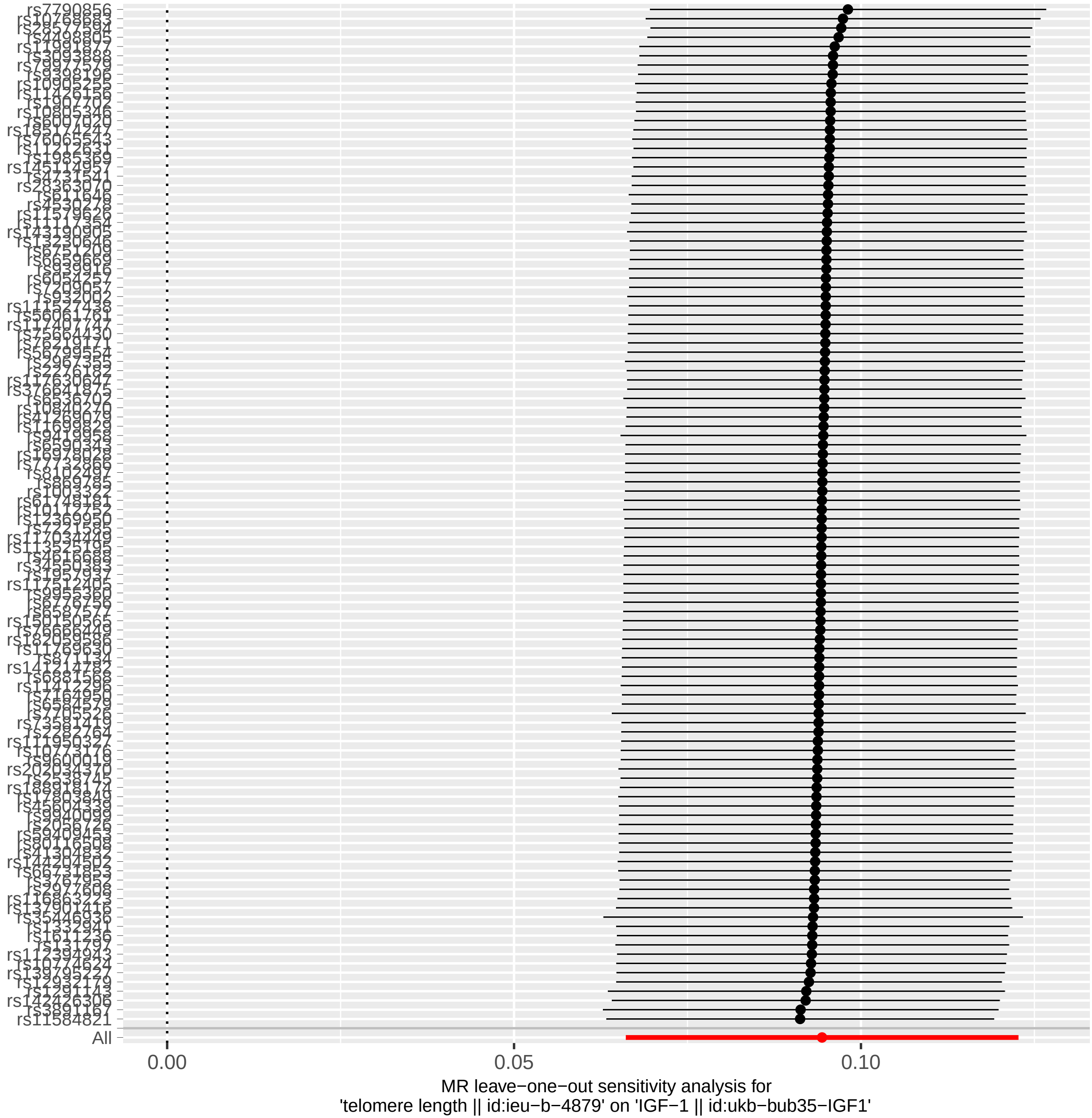
All
0.00
0.05
0.10
MR leave−one−out sensitivity analysis for
'telomere length || id:ieu−b−4879' on 'IGF−1 || id:ukb−bub35−IGF1'

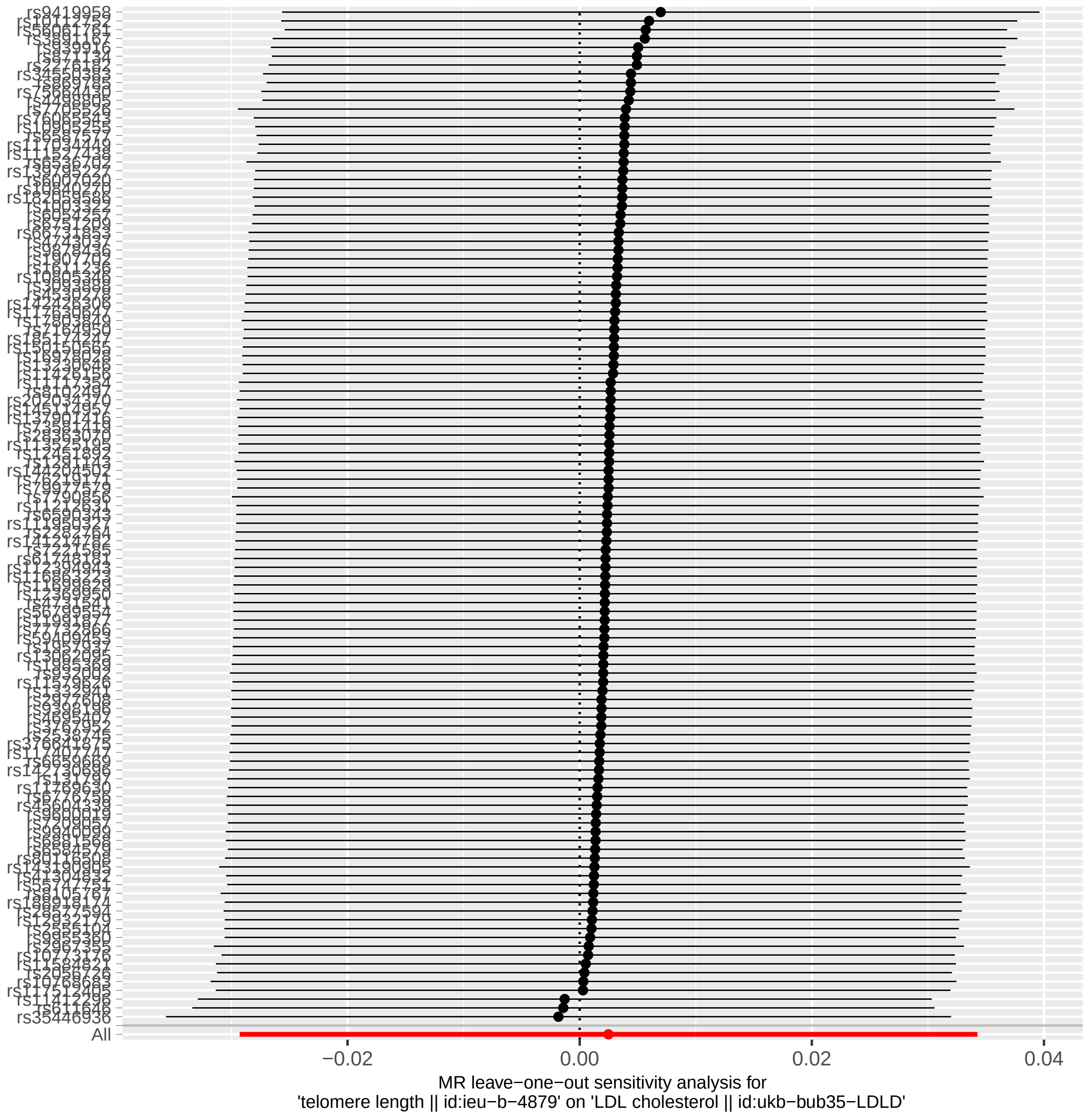
rs9419958
rs35446936
All
−0.02
0.00
0.02
0.04
MR leave−one−out sensitivity analysis for
'telomere length || id:ieu−b−4879' on 'LDL cholesterol || id:ukb−bub35−LDLD'

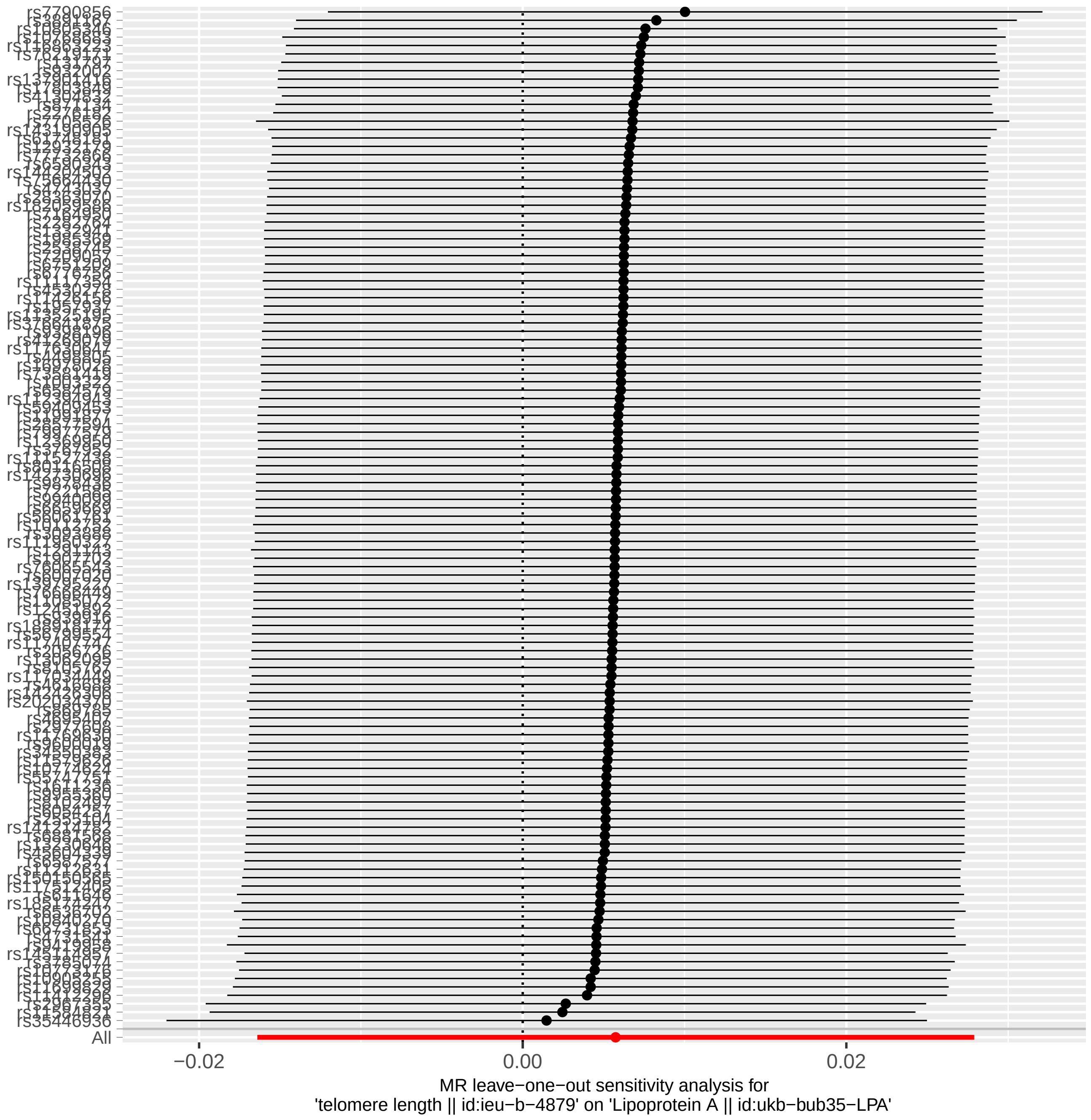
rs7790856
rs35446936
All
−0.02
0.00
0.02
MR leave−one−out sensitivity analysis for
'telomere length || id:ieu−b−4879' on 'Lipoprotein A || id:ukb−bub35−LPA'

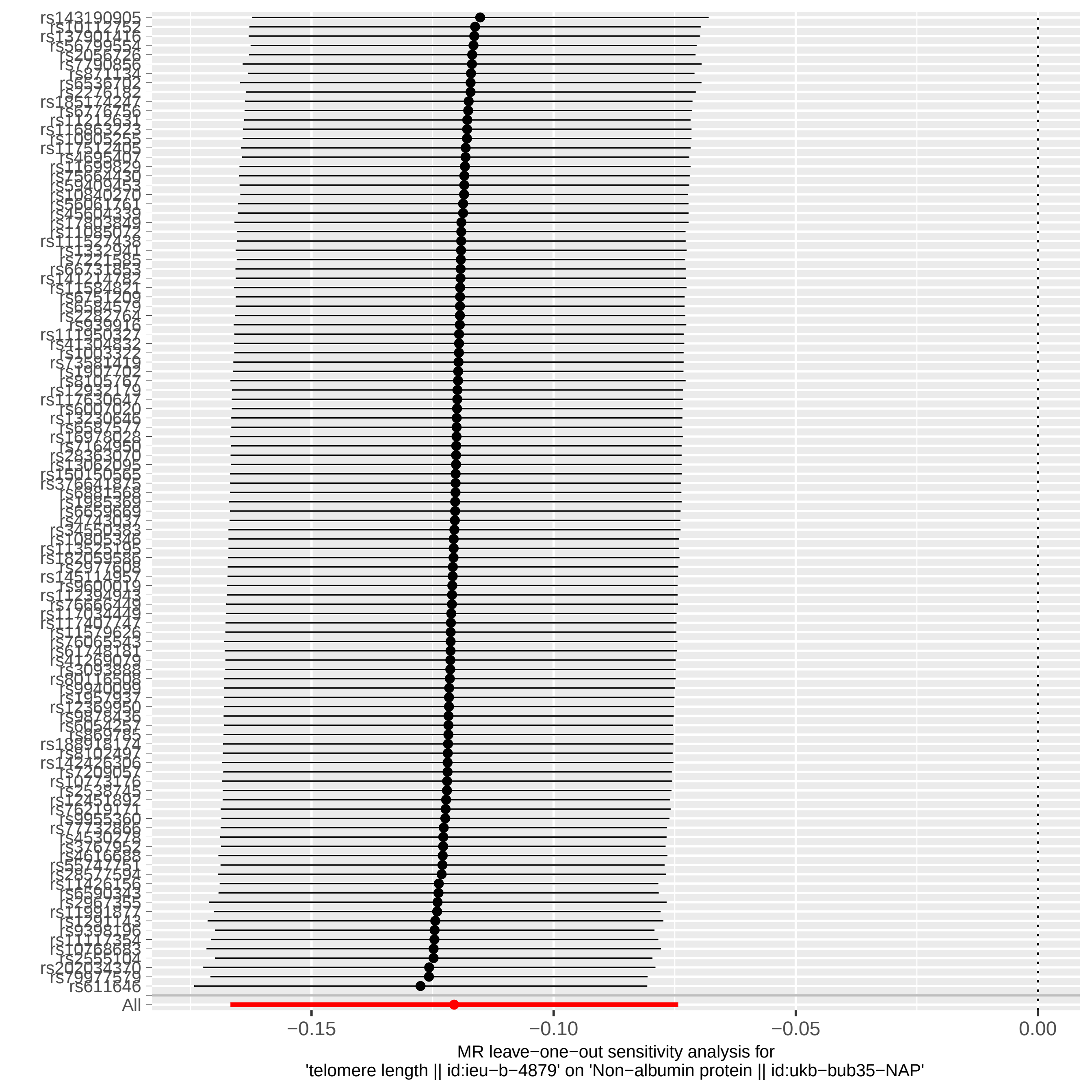
rs143190905
rs611646
All
−0.15
−0.10
−0.05
0.00
MR leave−one−out sensitivity analysis for
'telomere length || id:ieu−b−4879' on 'Non−albumin protein || id:ukb−bub35−NAP'

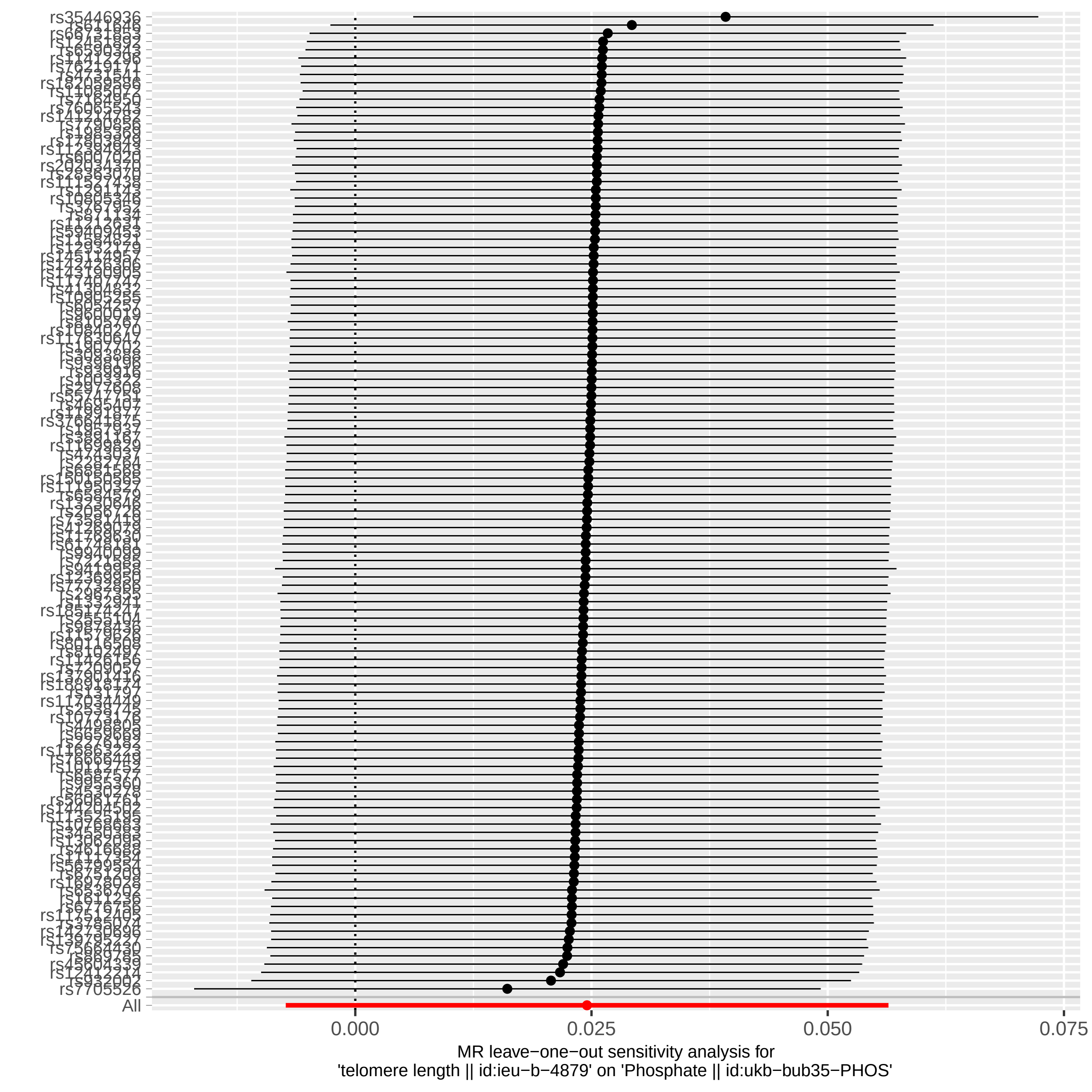
rs35446936
rs7705526
All
0.000
0.025
0.050
0.075
MR leave−one−out sensitivity analysis for
'telomere length || id:ieu−b−4879' on 'Phosphate || id:ukb−bub35−PHOS'

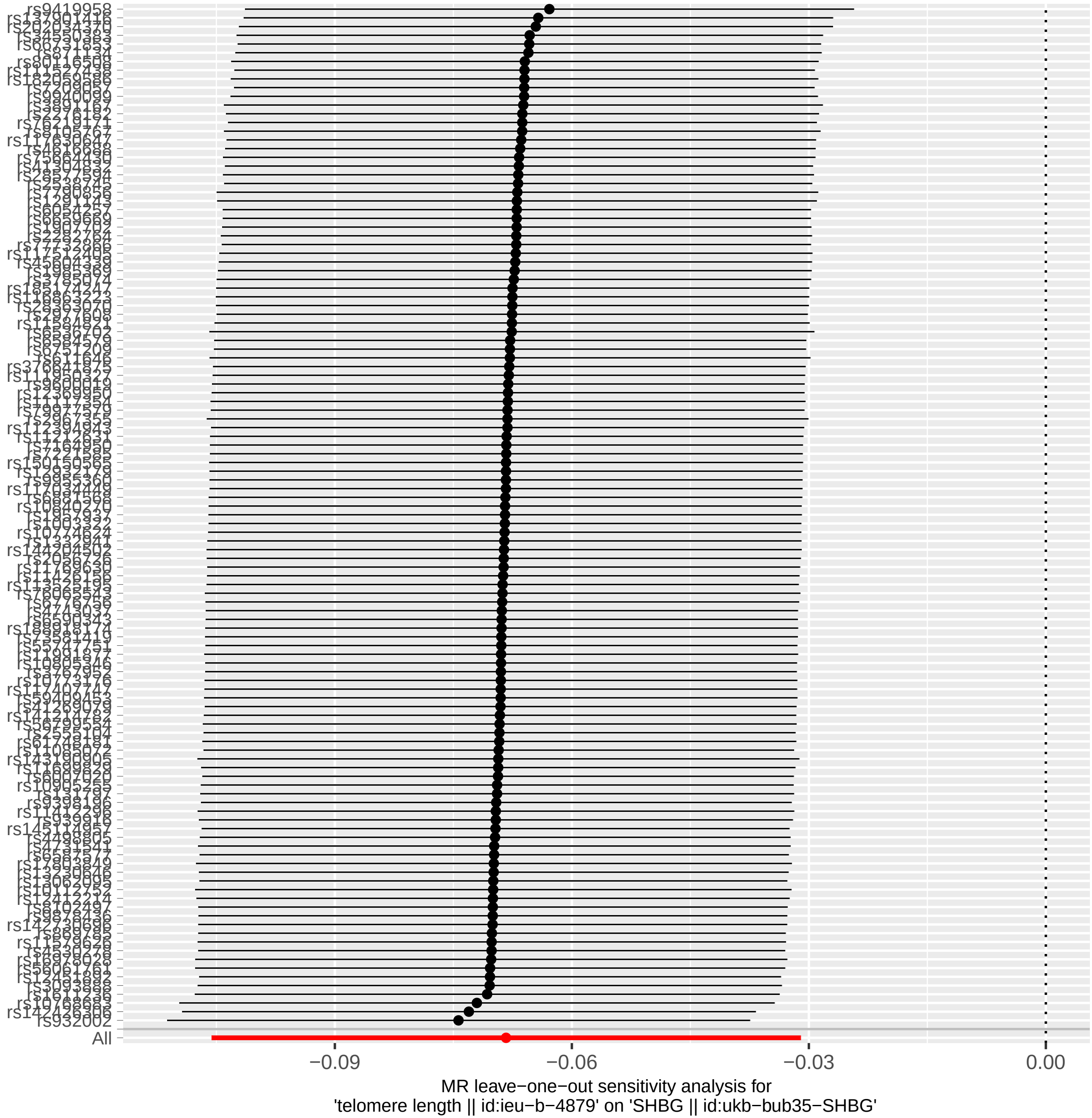
rs9419958
rs932002
All
−0.09
−0.06
−0.03
0.00
MR leave−one−out sensitivity analysis for
'telomere length || id:ieu−b−4879' on 'SHBG || id:ukb−bub35−SHBG'

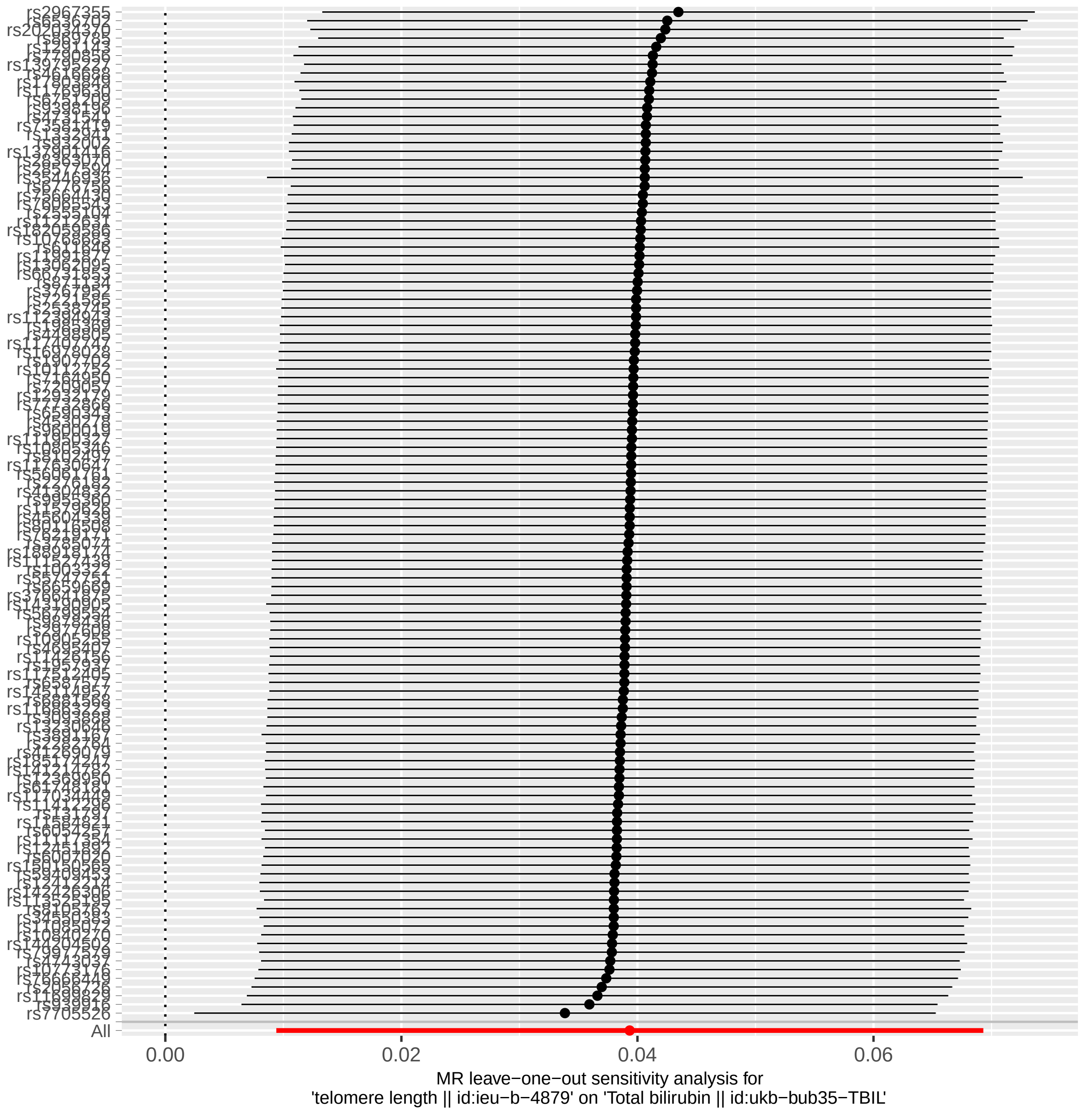
rs2967355
rs7705526
All
0.00
0.02
0.04
0.06
MR leave−one−out sensitivity analysis for
'telomere length || id:ieu−b−4879' on 'Total bilirubin || id:ukb−bub35−TBIL'

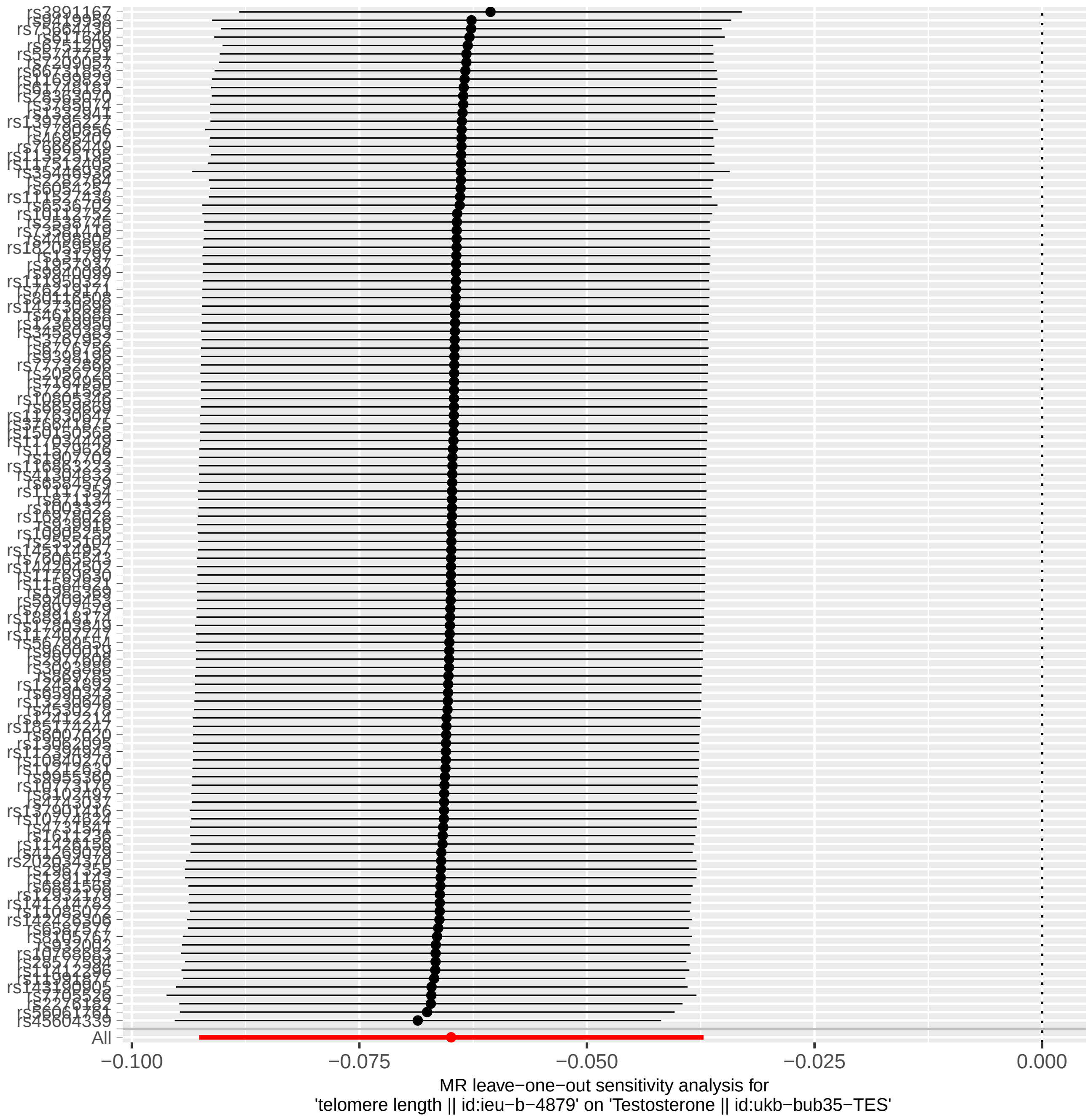
rs3891167
rs45604339
All
−0.100
−0.075
−0.050
−0.025
0.000
MR leave−one−out sensitivity analysis for
'telomere length || id:ieu−b−4879' on 'Testosterone || id:ukb−bub35−TES'

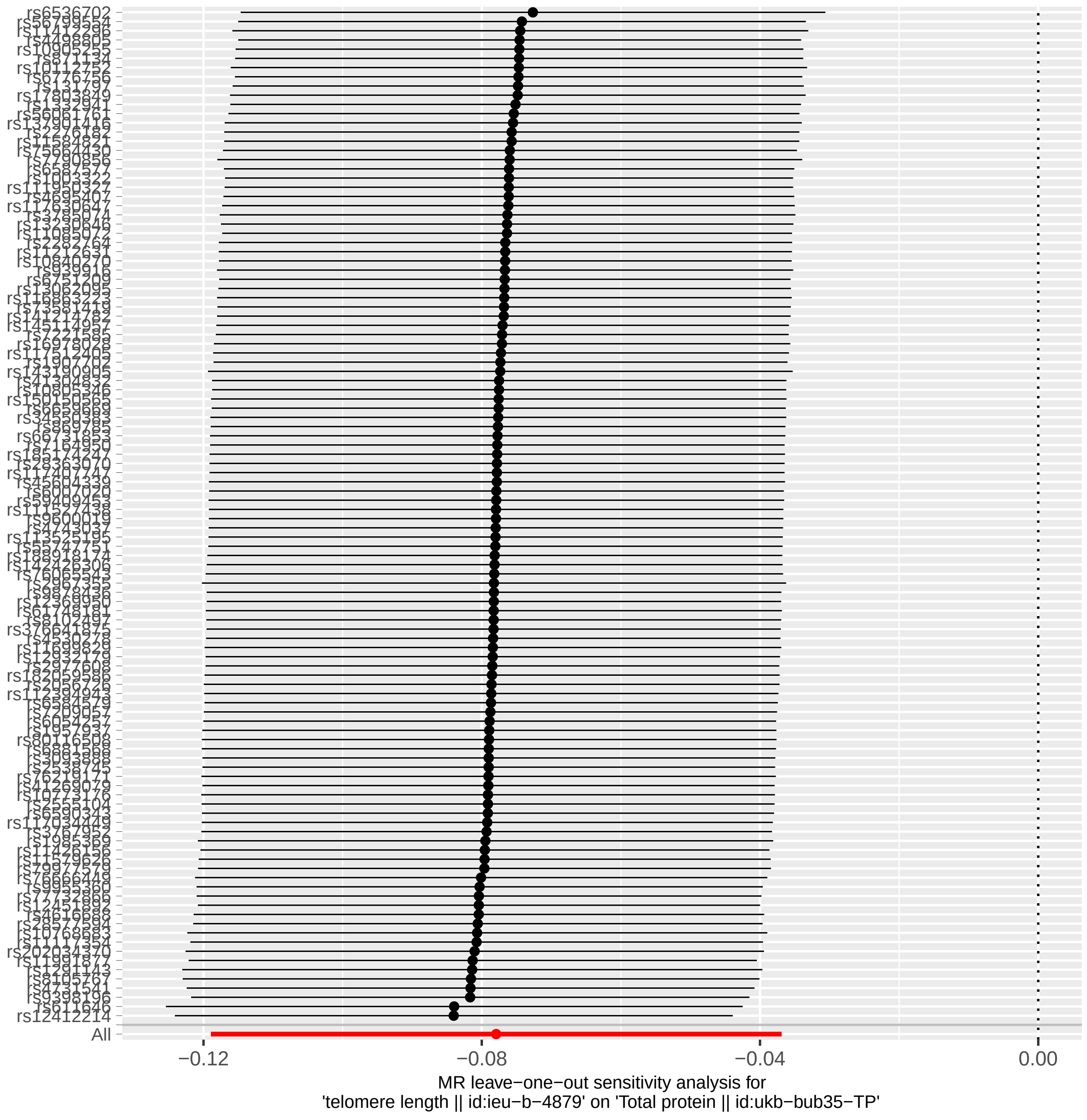

rs6536702
rs611646
rs12412214
All
−0.12
−0.08
−0.04
0.00
MR leave−one−out sensitivity analysis for
'telomere length || id:ieu−b−4879' on 'Total protein || id:ukb−bub35−TP'

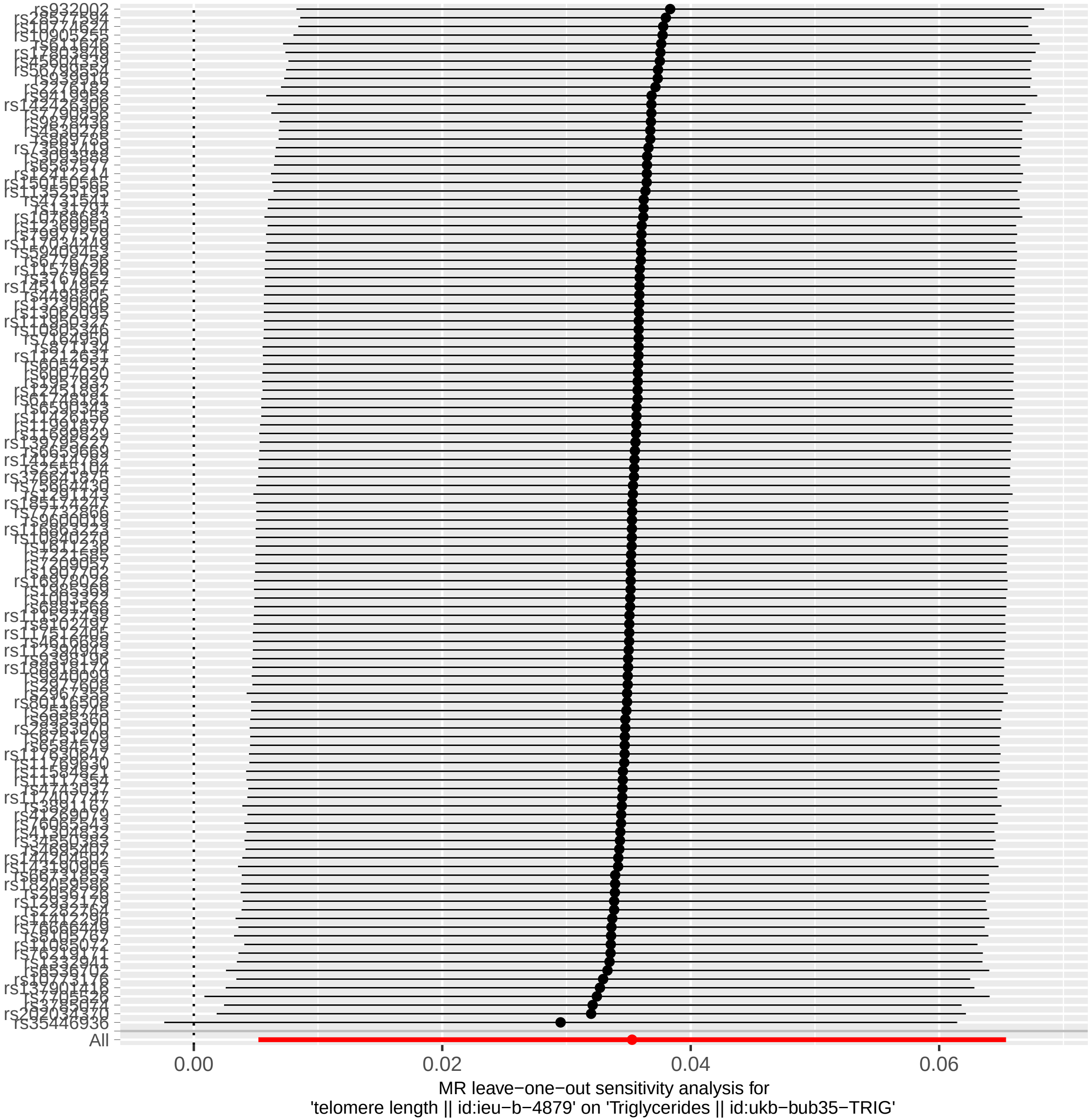
rs932002
rs35446936
All
0.00
0.02
0.04
0.06
MR leave−one−out sensitivity analysis for
'telomere length || id:ieu−b−4879' on 'Triglycerides || id:ukb−bub35−TRIG'

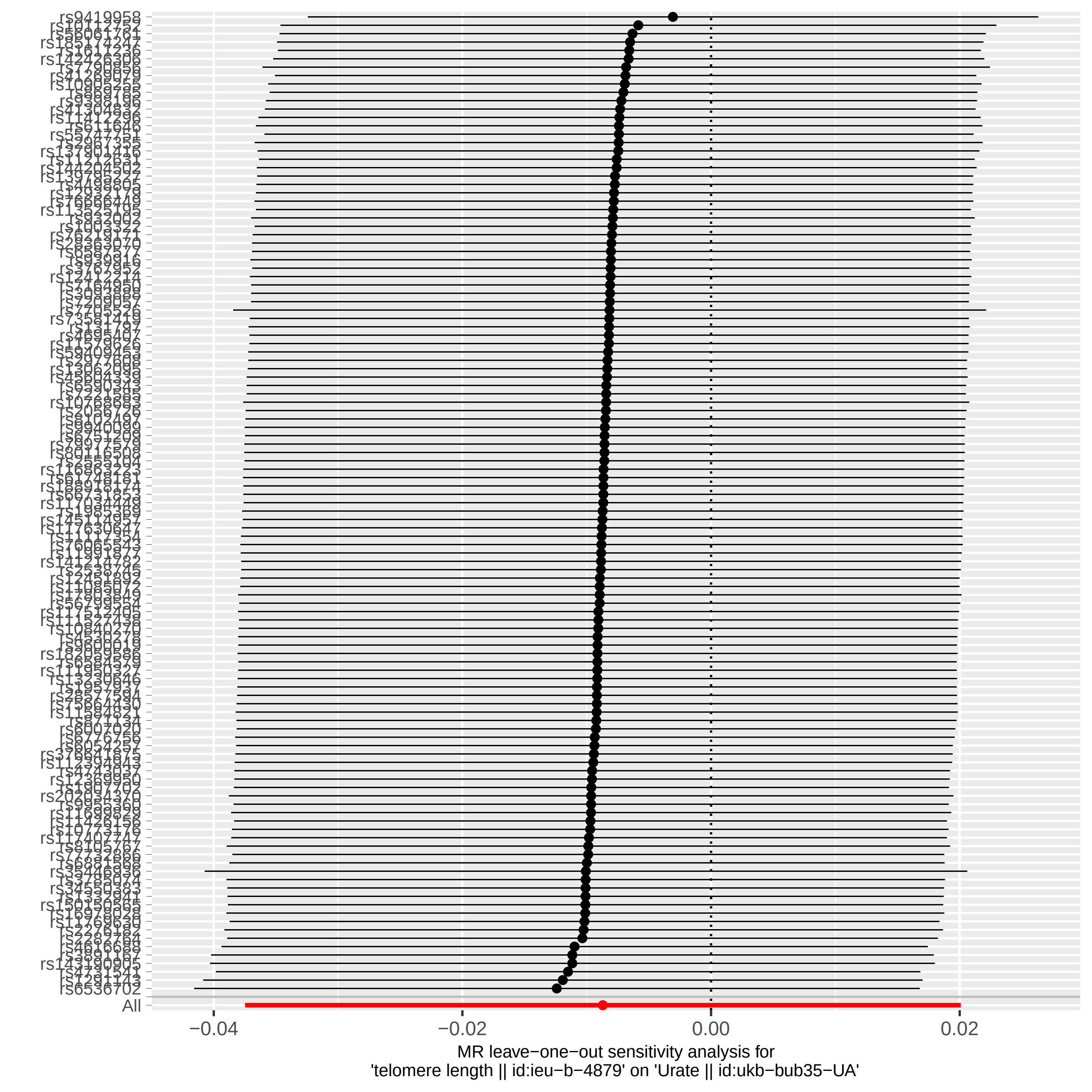
rs9419958
rs6536702
All
−0.04
−0.02
0.00
0.02
MR leave−one−out sensitivity analysis for
'telomere length || id:ieu−b−4879' on 'Urate || id:ukb−bub35−UA'

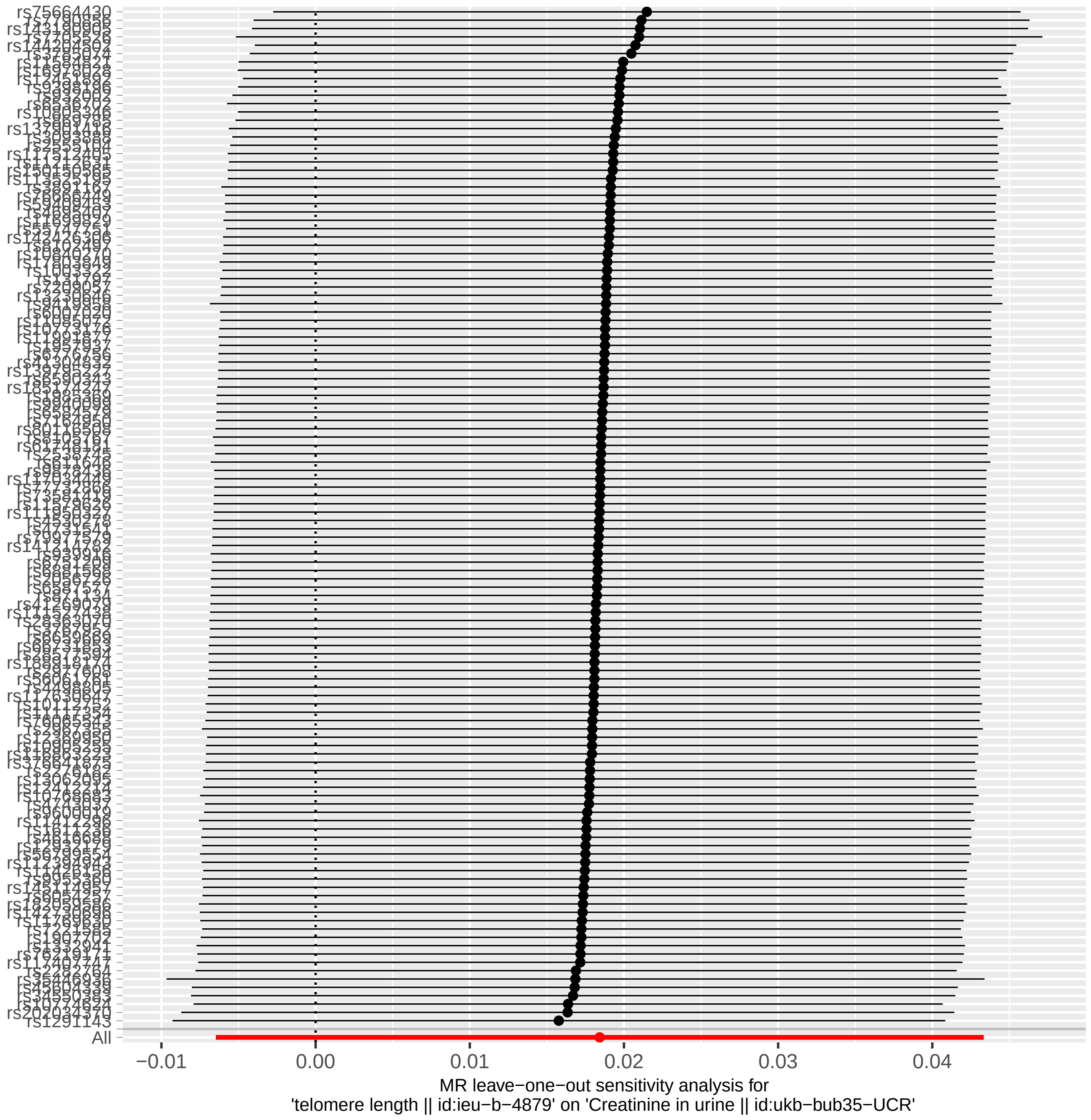
rs75664430
rs1291143
All
−0.01
0.00
0.01
0.02
0.03
0.04
MR leave−one−out sensitivity analysis for
'telomere length || id:ieu−b−4879' on 'Creatinine in urine || id:ukb−bub35−UCR'

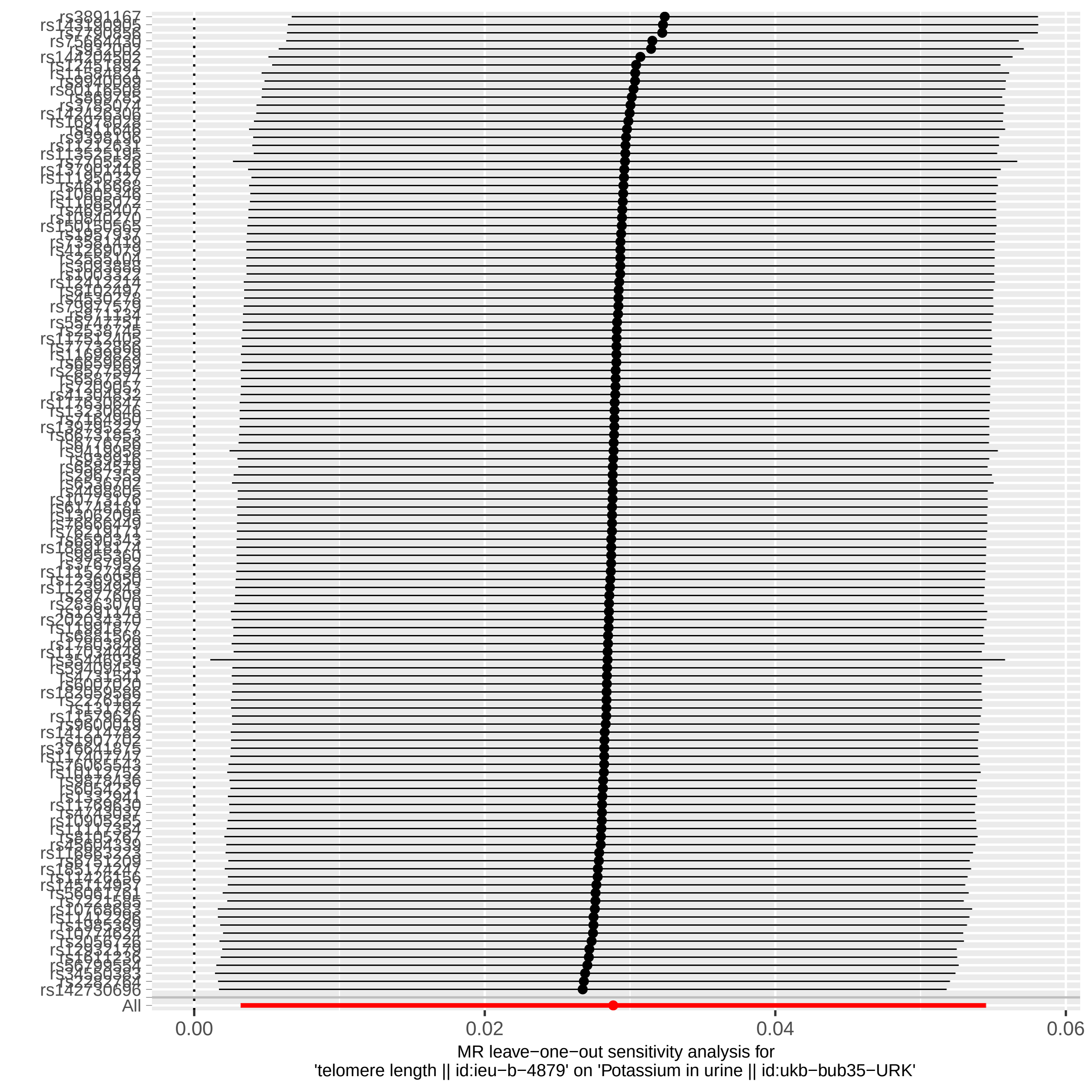
rs3891167
rs142730696
All
0.00
0.02
0.04
0.06
MR leave−one−out sensitivity analysis for
'telomere length || id:ieu−b−4879' on 'Potassium in urine || id:ukb−bub35−URK'

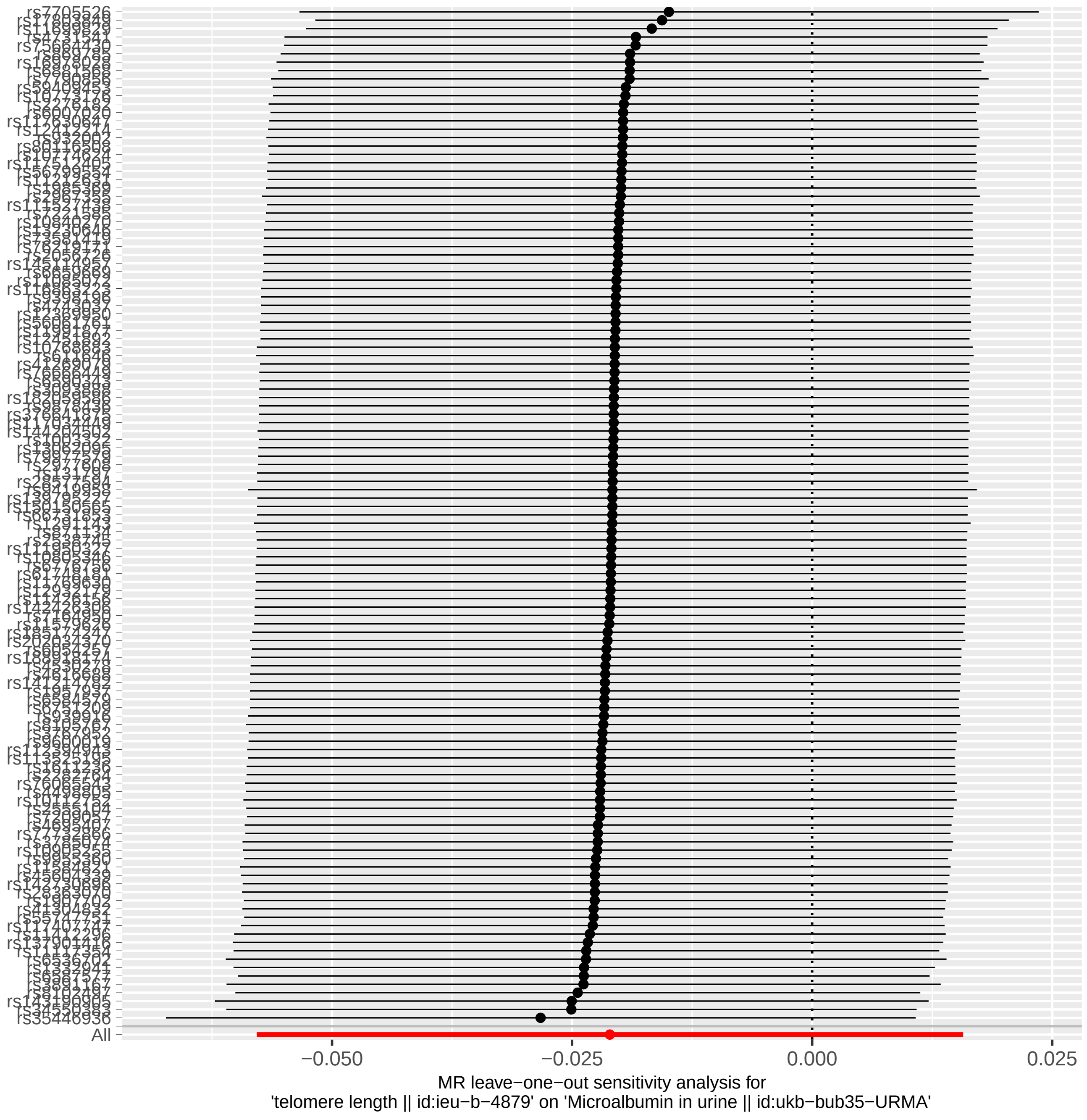
rs7705526
rs35446936
All
−0.050
−0.025
0.000
0.025
MR leave−one−out sensitivity analysis for
'telomere length || id:ieu−b−4879' on 'Microalbumin in urine || id:ukb−bub35−URMA'

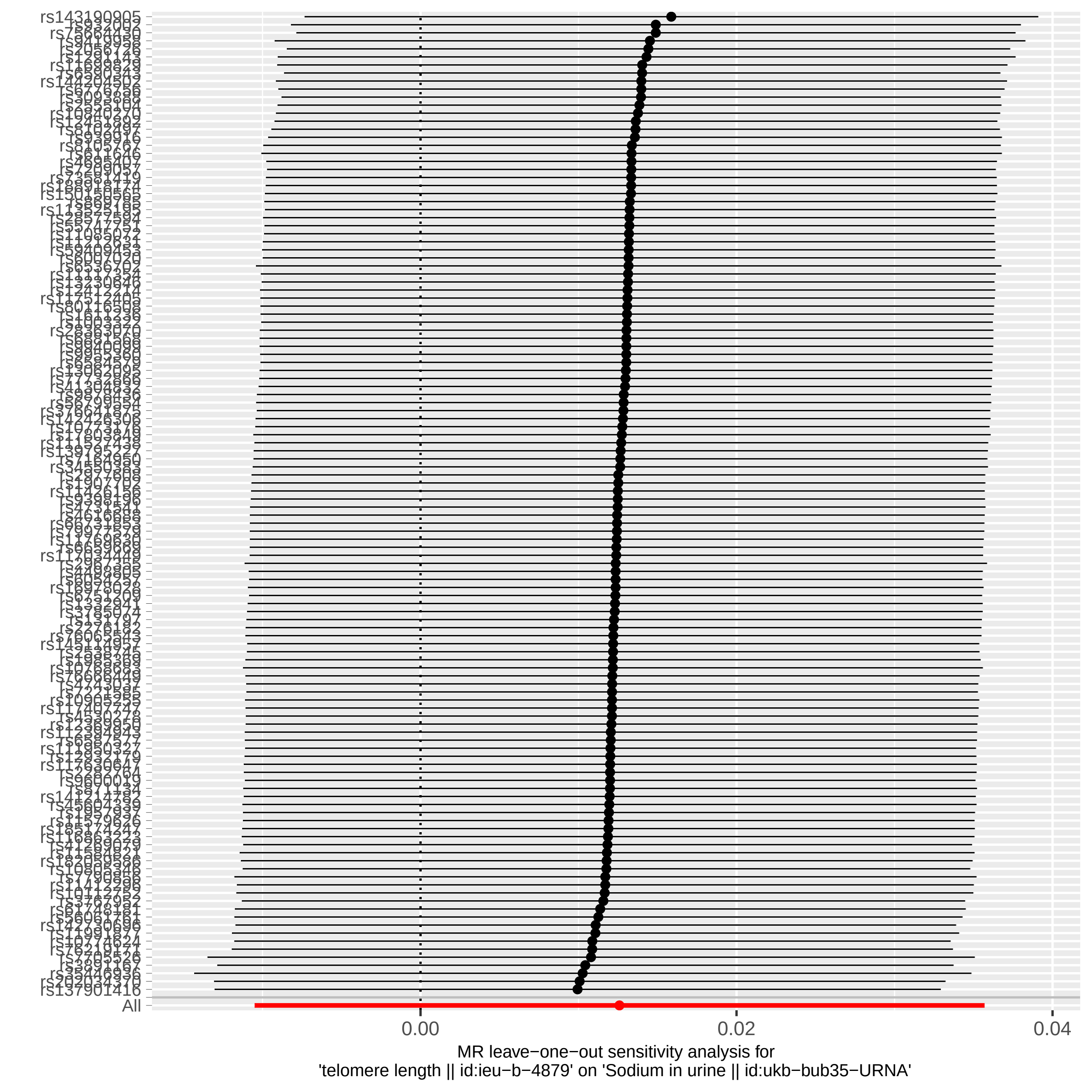
rs143190905
rs137901416
All
0.00
0.02
0.04
MR leave−one−out sensitivity analysis for
'telomere length || id:ieu−b−4879' on 'Sodium in urine || id:ukb−bub35−URNA'

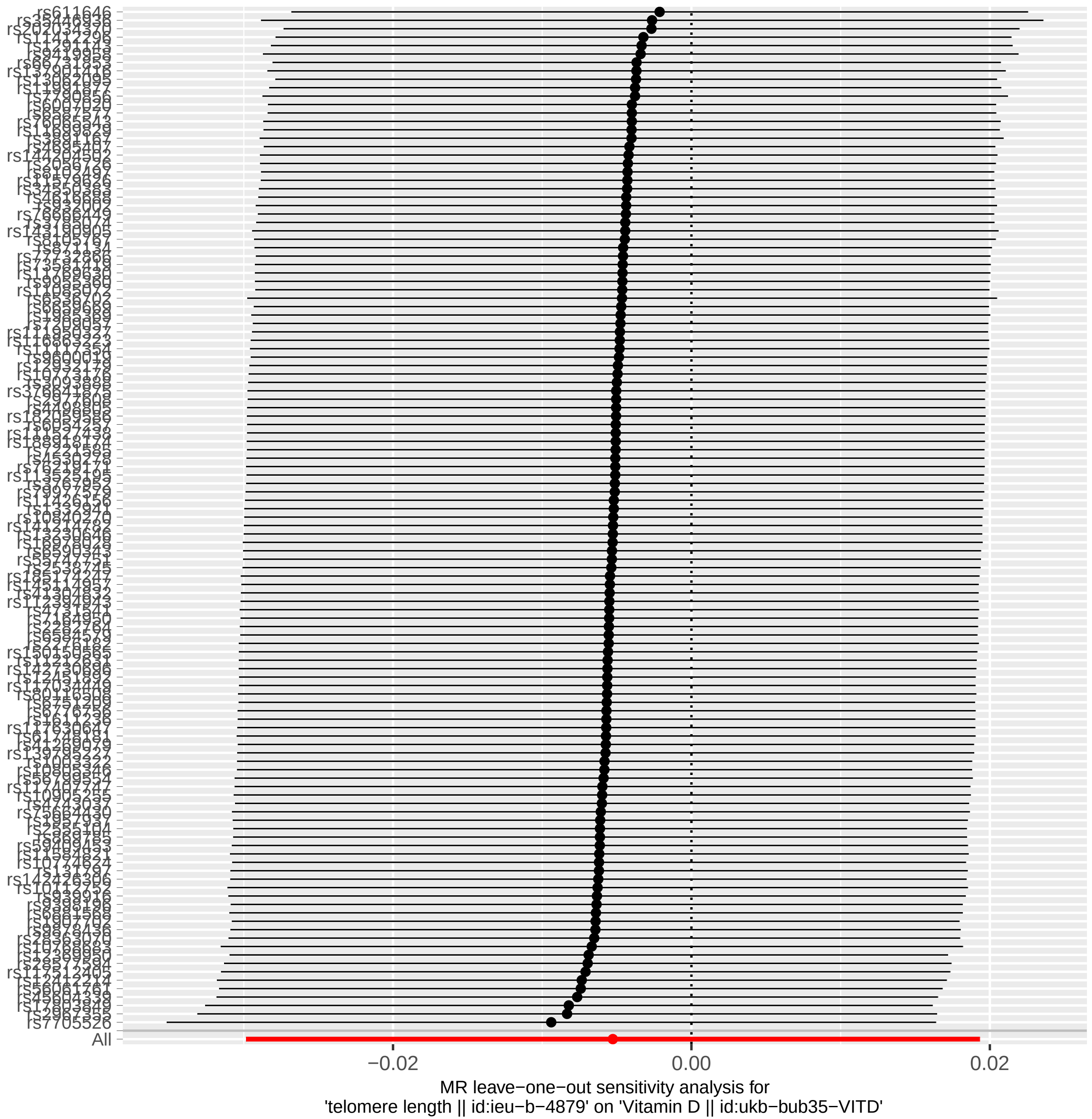
rs611646
rs7705526
All
−0.02
0.00
0.02
MR leave−one−out sensitivity analysis for
'telomere length || id:ieu−b−4879' on 'Vitamin D || id:ukb−bub35−VITD'

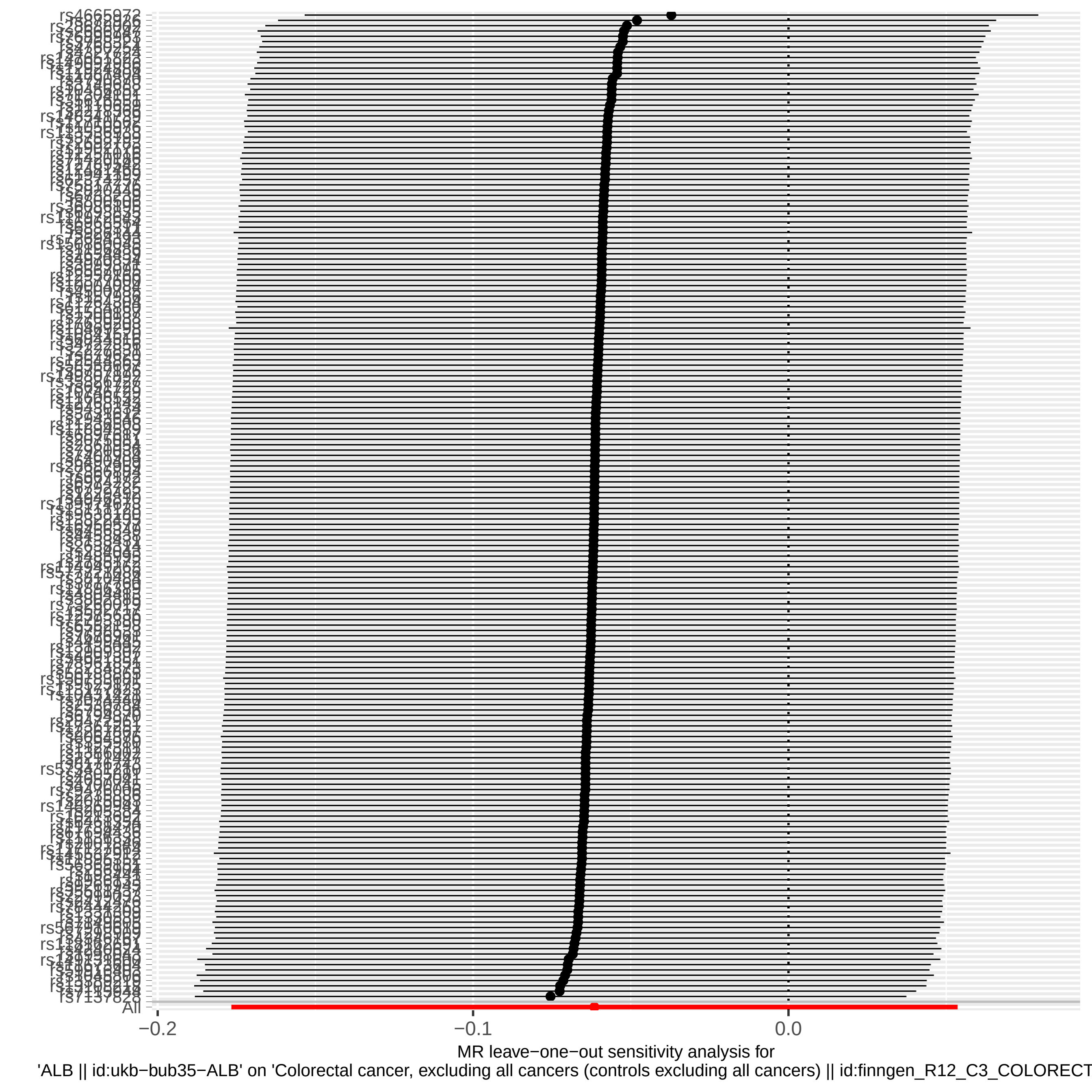
rs4665972
rs7137828
All
−0.2
−0.1
0.0
MR leave−one−out sensitivity analysis for
'ALB || id:ukb−bub35−ALB' on 'Colorectal cancer, excluding all cancers (controls excluding all cancers) || id:finngen_R12_C3_COLORECT

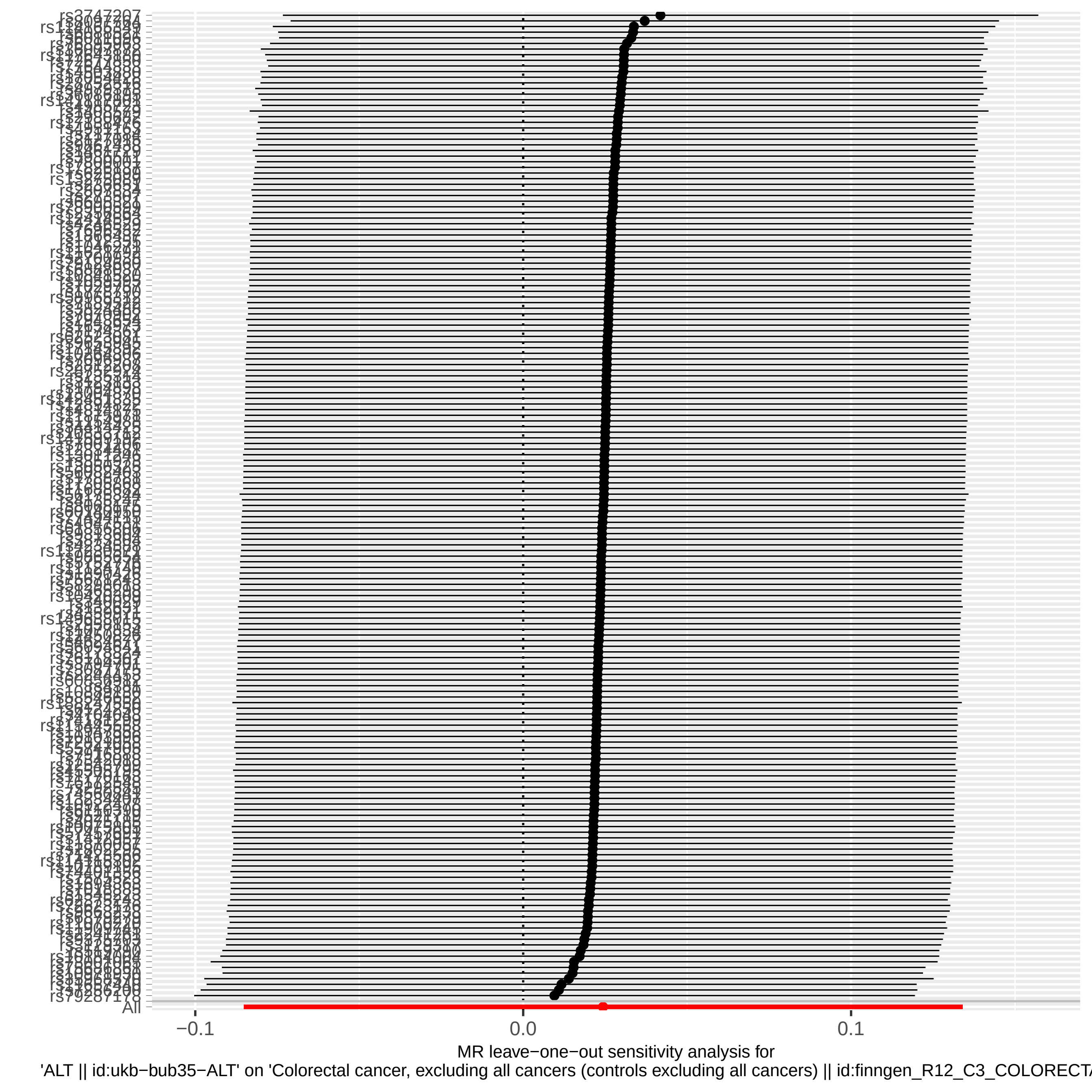

rs3747207
rs79287176
All
−0.1
0.0
0.1
MR leave−one−out sensitivity analysis for
'ALT || id:ukb−bub35−ALT' on 'Colorectal cancer, excluding all cancers (controls excluding all cancers) || id:finngen_R12_C3_COLORECTA

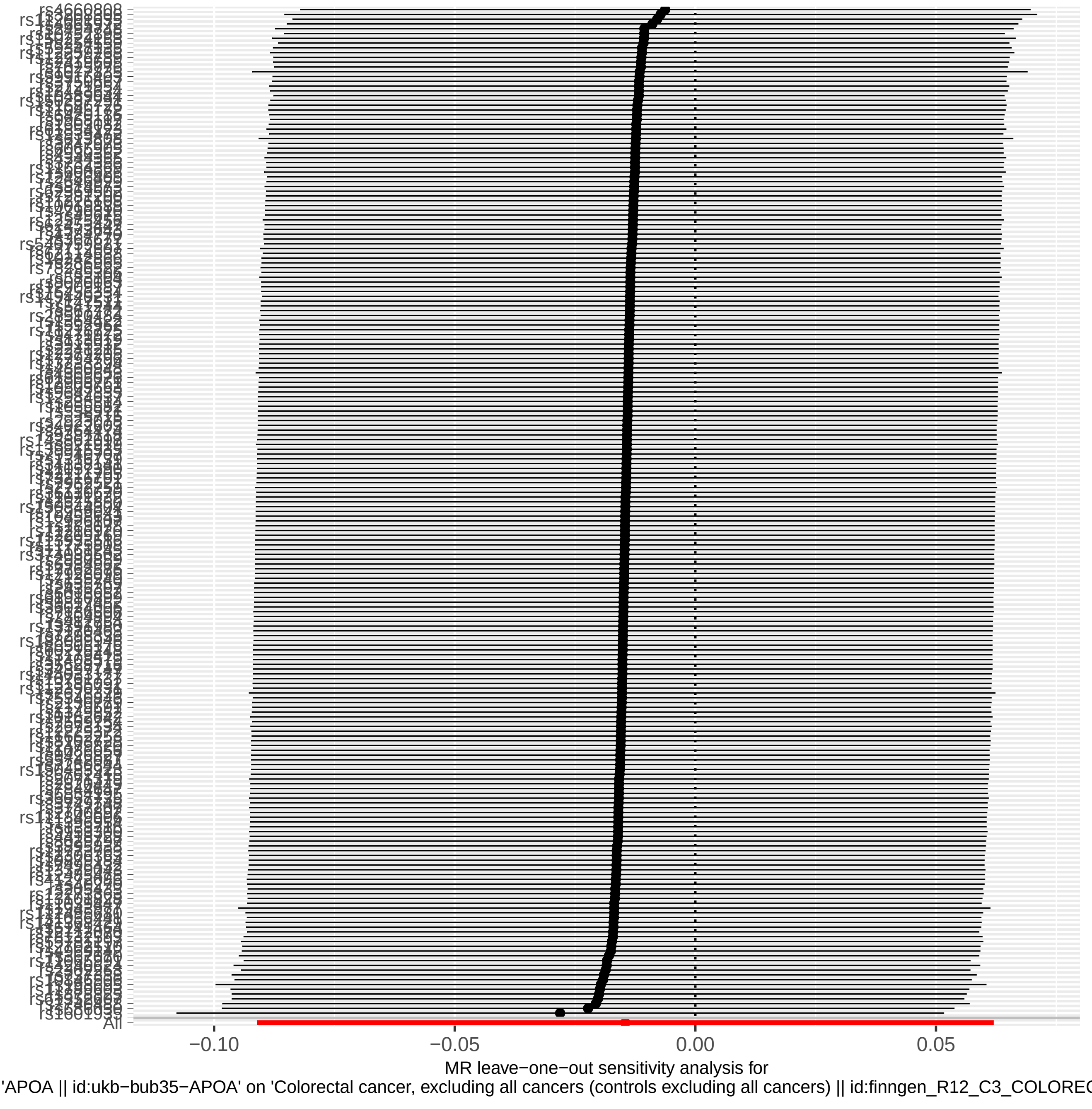

rs4660808
rs1001935
All
−0.10
−0.05
0.00
0.05
MR leave−one−out sensitivity analysis for
'APOA || id:ukb−bub35−APOA' on 'Colorectal cancer, excluding all cancers (controls excluding all cancers) || id:finngen_R12_C3_COLOREC

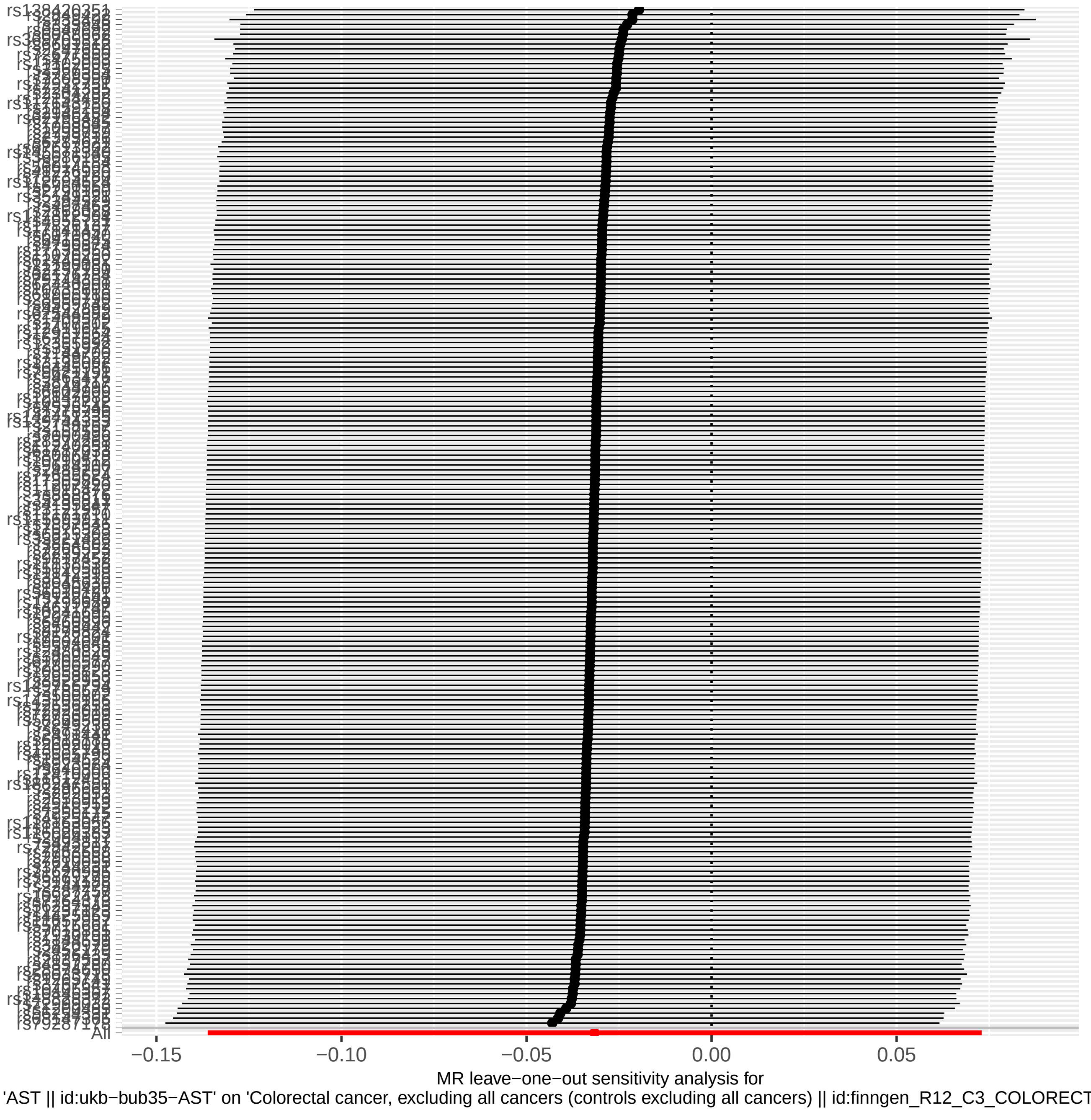

All
−0.15
−0.10
−0.05
0.00
0.05
MR leave−one−out sensitivity analysis for
'AST || id:ukb−bub35−AST' on 'Colorectal cancer, excluding all cancers (controls excluding all cancers) || id:finngen_R12_C3_COLORECT

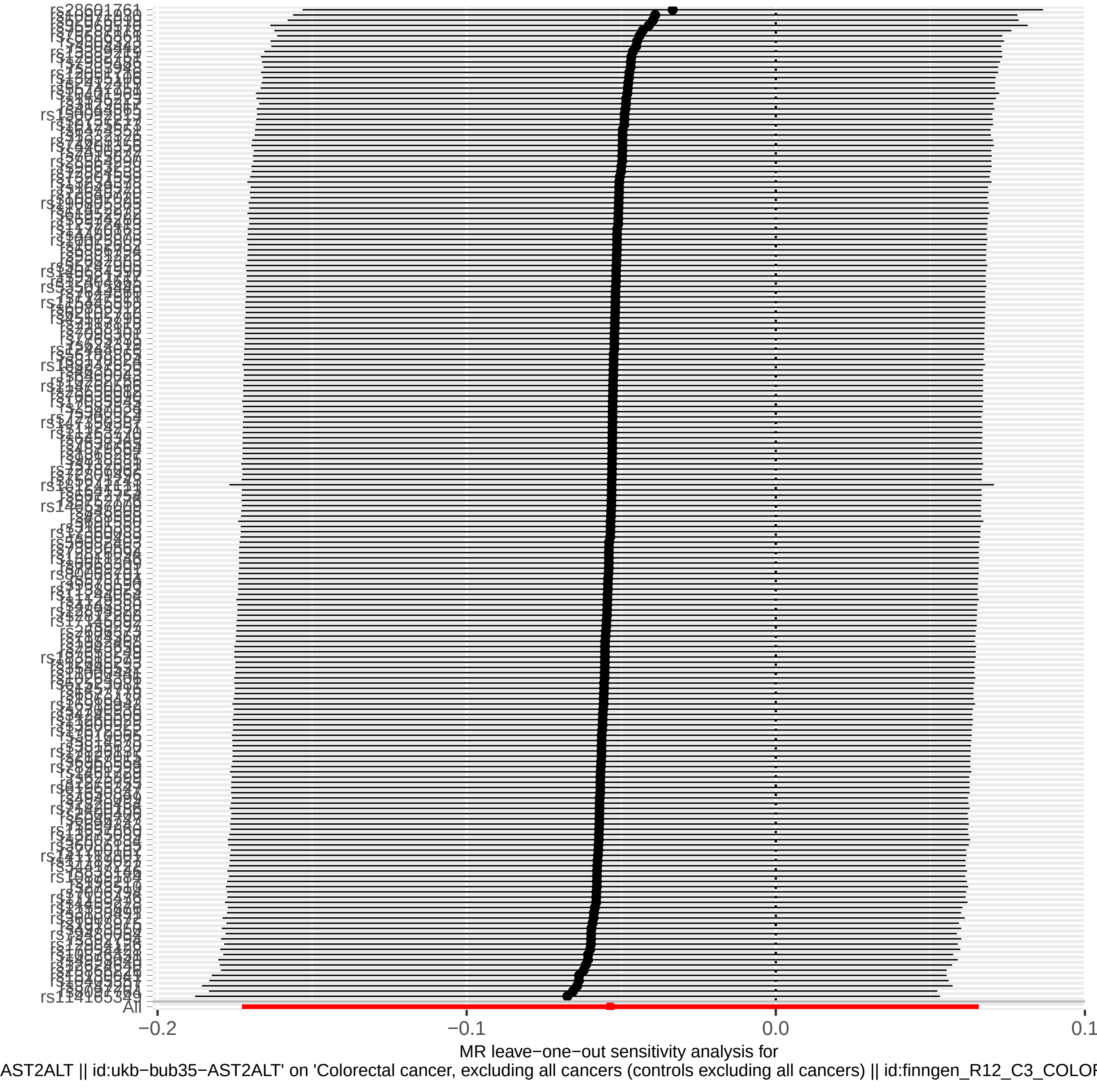

rs28601761
rs114165349
All
−0.2
−0.1
0.0
0.1
MR leave−one−out sensitivity analysis for
AST2ALT || id:ukb−bub35−AST2ALT' on 'Colorectal cancer, excluding all cancers (controls excluding all cancers) || id:finngen_R12_C3_COLOR

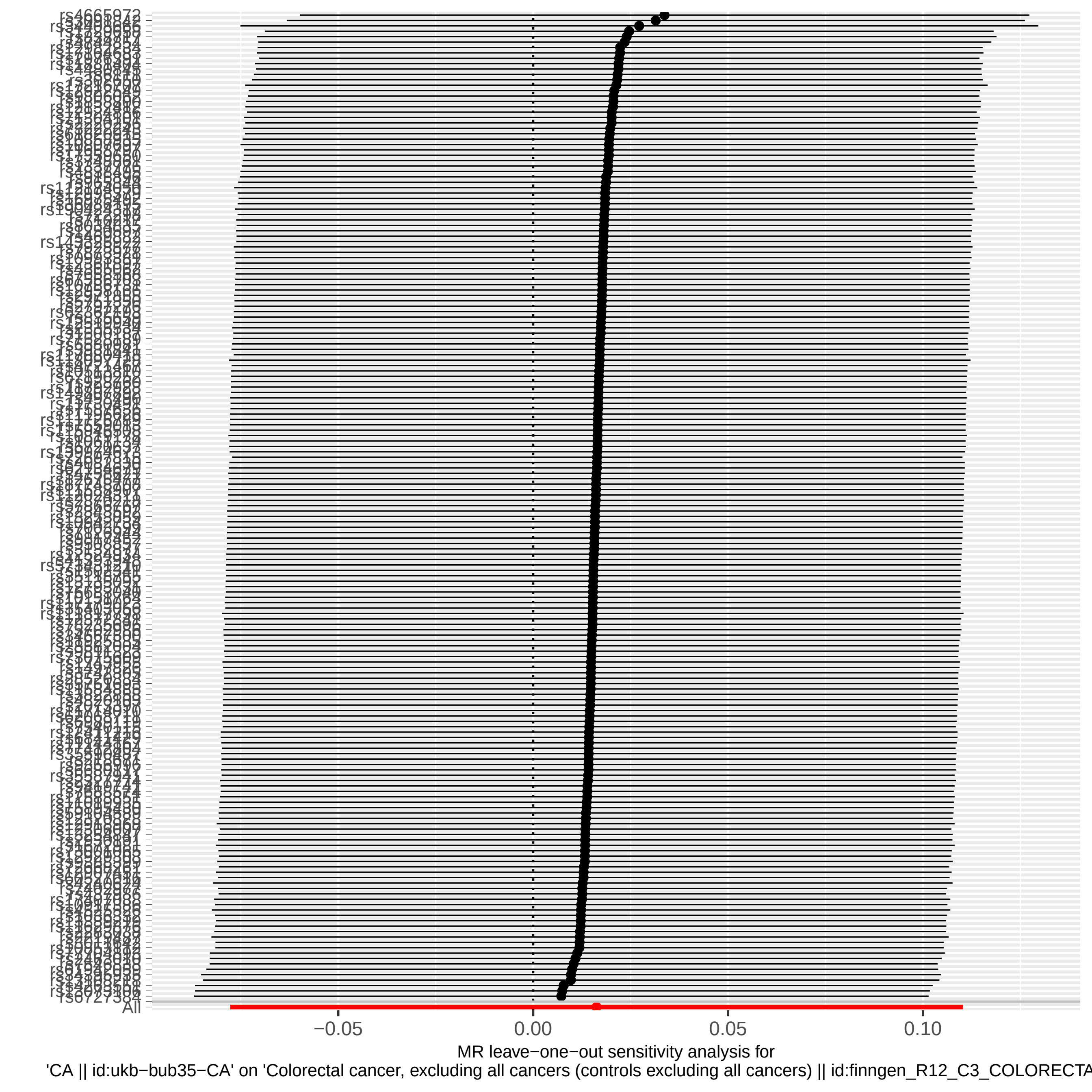

rs4665972
All
−0.05
0.00
0.05
0.10
MR leave−one−out sensitivity analysis for
'CA || id:ukb−bub35−CA' on 'Colorectal cancer, excluding all cancers (controls excluding all cancers) || id:finngen_R12_C3_COLORECTA

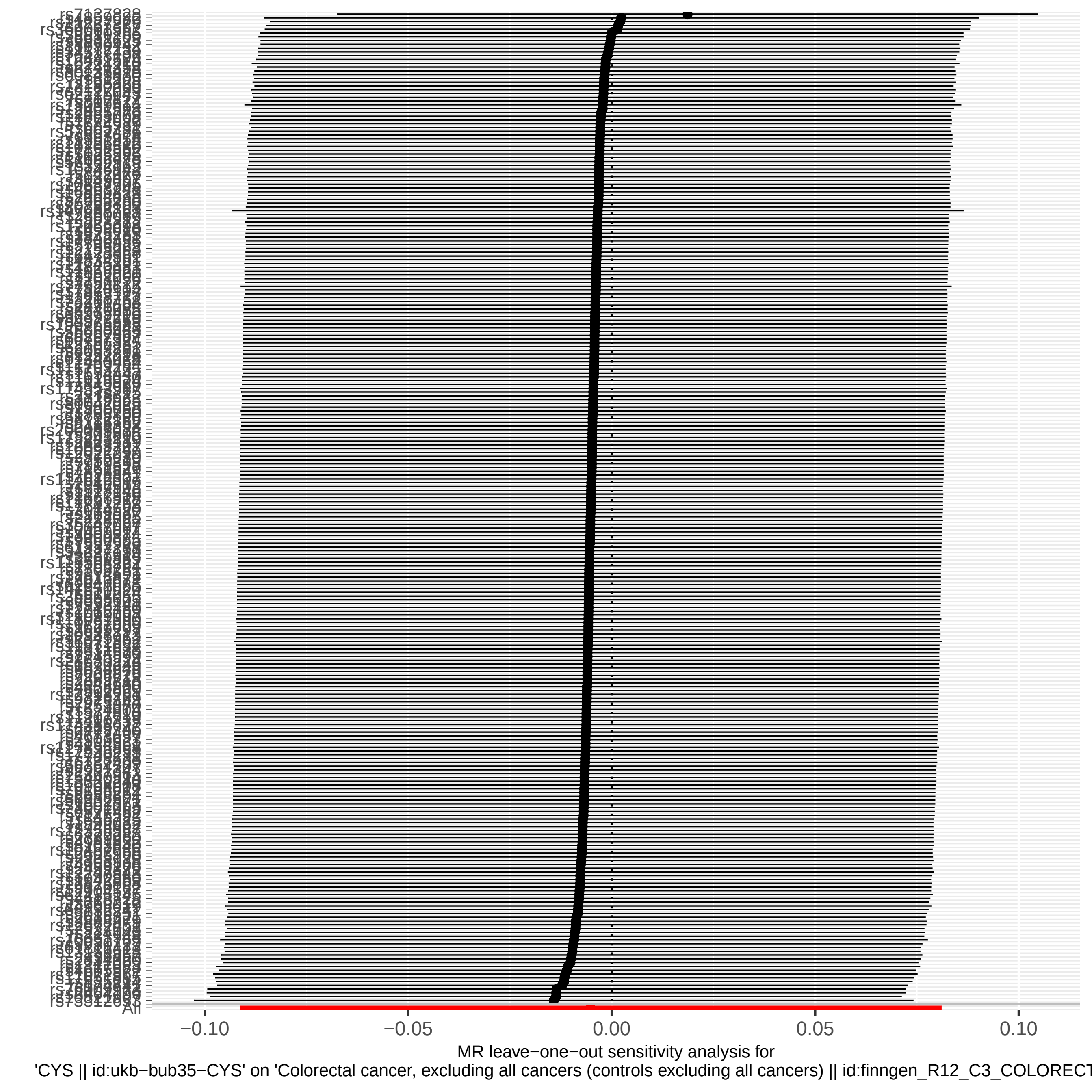

rs7137828
All
−0.10
−0.05
0.00
0.05
0.10
MR leave−one−out sensitivity analysis for
'CYS || id:ukb−bub35−CYS' on 'Colorectal cancer, excluding all cancers (controls excluding all cancers) || id:finngen_R12_C3_COLORECT

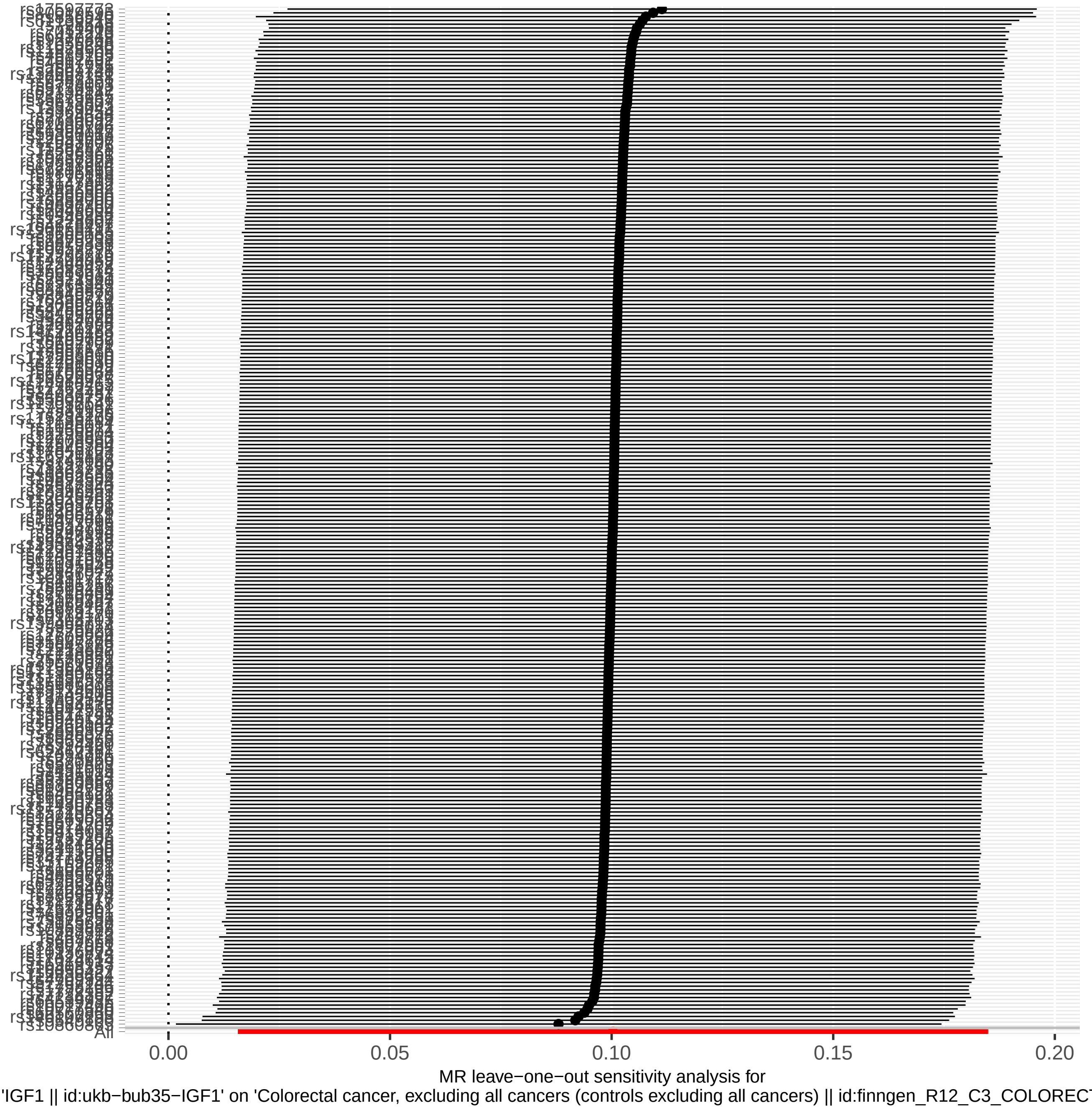

All
0.00
0.05
0.10
0.15
0.20
MR leave−one−out sensitivity analysis for
'IGF1 || id:ukb−bub35−IGF1' on 'Colorectal cancer, excluding all cancers (controls excluding all cancers) || id:finngen_R12_C3_COLOREC

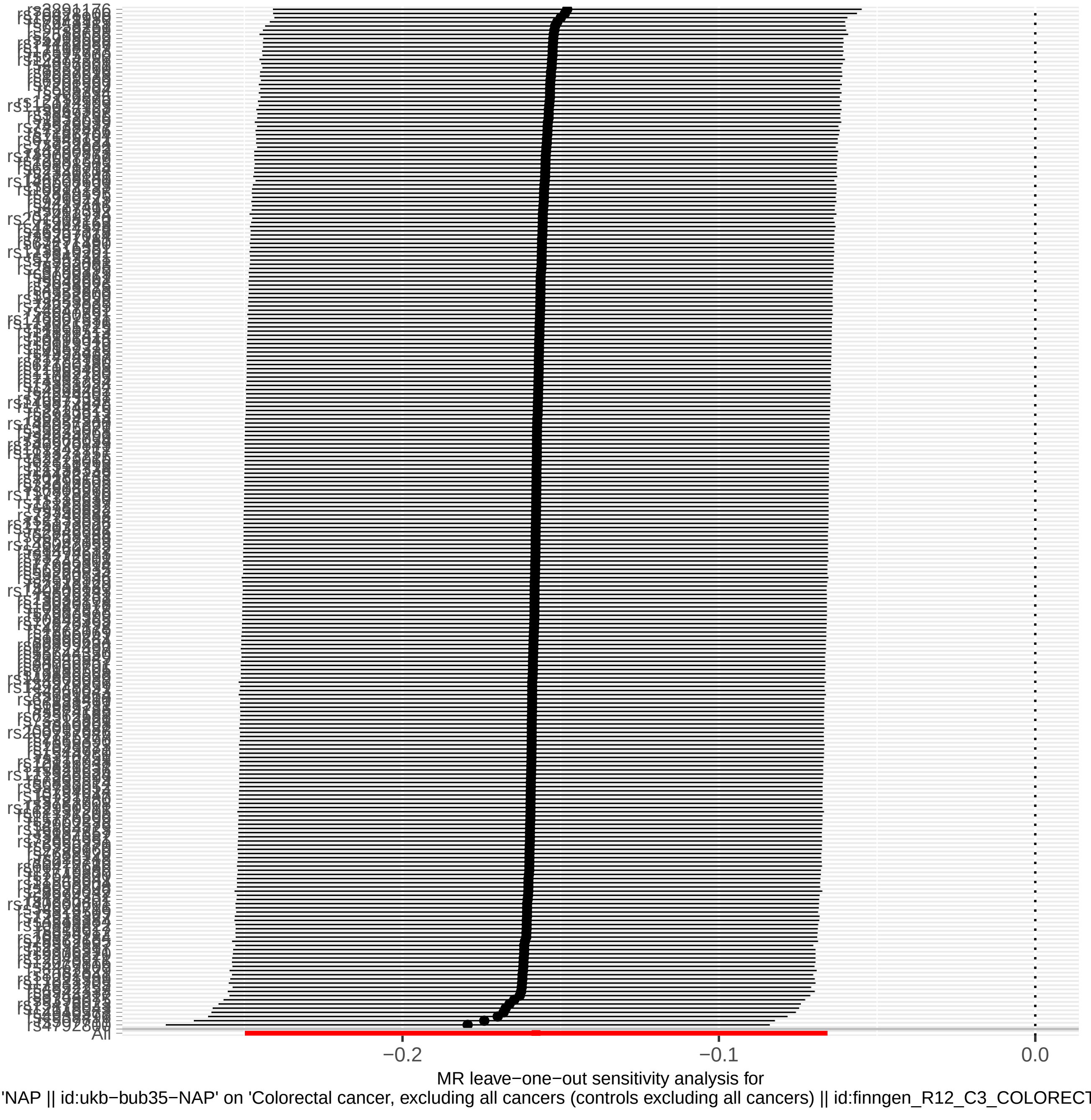

rs3891176
rs4792800
All
−0.2
−0.1
0.0
MR leave−one−out sensitivity analysis for
'NAP || id:ukb−bub35−NAP' on 'Colorectal cancer, excluding all cancers (controls excluding all cancers) || id:finngen_R12_C3_COLORECT

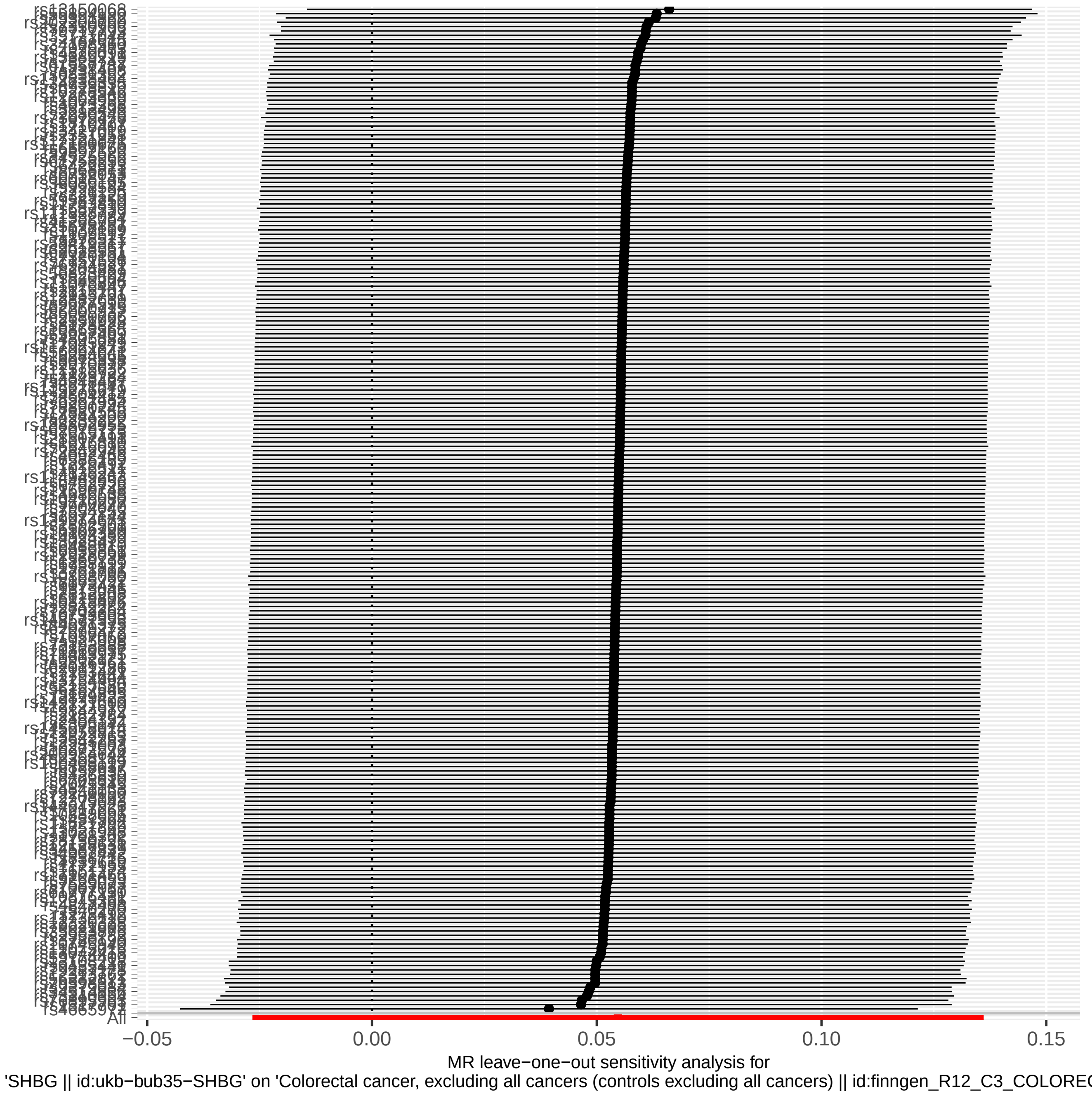
All
−0.05
0.00
0.05
0.10
0.15
MR leave−one−out sensitivity analysis for
'SHBG || id:ukb−bub35−SHBG' on 'Colorectal cancer, excluding all cancers (controls excluding all cancers) || id:finngen_R12_C3_COLOREC

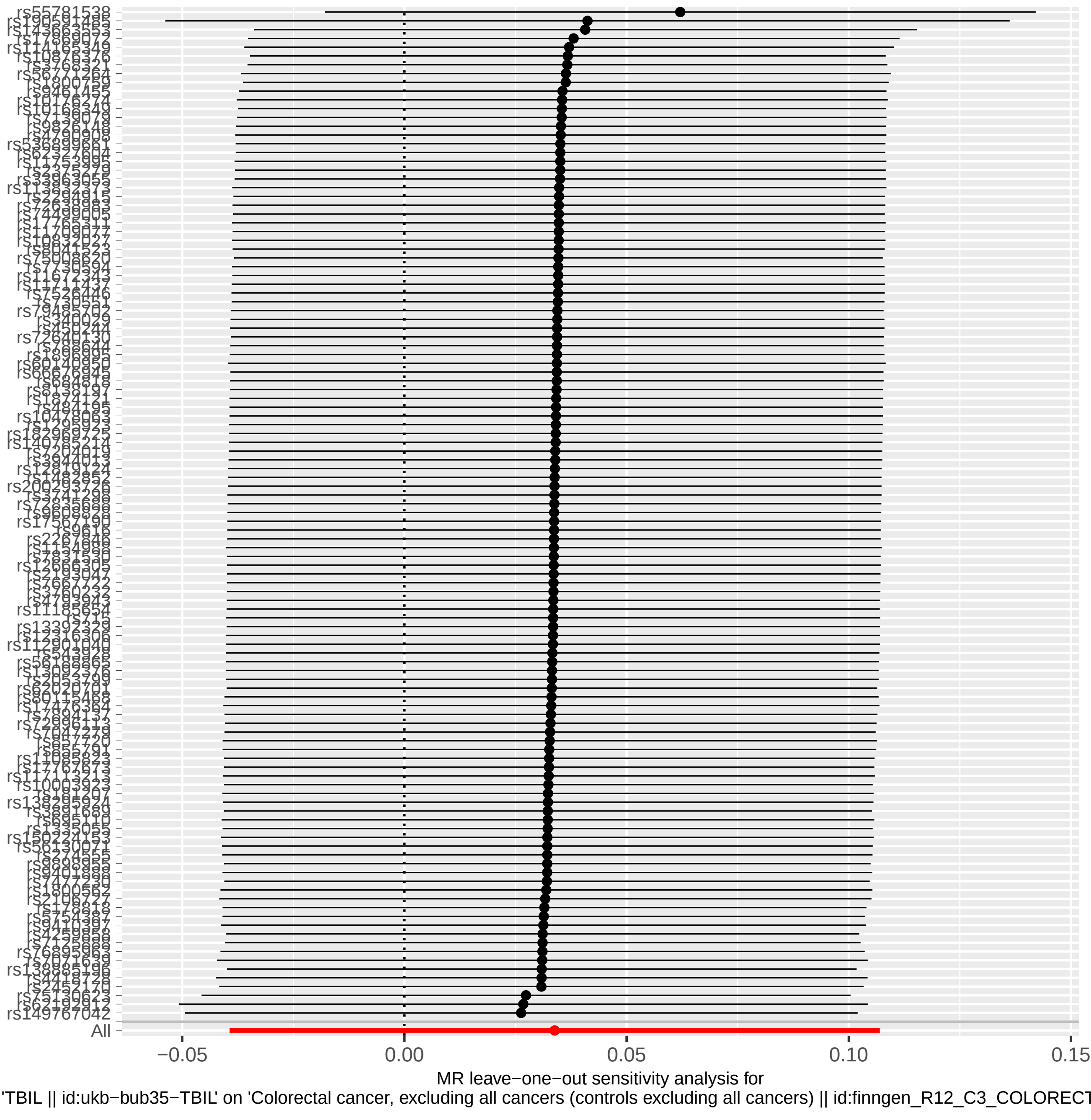

rs55781538
rs149767042
All
−0.05
0.00
0.05
0.10
0.15
MR leave−one−out sensitivity analysis for
'TBIL || id:ukb−bub35−TBIL' on 'Colorectal cancer, excluding all cancers (controls excluding all cancers) || id:finngen_R12_C3_COLORECT

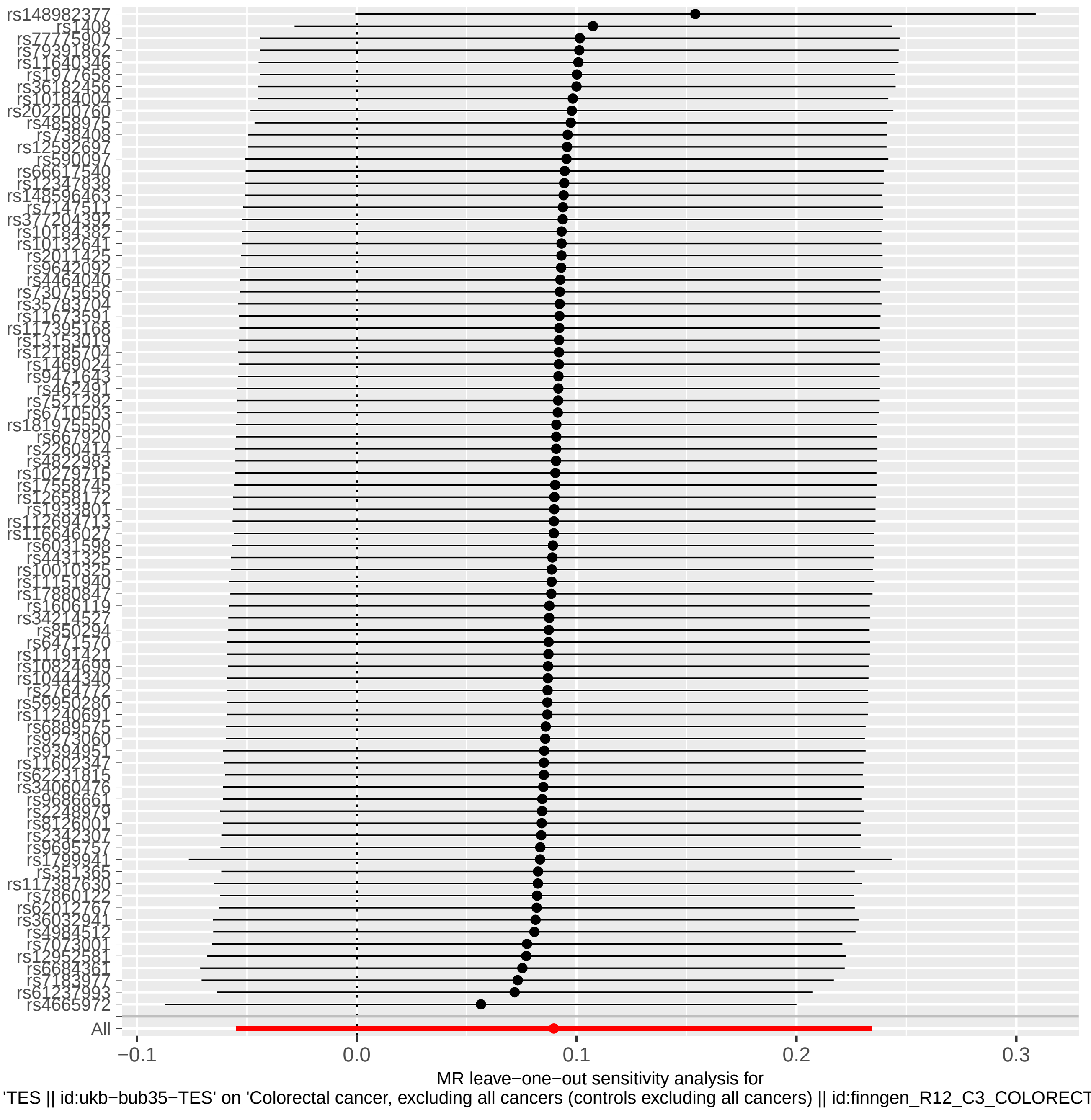
rs148982377
rs1408
rs77775907
rs79391862
rs11640346
rs1977658
rs36182456
rs10184004
rs202200760
rs4858975
rs738408
rs12592697
rs590097
rs66617540
rs12347838
rs148596463
rs7147511
rs377204392
rs10184382
rs10132641
rs2011425
rs9642092
rs4464040
rs73075656
rs35783704
rs11673591
rs117395168
rs13153019
rs12185704
rs1469024
rs9471643
rs462491
rs7521292
rs6710503
rs181975550
rs667920
rs2260414
rs4822983
rs10279715
rs17558745
rs12658172
rs1933801
rs112694713
rs116646027
rs6031598
rs4431325
rs10010325
rs11151940
rs17880847
rs1606119
rs34214527
rs850294
rs6471570
rs11191421
rs10824699
rs10444340
rs2764772
rs59950280
rs11240691
rs6889575
rs9273060
rs9394951
rs11602347
rs62231815
rs34060476
rs9686661
rs2248979
rs8126001
rs2342307
rs9695757
rs1799941
rs351365
rs117387630
rs7860122
rs62012767
rs36032941
rs4984512
rs7073001
rs12952581
rs6684361
rs7183977
rs61237993
rs4665972
All
−0.1
0.0
0.1
0.2
0.3
MR leave−one−out sensitivity analysis for
'TES || id:ukb−bub35−TES' on 'Colorectal cancer, excluding all cancers (controls excluding all cancers) || id:finngen_R12_C3_COLORECT

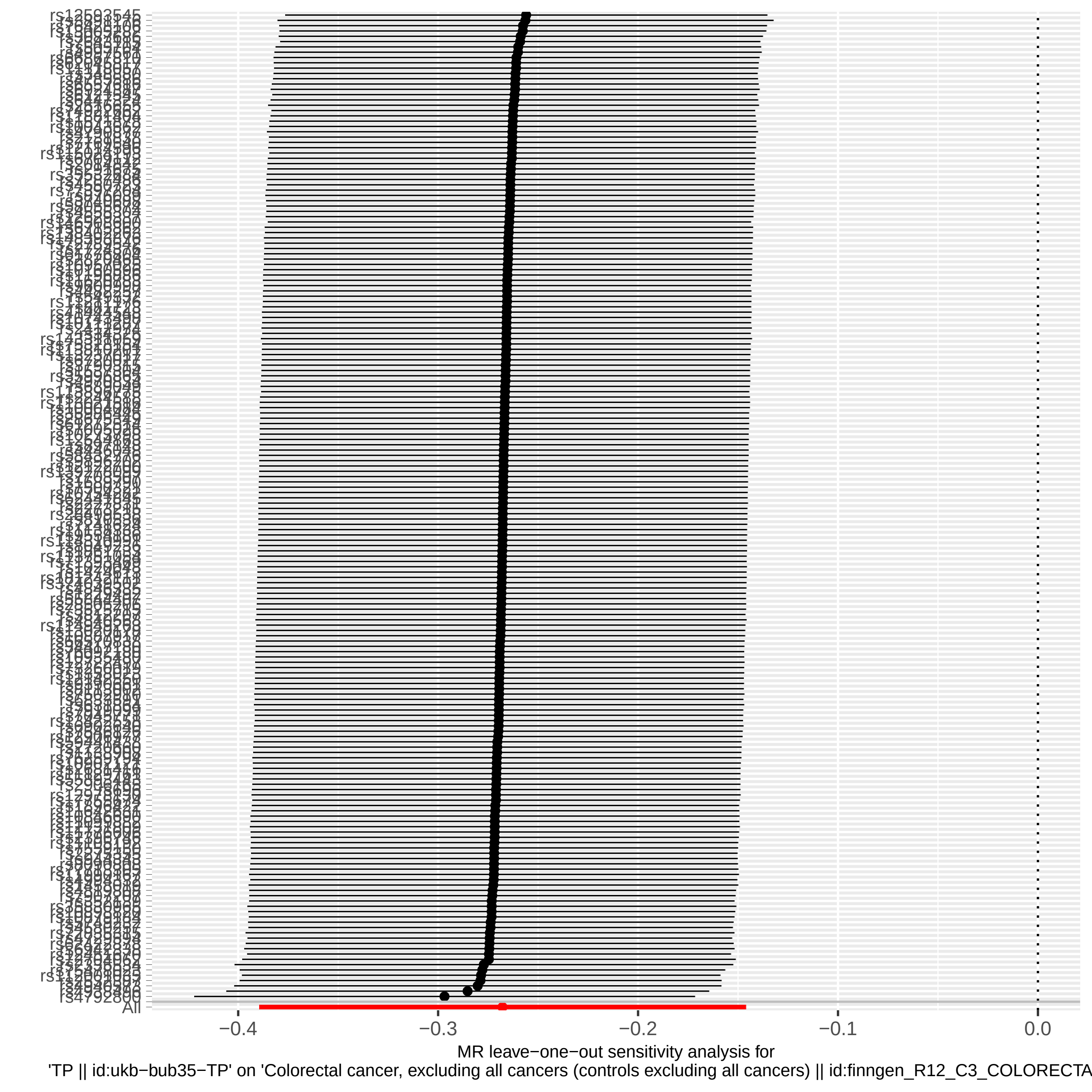

rs12503545
All
−0.4
−0.3
−0.2
−0.1
0.0
MR leave−one−out sensitivity analysis for
'TP || id:ukb−bub35−TP' on 'Colorectal cancer, excluding all cancers (controls excluding all cancers) || id:finngen_R12_C3_COLORECTA

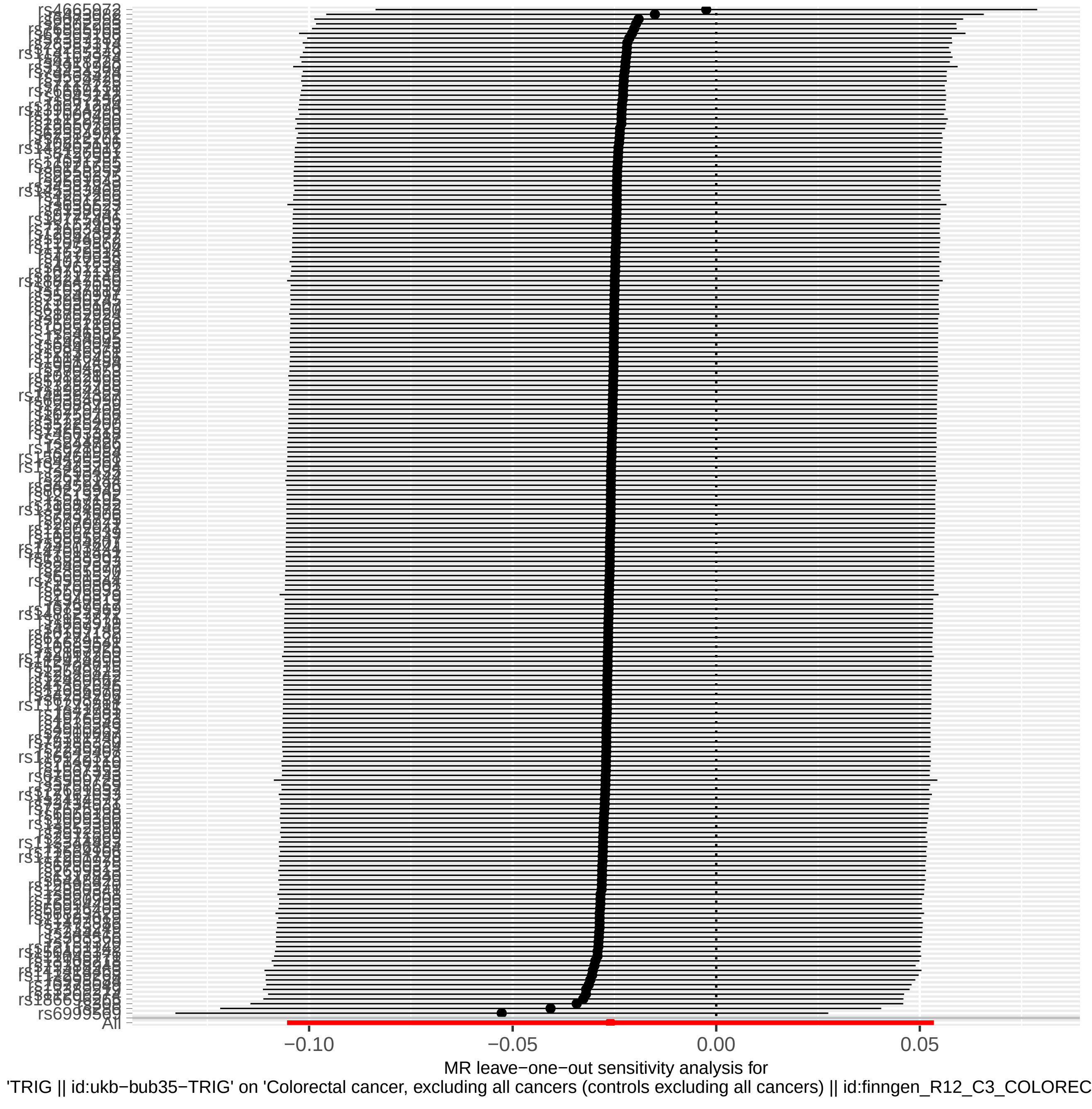
rs4665972
rs6999569
All
−0.10
−0.05
0.00
0.05
MR leave−one−out sensitivity analysis for
'TRIG || id:ukb−bub35−TRIG' on 'Colorectal cancer, excluding all cancers (controls excluding all cancers) || id:finngen_R12_C3_COLOREC

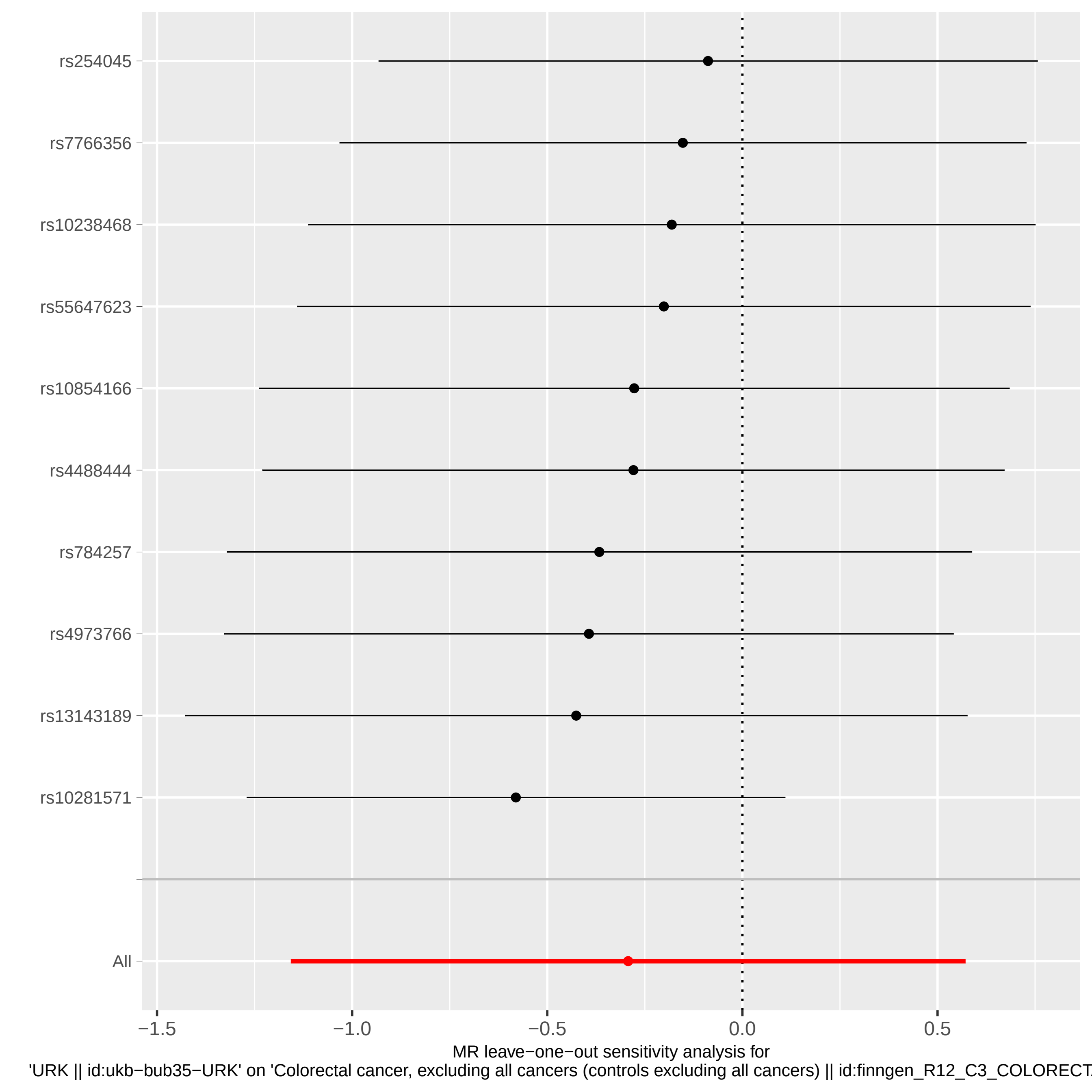
rs254045
rs7766356
rs10238468
rs55647623
rs10854166
rs4488444
rs784257
rs4973766
rs13143189
rs10281571
All
−1.5
−1.0
−0.5
0.0
0.5
MR leave−one−out sensitivity analysis for
'URK || id:ukb−bub35−URK' on 'Colorectal cancer, excluding all cancers (controls excluding all cancers) || id:finngen_R12_C3_COLORECT

Supplementary Figure 2. All scatter plot visualization results.

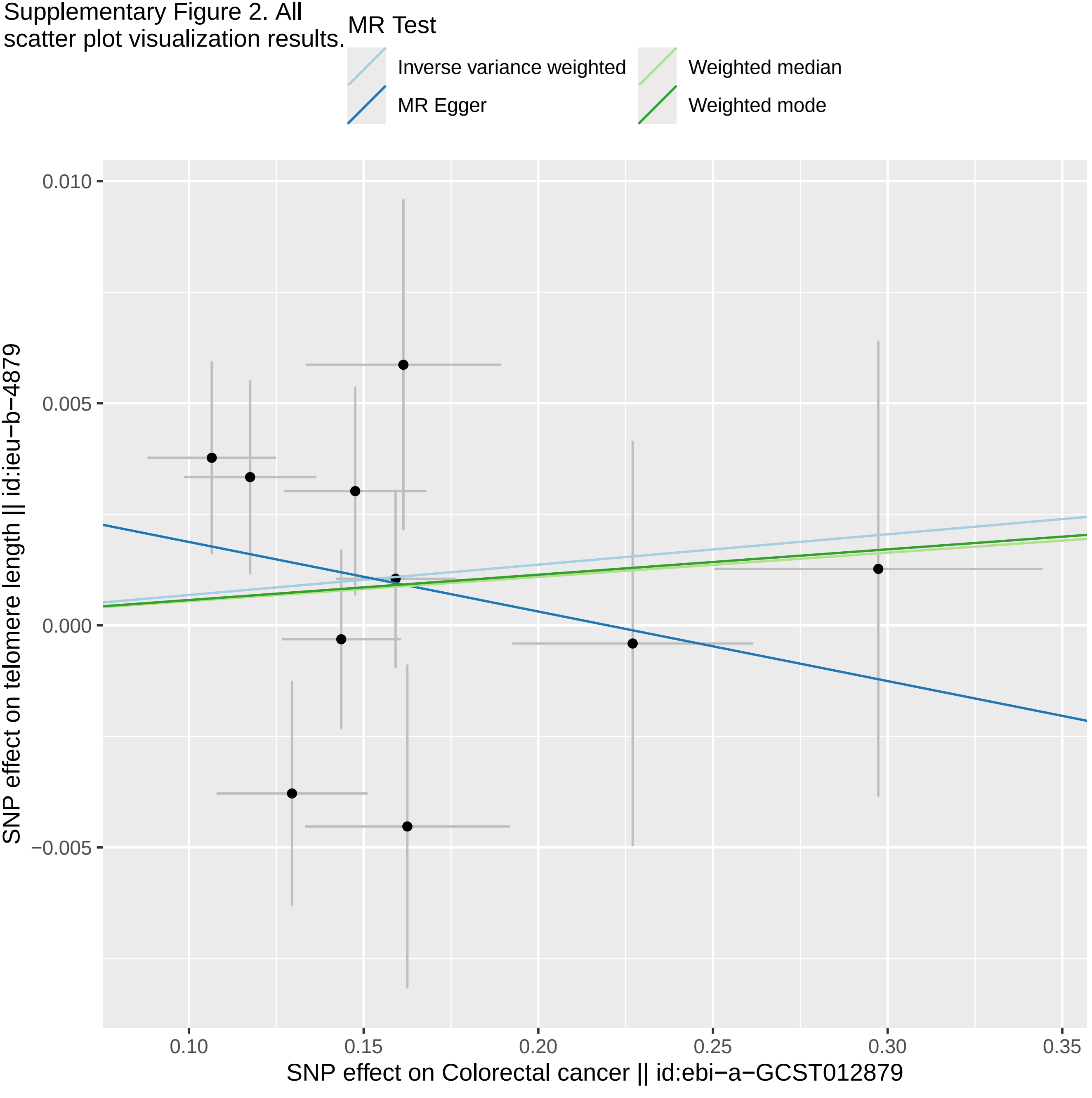

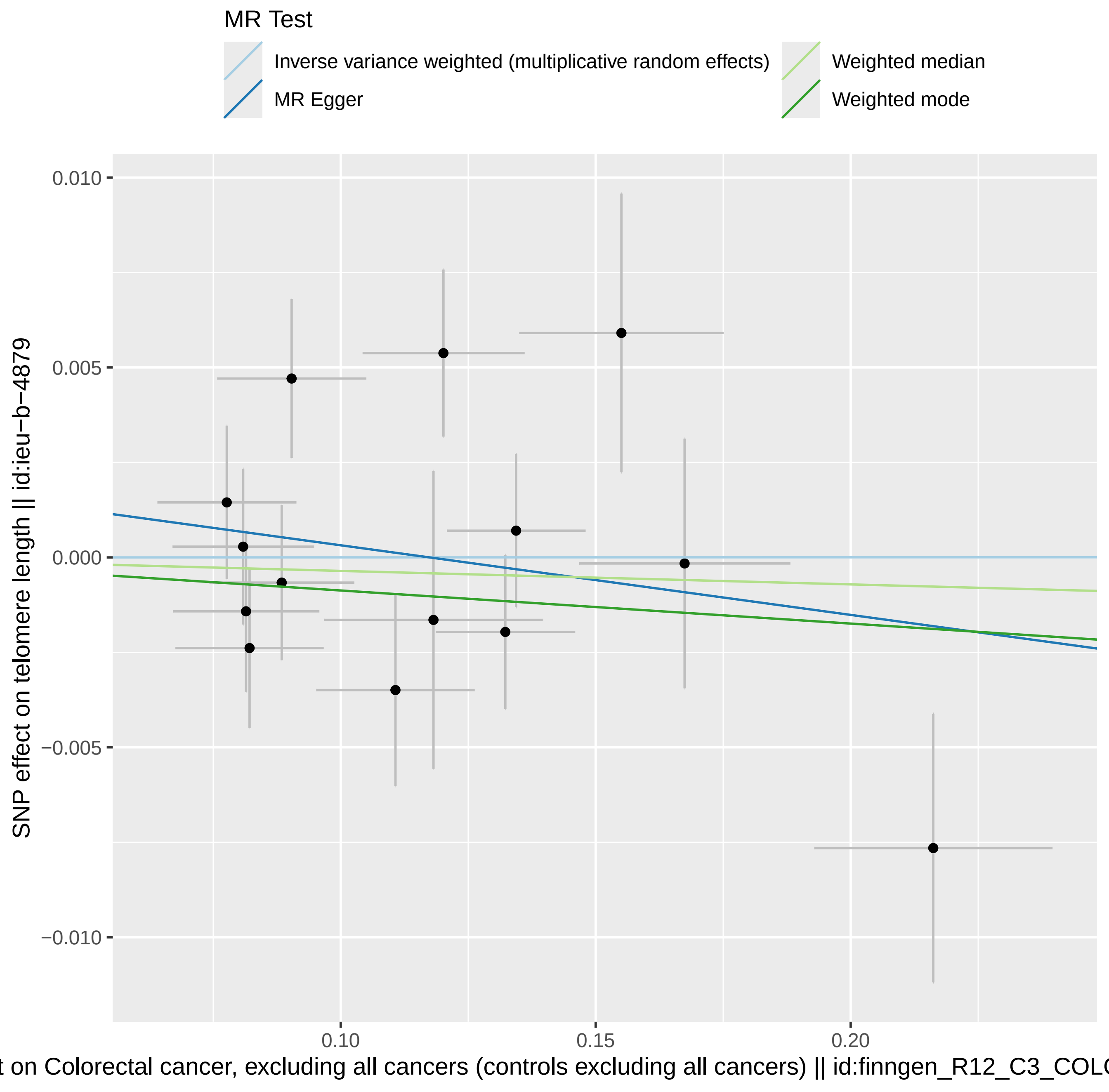

MR Test
Inverse variance weighted (multiplicative random effects)
MR Egger
Weighted median
Weighted mode
0.010
0.005
0.000
−0.005
−0.010
SNP effect on telomere length || id:ieu−b−4879
0.10
0.15
0.20
t on Colorectal cancer, excluding all cancers (controls excluding all cancers) || id:finngen_R12_C3_COLO

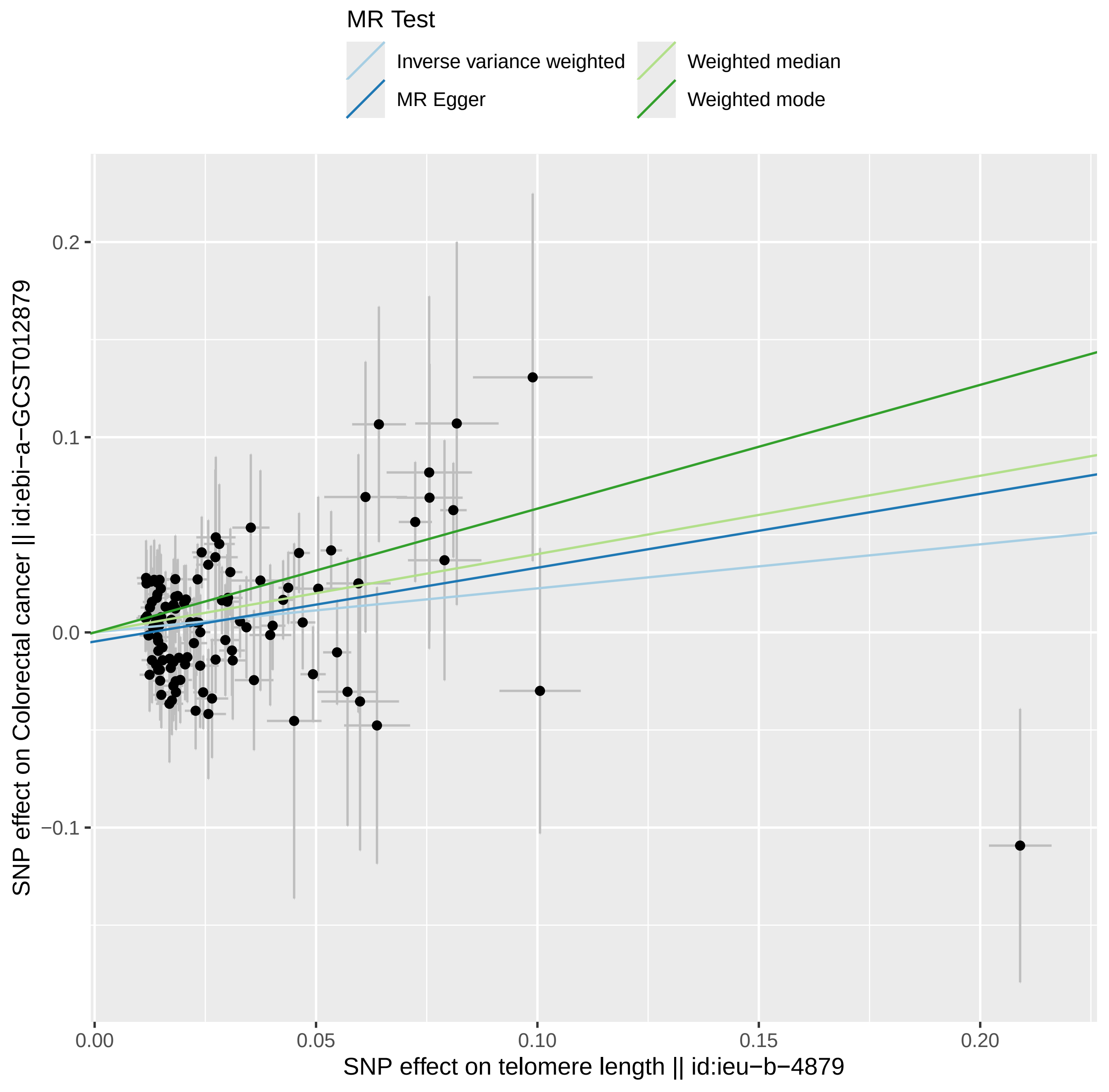
MR Test
Inverse variance weighted
MR Egger
Weighted median
Weighted mode
0.2
0.1
0.0
−0.1
0.00
0.05
0.10
0.15
0.20
SNP effect on Colorectal cancer || id:ebi−a−GCST012879
SNP effect on telomere length || id:ieu−b−4879

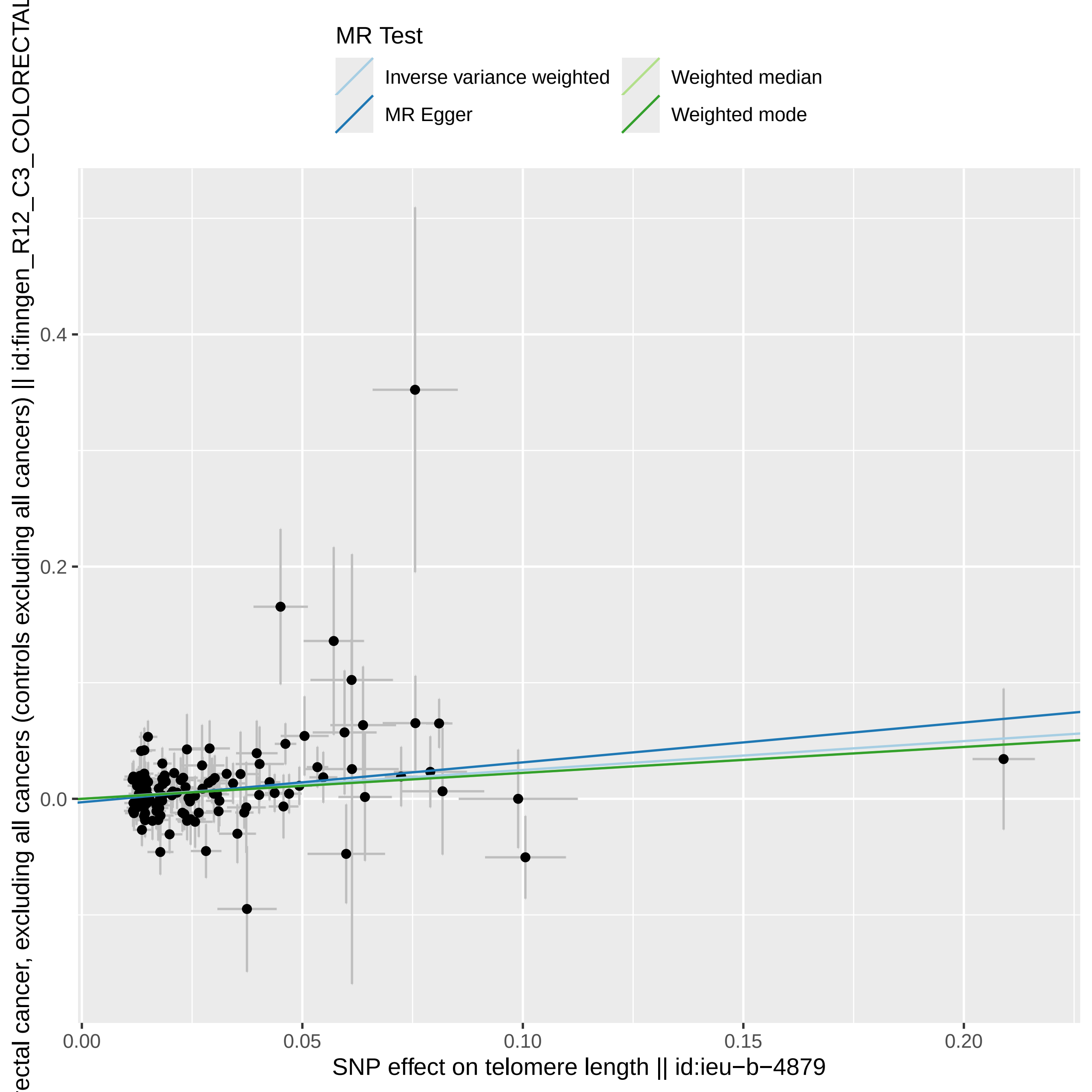

MR Test
Inverse variance weighted
MR Egger
Weighted median
Weighted mode
ectal cancer, excluding all cancers (controls excluding all cancers) || id:finngen_R12_C3_COLORECTAL
0.4
0.2
0.0
0.00
0.05
0.10
0.15
0.20
SNP effect on telomere length || id:ieu−b−4879

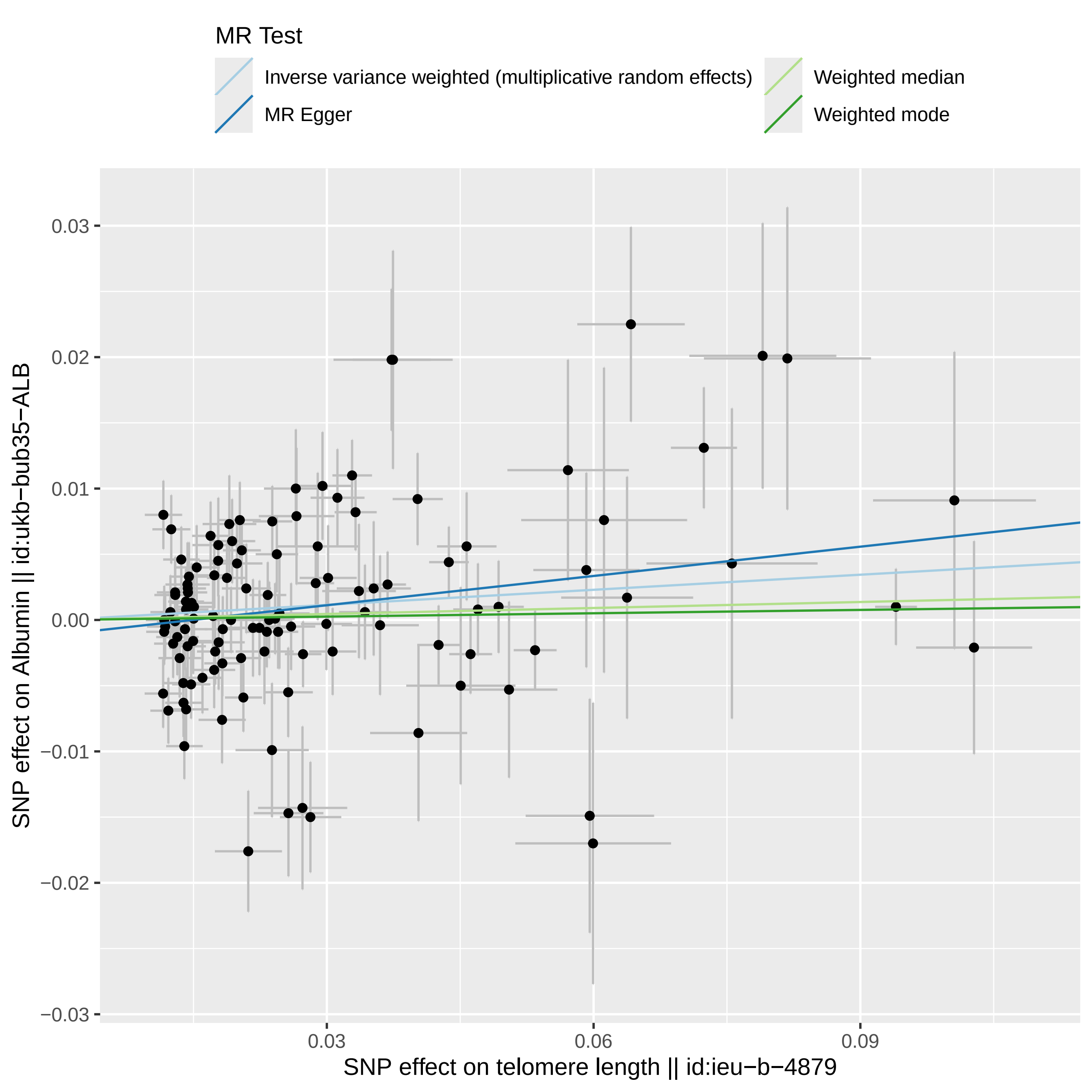

MR Test
Inverse variance weighted (multiplicative random effects)
MR Egger
Weighted median
Weighted mode
0.03
0.02
0.01
0.00
−0.01
−0.02
−0.03
0.03
0.06
0.09
SNP effect on Albumin || id:ukb−bub35−ALB
SNP effect on telomere length || id:ieu−b−4879

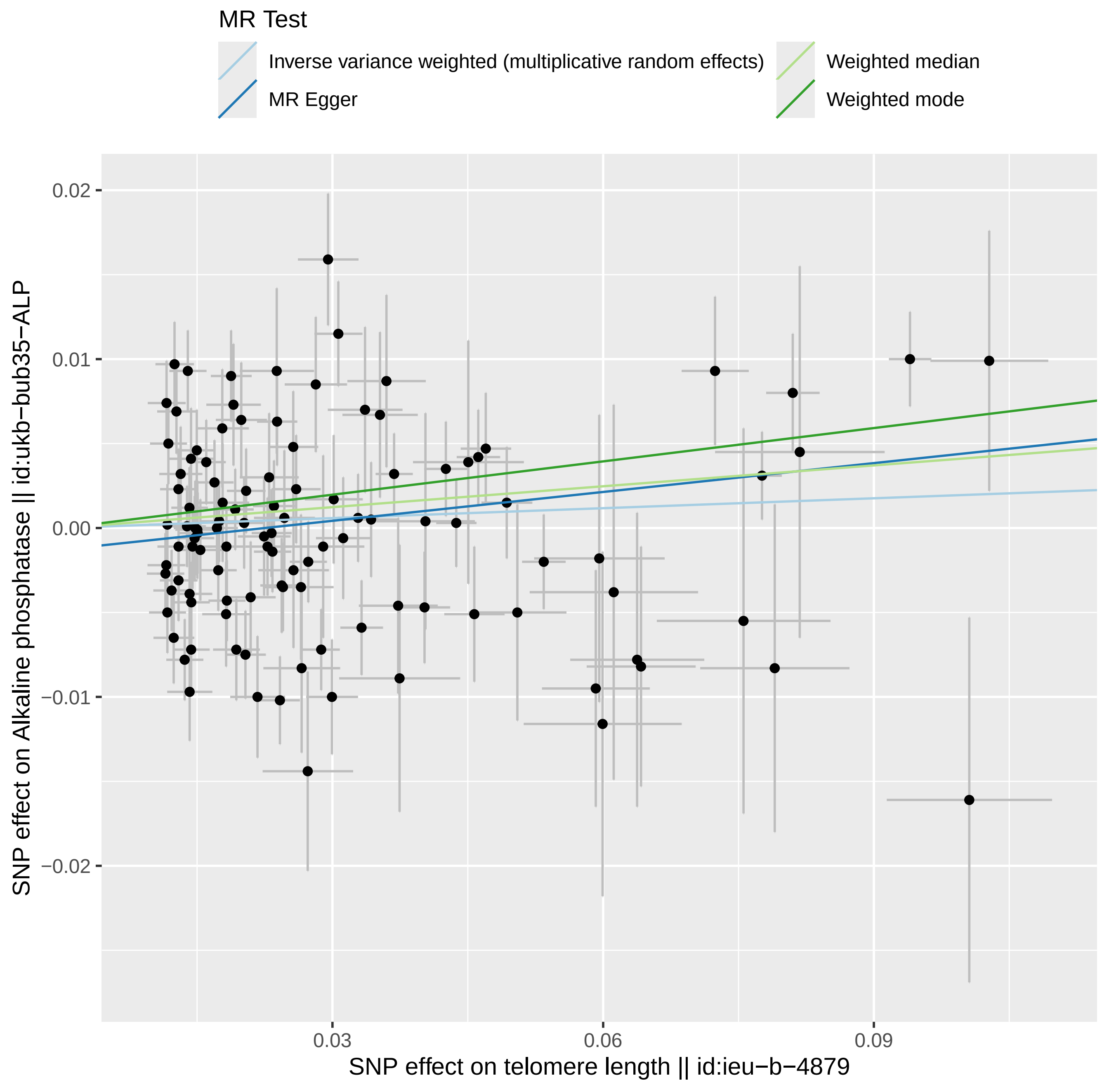
MR Test
Inverse variance weighted (multiplicative random effects)
MR Egger
Weighted median
Weighted mode
0.02
0.01
0.00
−0.01
−0.02
SNP effect on Alkaline phosphatase || id:ukb−bub35−ALP
0.03
0.06
0.09
SNP effect on telomere length || id:ieu−b−4879

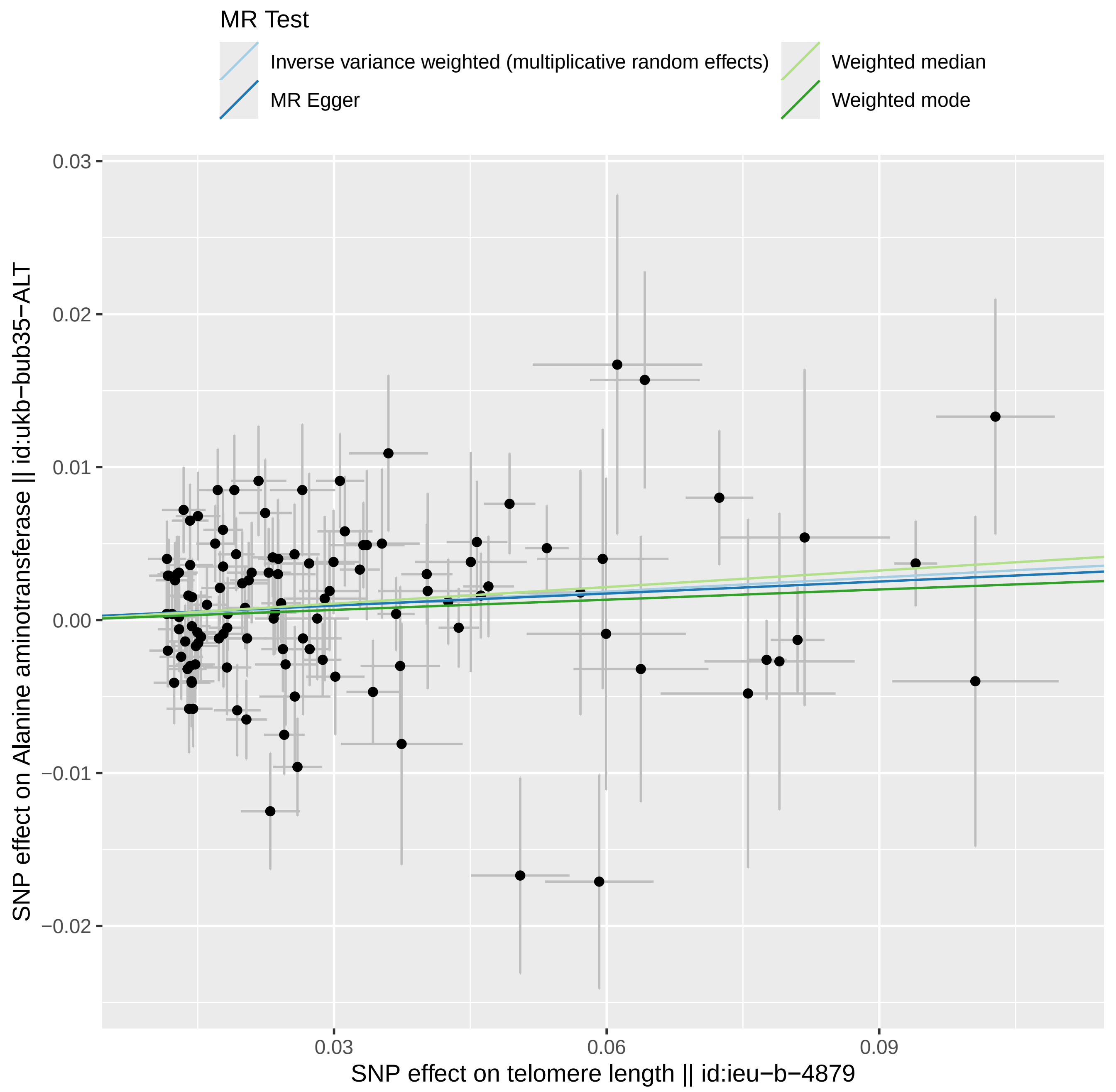
MR Test
Inverse variance weighted (multiplicative random effects)
MR Egger
Weighted median
Weighted mode
0.03
0.02
0.01
0.00
−0.01
−0.02
SNP effect on Alanine aminotransferase || id:ukb−bub35−ALT
0.03
0.06
0.09
SNP effect on telomere length || id:ieu−b−4879

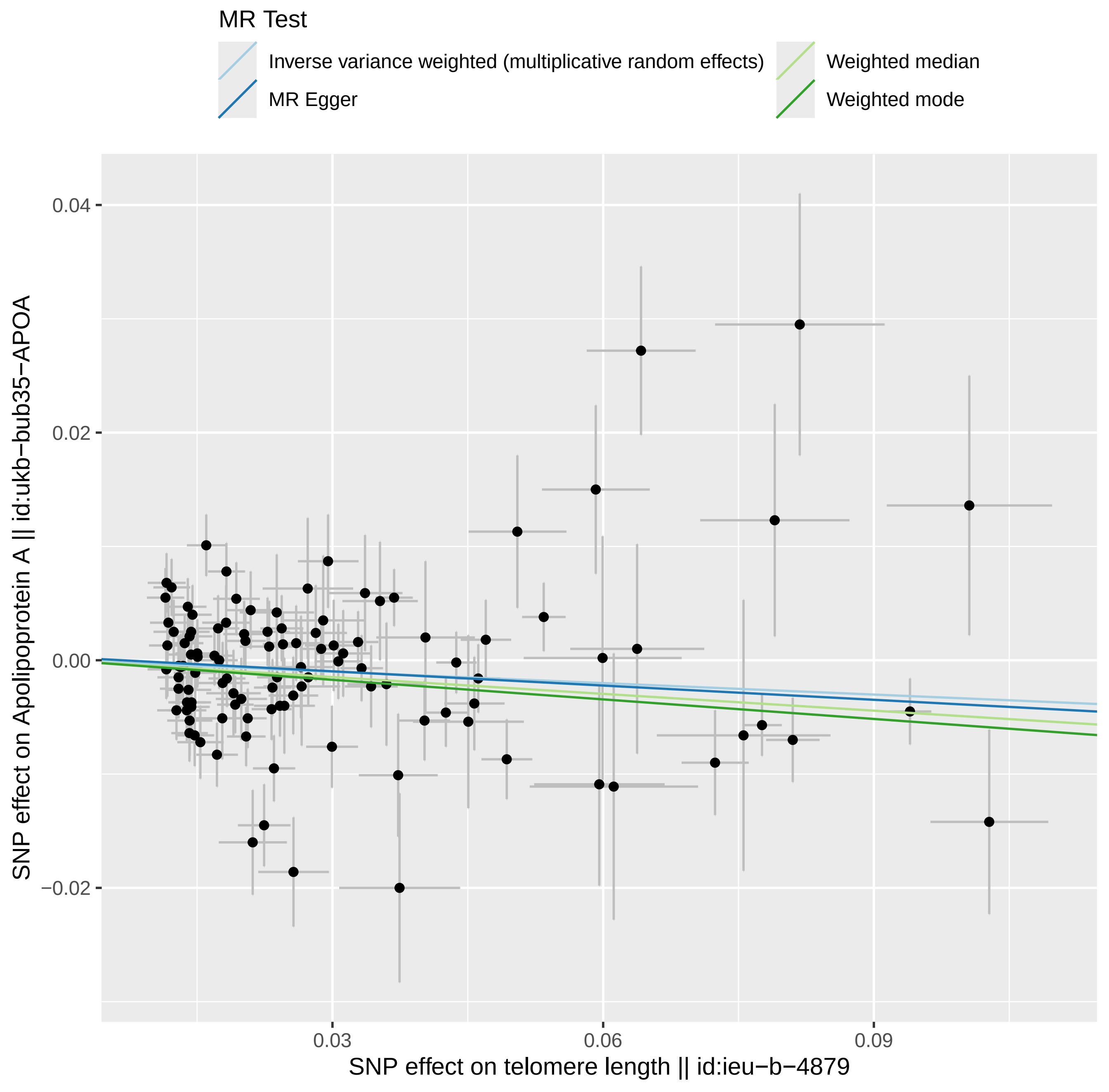

MR Test
Inverse variance weighted (multiplicative random effects)
MR Egger
Weighted median
Weighted mode
0.04
0.02
0.00
−0.02
0.03
0.06
0.09
SNP effect on Apolipoprotein A || id:ukb−bub35−APOA
SNP effect on telomere length || id:ieu−b−4879

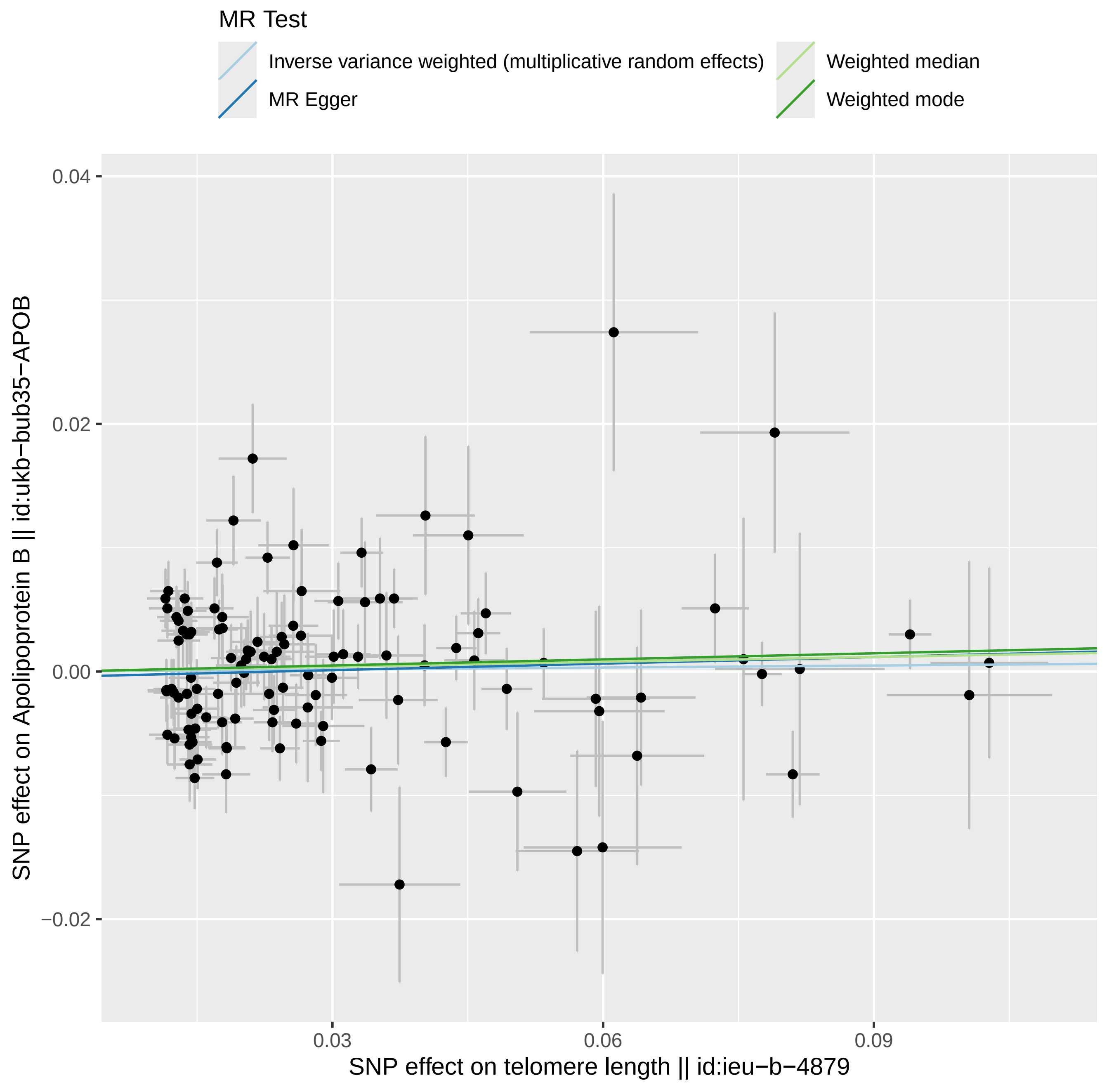
MR Test
Inverse variance weighted (multiplicative random effects)
MR Egger
Weighted median
Weighted mode
0.04
0.02
0.00
−0.02
0.03
0.06
0.09
SNP effect on Apolipoprotein B || id:ukb−bub35−APOB
SNP effect on telomere length || id:ieu−b−4879

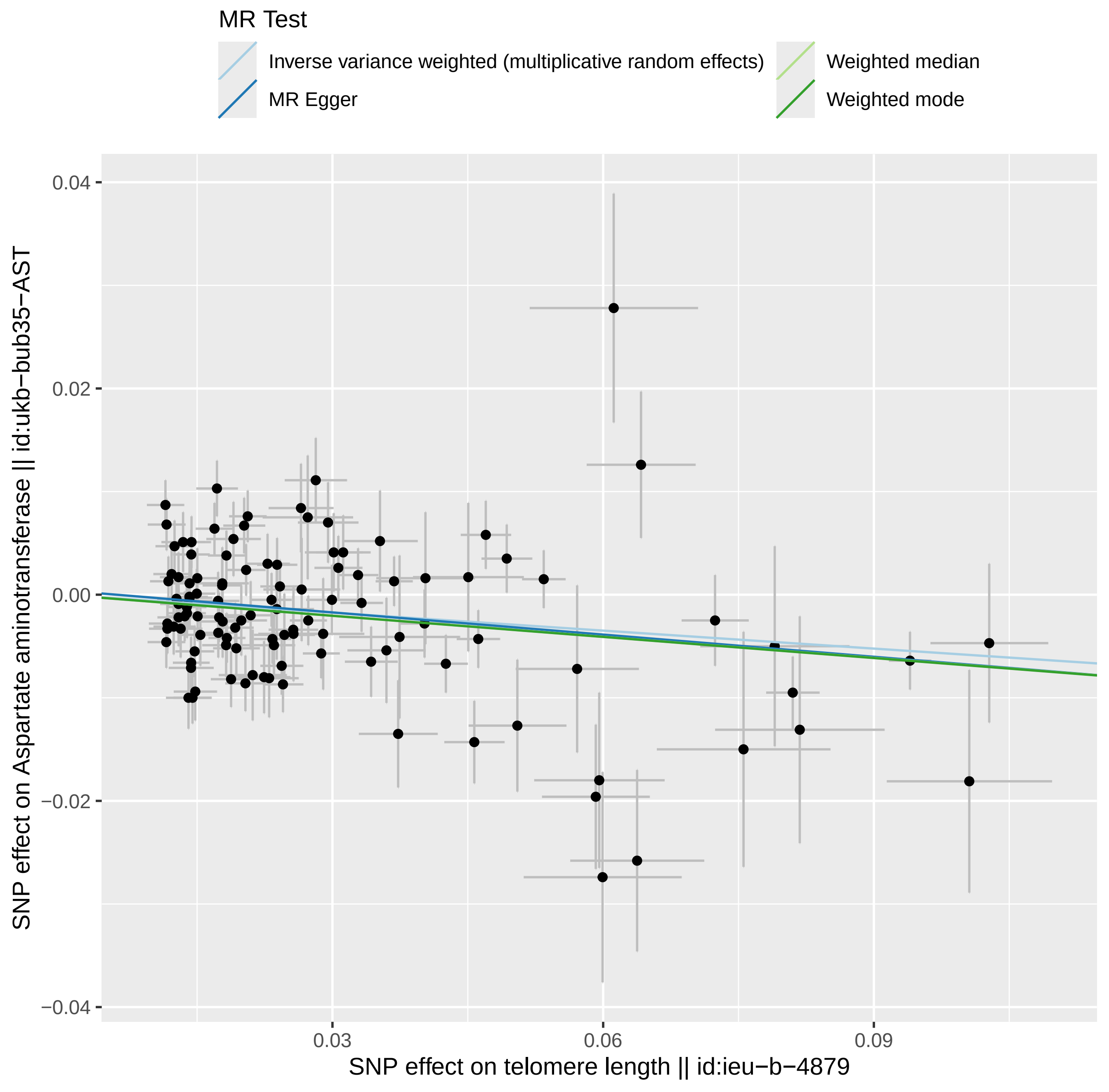

MR Test
Inverse variance weighted (multiplicative random effects)
MR Egger
Weighted median
Weighted mode
0.04
0.02
0.00
−0.02
−0.04
SNP effect on Aspartate aminotransferase || id:ukb−bub35−AST
0.03
0.06
0.09
SNP effect on telomere length || id:ieu−b−4879

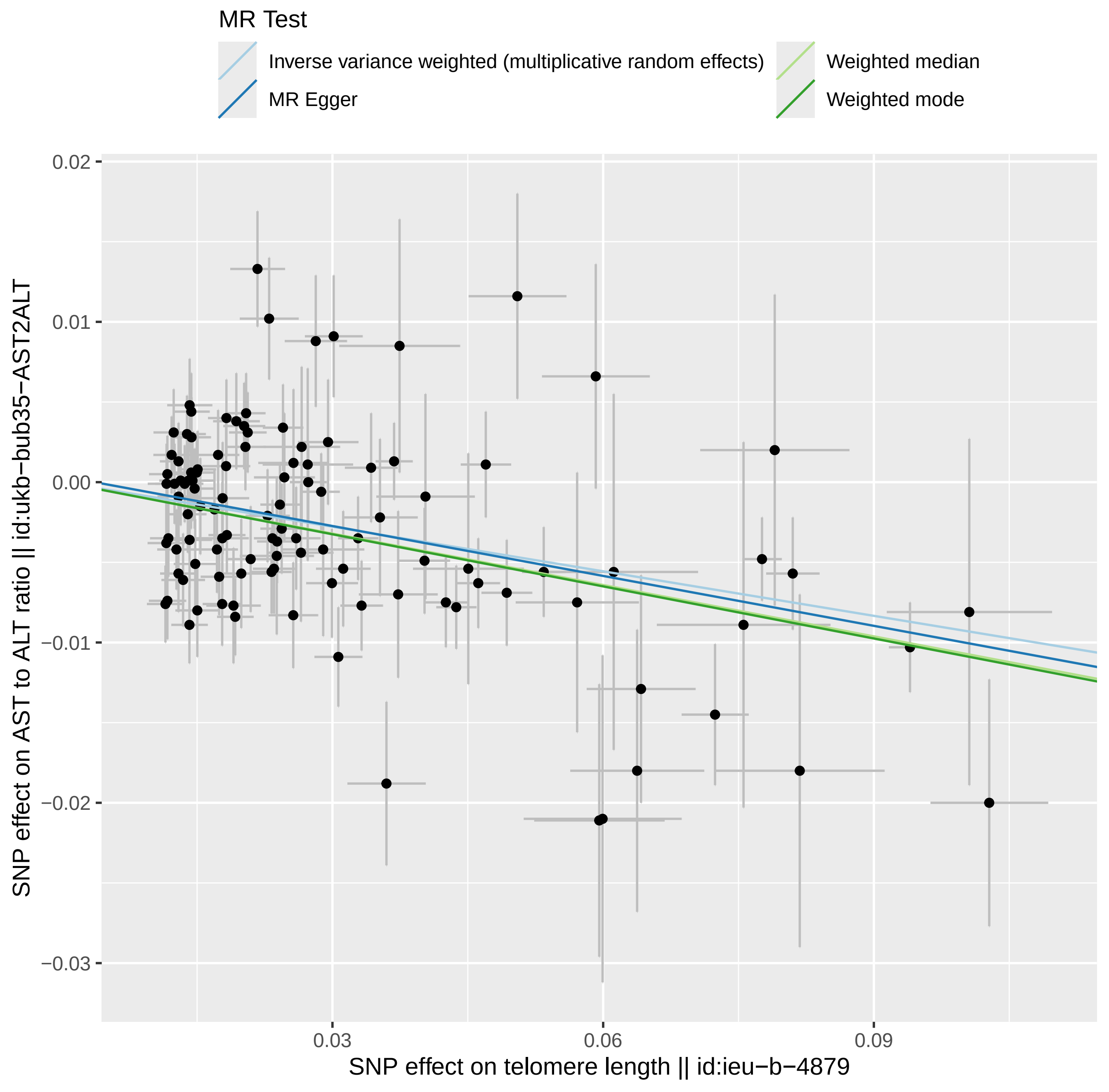
MR Test
Inverse variance weighted (multiplicative random effects)
MR Egger
Weighted median
Weighted mode
0.02
0.01
0.00
−0.01
−0.02
−0.03
0.03
0.06
0.09
SNP effect on AST to ALT ratio || id:ukb−bub35−AST2ALT
SNP effect on telomere length || id:ieu−b−4879

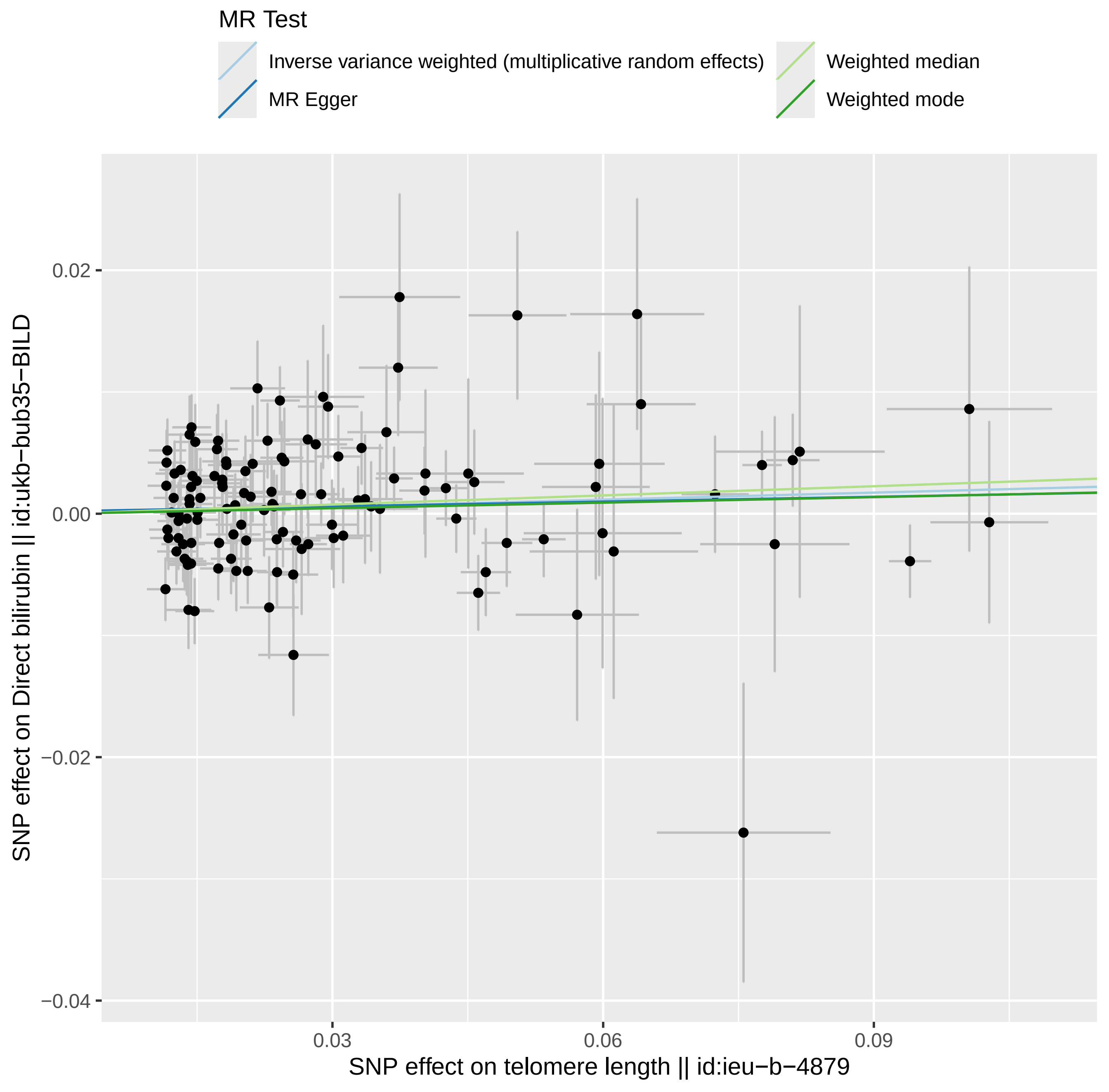

MR Test
Inverse variance weighted (multiplicative random effects)
MR Egger
Weighted median
Weighted mode
0.02
0.00
−0.02
−0.04
0.03
0.06
0.09
SNP effect on Direct bilirubin || id:ukb−bub35−BILD
SNP effect on telomere length || id:ieu−b−4879

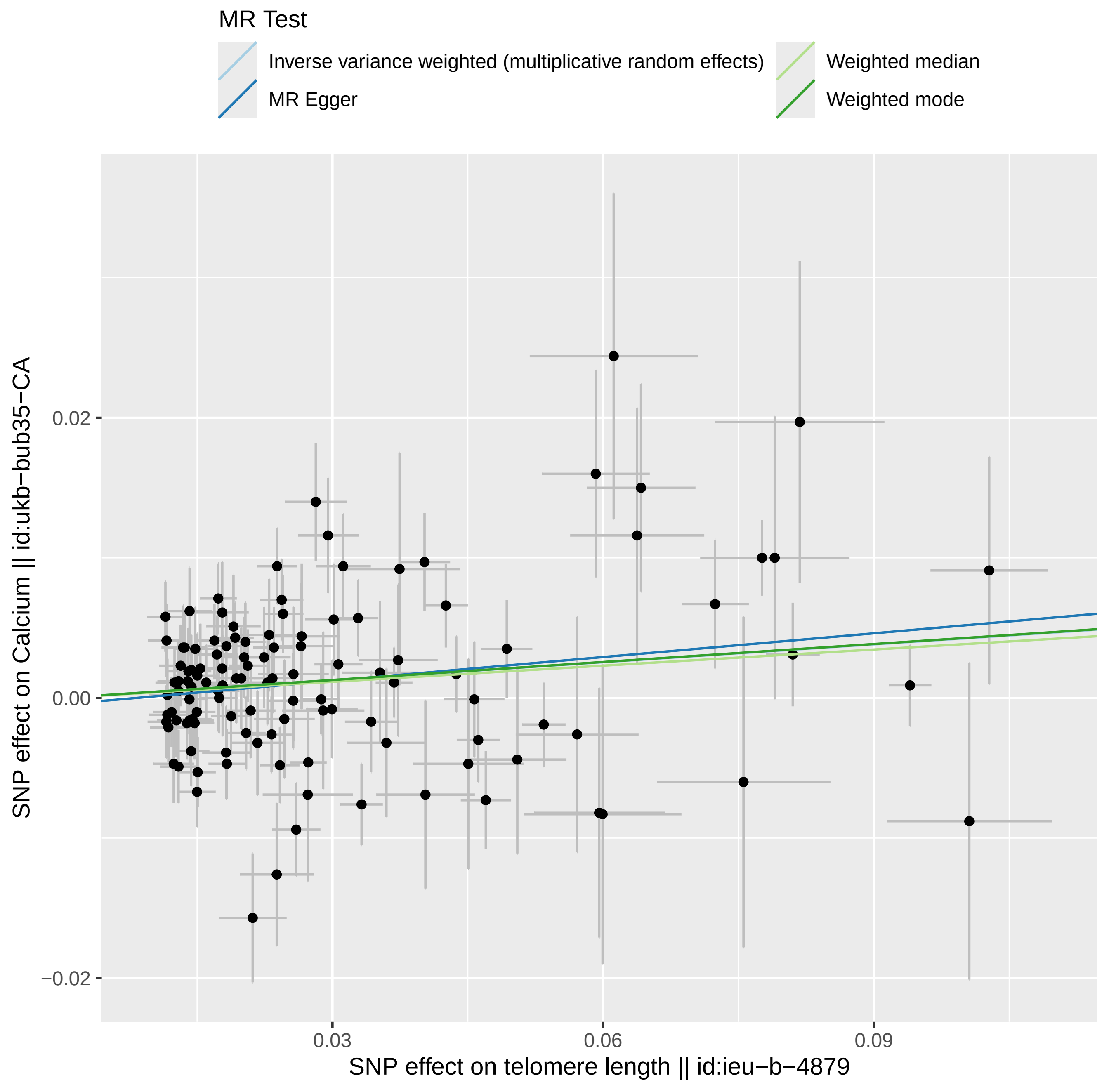
MR Test
Inverse variance weighted (multiplicative random effects)
MR Egger
Weighted median
Weighted mode
SNP effect on Calcium || id:ukb−bub35−CA
0.02
0.00
−0.02
0.03
0.06
0.09
SNP effect on telomere length || id:ieu−b−4879

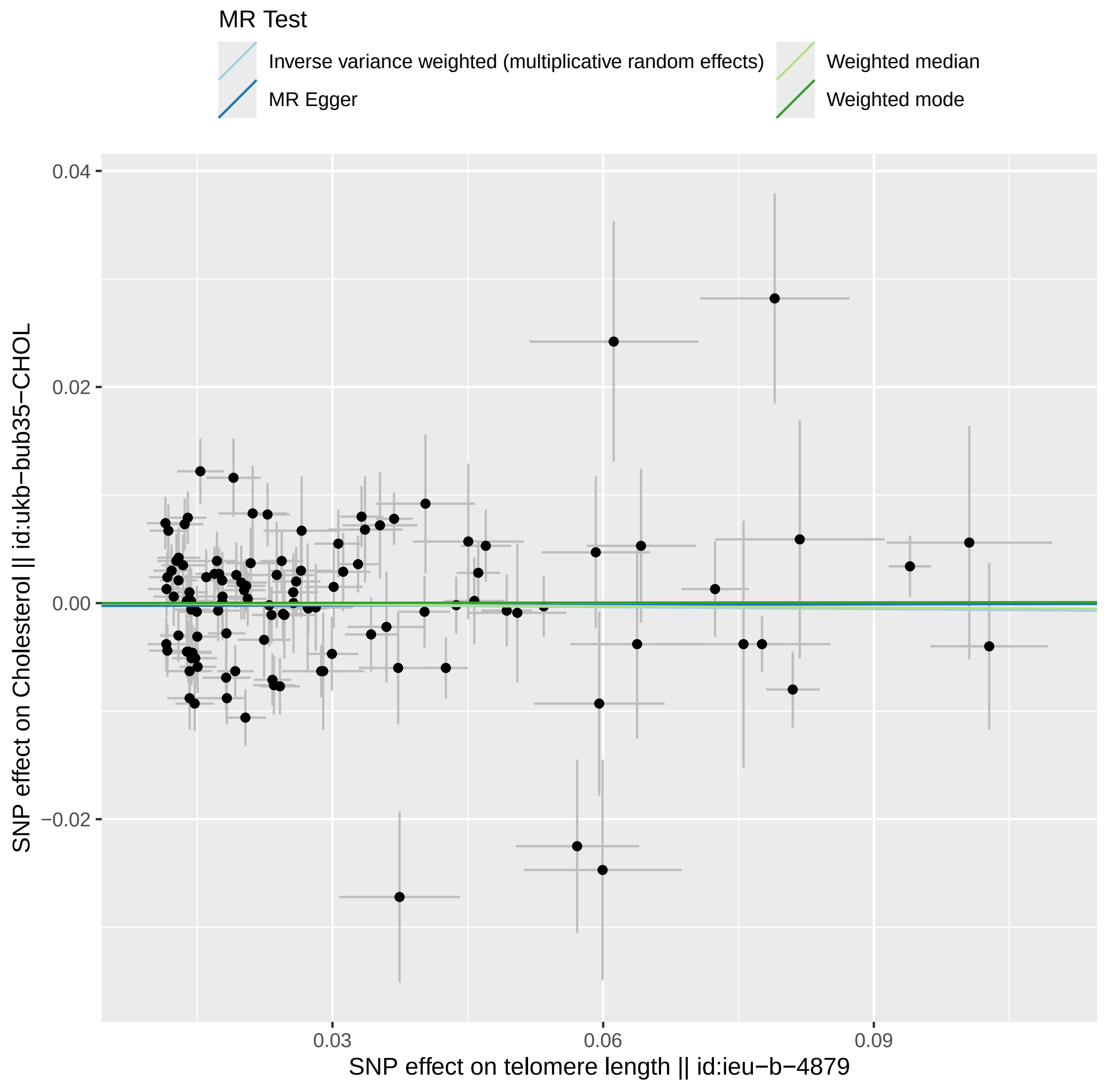
MR Test
Inverse variance weighted (multiplicative random effects)
MR Egger
Weighted median
Weighted mode
0.04
0.02
0.00
−0.02
0.03
0.06
0.09
SNP effect on Cholesterol || id:ukb−bub35−CHOL
SNP effect on telomere length || id:ieu−b−4879

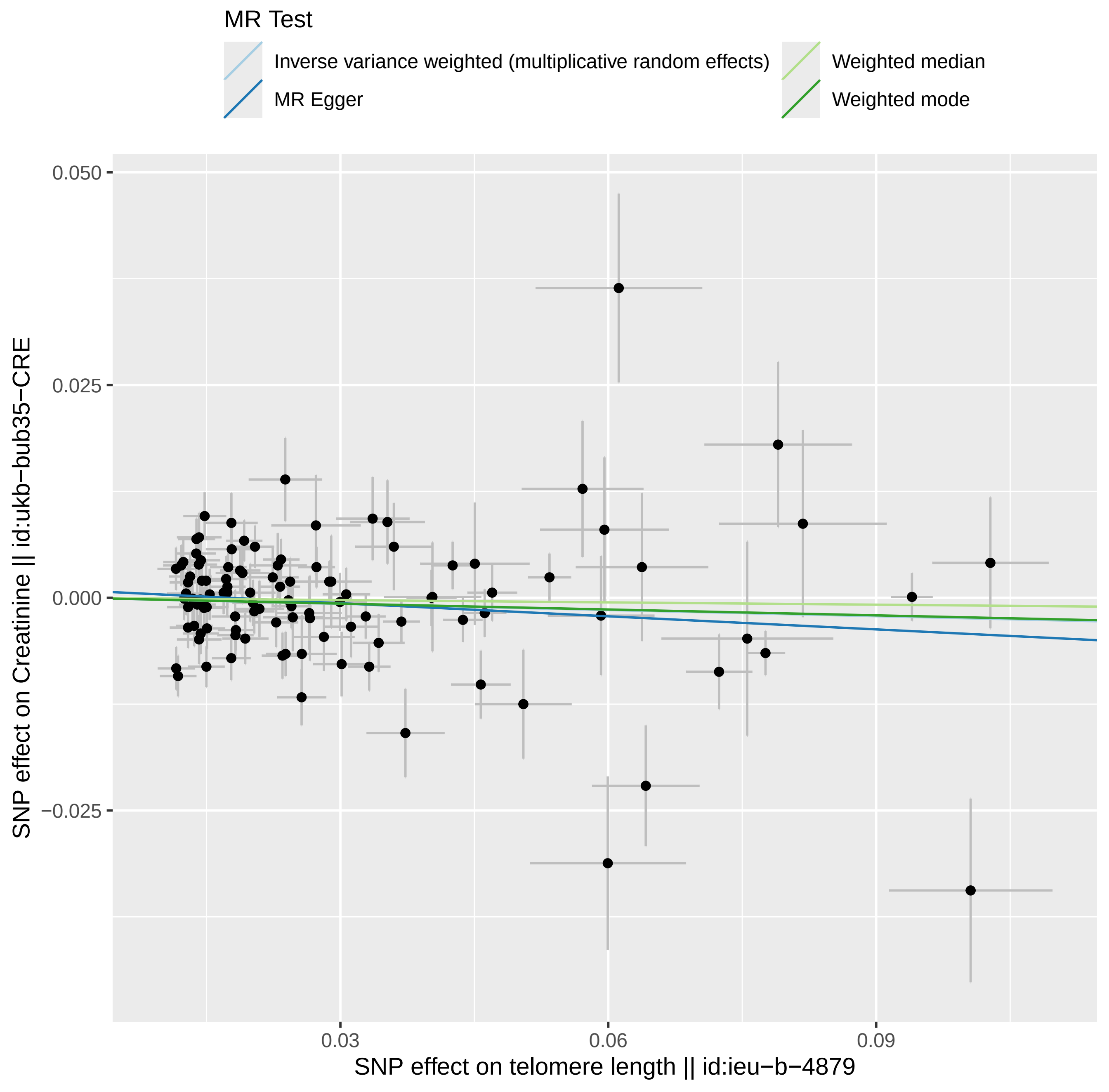

MR Test
Inverse variance weighted (multiplicative random effects)
MR Egger
Weighted median
Weighted mode
0.050
0.025
0.000
−0.025
SNP effect on Creatinine || id:ukb−bub35−CRE
0.03
0.06
0.09
SNP effect on telomere length || id:ieu−b−4879

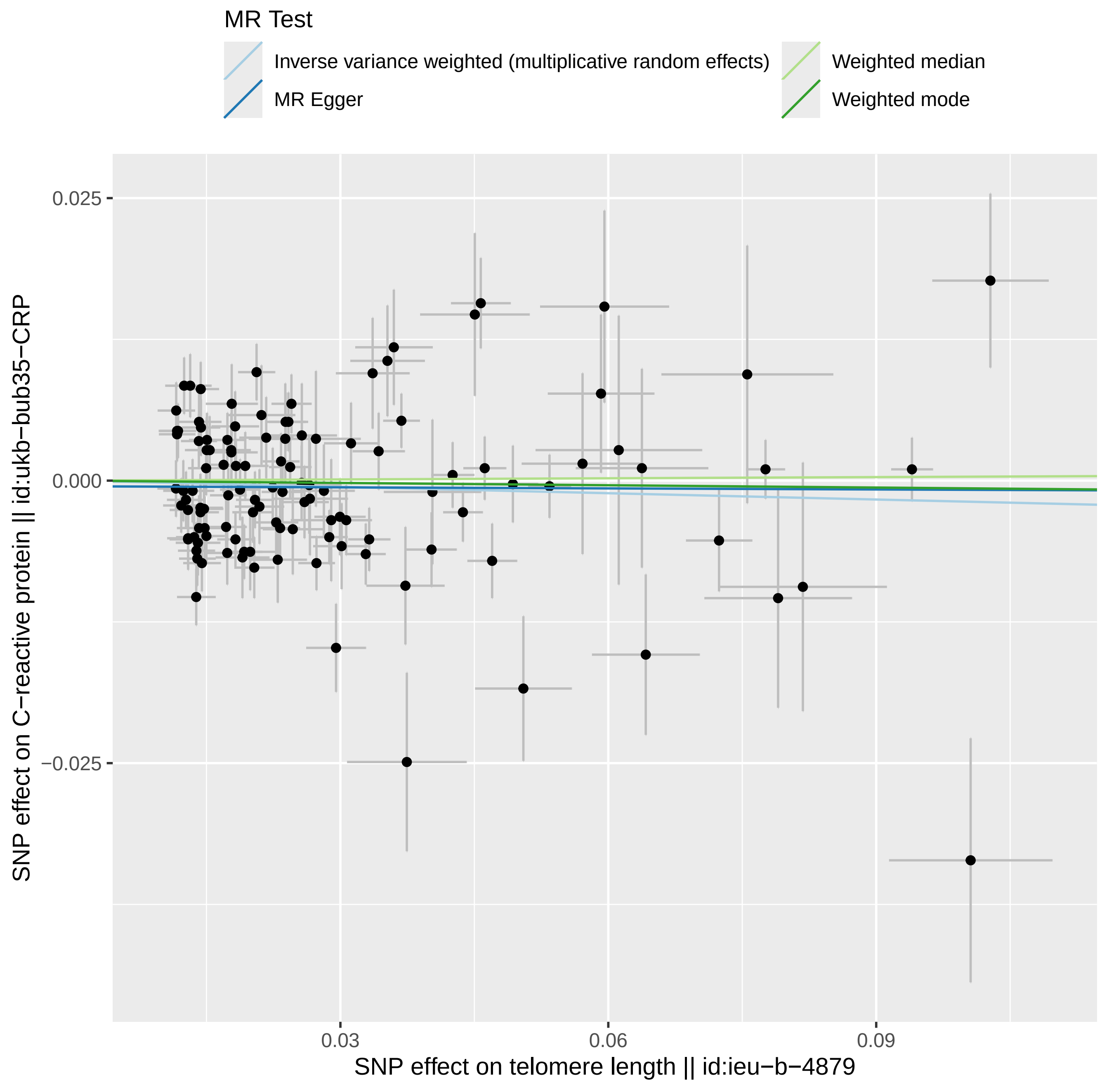
MR Test
Inverse variance weighted (multiplicative random effects)
MR Egger
Weighted median
Weighted mode
0.025
0.000
−0.025
0.03
0.06
0.09
SNP effect on telomere length || id:ieu−b−4879
SNP effect on C−reactive protein || id:ukb−bub35−CRP

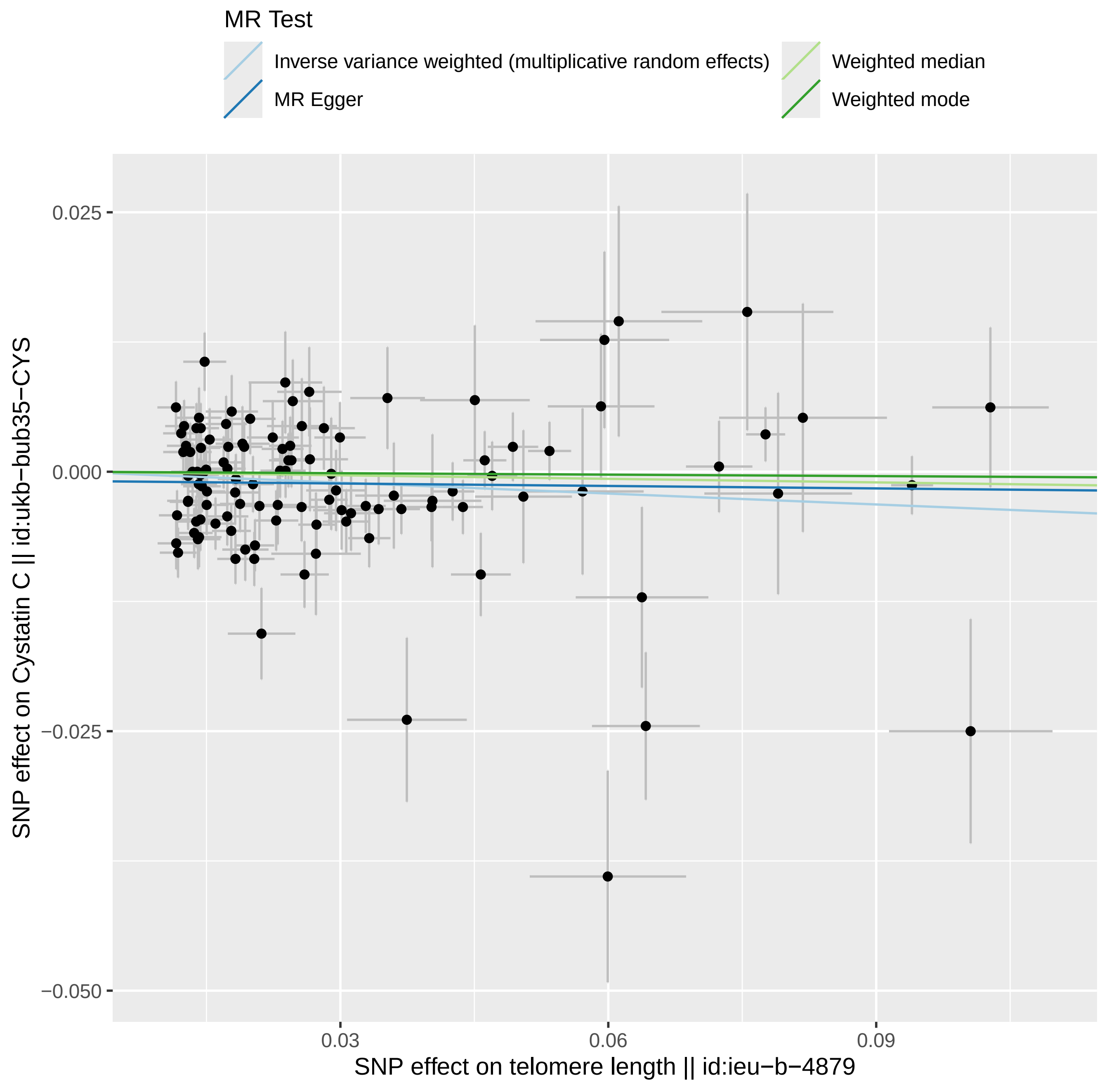

MR Test
Inverse variance weighted (multiplicative random effects)
MR Egger
Weighted median
Weighted mode
0.025
0.000
−0.025
−0.050
0.03
0.06
0.09
SNP effect on Cystatin C || id:ukb−bub35−CYS
SNP effect on telomere length || id:ieu−b−4879

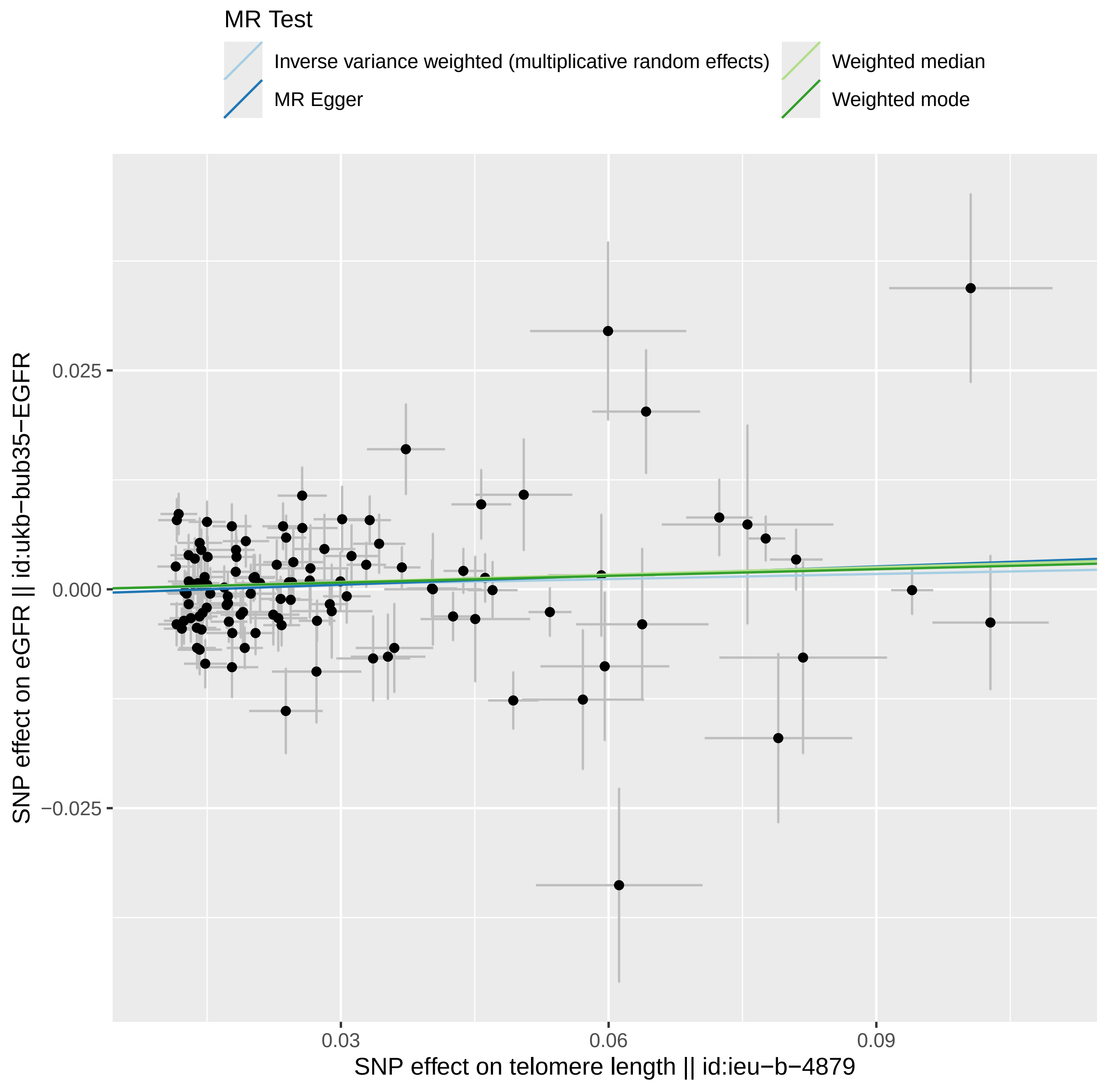

MR Test
Inverse variance weighted (multiplicative random effects)
MR Egger
Weighted median
Weighted mode
0.025
0.000
−0.025
0.03
0.06
0.09
SNP effect on eGFR || id:ukb−bub35−EGFR
SNP effect on telomere length || id:ieu−b−4879

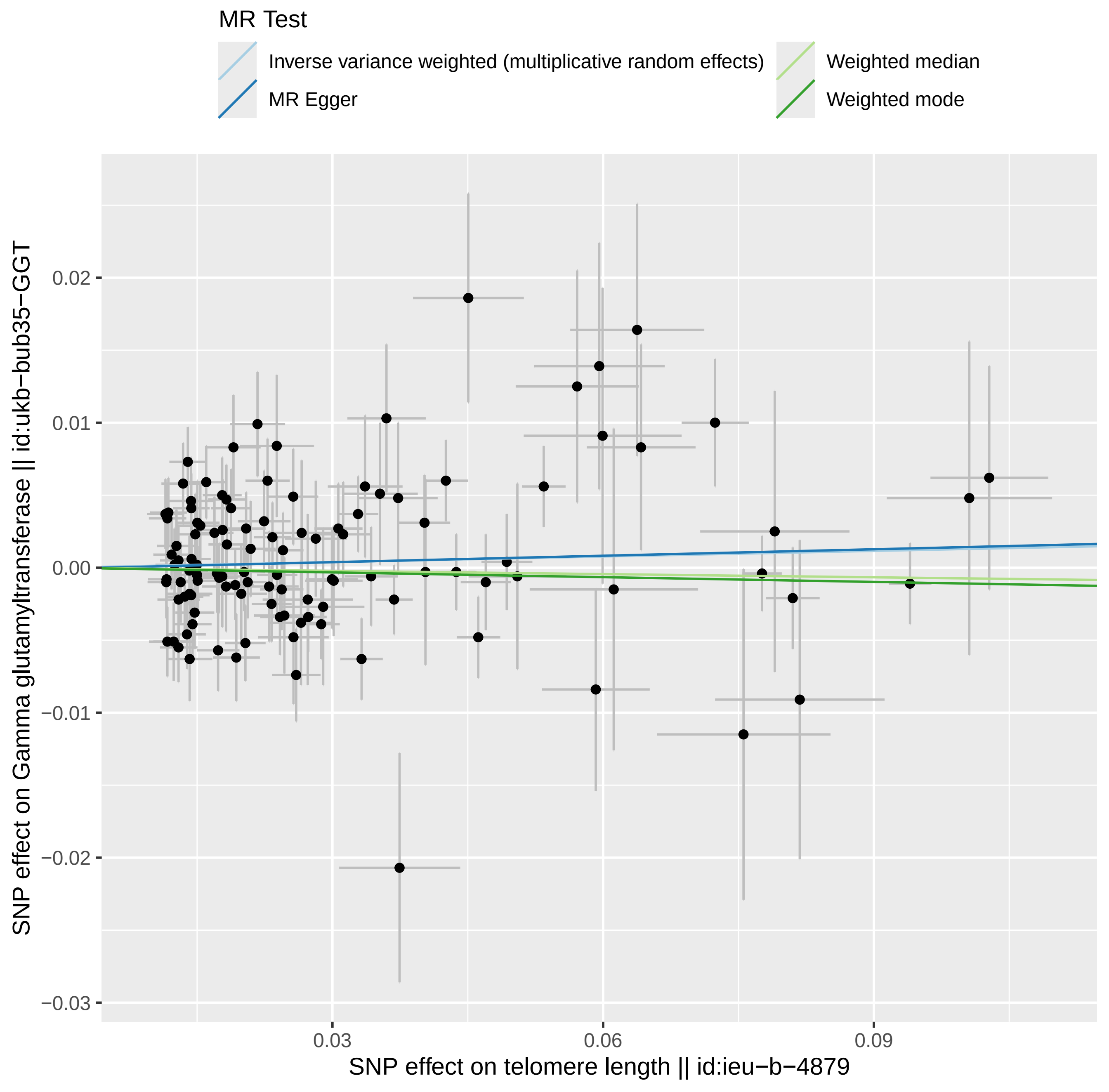

MR Test
Inverse variance weighted (multiplicative random effects)
MR Egger
Weighted median
Weighted mode
SNP effect on Gamma glutamyltransferase || id:ukb−bub35−GGT
0.02
0.01
0.00
−0.01
−0.02
−0.03
0.03
0.06
0.09
SNP effect on telomere length || id:ieu−b−4879

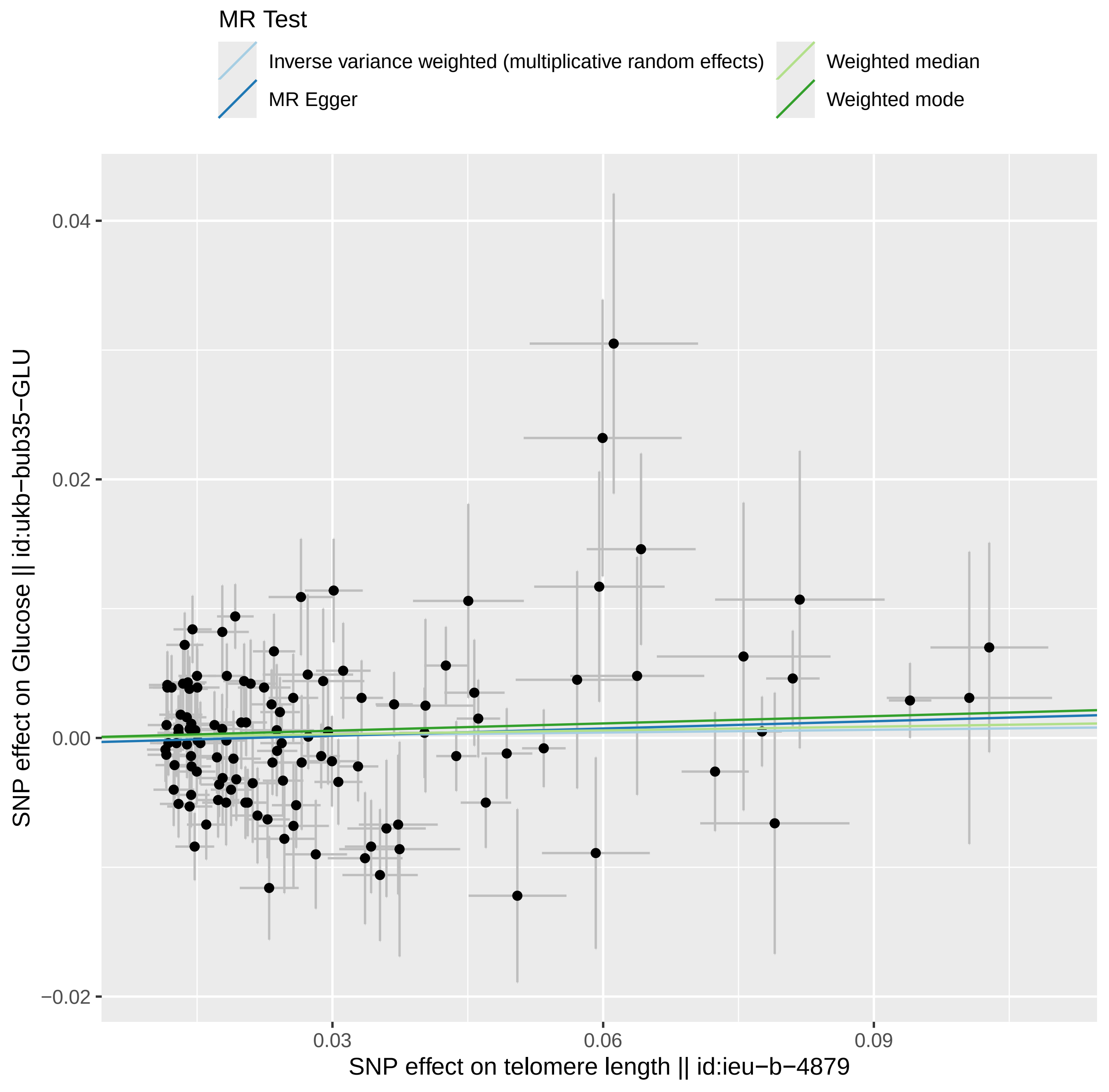

MR Test
Inverse variance weighted (multiplicative random effects)
MR Egger
Weighted median
Weighted mode
0.04
0.02
0.00
−0.02
SNP effect on Glucose || id:ukb−bub35−GLU
0.03
0.06
0.09
SNP effect on telomere length || id:ieu−b−4879

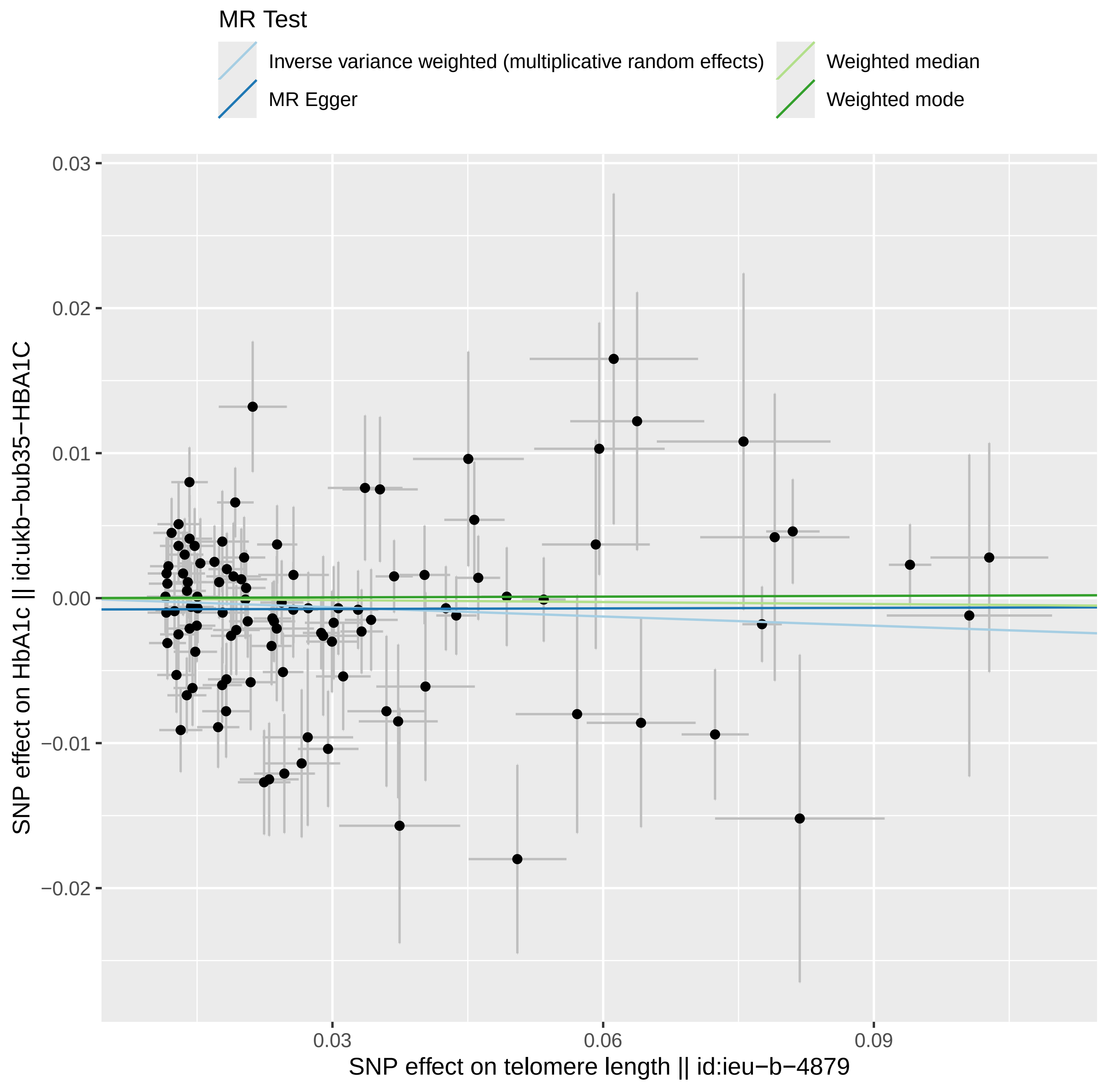

MR Test
Inverse variance weighted (multiplicative random effects)
MR Egger
Weighted median
Weighted mode
0.03
0.02
0.01
0.00
−0.01
−0.02
SNP effect on HbA1c || id:ukb−bub35−HBA1C
0.03
0.06
0.09
SNP effect on telomere length || id:ieu−b−4879

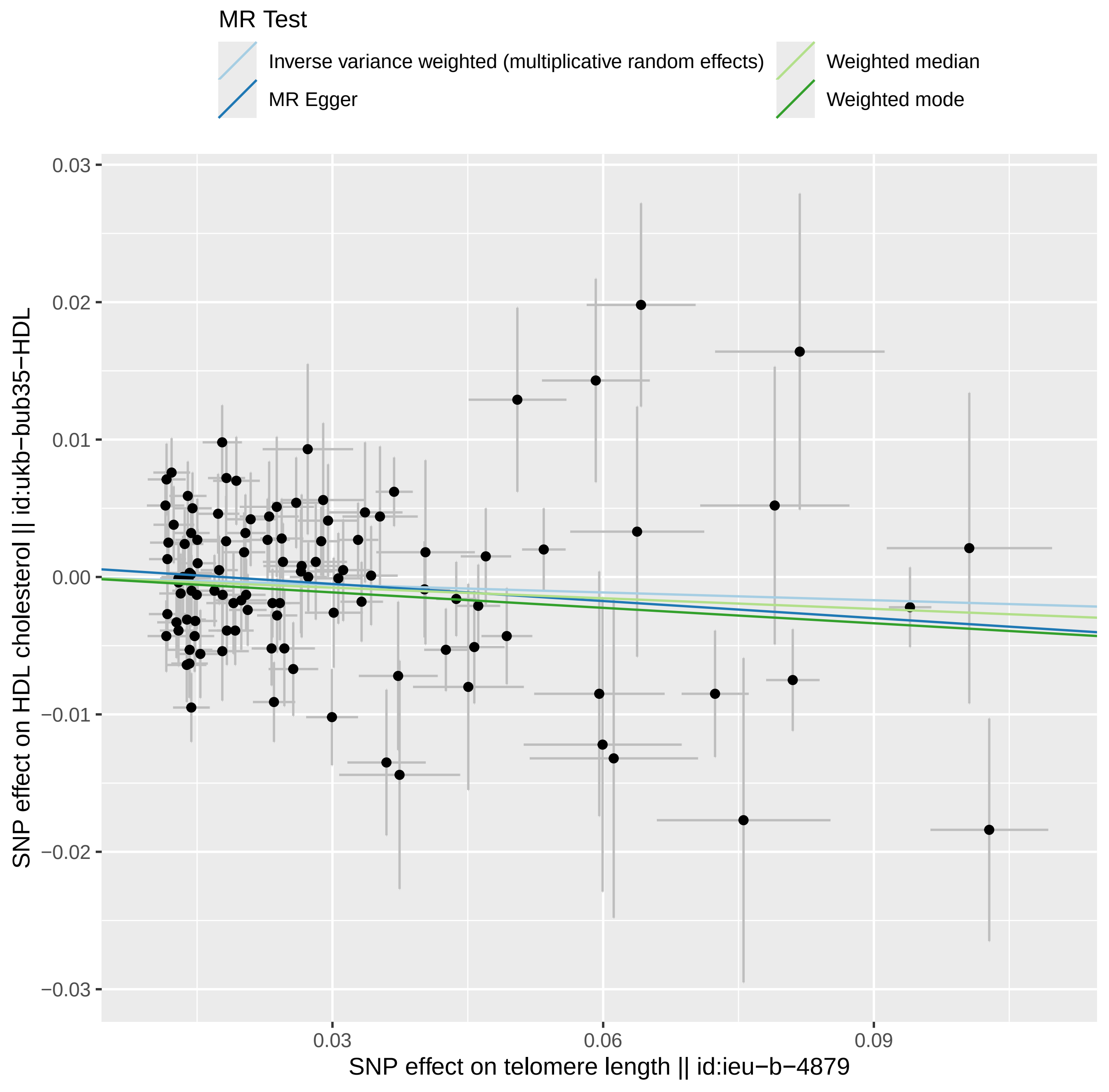

MR Test
Inverse variance weighted (multiplicative random effects)
MR Egger
Weighted median
Weighted mode
0.03
0.02
0.01
0.00
−0.01
−0.02
−0.03
0.03
0.06
0.09
SNP effect on telomere length || id:ieu−b−4879
SNP effect on HDL cholesterol || id:ukb−bub35−HDL

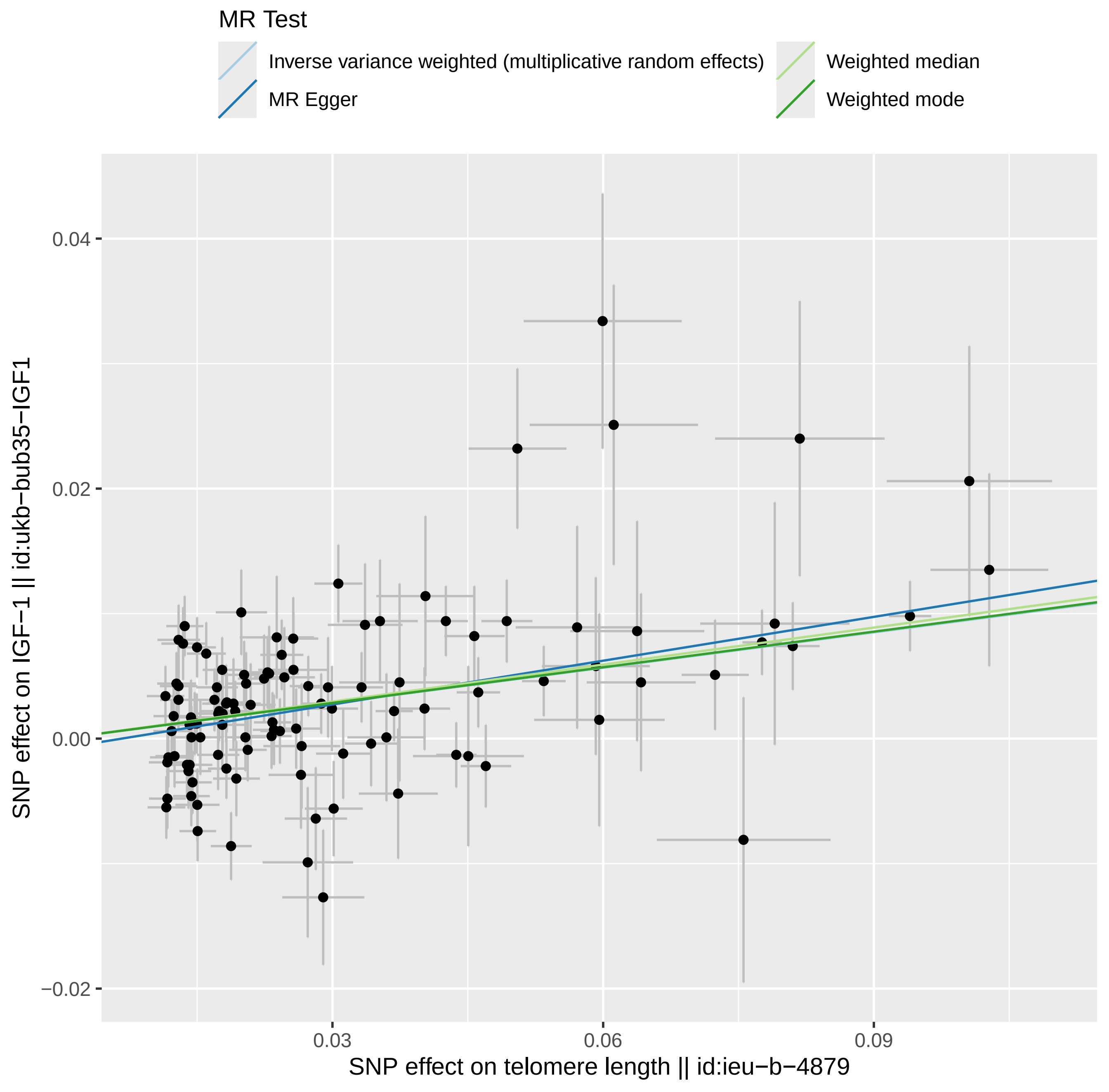
MR Test
Inverse variance weighted (multiplicative random effects)
MR Egger
Weighted median
Weighted mode
SNP effect on IGF−1 || id:ukb−bub35−IGF1
0.04
0.02
0.00
−0.02
0.03
0.06
0.09
SNP effect on telomere length || id:ieu−b−4879

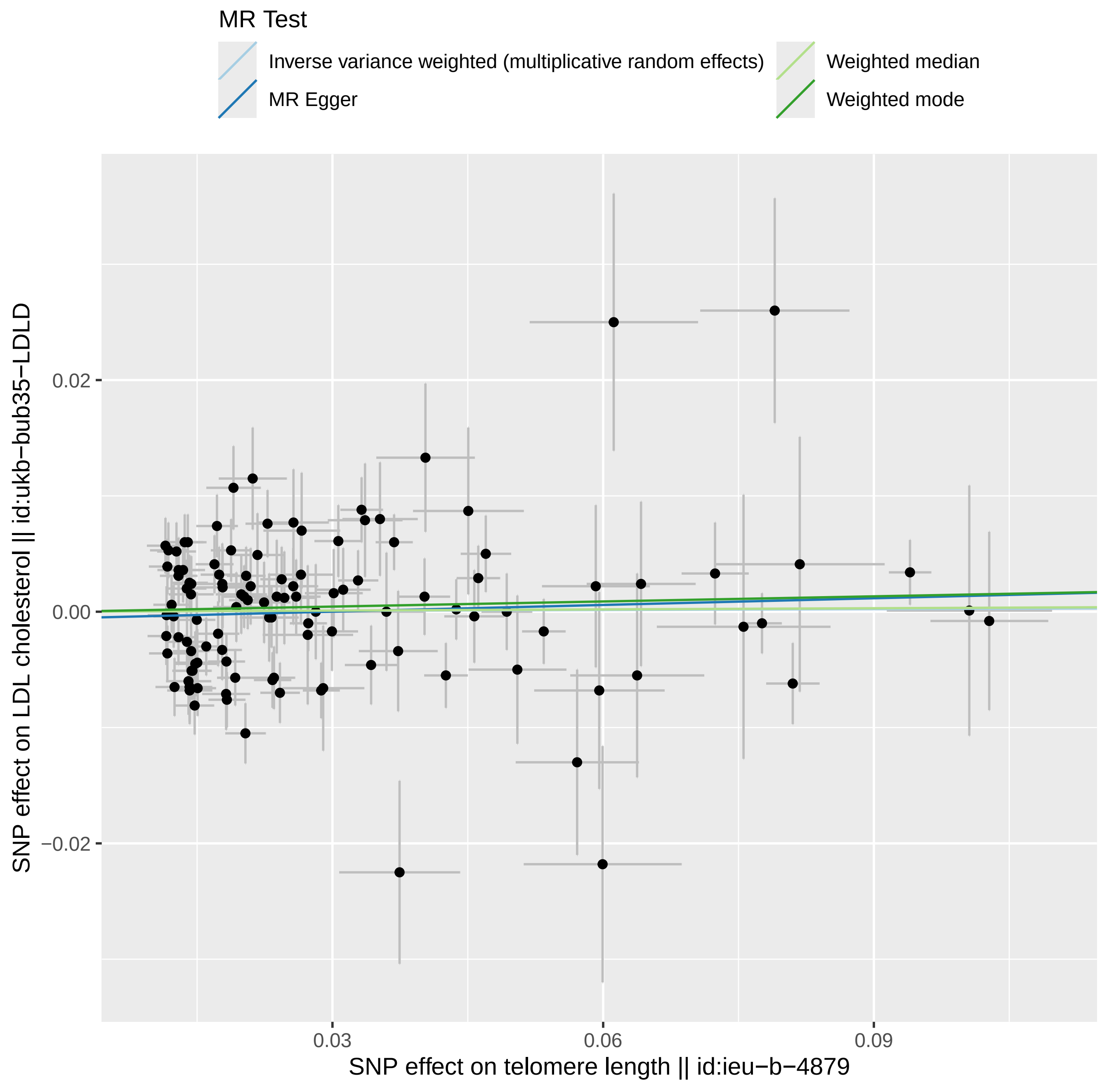
MR Test
Inverse variance weighted (multiplicative random effects)
MR Egger
Weighted median
Weighted mode
0.02
0.00
−0.02
0.03
0.06
0.09
SNP effect on LDL cholesterol || id:ukb−bub35−LDLD
SNP effect on telomere length || id:ieu−b−4879

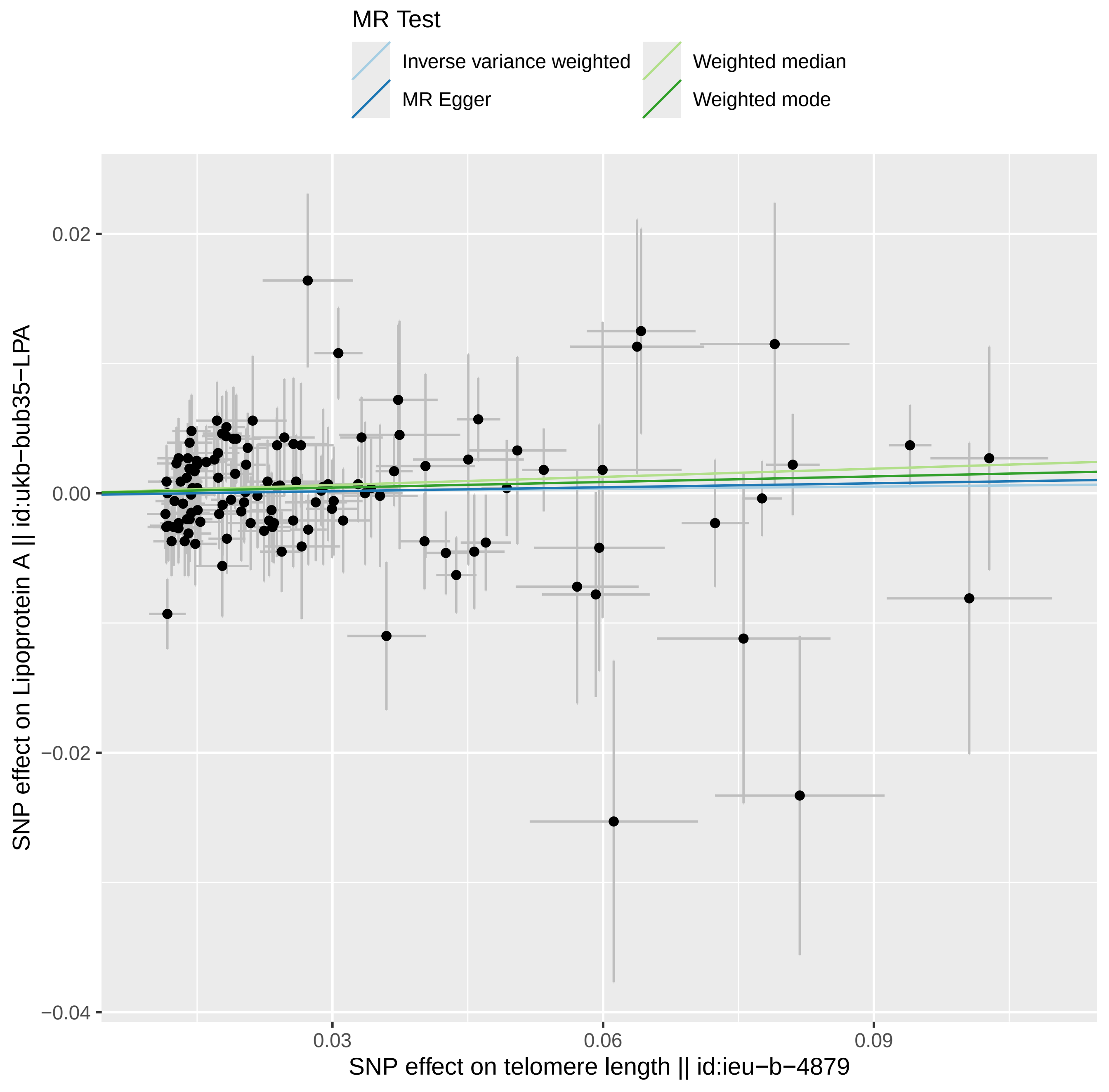

MR Test
Inverse variance weighted
MR Egger
Weighted median
Weighted mode
0.02
0.00
−0.02
−0.04
0.03
0.06
0.09
SNP effect on Lipoprotein A || id:ukb−bub35−LPA
SNP effect on telomere length || id:ieu−b−4879

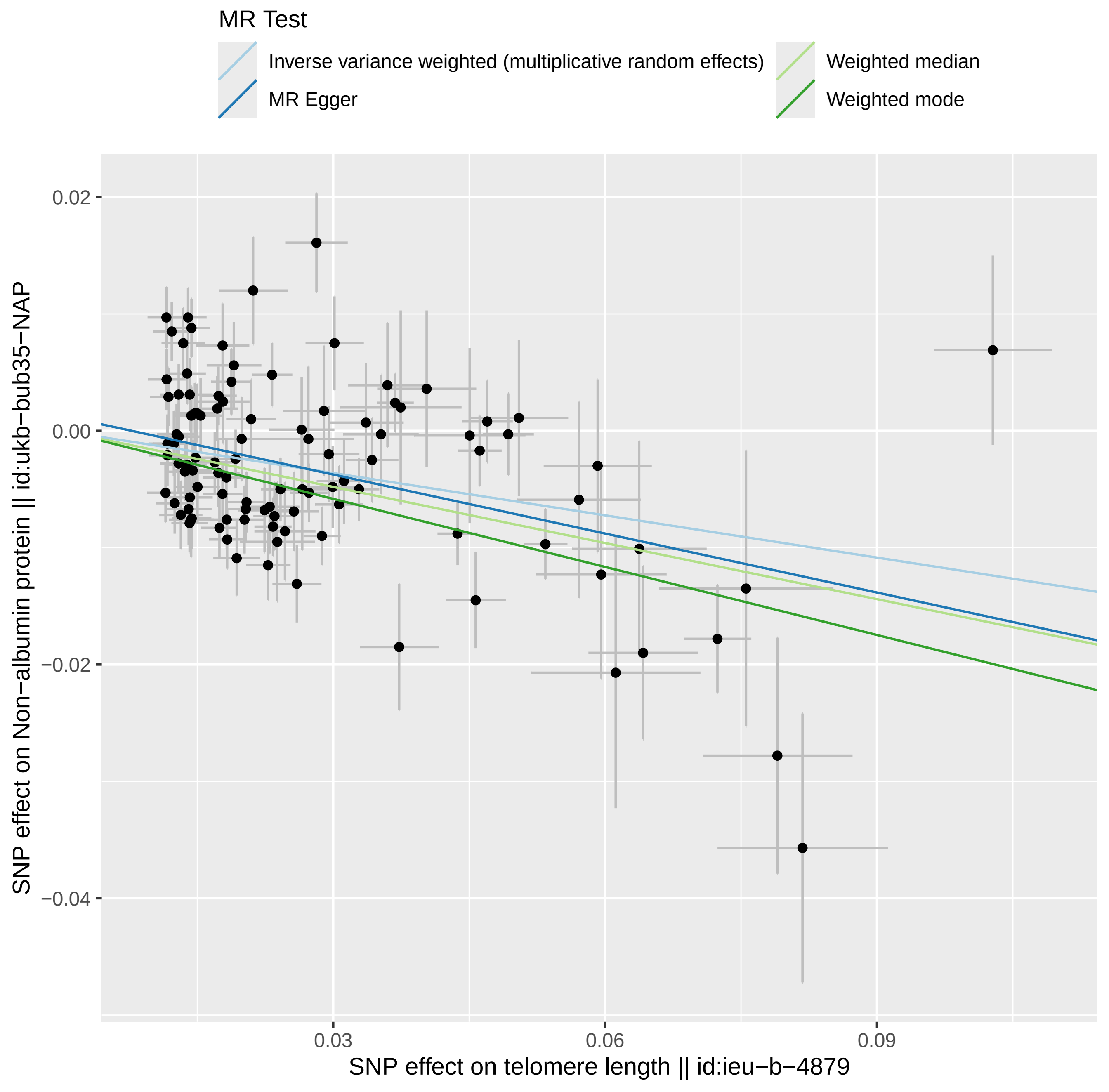

MR Test
Inverse variance weighted (multiplicative random effects)
MR Egger
Weighted median
Weighted mode
0.02
0.00
−0.02
−0.04
0.03
0.06
0.09
SNP effect on Non−albumin protein || id:ukb−bub35−NAP
SNP effect on telomere length || id:ieu−b−4879

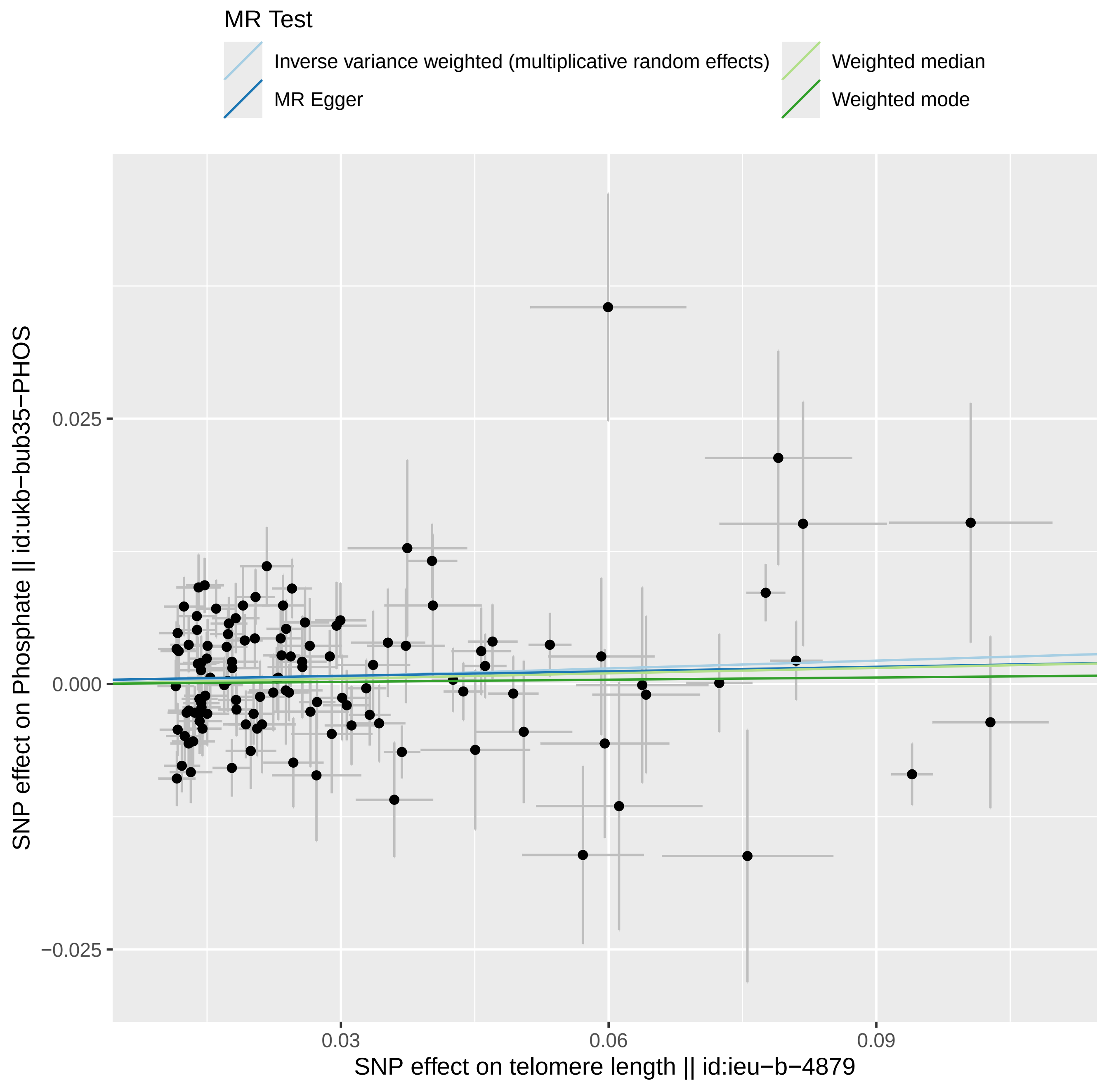

MR Test
Inverse variance weighted (multiplicative random effects)
MR Egger
Weighted median
Weighted mode
0.025
0.000
−0.025
0.03
0.06
0.09
SNP effect on telomere length || id:ieu−b−4879
SNP effect on Phosphate || id:ukb−bub35−PHOS

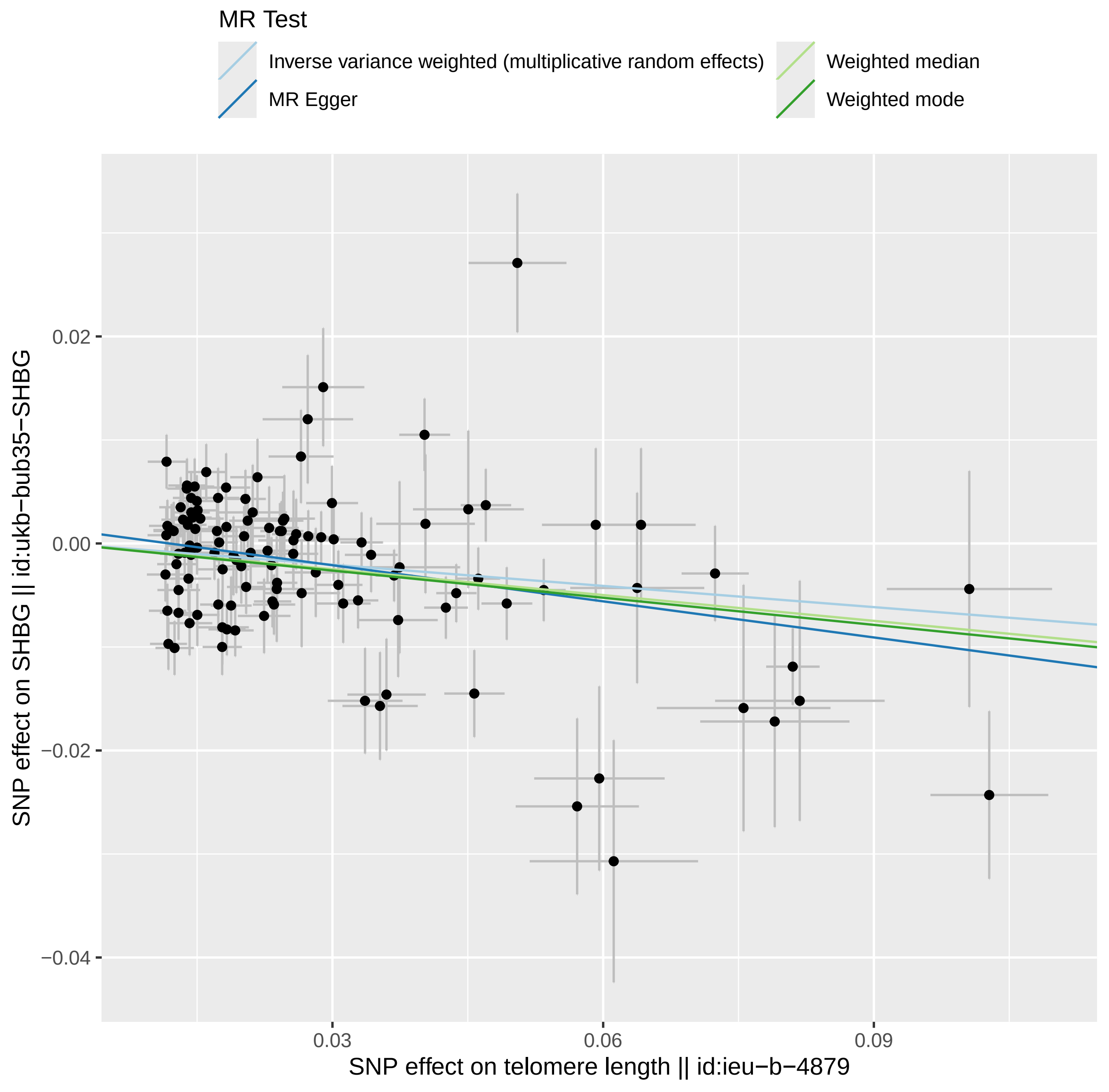

MR Test
Inverse variance weighted (multiplicative random effects)
MR Egger
Weighted median
Weighted mode
0.02
0.00
−0.02
−0.04
0.03
0.06
0.09
SNP effect on SHBG || id:ukb−bub35−SHBG
SNP effect on telomere length || id:ieu−b−4879

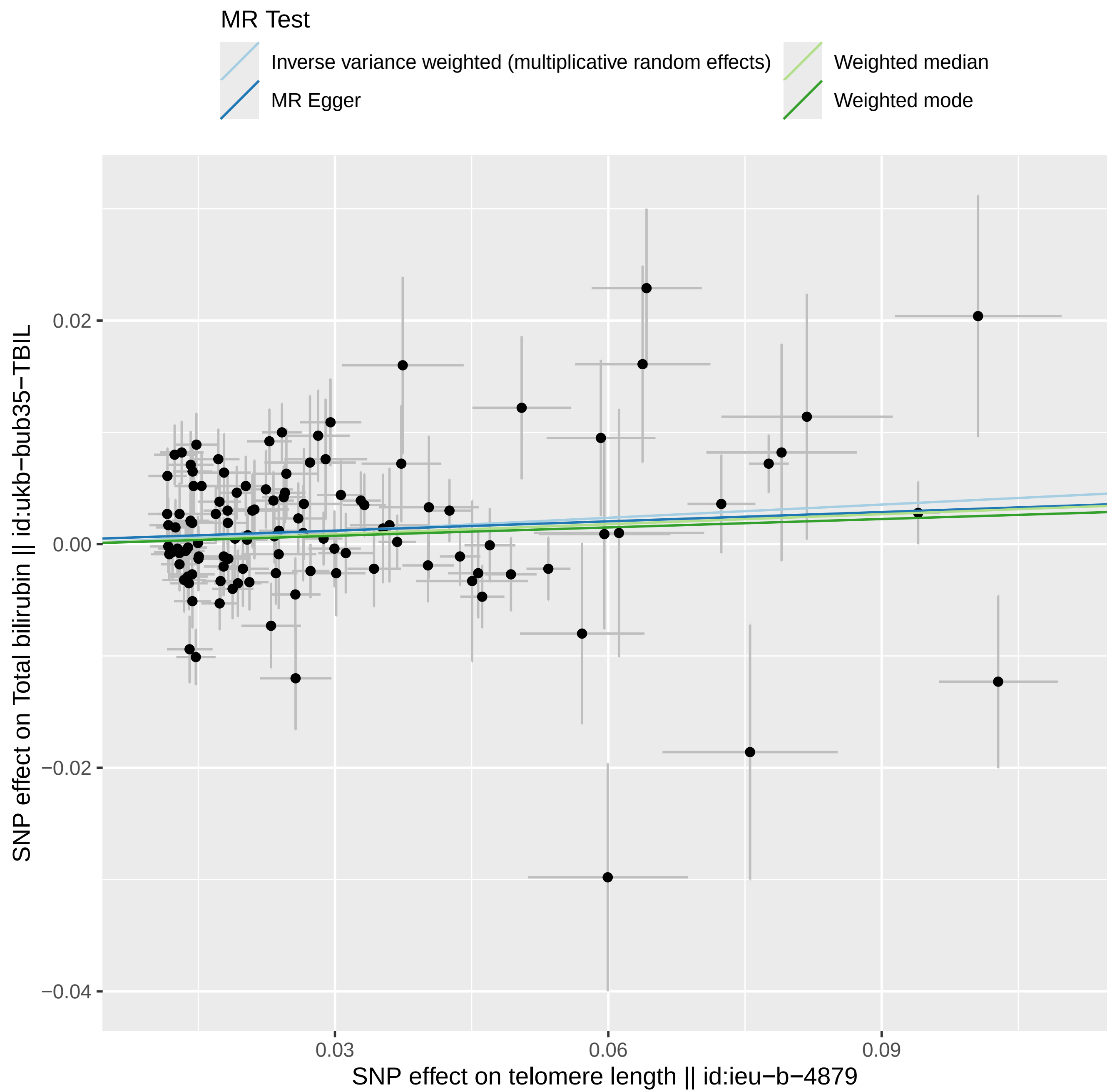

MR Test
Inverse variance weighted (multiplicative random effects)
MR Egger
Weighted median
Weighted mode
0.02
0.00
−0.02
−0.04
0.03
0.06
0.09
SNP effect on Total bilirubin || id:ukb−bub35−TBIL
SNP effect on telomere length || id:ieu−b−4879

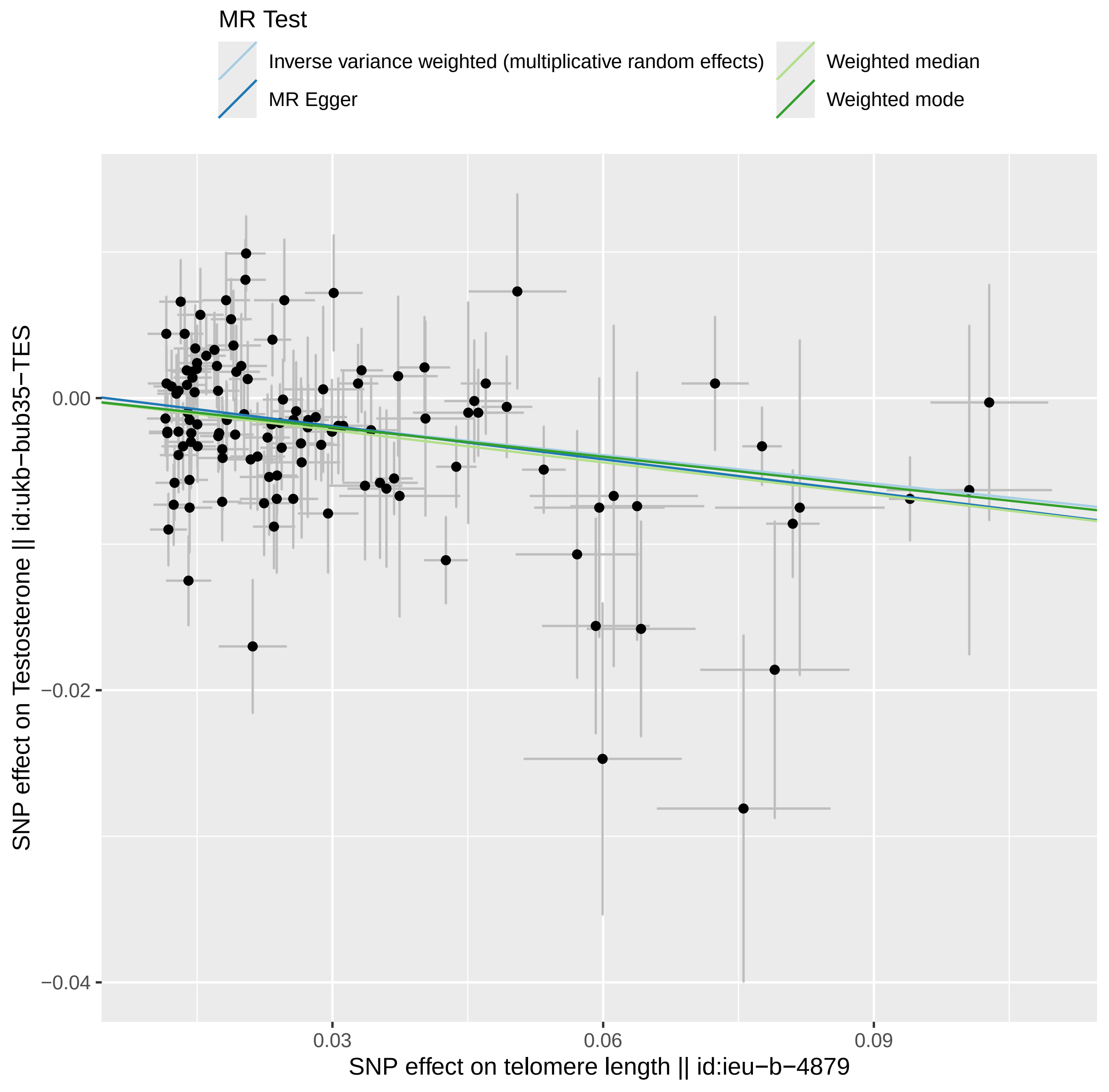

MR Test
Inverse variance weighted (multiplicative random effects)
MR Egger
Weighted median
Weighted mode
SNP effect on Testosterone || id:ukb−bub35−TES
0.00
−0.02
−0.04
0.03
0.06
0.09
SNP effect on telomere length || id:ieu−b−4879

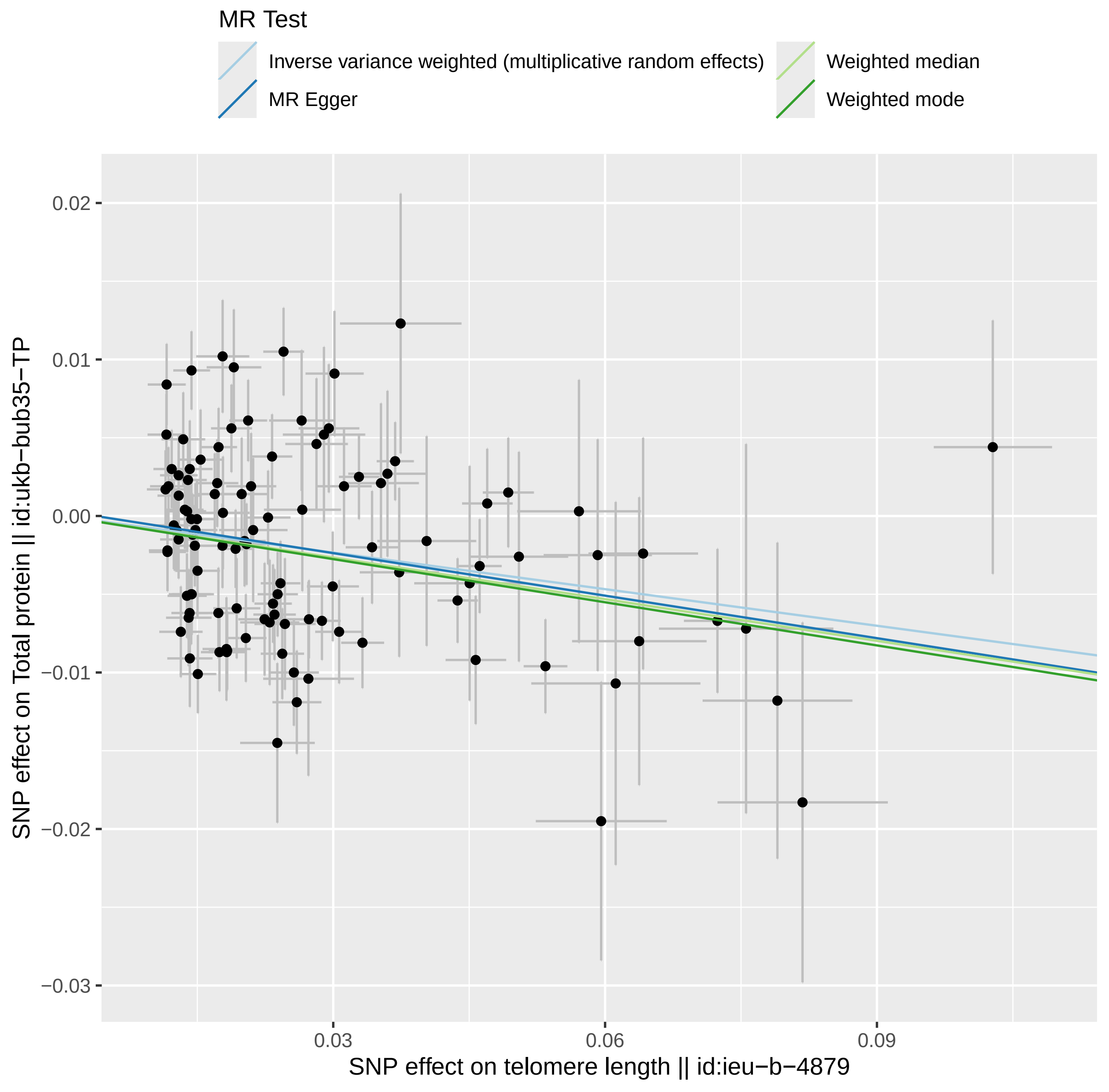
MR Test
Inverse variance weighted (multiplicative random effects)
MR Egger
Weighted median
Weighted mode
0.02
0.01
0.00
−0.01
−0.02
−0.03
SNP effect on Total protein || id:ukb−bub35−TP
0.03
0.06
0.09
SNP effect on telomere length || id:ieu−b−4879

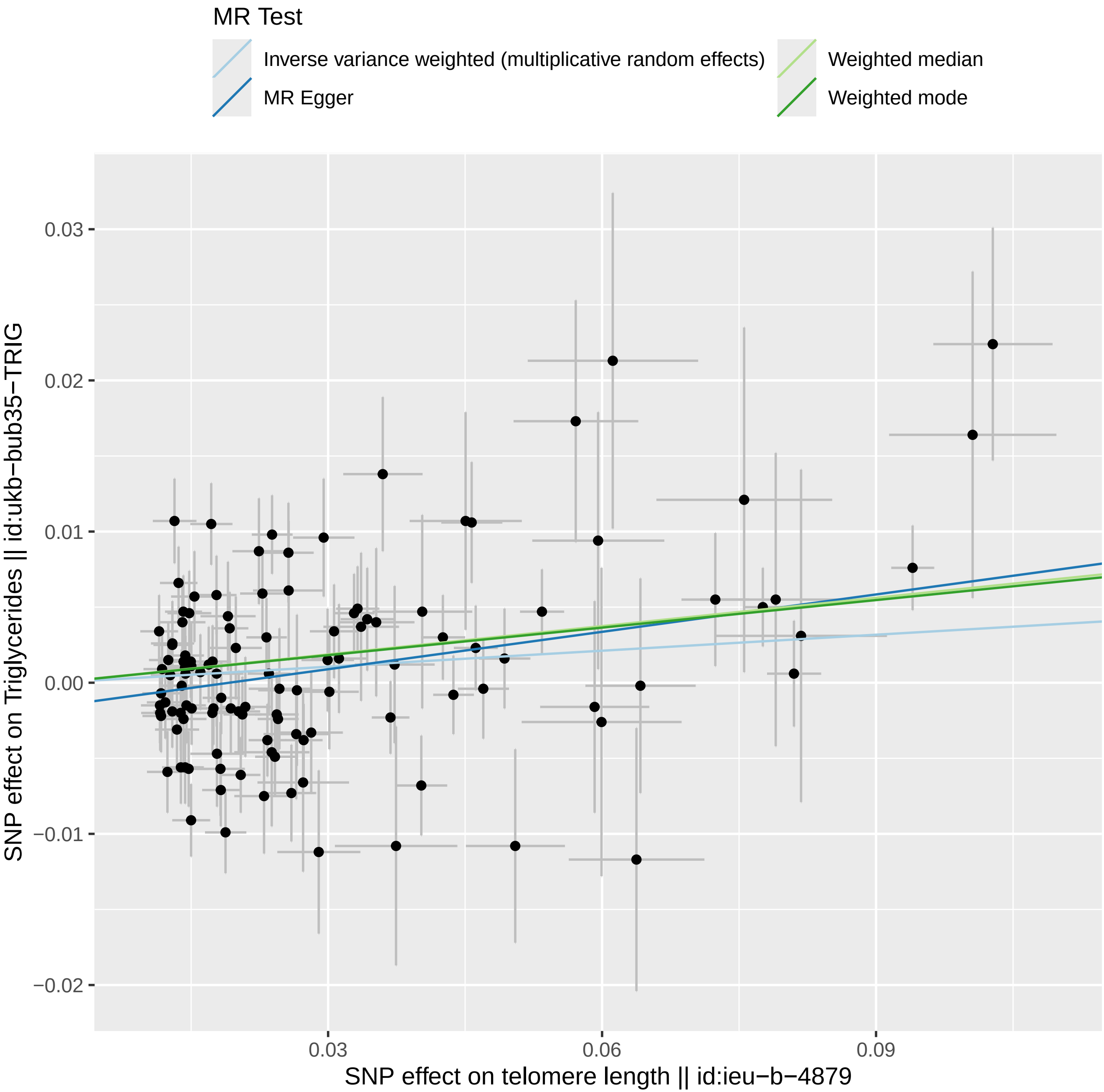
MR Test
Inverse variance weighted (multiplicative random effects)
MR Egger
Weighted median
Weighted mode
0.03
0.02
0.01
0.00
−0.01
−0.02
SNP effect on Triglycerides || id:ukb−bub35−TRIG
0.03
0.06
0.09
SNP effect on telomere length || id:ieu−b−4879

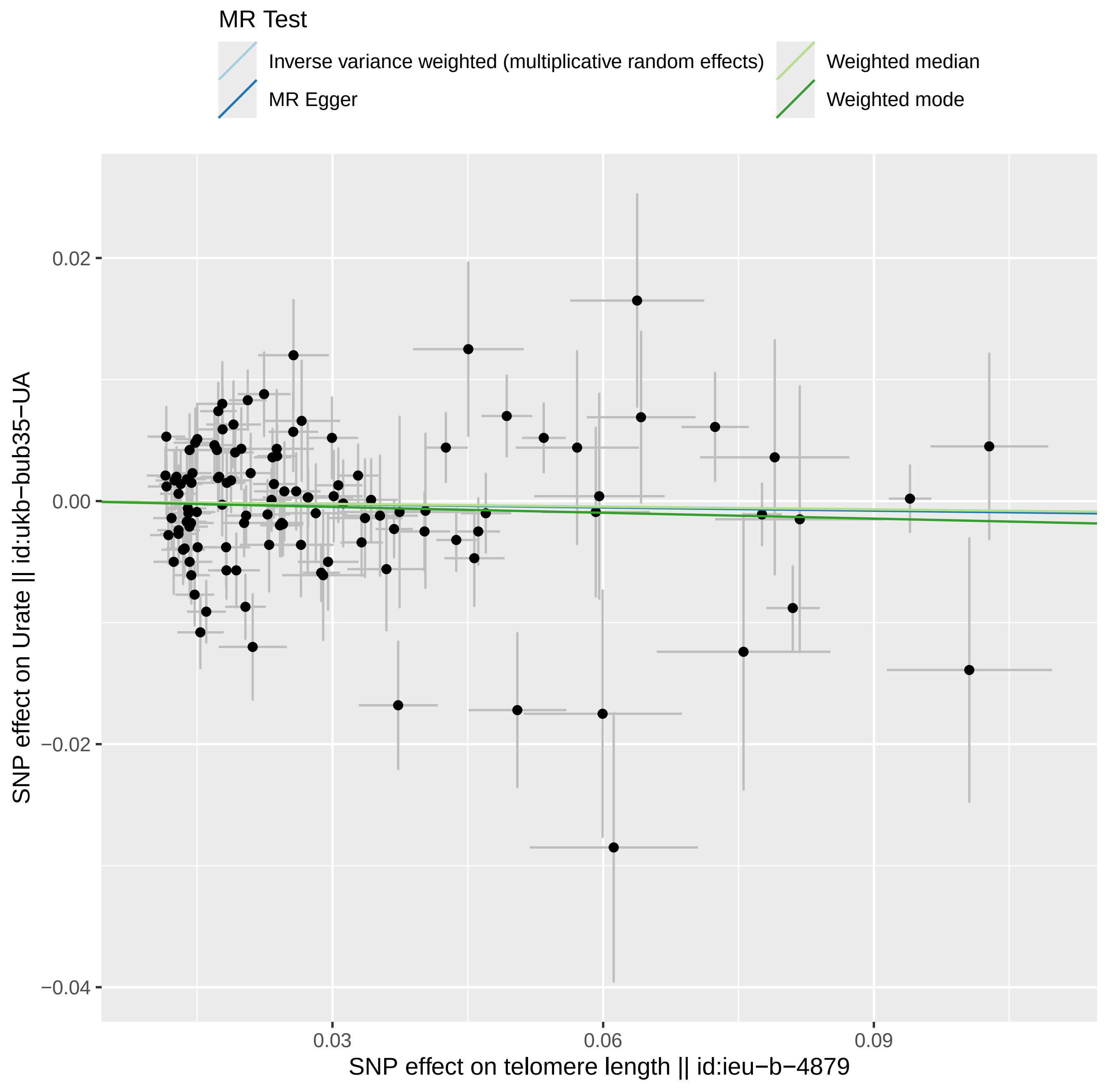

MR Test
Inverse variance weighted (multiplicative random effects)
MR Egger
Weighted median
Weighted mode
0.02
0.00
−0.02
−0.04
0.03
0.06
0.09
SNP effect on telomere length || id:ieu−b−4879
SNP effect on Urate || id:ukb−bub35−UA

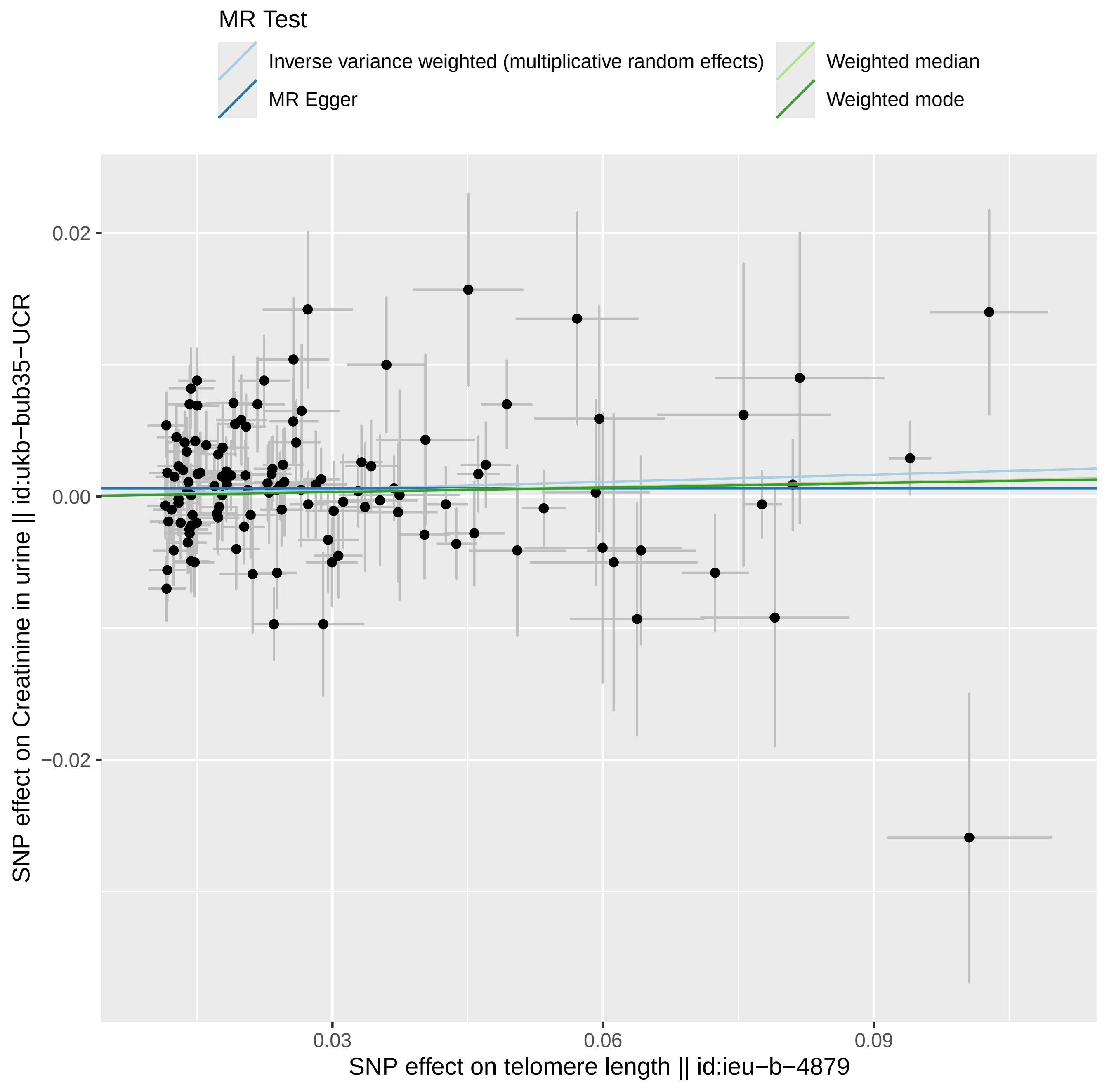

MR Test
Inverse variance weighted (multiplicative random effects)
MR Egger
Weighted median
Weighted mode
0.02
0.00
−0.02
0.03
0.06
0.09
SNP effect on Creatinine in urine || id:ukb−bub35−UCR
SNP effect on telomere length || id:ieu−b−4879

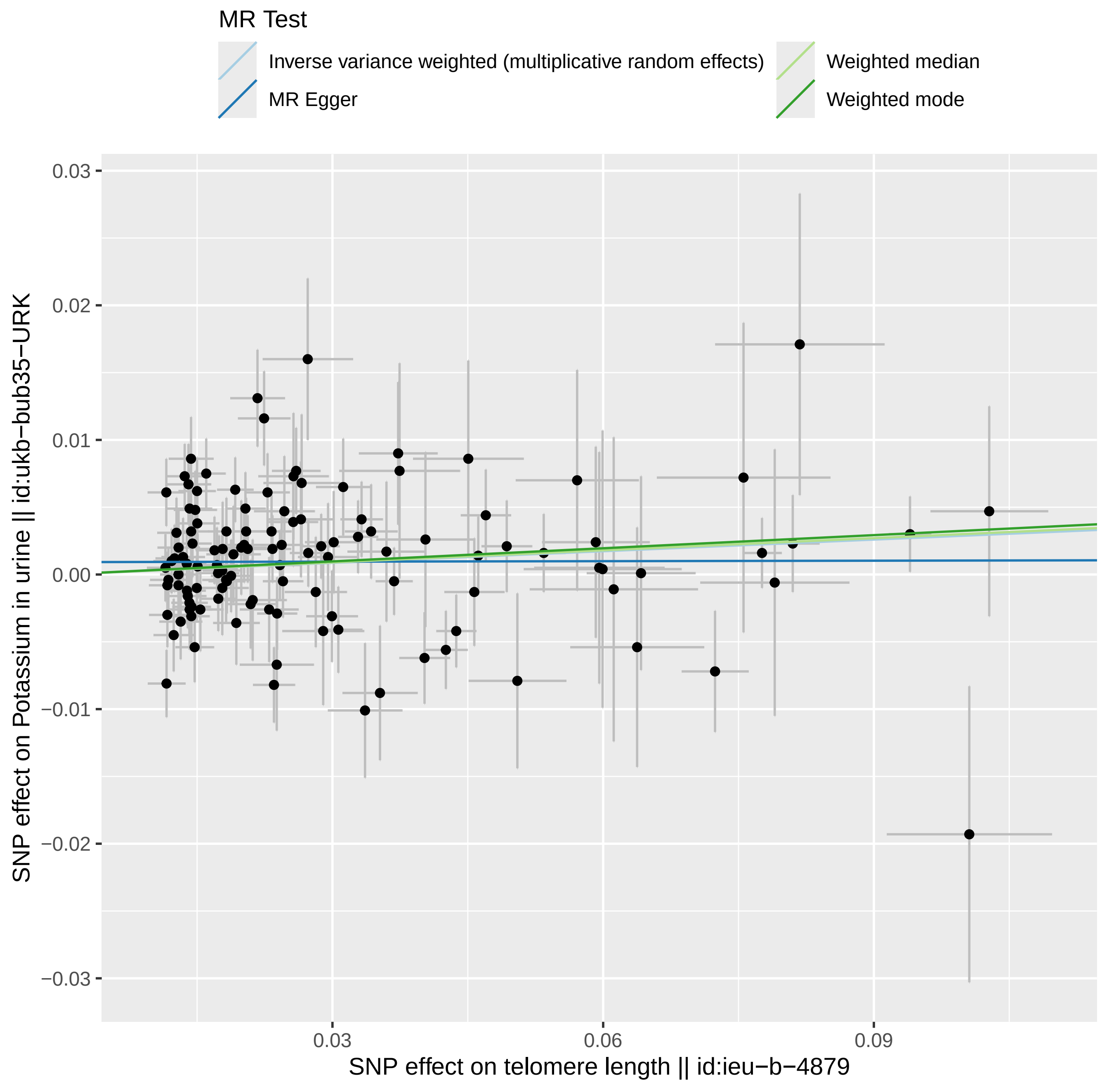

MR Test
Inverse variance weighted (multiplicative random effects)
MR Egger
Weighted median
Weighted mode
0.03
0.02
0.01
0.00
−0.01
−0.02
−0.03
0.03
0.06
0.09
SNP effect on Potassium in urine || id:ukb−bub35−URK
SNP effect on telomere length || id:ieu−b−4879

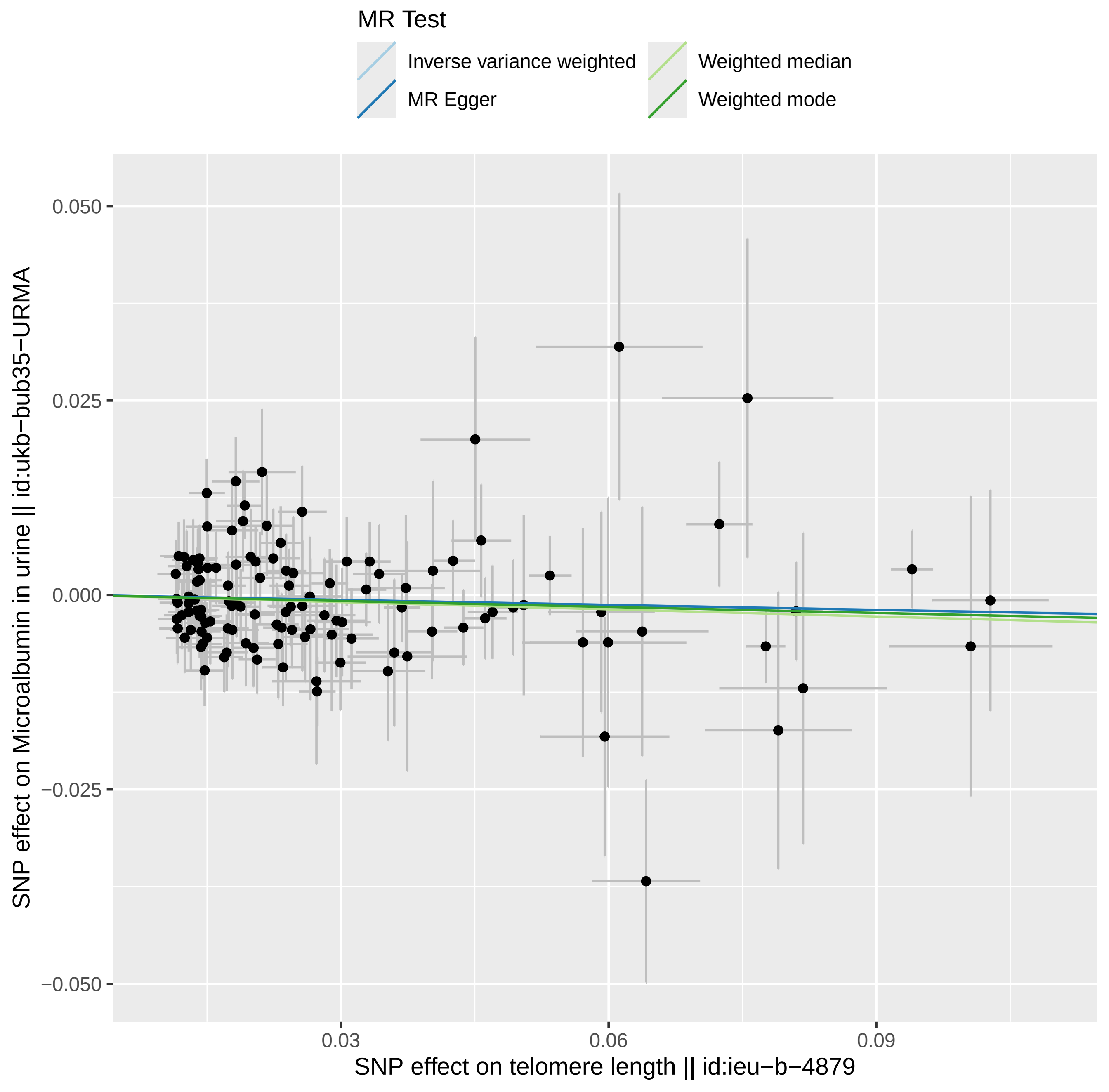

MR Test
Inverse variance weighted
MR Egger
Weighted median
Weighted mode
0.050
0.025
0.000
−0.025
−0.050
SNP effect on Microalbumin in urine || id:ukb−bub35−URMA
0.03
0.06
0.09
SNP effect on telomere length || id:ieu−b−4879

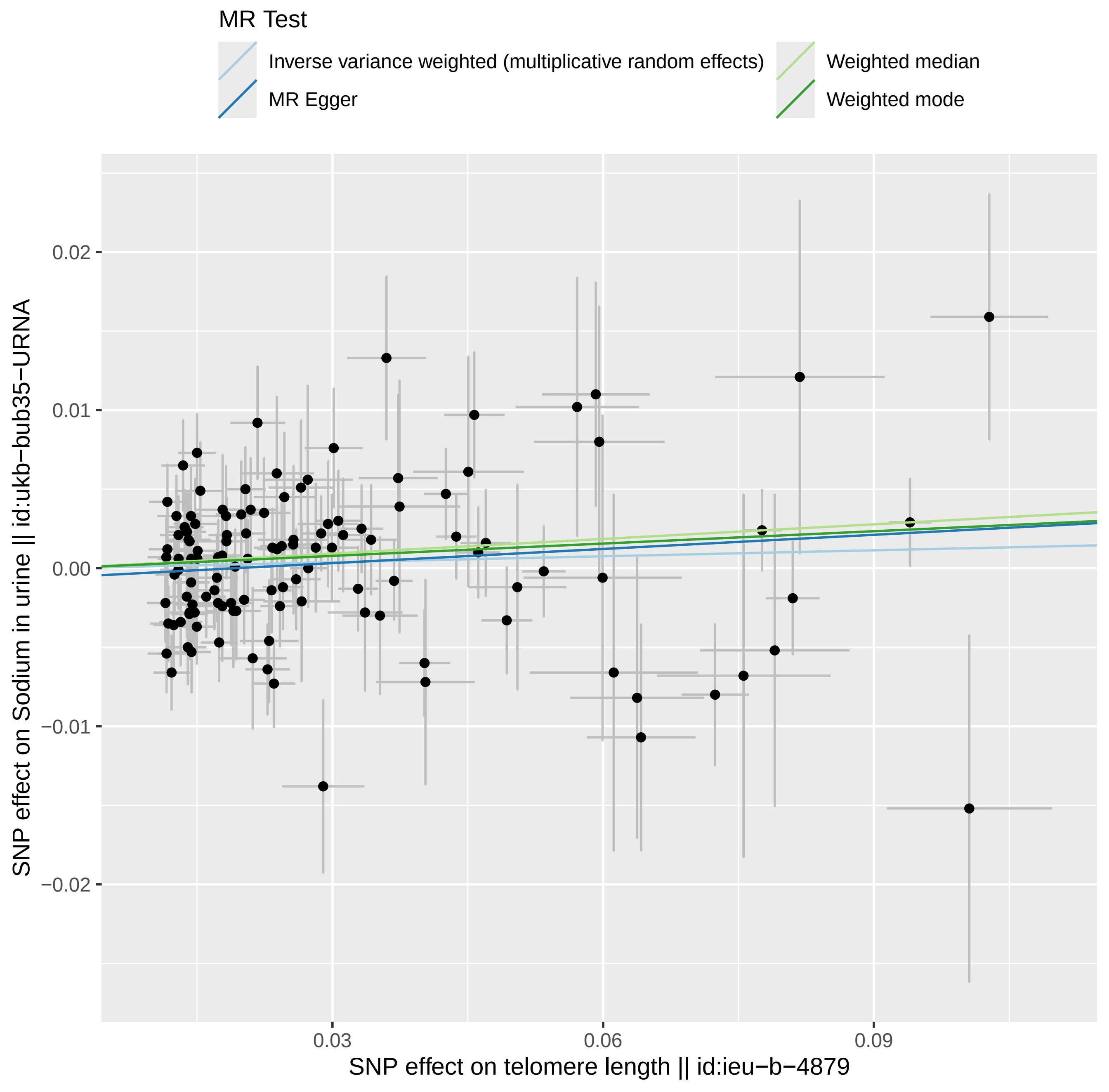

MR Test
Inverse variance weighted (multiplicative random effects)
MR Egger
Weighted median
Weighted mode
SNP effect on Sodium in urine || id:ukb−bub35−URNA
0.02
0.01
0.00
−0.01
−0.02
0.03
0.06
0.09
SNP effect on telomere length || id:ieu−b−4879

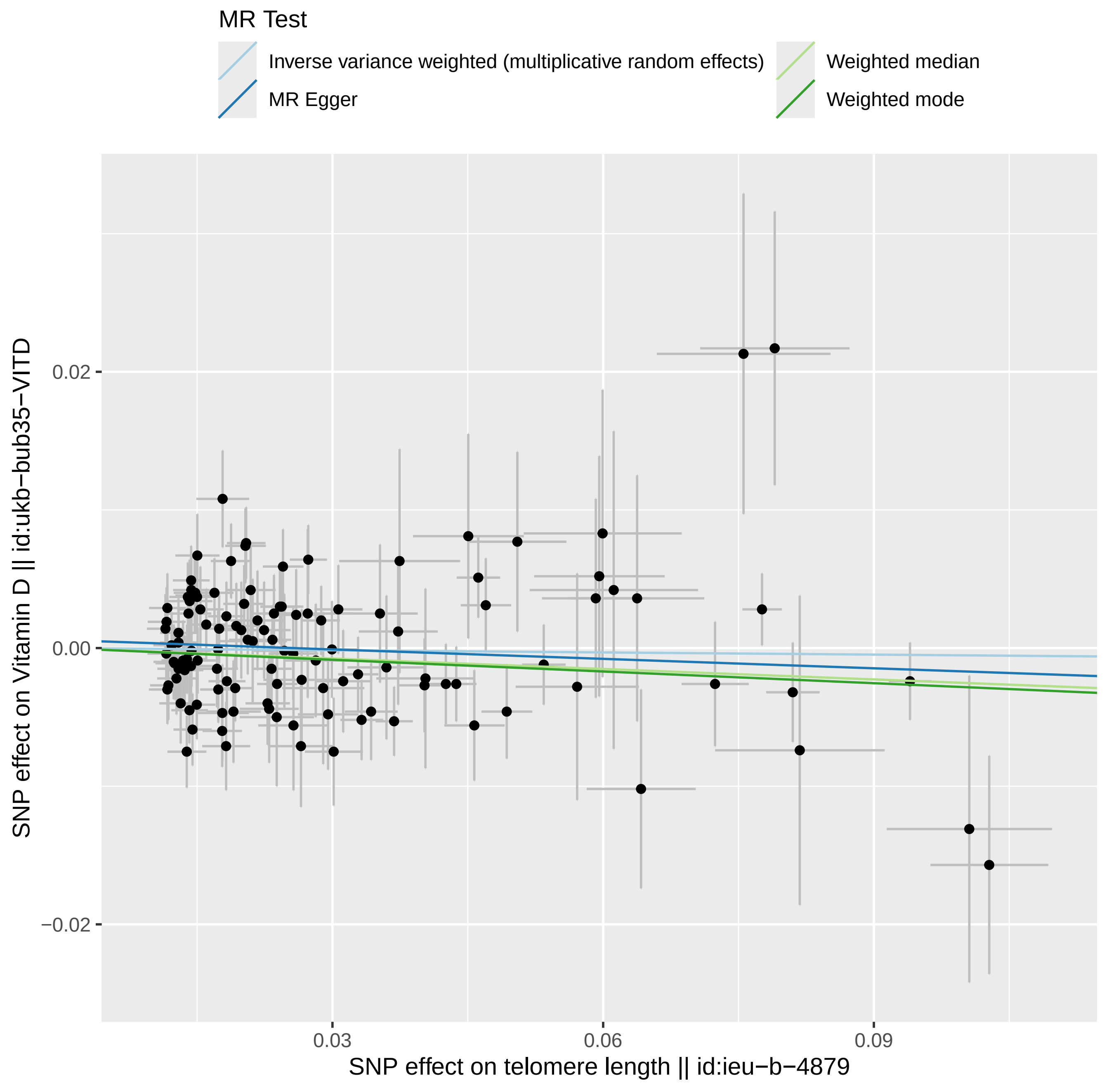

MR Test
Inverse variance weighted (multiplicative random effects)
MR Egger
Weighted median
Weighted mode
0.02
0.00
−0.02
0.03
0.06
0.09
SNP effect on Vitamin D || id:ukb−bub35−VITD
SNP effect on telomere length || id:ieu−b−4879

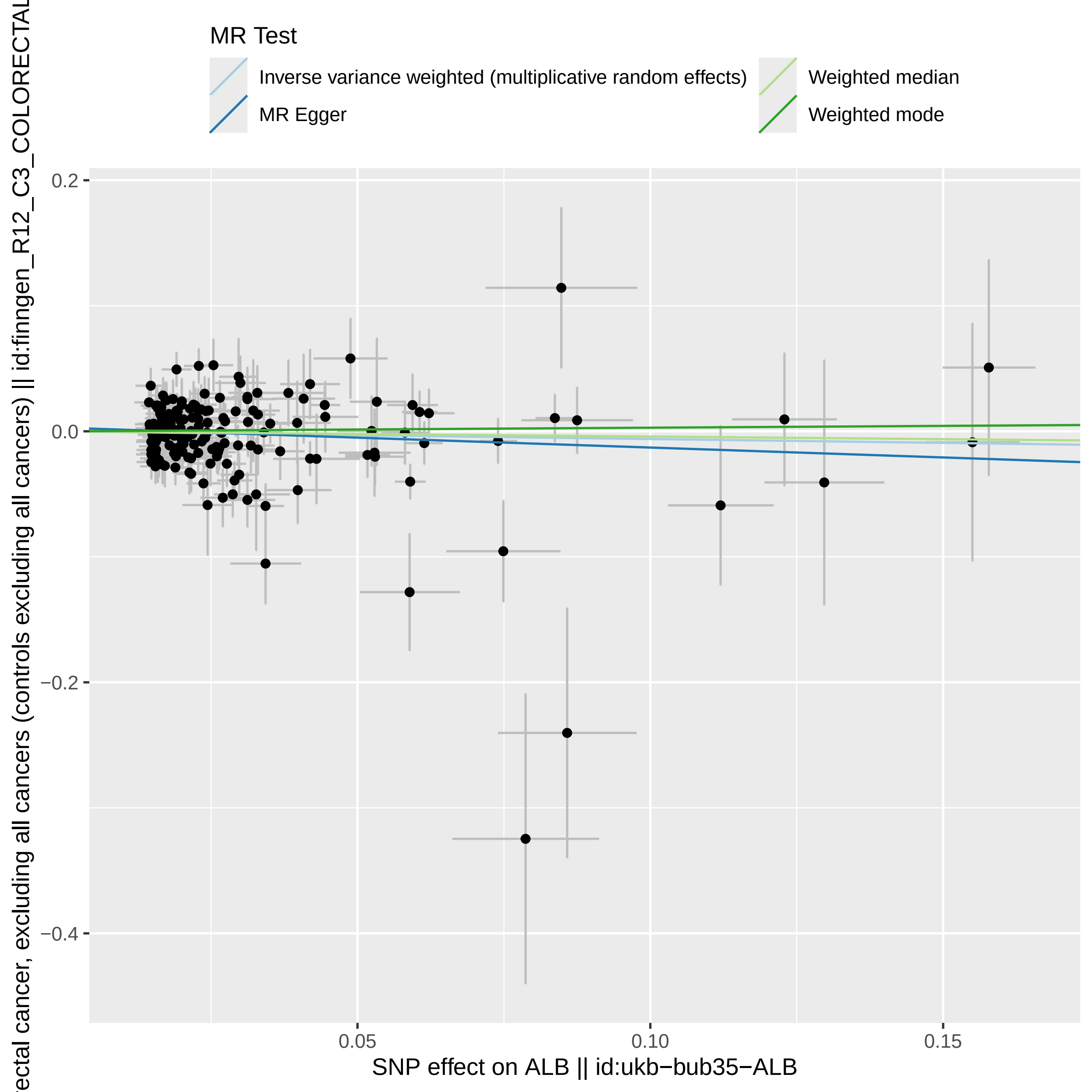

MR Test
Inverse variance weighted (multiplicative random effects)
MR Egger
Weighted median
Weighted mode
0.2
0.0
−0.2
−0.4
0.05
0.10
0.15
SNP effect on ALB || id:ukb−bub35−ALB
ectal cancer, excluding all cancers (controls excluding all cancers) || id:finngen_R12_C3_COLORECTAL

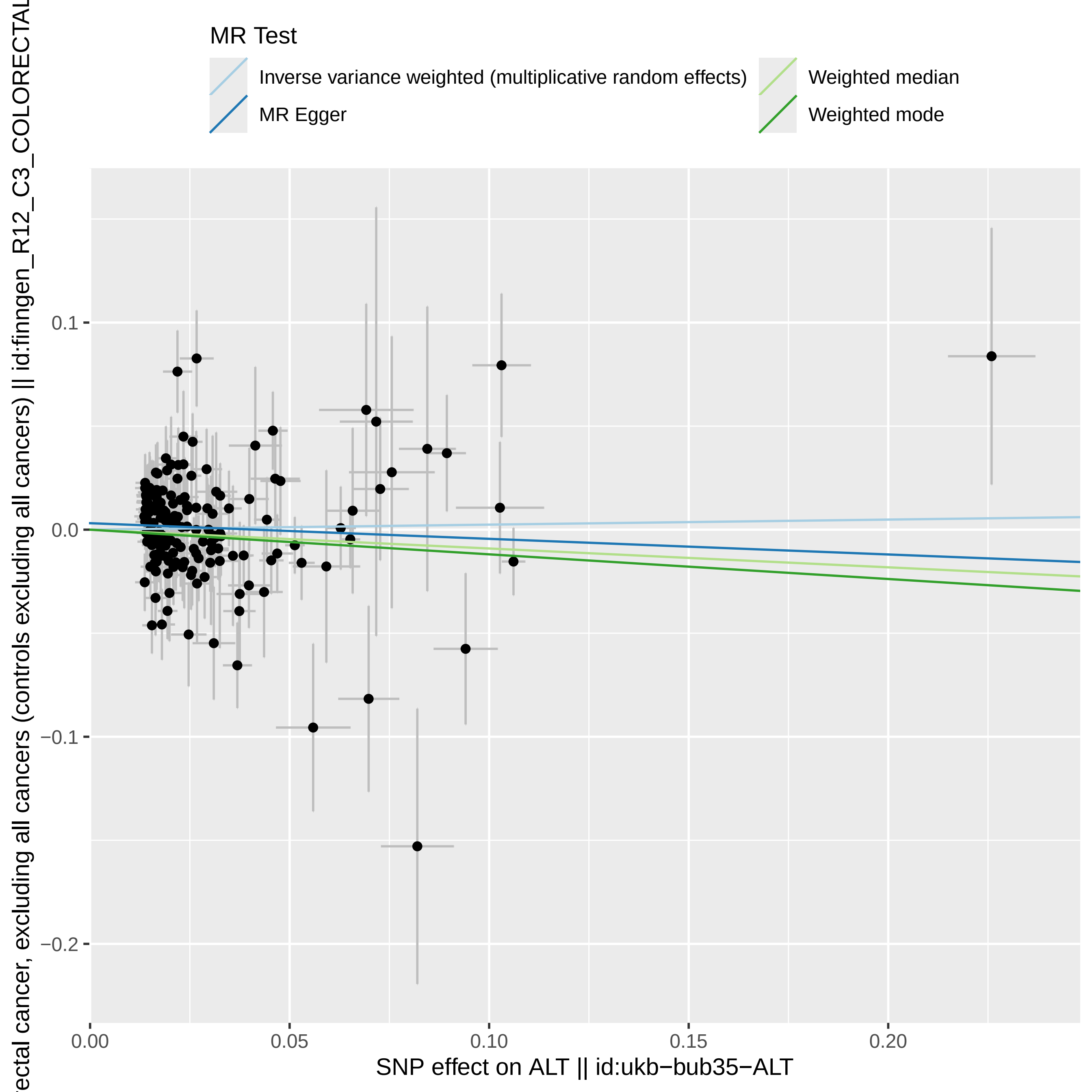

MR Test
Inverse variance weighted (multiplicative random effects)
MR Egger
Weighted median
Weighted mode
0.1
0.0
−0.1
−0.2
0.00
0.05
0.10
0.15
0.20
SNP effect on ALT || id:ukb−bub35−ALT
ectal cancer, excluding all cancers (controls excluding all cancers) || id:finngen_R12_C3_COLORECTAL

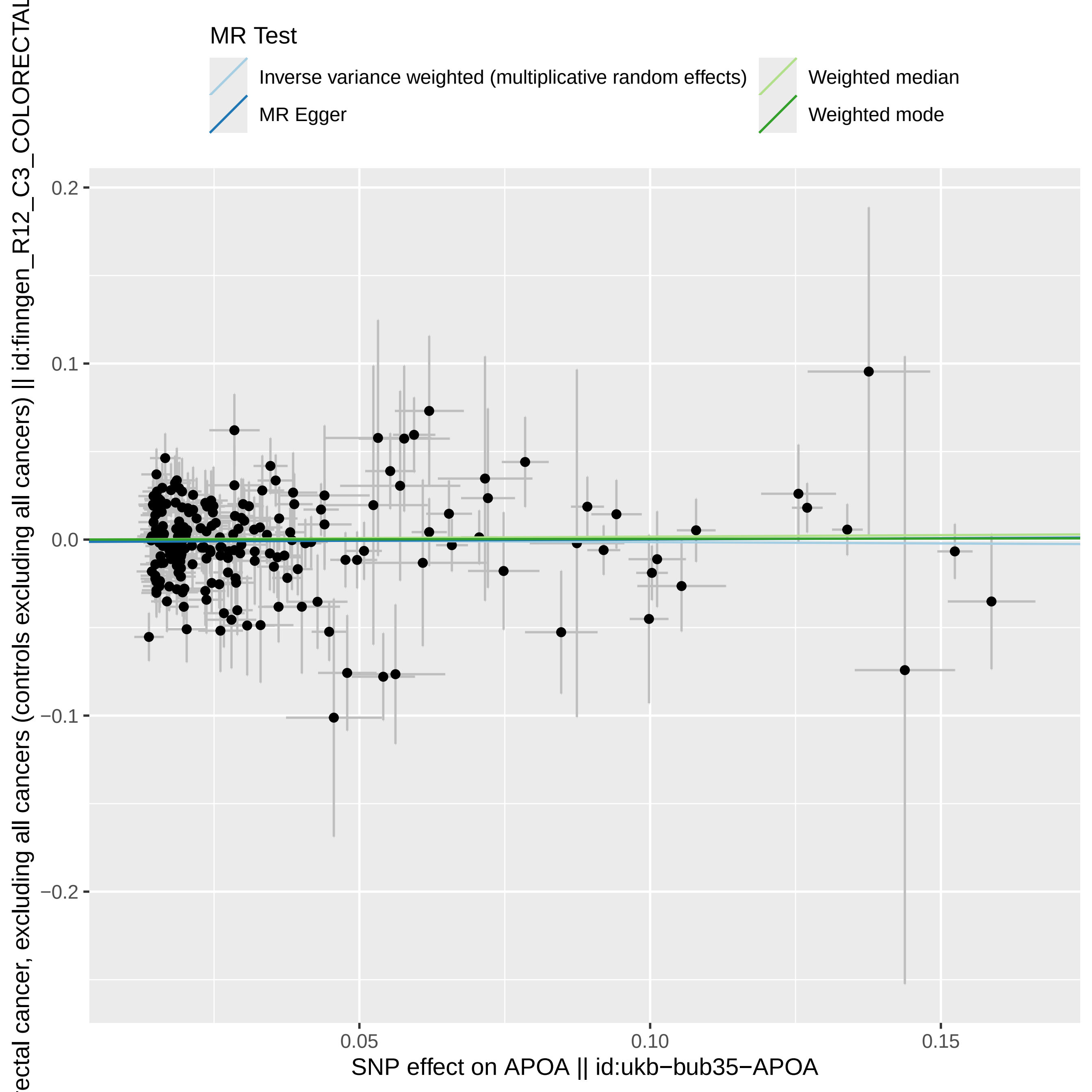

MR Test
Inverse variance weighted (multiplicative random effects)
MR Egger
Weighted median
Weighted mode
0.2
0.1
0.0
−0.1
−0.2
0.05
0.10
0.15
SNP effect on APOA || id:ukb−bub35−APOA
ectal cancer, excluding all cancers (controls excluding all cancers) || id:finngen_R12_C3_COLORECTAL

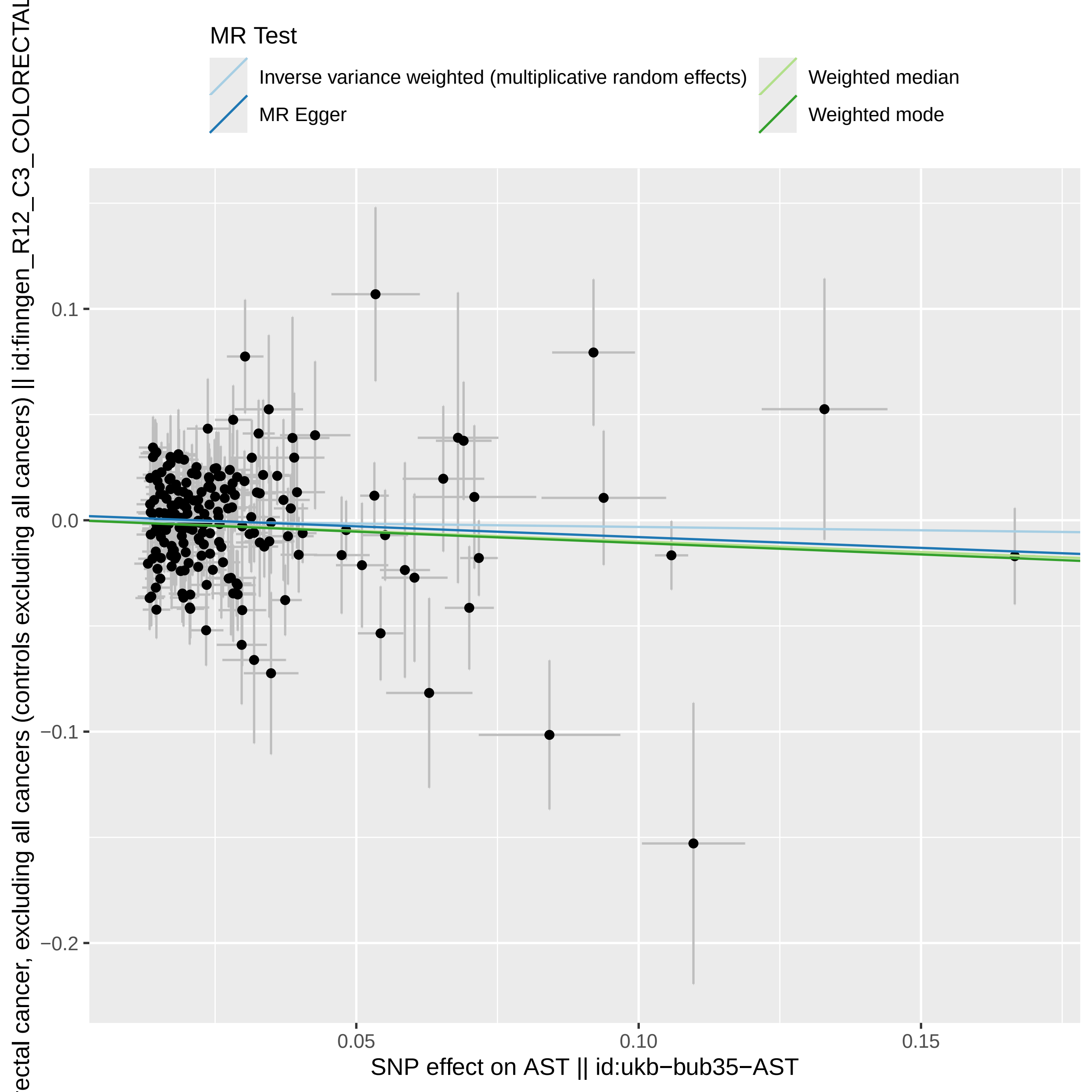

MR Test
Inverse variance weighted (multiplicative random effects)
Weighted median
MR Egger
Weighted mode
0.1
0.0
−0.1
−0.2
0.05
0.10
0.15
SNP effect on AST || id:ukb−bub35−AST
ectal cancer, excluding all cancers (controls excluding all cancers) || id:finngen_R12_C3_COLORECTAL

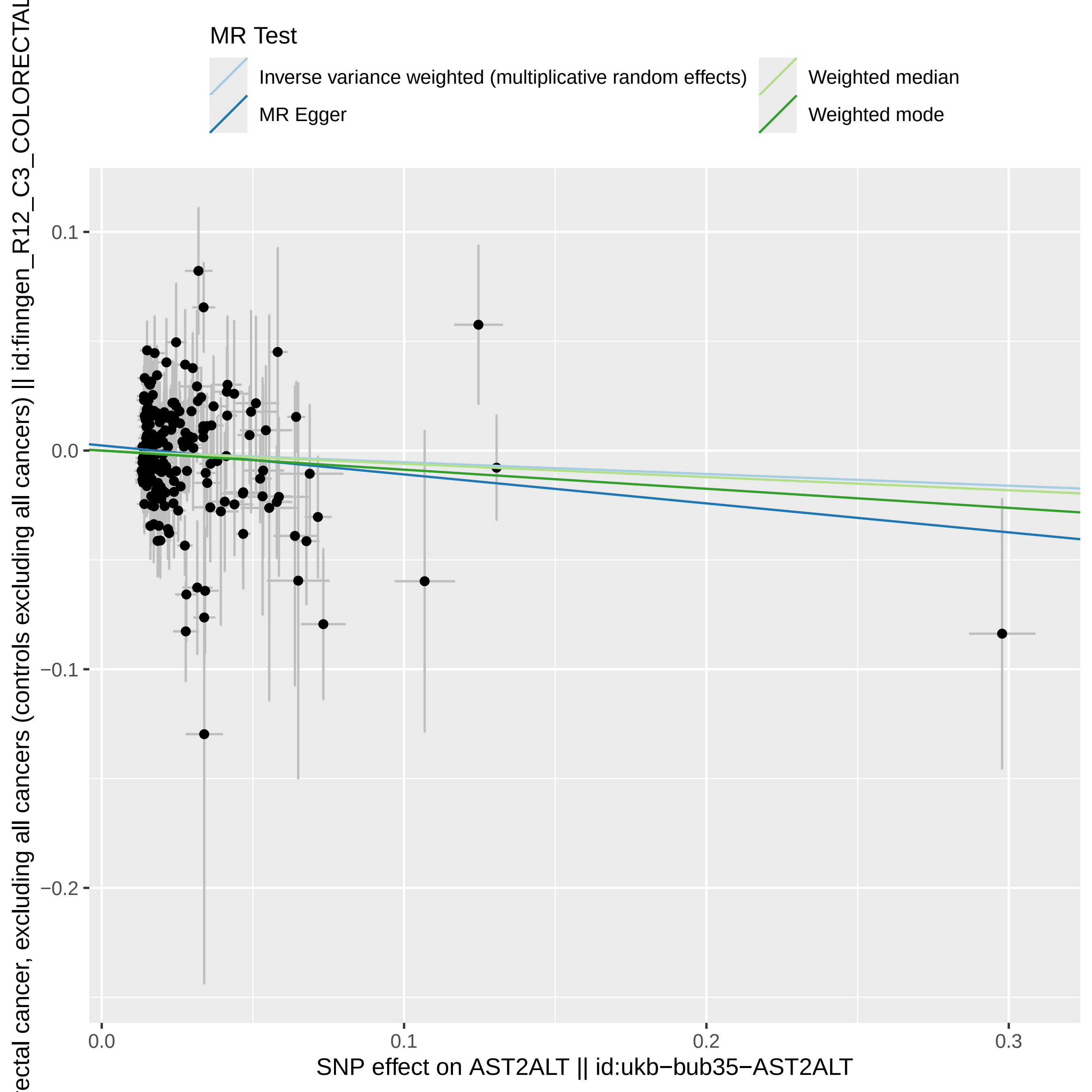

MR Test
Inverse variance weighted (multiplicative random effects)
MR Egger
Weighted median
Weighted mode
0.1
0.0
−0.1
−0.2
0.0
0.1
0.2
0.3
SNP effect on AST2ALT || id:ukb−bub35−AST2ALT
ectal cancer, excluding all cancers (controls excluding all cancers) || id:finngen_R12_C3_COLORECTAL

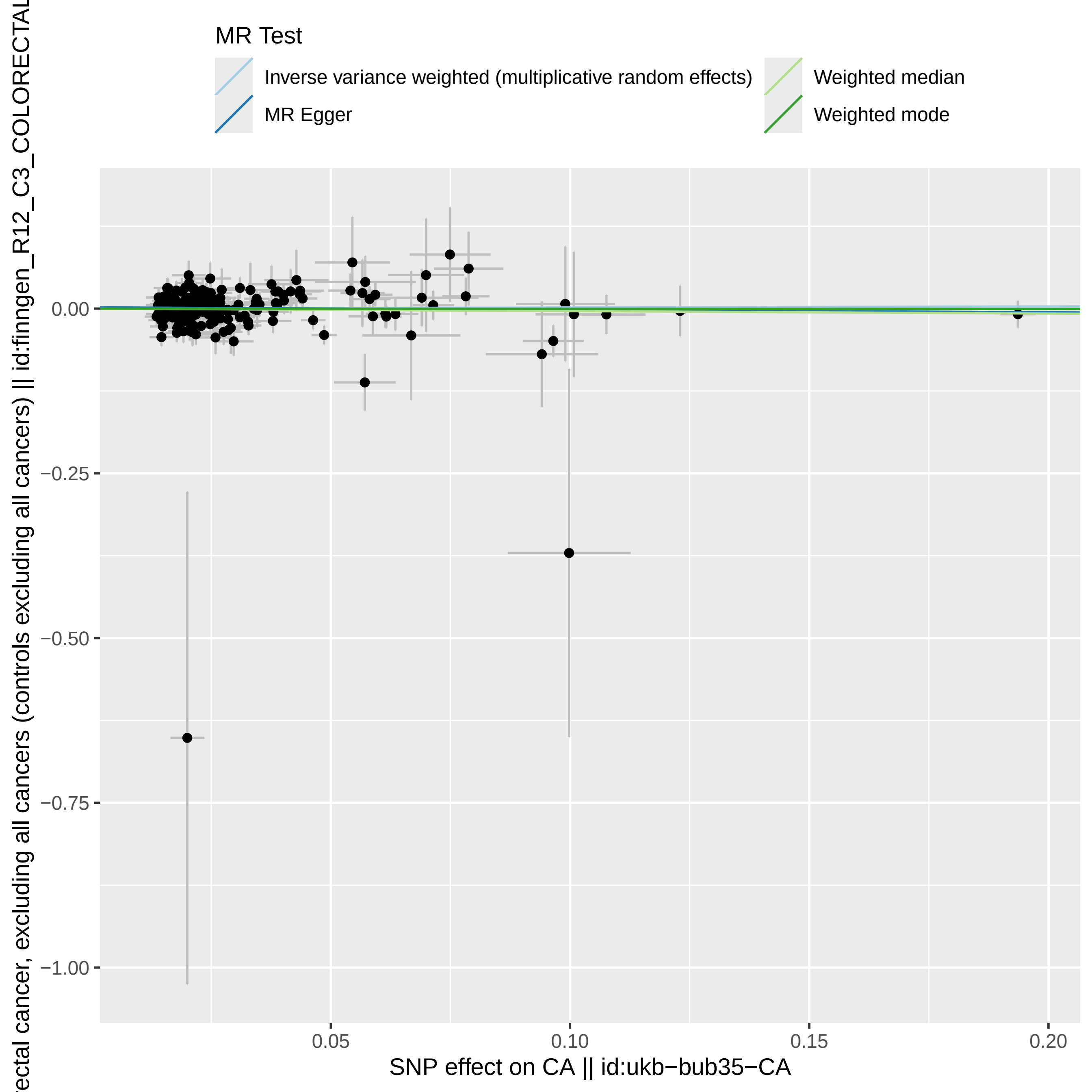

MR Test
Inverse variance weighted (multiplicative random effects)
MR Egger
Weighted median
Weighted mode
0.00
−0.25
−0.50
−0.75
−1.00
0.05
0.10
0.15
0.20
SNP effect on CA || id:ukb−bub35−CA
ectal cancer, excluding all cancers (controls excluding all cancers) || id:finngen_R12_C3_COLORECTAL

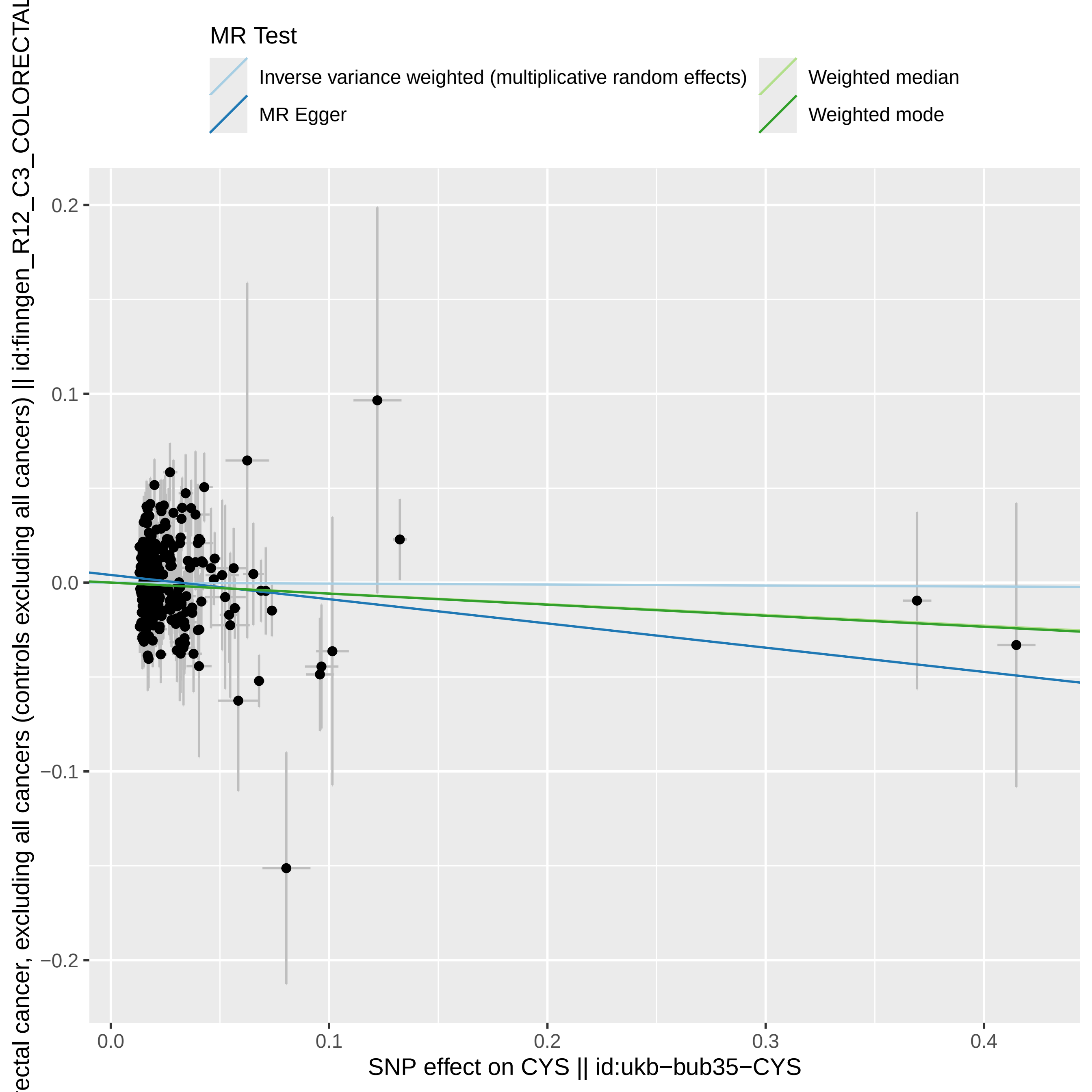
MR Test
Inverse variance weighted (multiplicative random effects)
Weighted median
MR Egger
Weighted mode
0.2
0.1
0.0
−0.1
−0.2
0.0
0.1
0.2
0.3
0.4
SNP effect on CYS || id:ukb−bub35−CYS
ectal cancer, excluding all cancers (controls excluding all cancers) || id:finngen_R12_C3_COLORECTAL

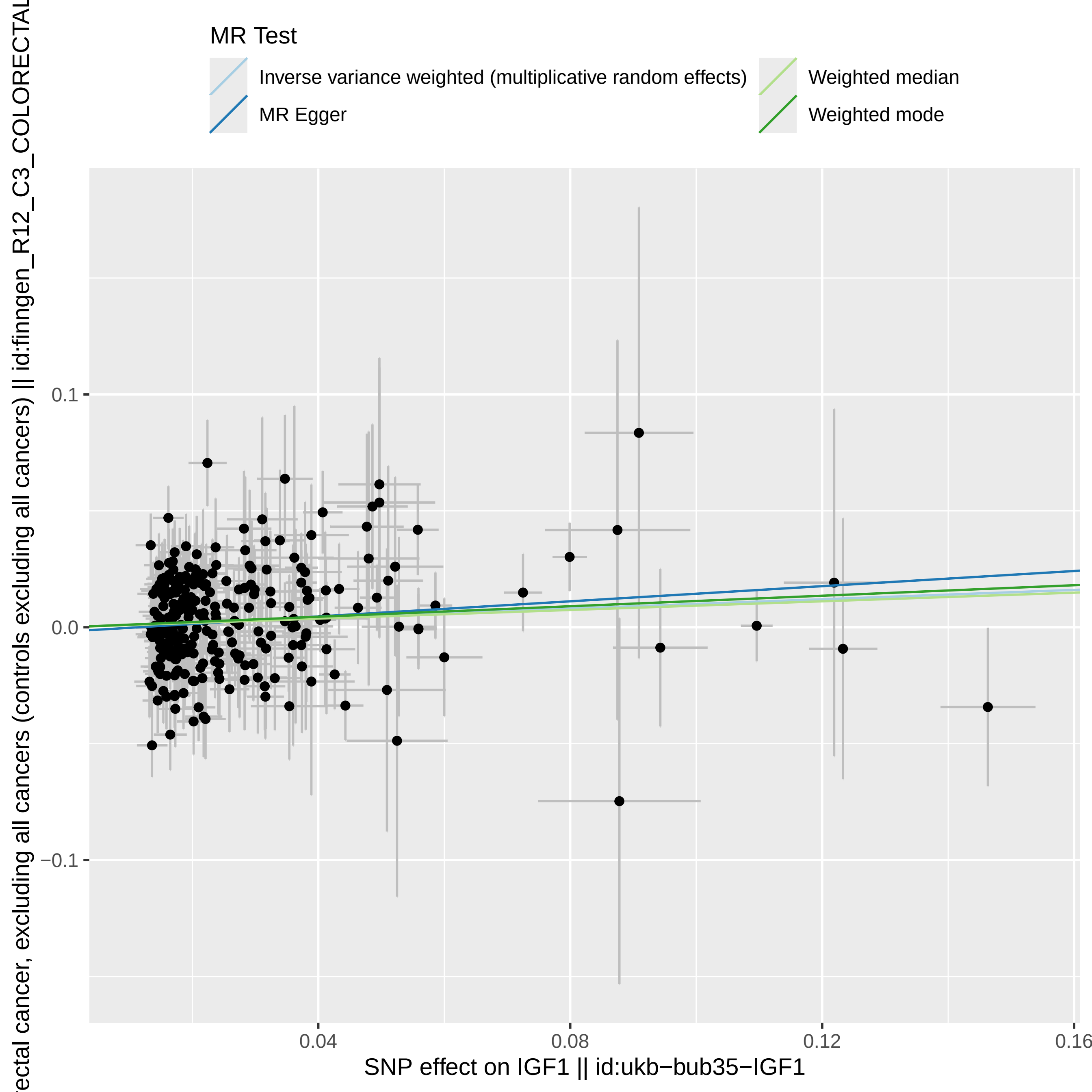
MR Test
Inverse variance weighted (multiplicative random effects)
MR Egger
Weighted median
Weighted mode
0.1
0.0
−0.1
0.04
0.08
0.12
0.16
SNP effect on IGF1 || id:ukb−bub35−IGF1
ectal cancer, excluding all cancers (controls excluding all cancers) || id:finngen_R12_C3_COLORECTAL

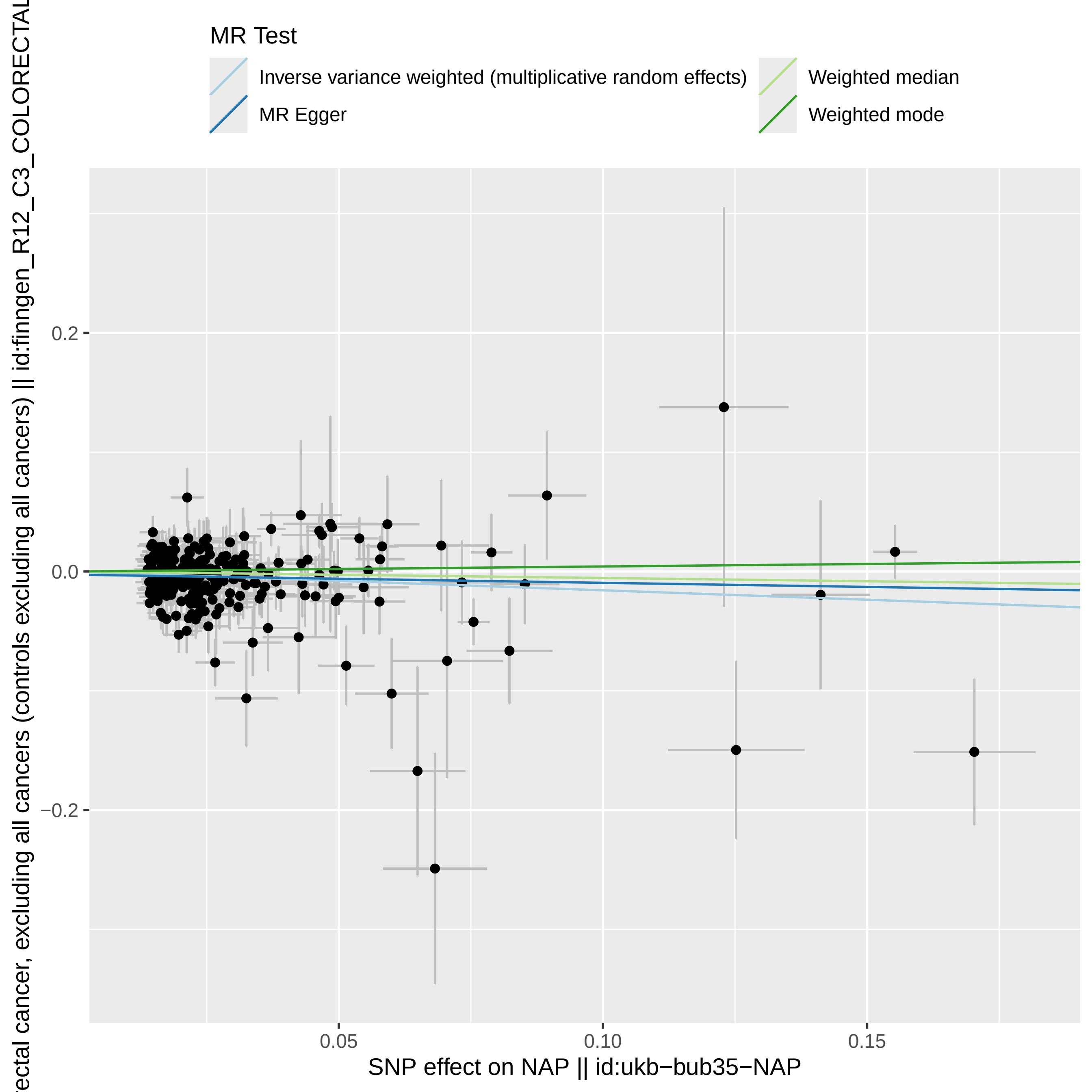

MR Test
Inverse variance weighted (multiplicative random effects)
MR Egger
Weighted median
Weighted mode
0.2
0.0
−0.2
0.05
0.10
0.15
SNP effect on NAP || id:ukb−bub35−NAP
ectal cancer, excluding all cancers (controls excluding all cancers) || id:finngen_R12_C3_COLORECTAL

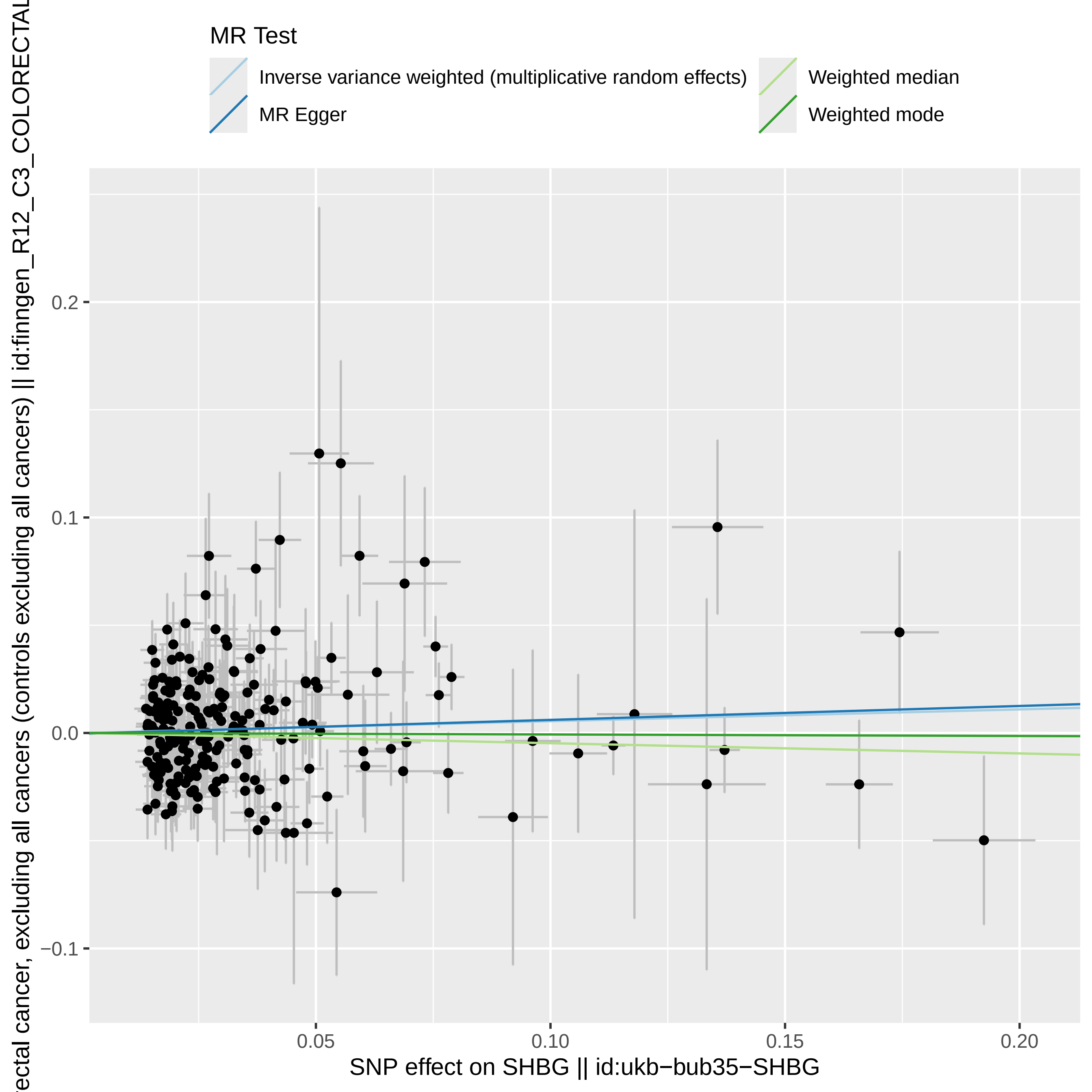

MR Test
Inverse variance weighted (multiplicative random effects)
MR Egger
Weighted median
Weighted mode
0.2
0.1
0.0
−0.1
0.05
0.10
0.15
0.20
SNP effect on SHBG || id:ukb−bub35−SHBG
ectal cancer, excluding all cancers (controls excluding all cancers) || id:finngen_R12_C3_COLORECTAL

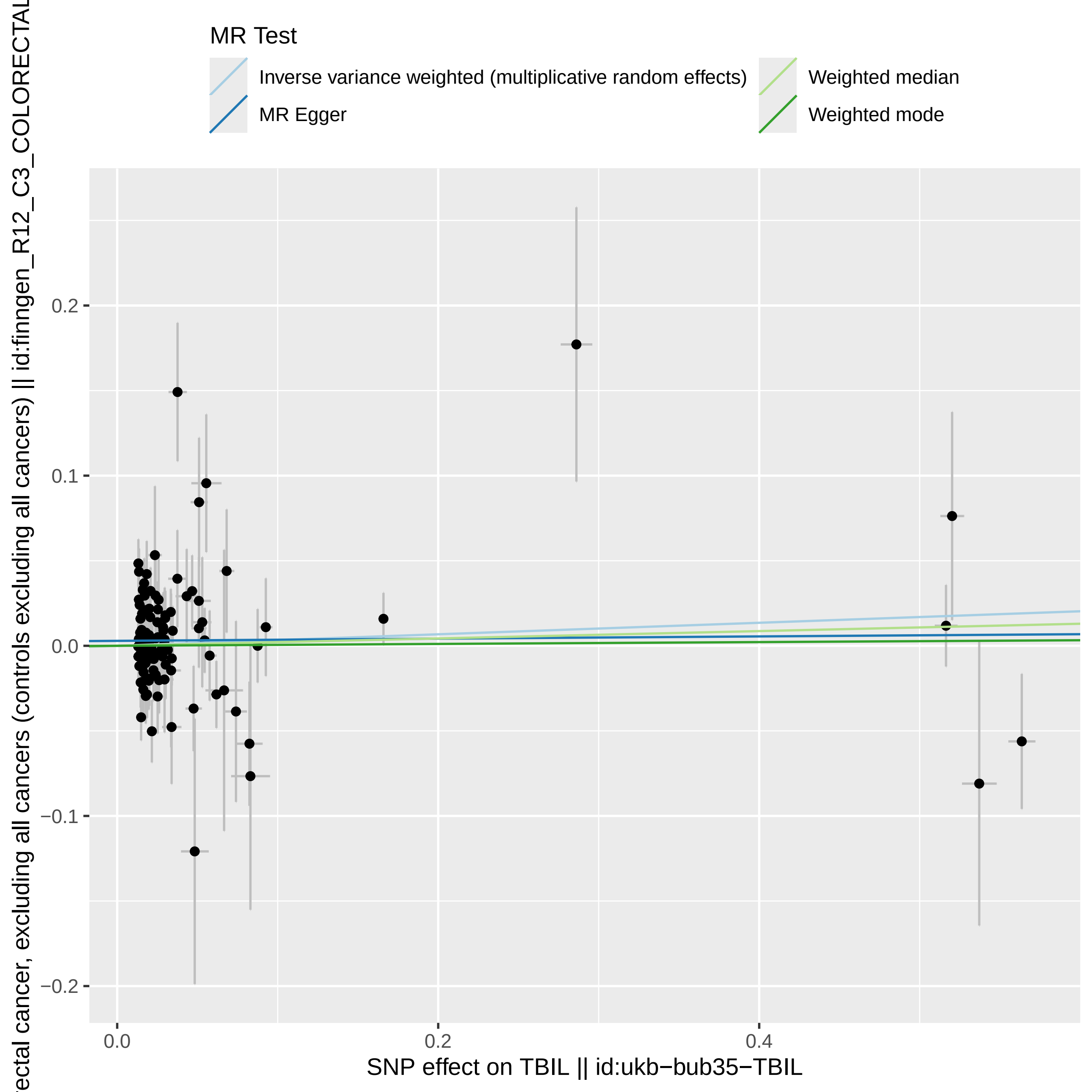

MR Test
Inverse variance weighted (multiplicative random effects)
MR Egger
Weighted median
Weighted mode
0.2
0.1
0.0
−0.1
−0.2
0.0
0.2
0.4
SNP effect on TBIL || id:ukb−bub35−TBIL
ectal cancer, excluding all cancers (controls excluding all cancers) || id:finngen_R12_C3_COLORECTAL

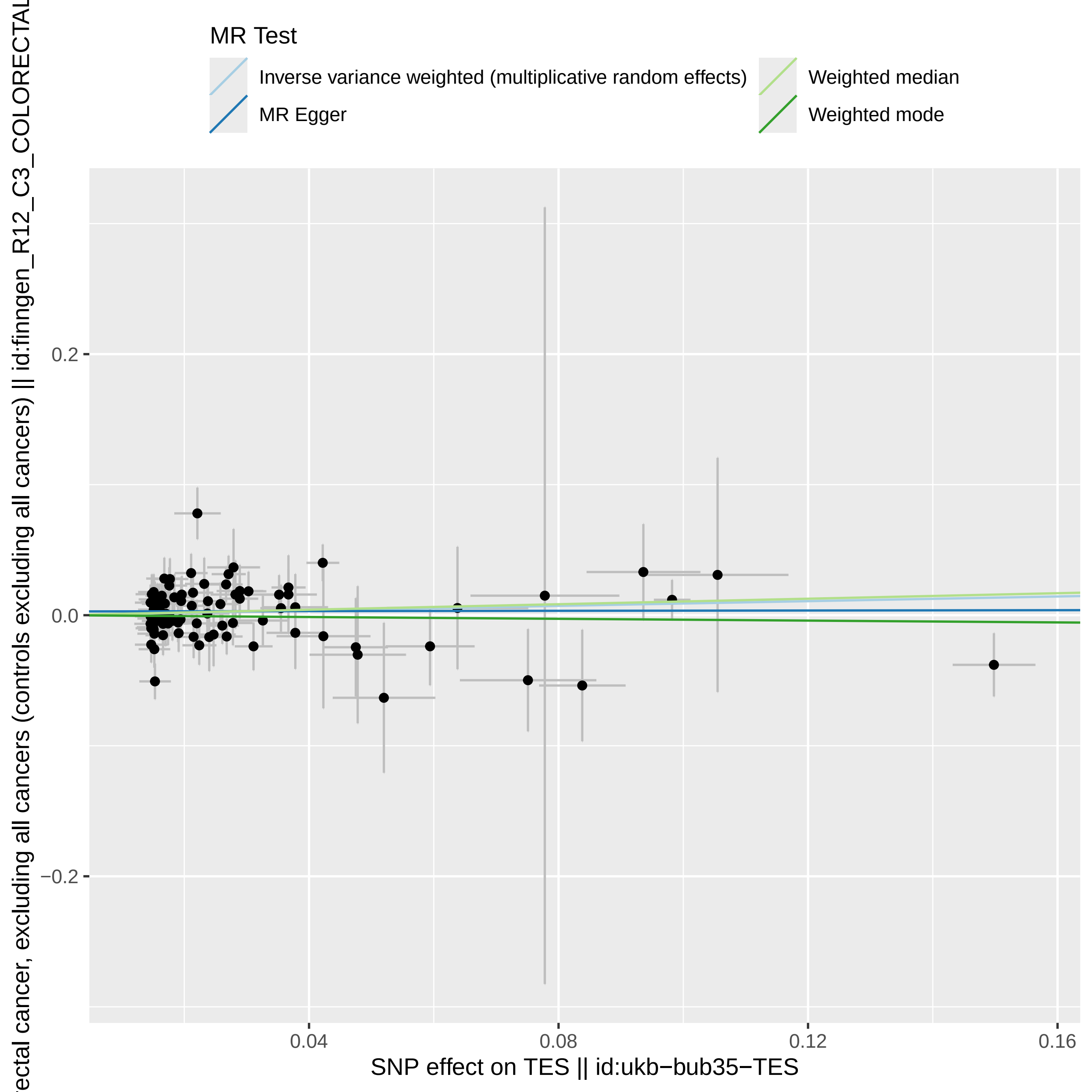

MR Test
Inverse variance weighted (multiplicative random effects)
MR Egger
Weighted median
Weighted mode
0.2
0.0
−0.2
0.04
0.08
0.12
0.16
SNP effect on TES || id:ukb−bub35−TES
ectal cancer, excluding all cancers (controls excluding all cancers) || id:finngen_R12_C3_COLORECTAL

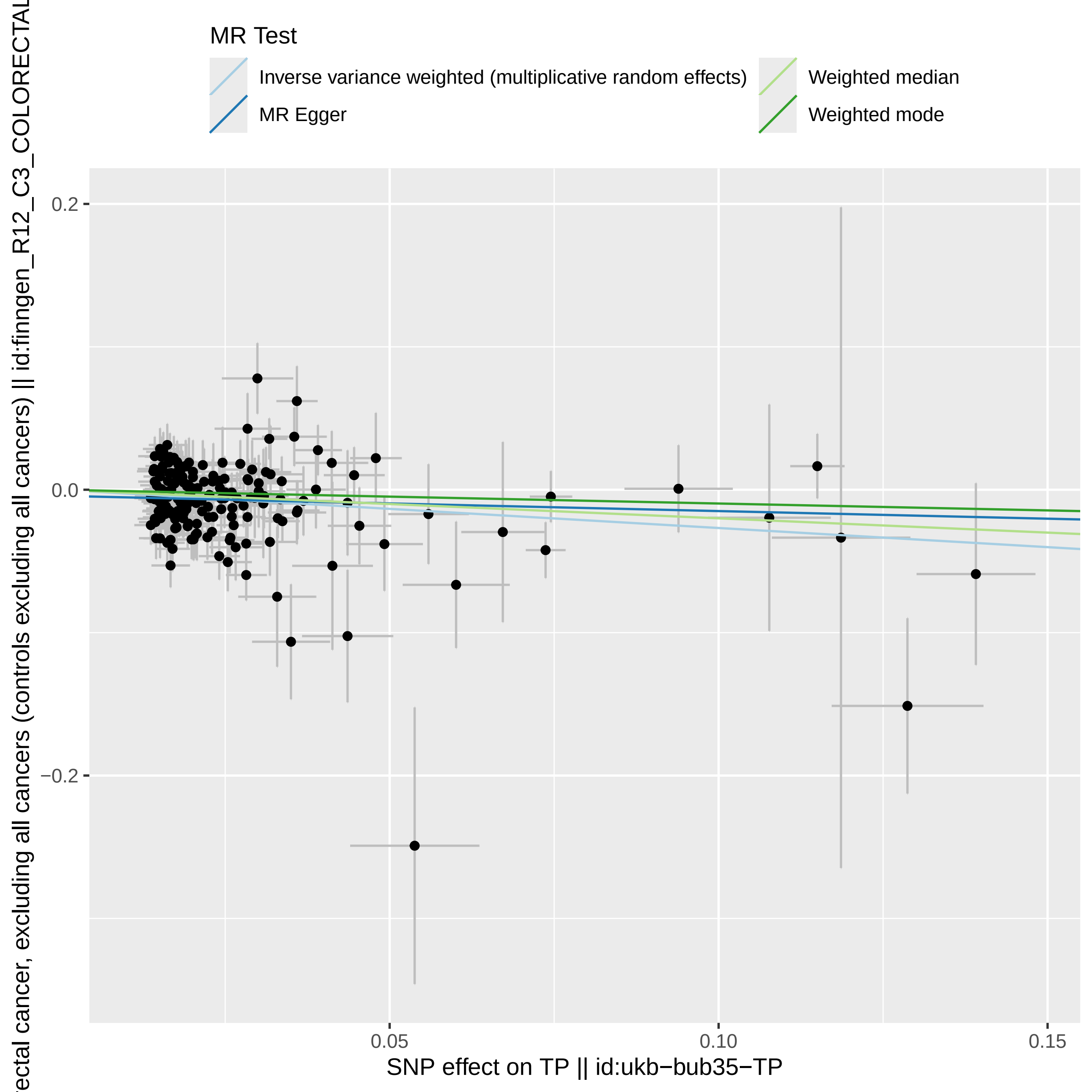

MR Test
Inverse variance weighted (multiplicative random effects)
Weighted median
MR Egger
Weighted mode
0.2
0.0
−0.2
0.05
0.10
0.15
SNP effect on TP || id:ukb−bub35−TP
ectal cancer, excluding all cancers (controls excluding all cancers) || id:finngen_R12_C3_COLORECTAL

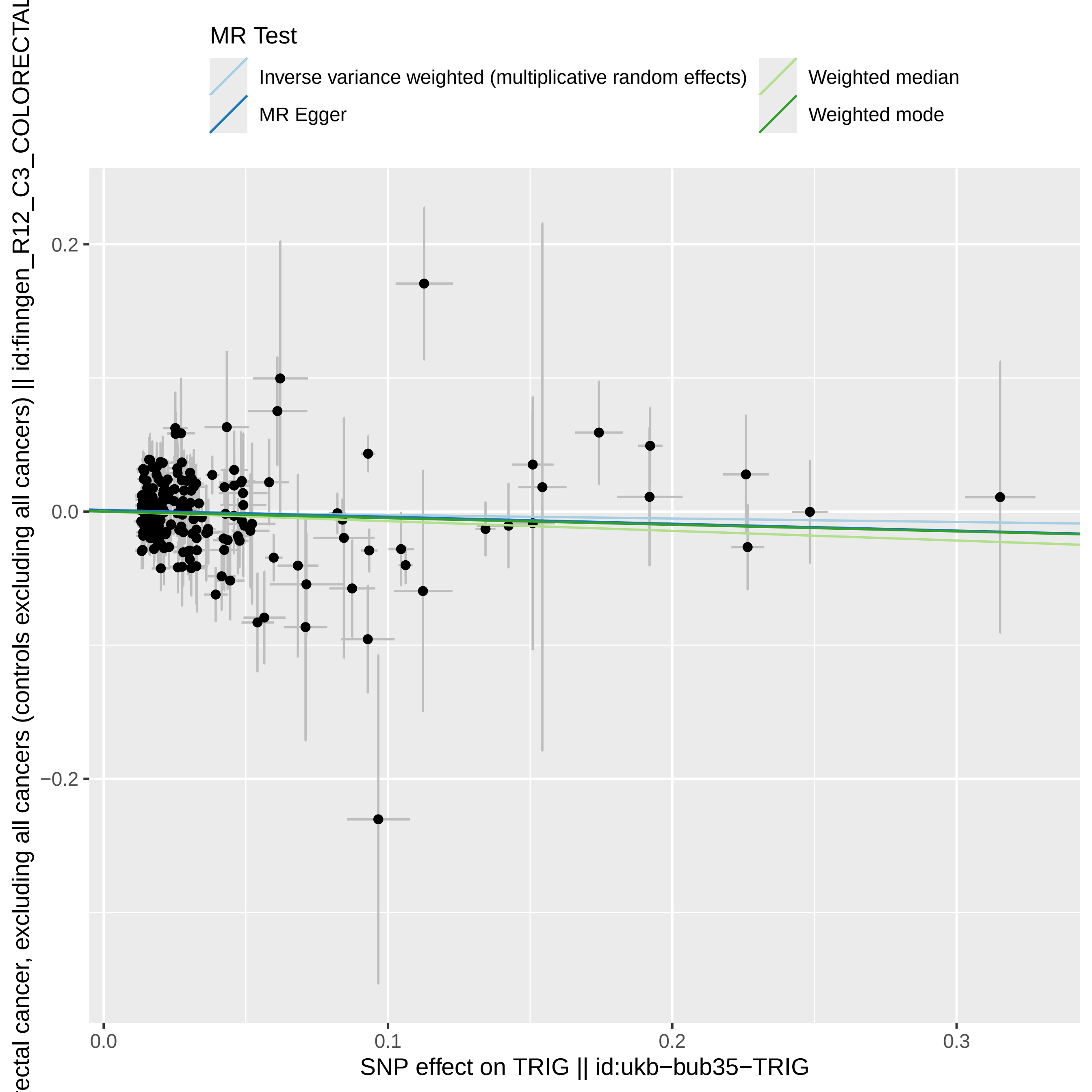

MR Test
Inverse variance weighted (multiplicative random effects)
MR Egger
Weighted median
Weighted mode
0.2
0.0
−0.2
0.0
0.1
0.2
0.3
SNP effect on TRIG || id:ukb−bub35−TRIG
ectal cancer, excluding all cancers (controls excluding all cancers) || id:finngen_R12_C3_COLORECTAL

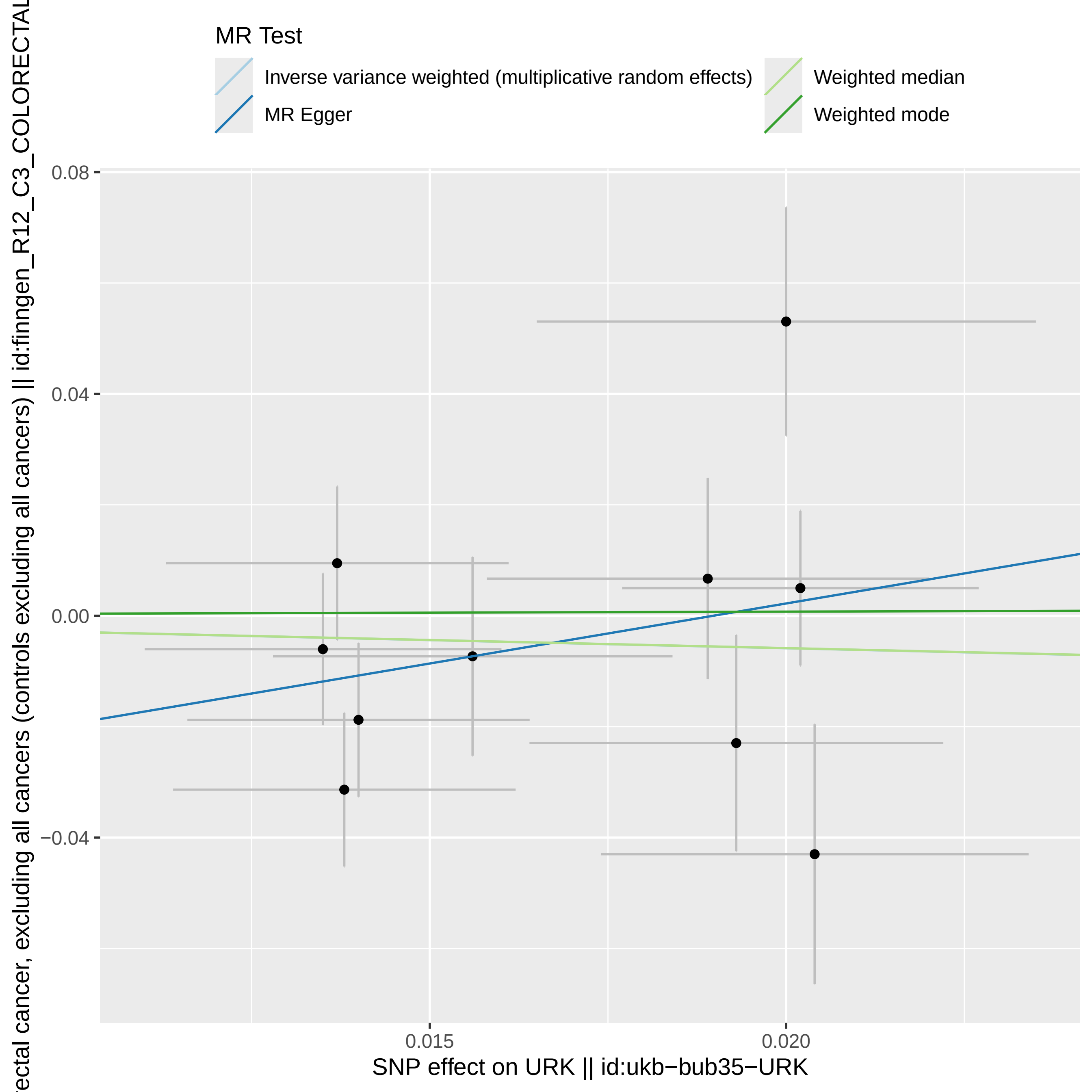
MR Test
Inverse variance weighted (multiplicative random effects)
MR Egger
Weighted median
Weighted mode
0.08
0.04
0.00
−0.04
0.015
0.020
SNP effect on URK || id:ukb−bub35−URK
ectal cancer, excluding all cancers (controls excluding all cancers) || id:finngen_R12_C3_COLORECTAL

Supplementary Figure 3. All Forest plot results.

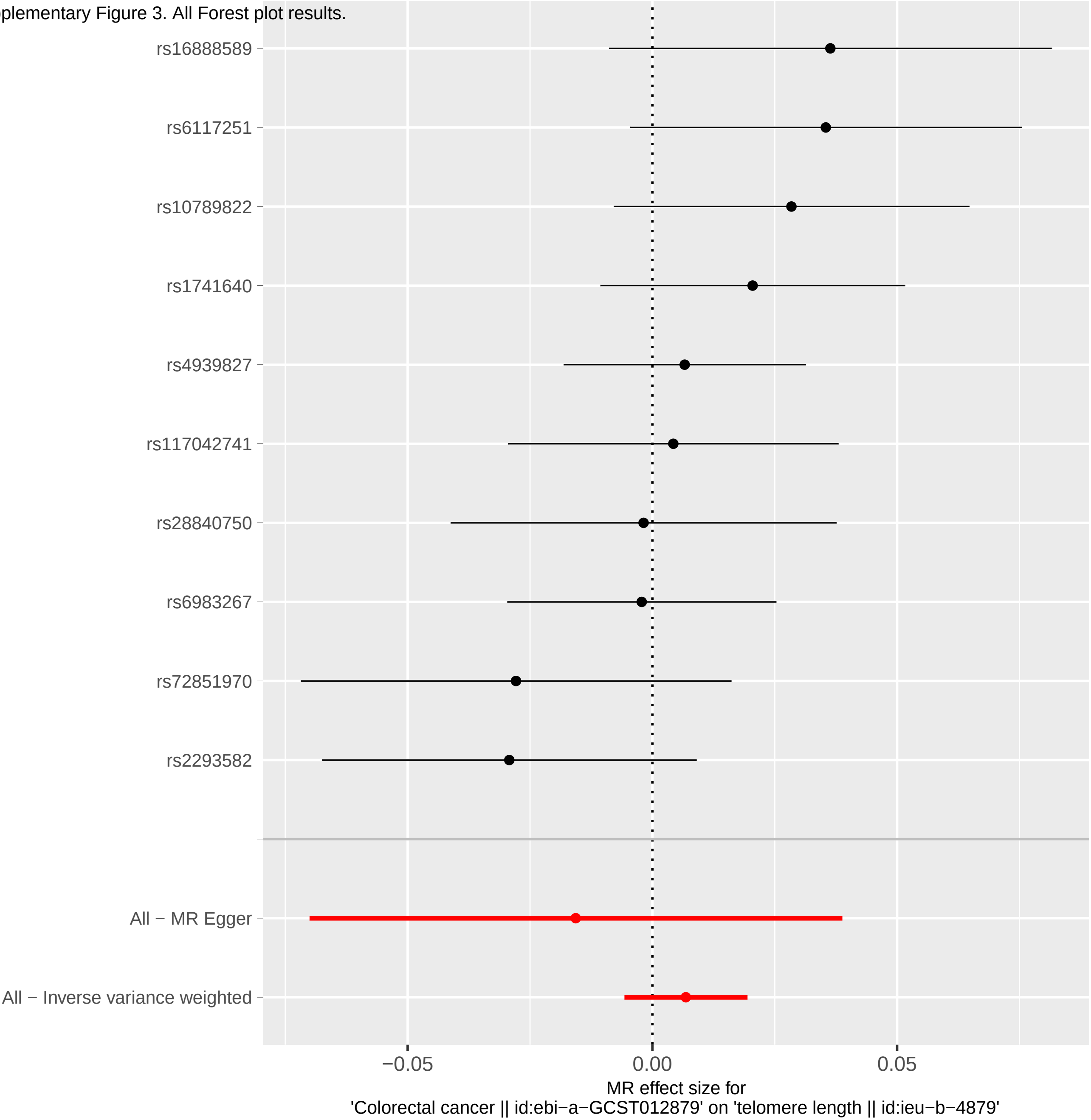

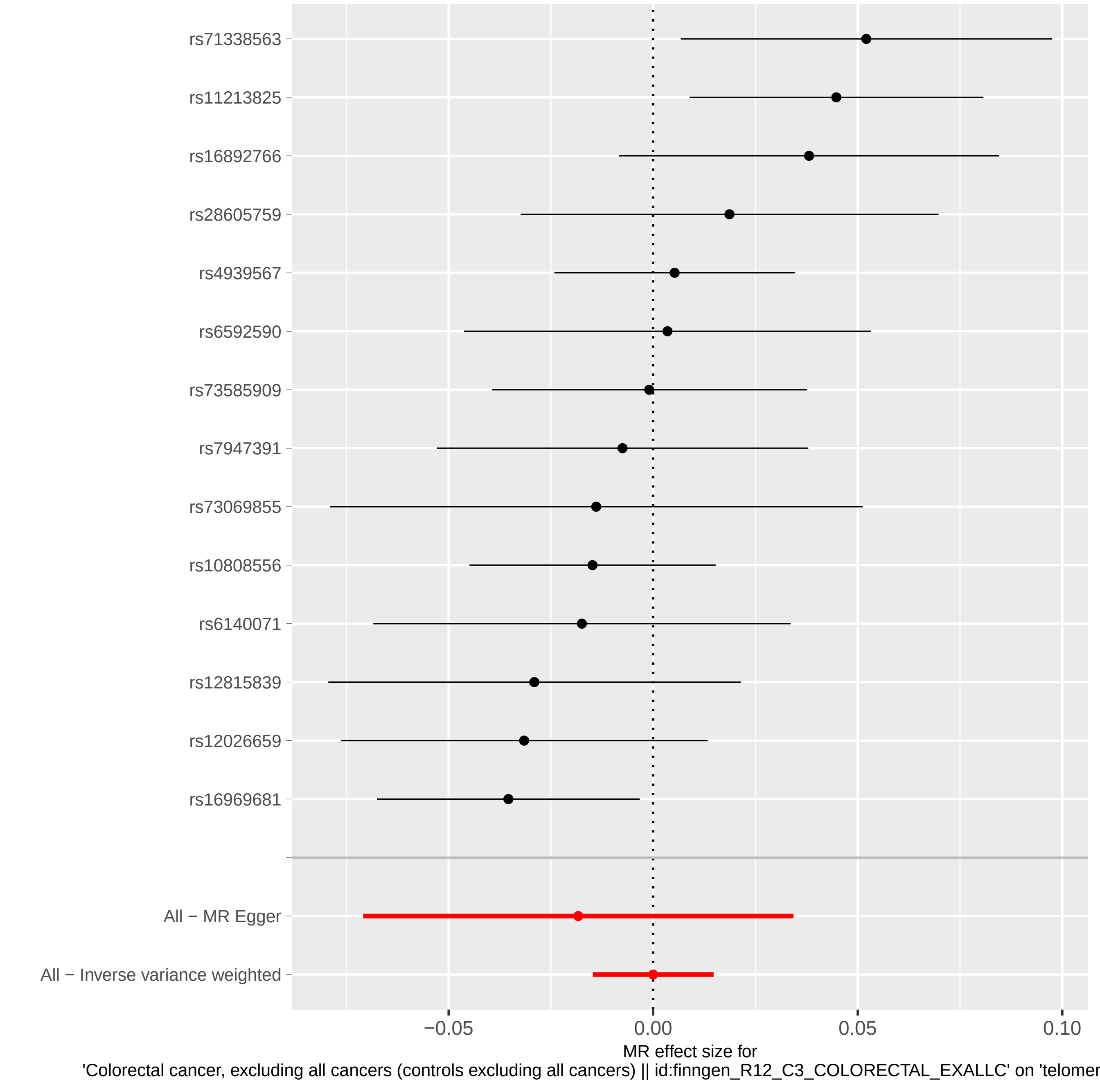
rs71338563
rs11213825
rs16892766
rs28605759
rs4939567
rs6592590
rs73585909
rs7947391
rs73069855
rs10808556
rs6140071
rs12815839
rs12026659
rs16969681
All − MR Egger
All − Inverse variance weighted
−0.05
0.00
0.05
0.10
MR effect size for
'Colorectal cancer, excluding all cancers (controls excluding all cancers) || id:finngen_R12_C3_COLORECTAL_EXALLC' on 'telomer

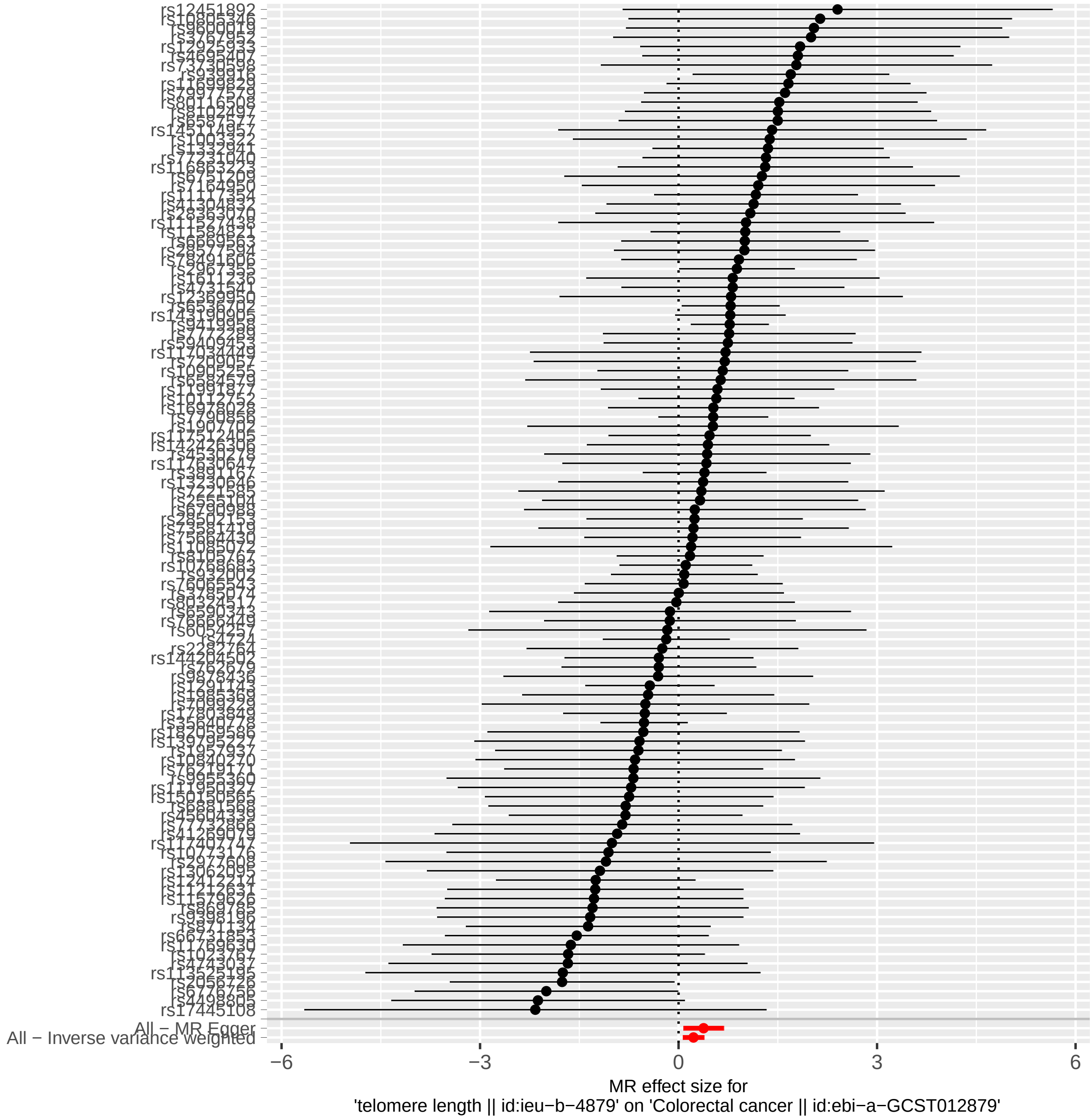
All − MR Egger
All − Inverse variance weighted
−6
−3
0
3
6
MR effect size for
'telomere length || id:ieu−b−4879' on 'Colorectal cancer || id:ebi−a−GCST012879'

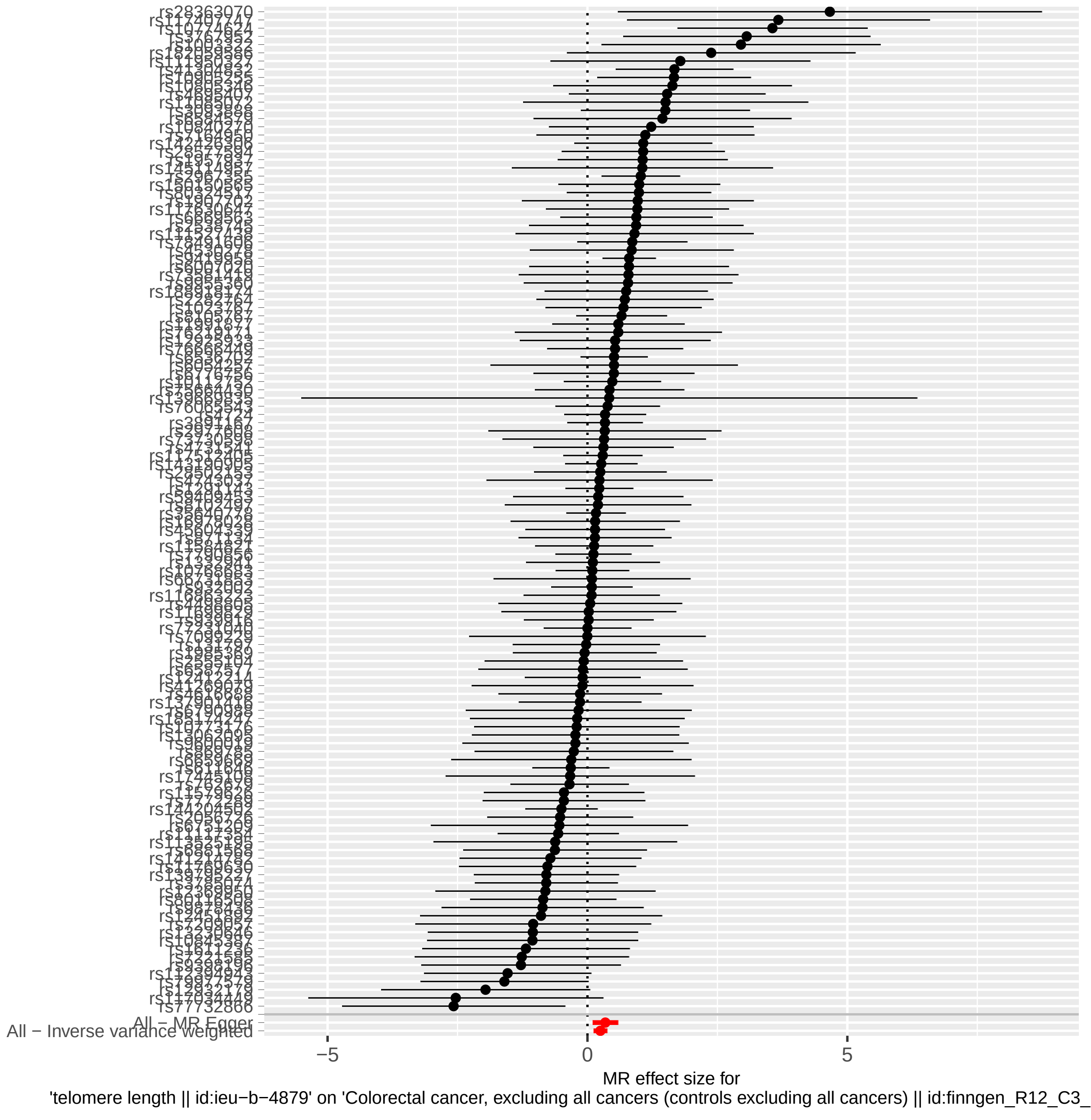
rs28363070
rs77732860
All − MR Egger
All − Inverse variance weighted
−5
0
5
MR effect size for
'telomere length || id:ieu−b−4879' on 'Colorectal cancer, excluding all cancers (controls excluding all cancers) || id:finngen_R12_C3_

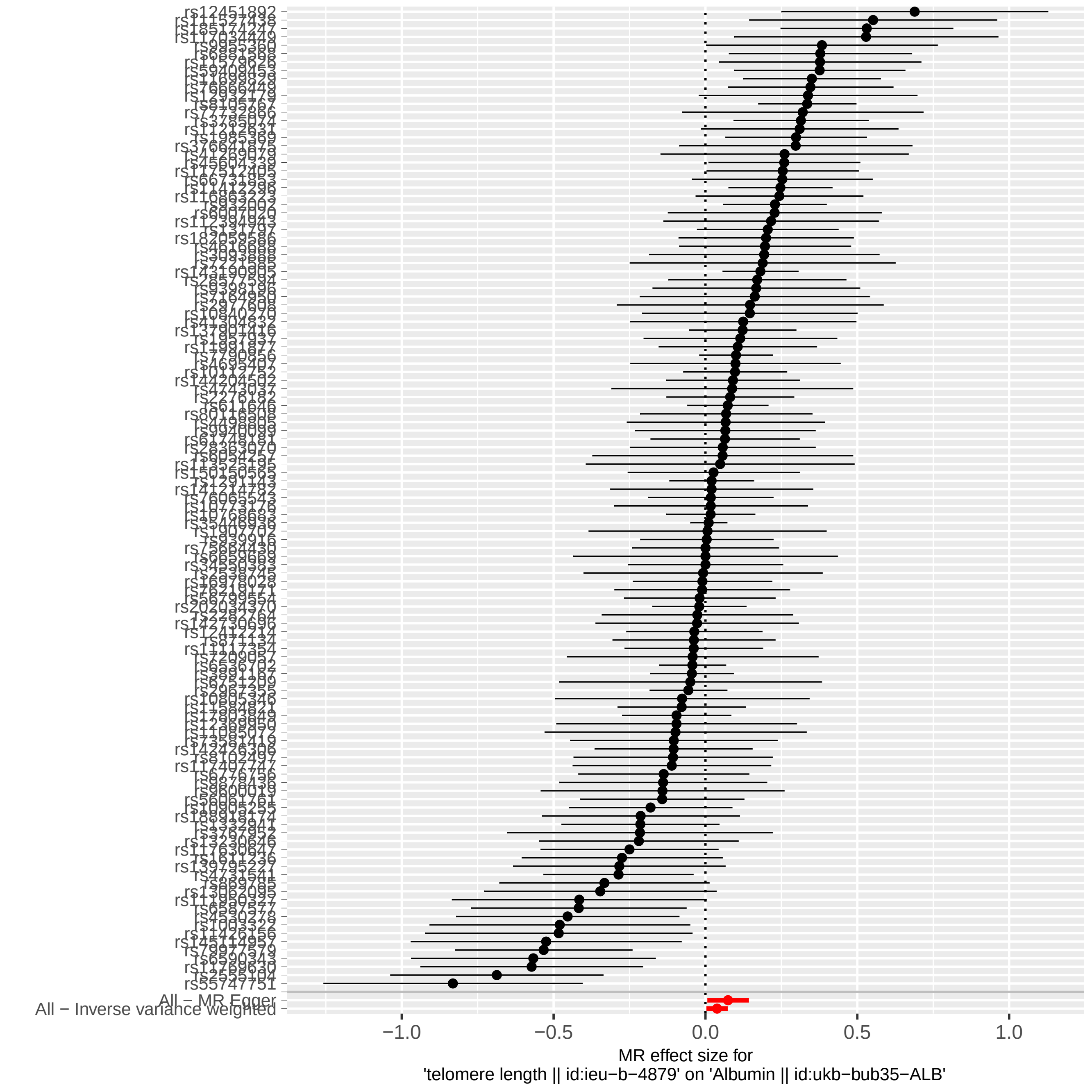
rs12451892
rs55747751
All − MR Egger
All − Inverse variance weighted
−1.0
−0.5
0.0
0.5
1.0
MR effect size for
'telomere length || id:ieu−b−4879' on 'Albumin || id:ukb−bub35−ALB'

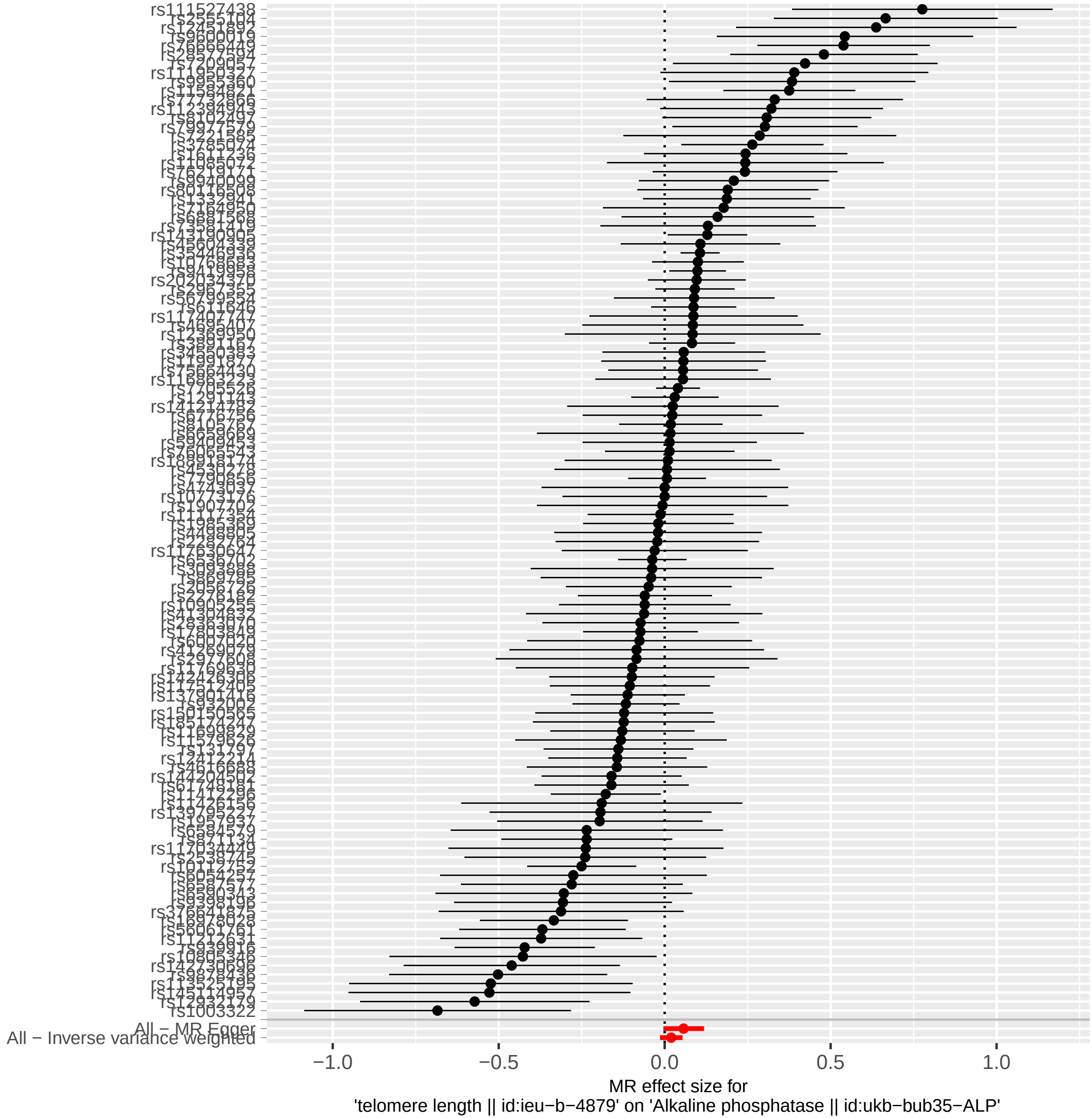
rs111527438
rs1003322
All − MR Egger
All − Inverse variance weighted
−1.0
−0.5
0.0
0.5
1.0
MR effect size for
'telomere length || id:ieu−b−4879' on 'Alkaline phosphatase || id:ukb−bub35−ALP'

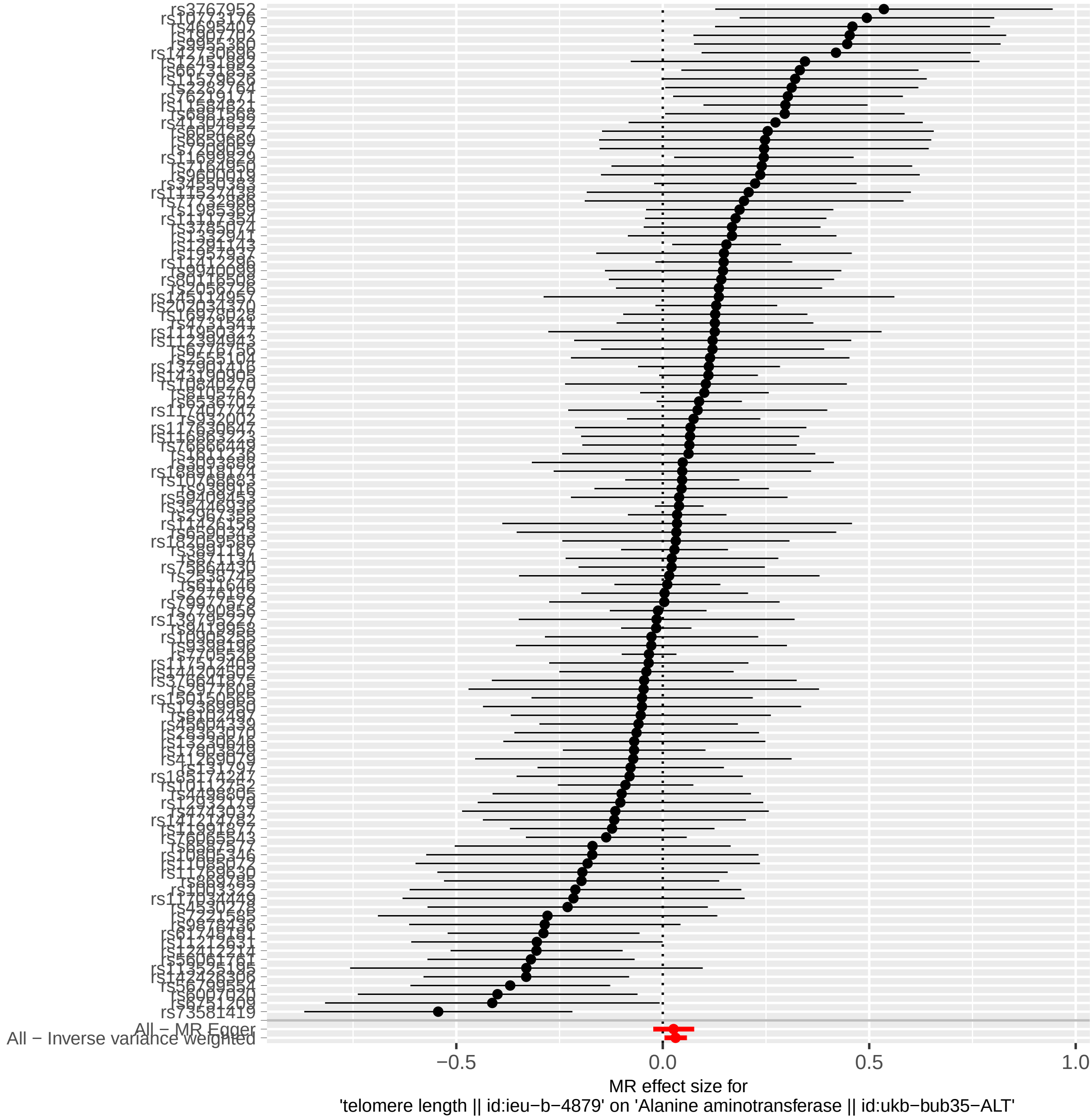

rs3767952
rs73581419
All − MR Egger
All − Inverse variance weighted
−0.5
0.0
0.5
1.0
MR effect size for
'telomere length || id:ieu−b−4879' on 'Alanine aminotransferase || id:ukb−bub35−ALT'

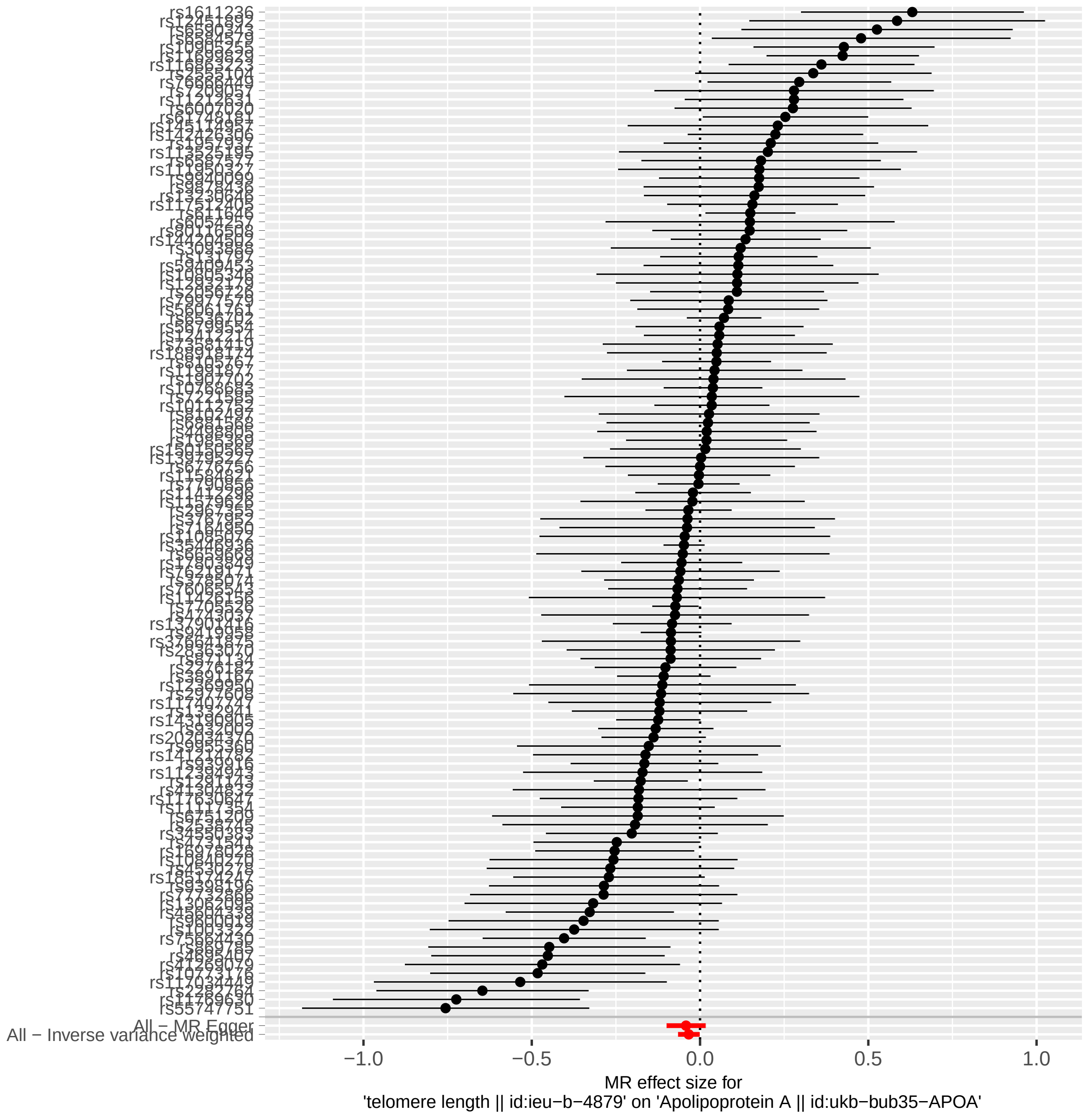
rs1611236
rs55747751
All − MR Egger
All − Inverse variance weighted
−1.0
−0.5
0.0
0.5
1.0
MR effect size for
'telomere length || id:ieu−b−4879' on 'Apolipoprotein A || id:ukb−bub35−APOA'

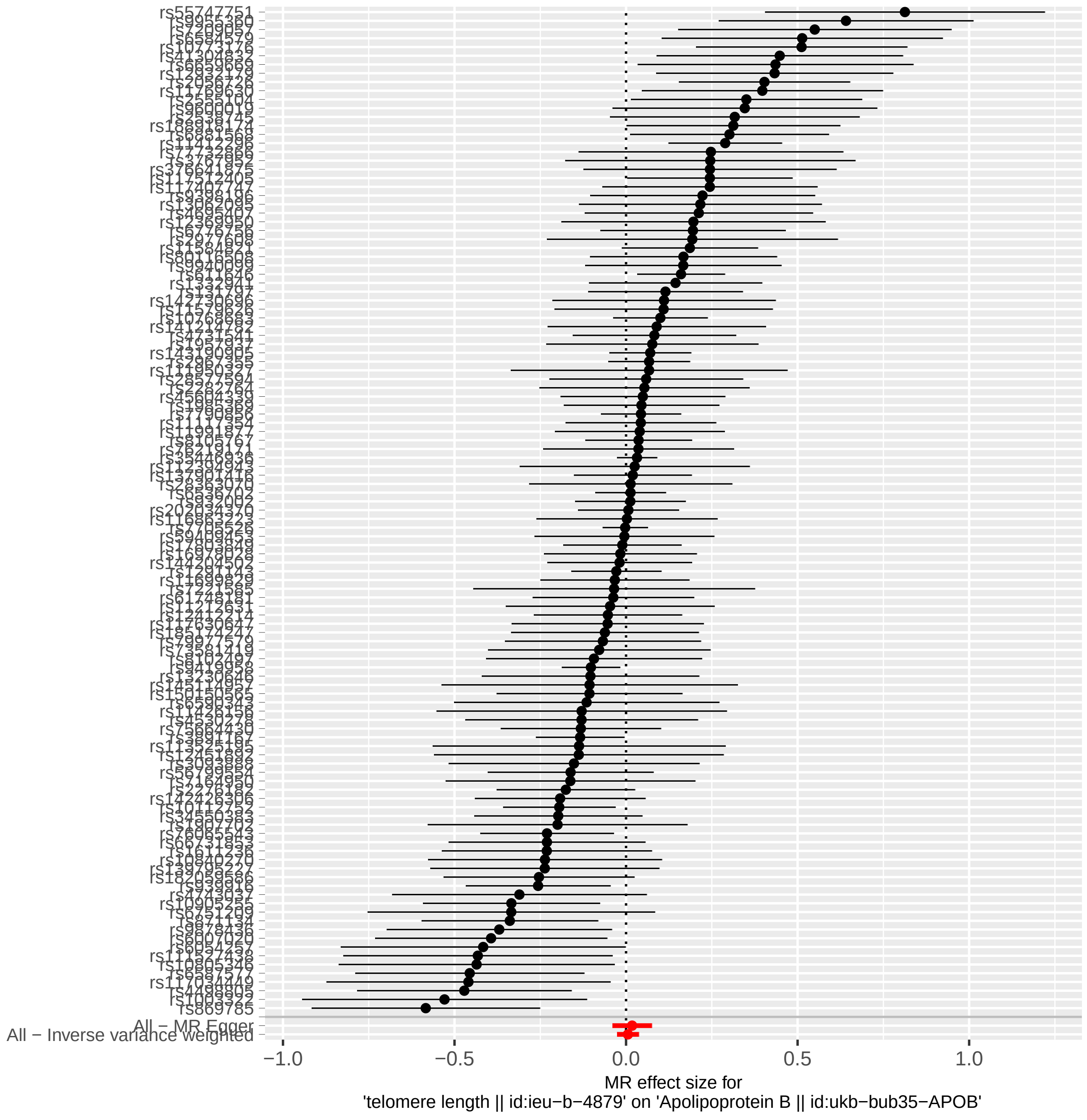

rs55747751
rs869785
All − MR Egger
All − Inverse variance weighted
−1.0
−0.5
0.0
0.5
1.0
MR effect size for
'telomere length || id:ieu−b−4879' on 'Apolipoprotein B || id:ukb−bub35−APOB'

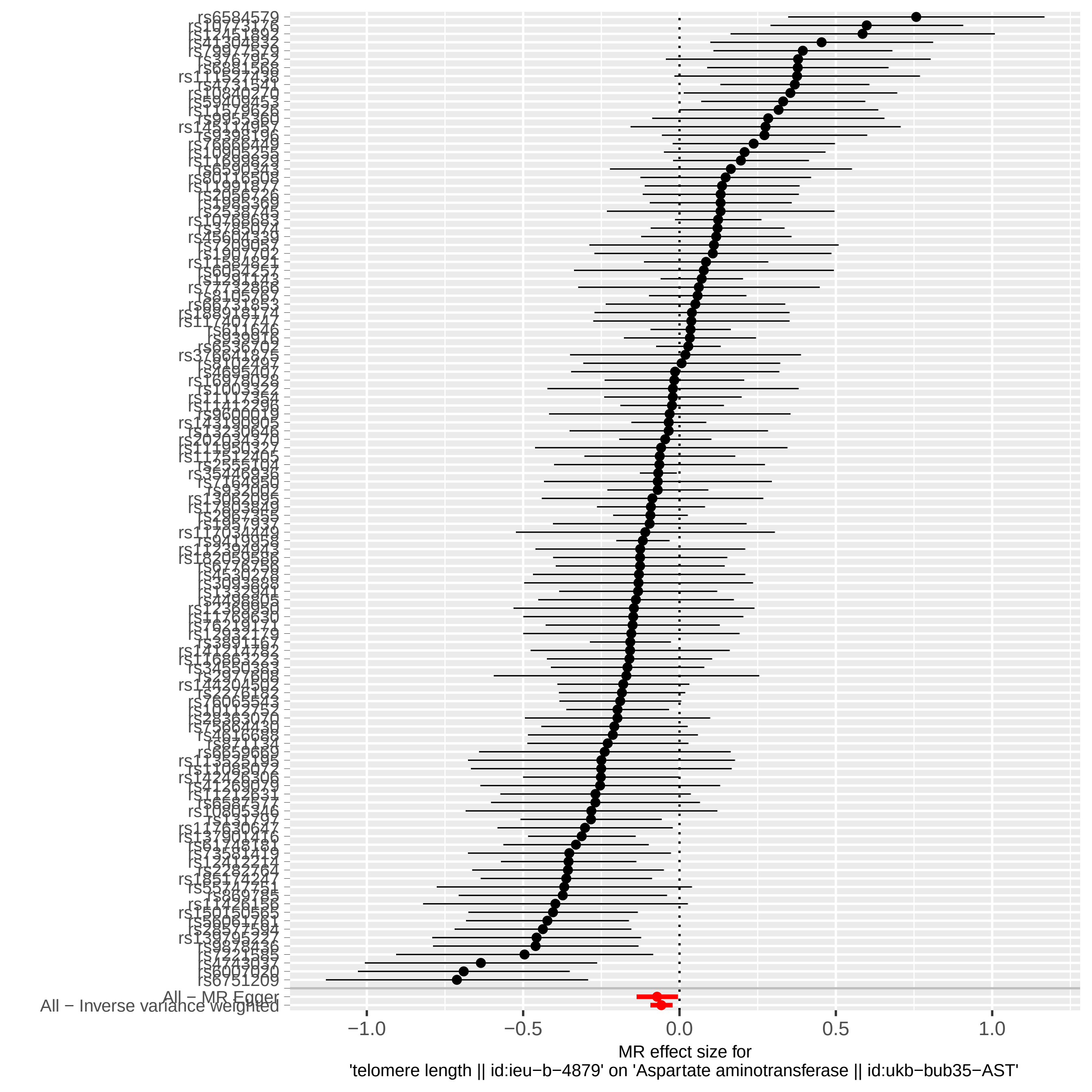

rs6584579
rs6751209
All − MR Egger
All − Inverse variance weighted
−1.0
−0.5
0.0
0.5
1.0
MR effect size for
'telomere length || id:ieu−b−4879' on 'Aspartate aminotransferase || id:ukb−bub35−AST'

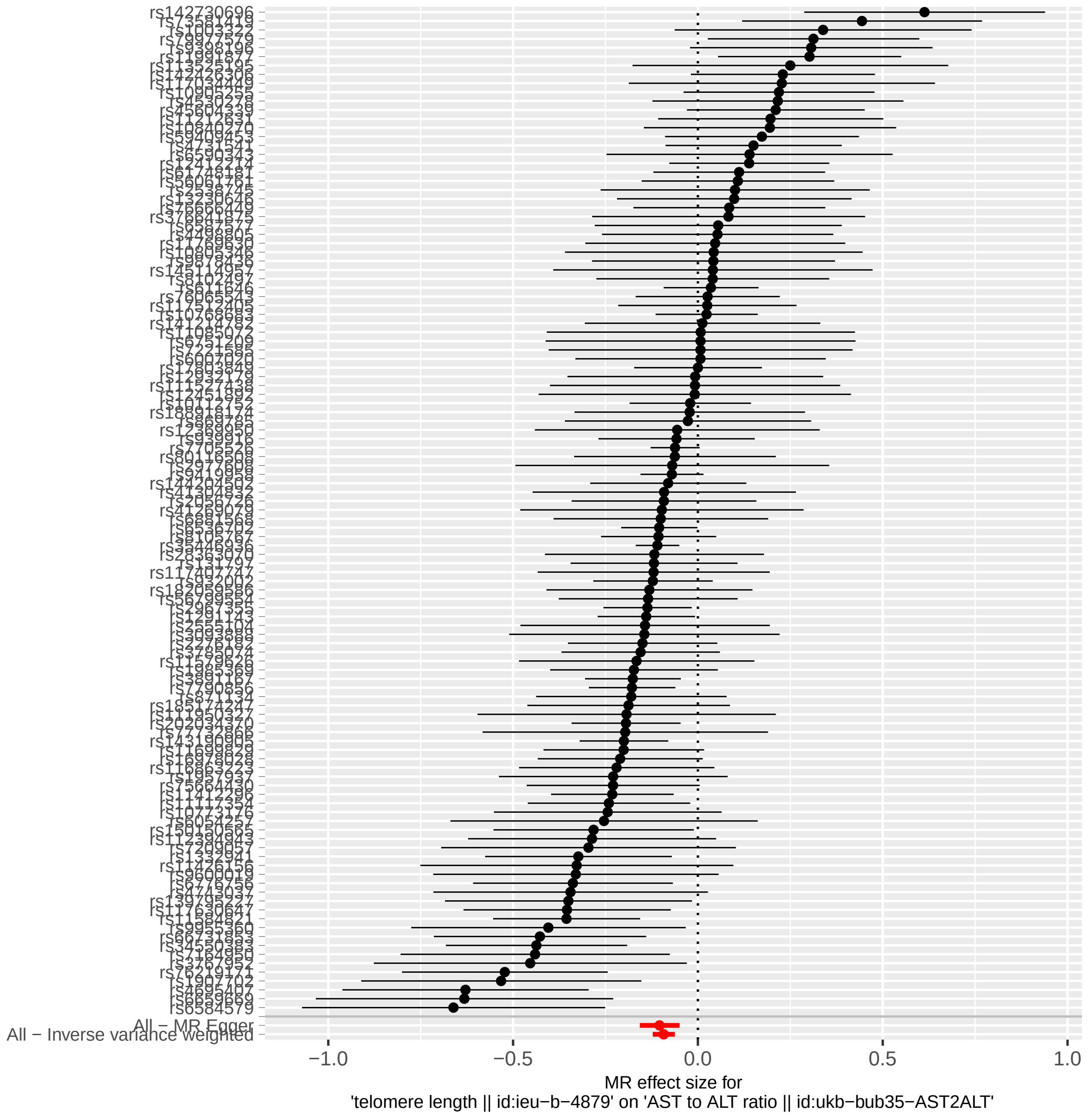

rs142730696
rs6584579
All − MR Egger
All − Inverse variance weighted
−1.0
−0.5
0.0
0.5
1.0
MR effect size for
'telomere length || id:ieu−b−4879' on 'AST to ALT ratio || id:ukb−bub35−AST2ALT'

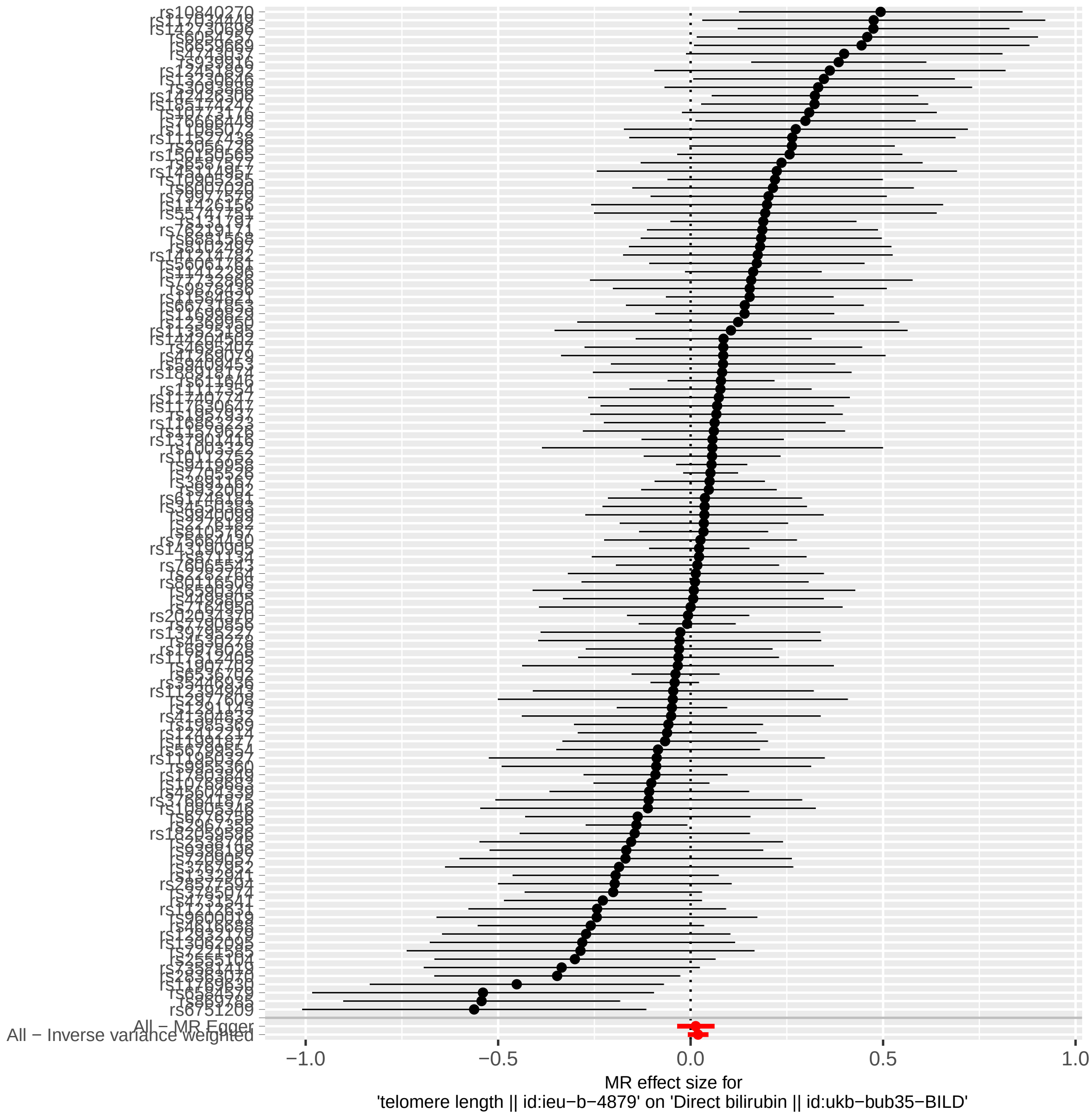
rs10840270
rs6751209
All − MR Egger
All − Inverse variance weighted
−1.0
−0.5
0.0
0.5
1.0
MR effect size for
'telomere length || id:ieu−b−4879' on 'Direct bilirubin || id:ukb−bub35−BILD'

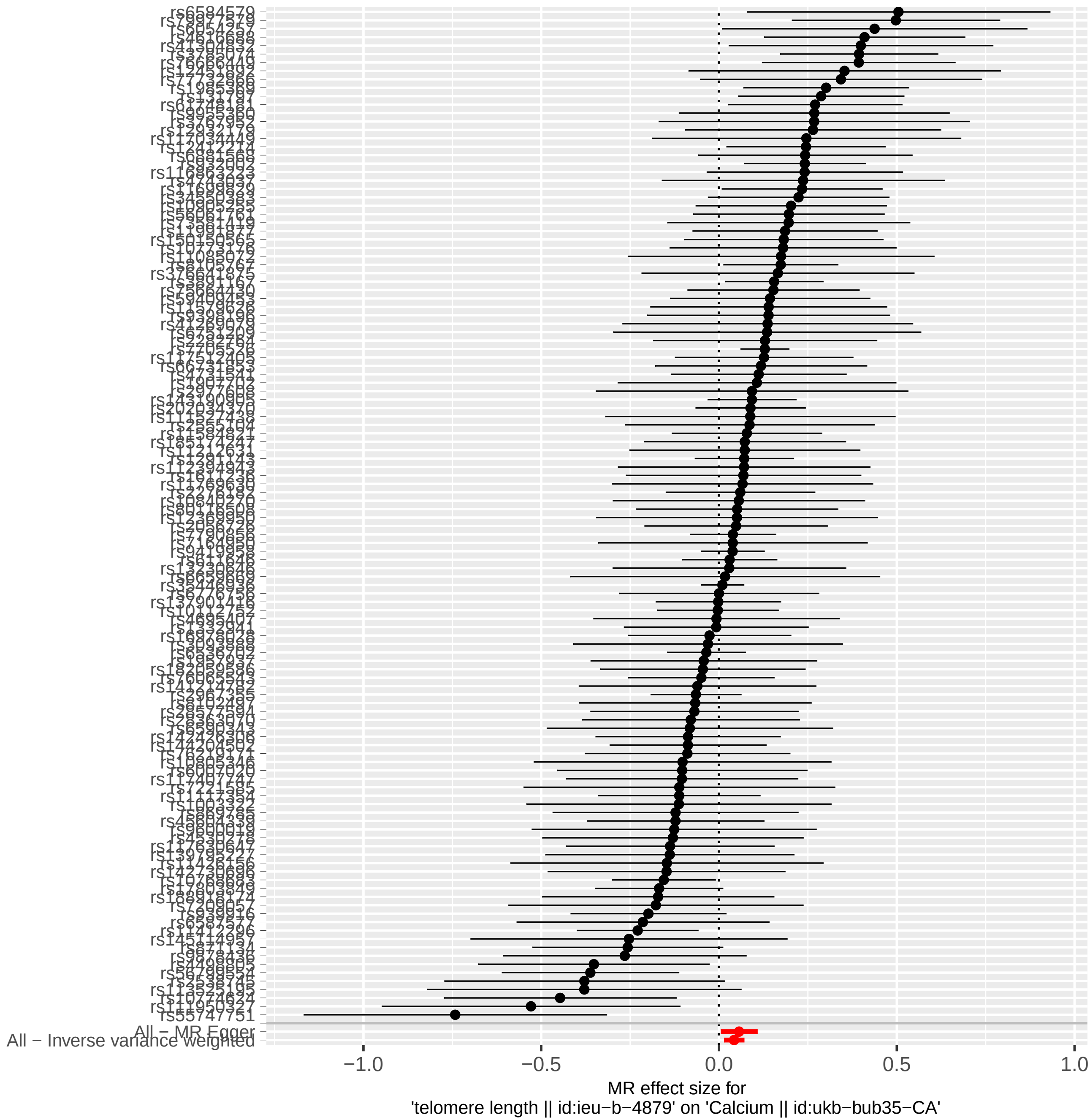
rs6584579
rs55747751
All − MR Egger
All − Inverse variance weighted
−1.0
−0.5
0.0
0.5
1.0
MR effect size for
'telomere length || id:ieu−b−4879' on 'Calcium || id:ukb−bub35−CA'

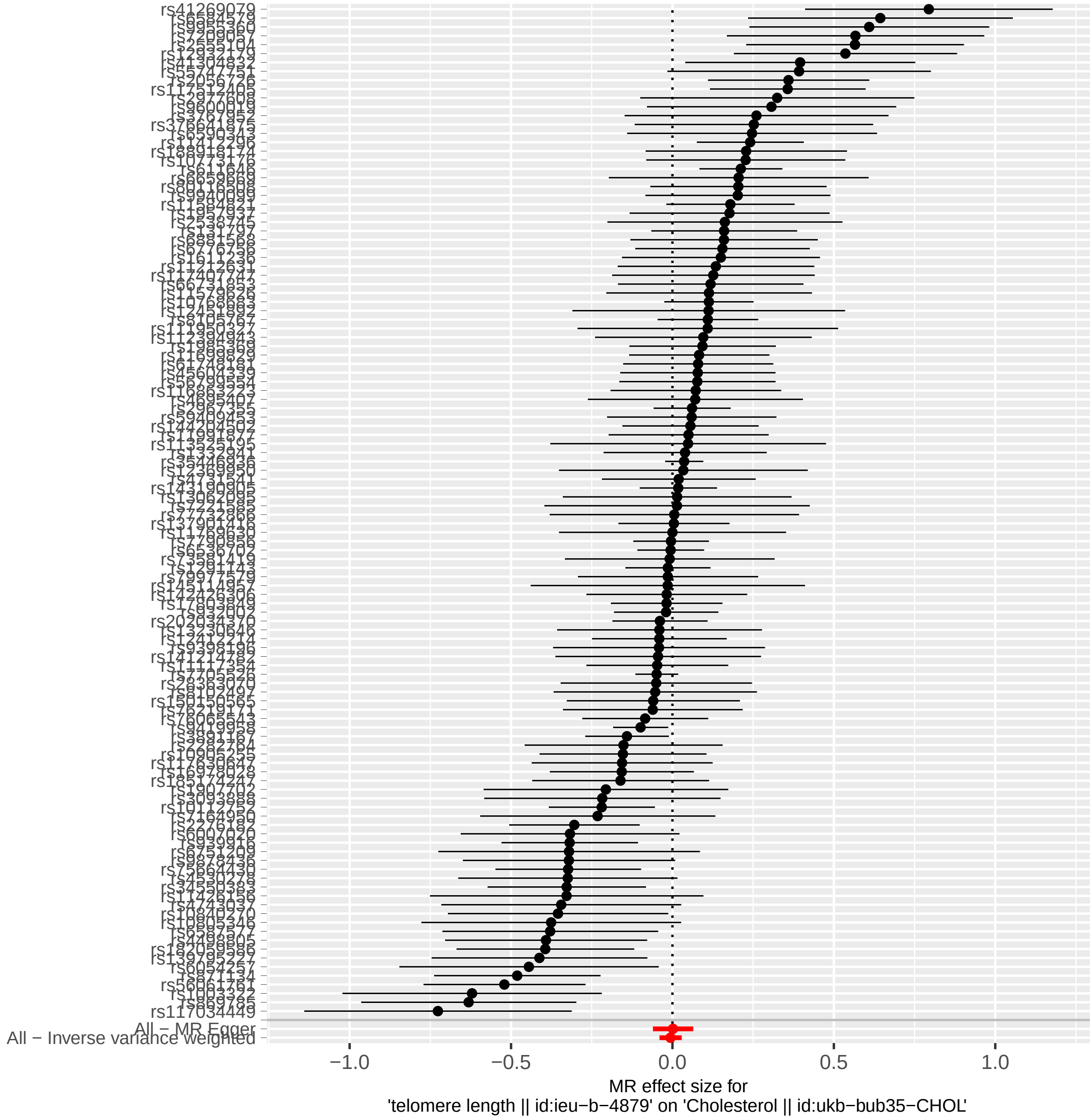

rs41269079
rs117034449
All − MR Egger
All − Inverse variance weighted
−1.0
−0.5
0.0
0.5
1.0
MR effect size for
'telomere length || id:ieu−b−4879' on 'Cholesterol || id:ukb−bub35−CHOL'

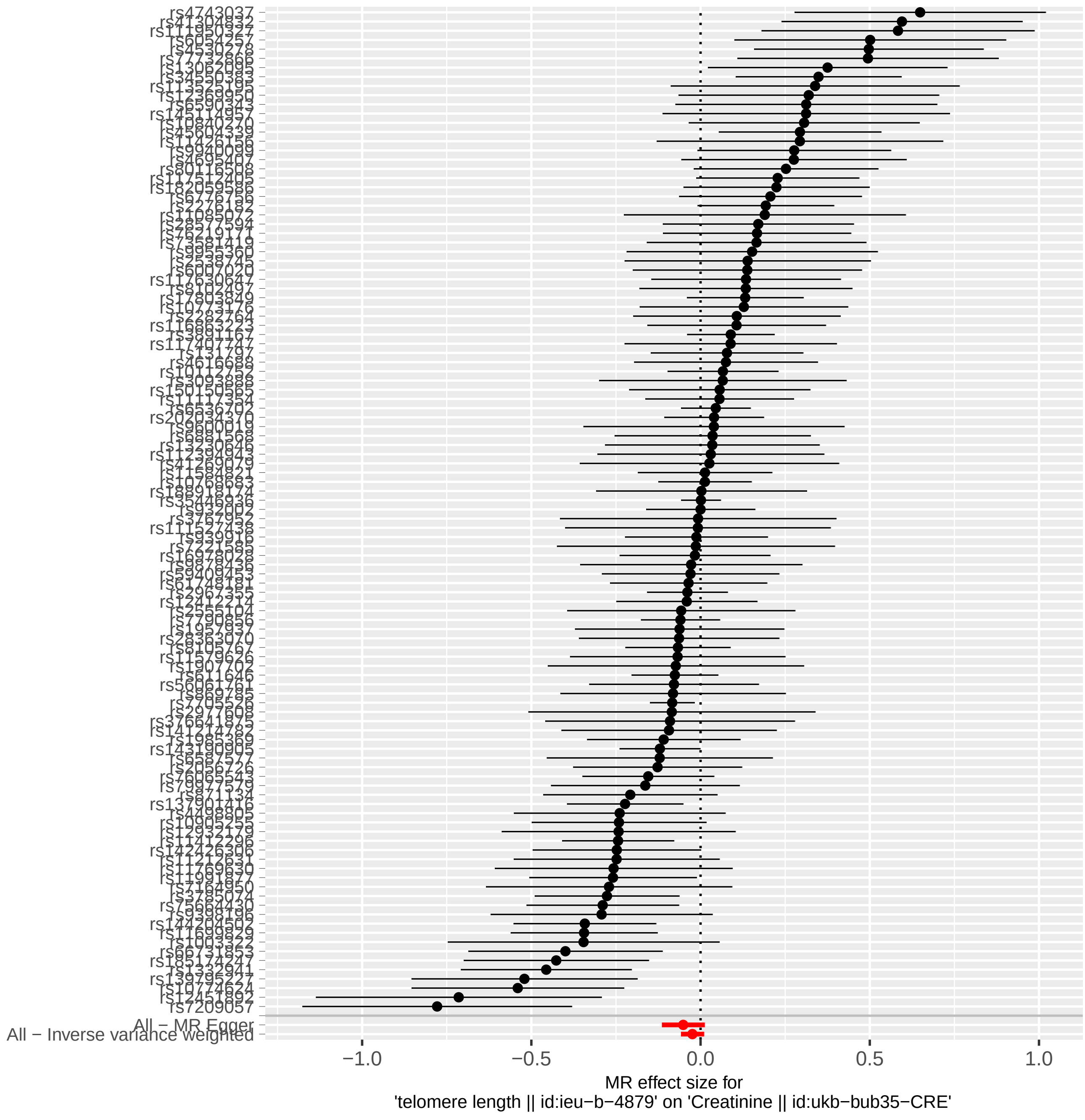

rs4743037
rs7209057
All − MR Egger
All − Inverse variance weighted
−1.0
−0.5
0.0
0.5
1.0
MR effect size for
'telomere length || id:ieu−b−4879' on 'Creatinine || id:ukb−bub35−CRE'

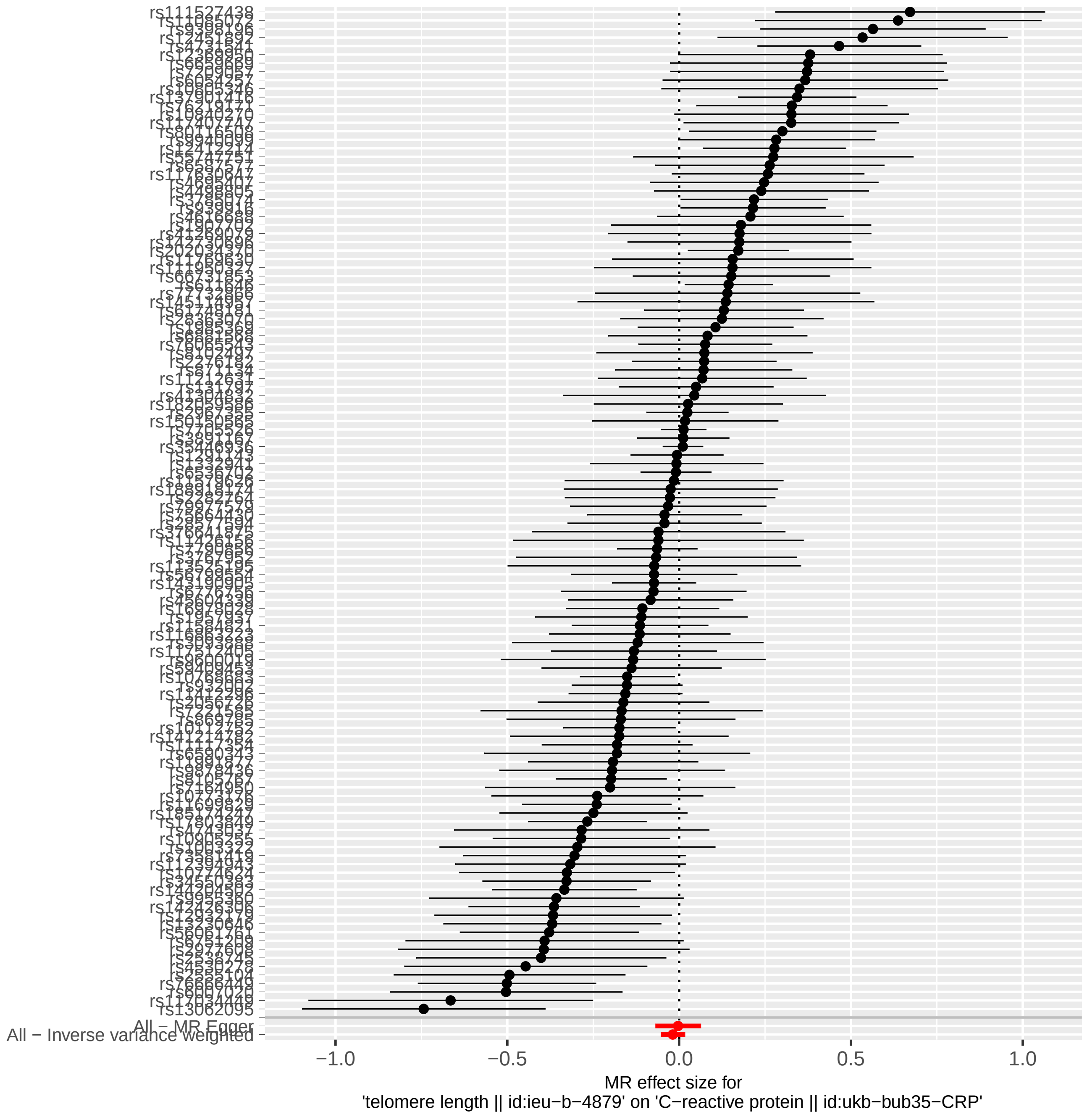

rs111527438
rs13062095
All − MR Egger
All − Inverse variance weighted
−1.0
−0.5
0.0
0.5
1.0
MR effect size for
'telomere length || id:ieu−b−4879' on 'C−reactive protein || id:ukb−bub35−CRP'

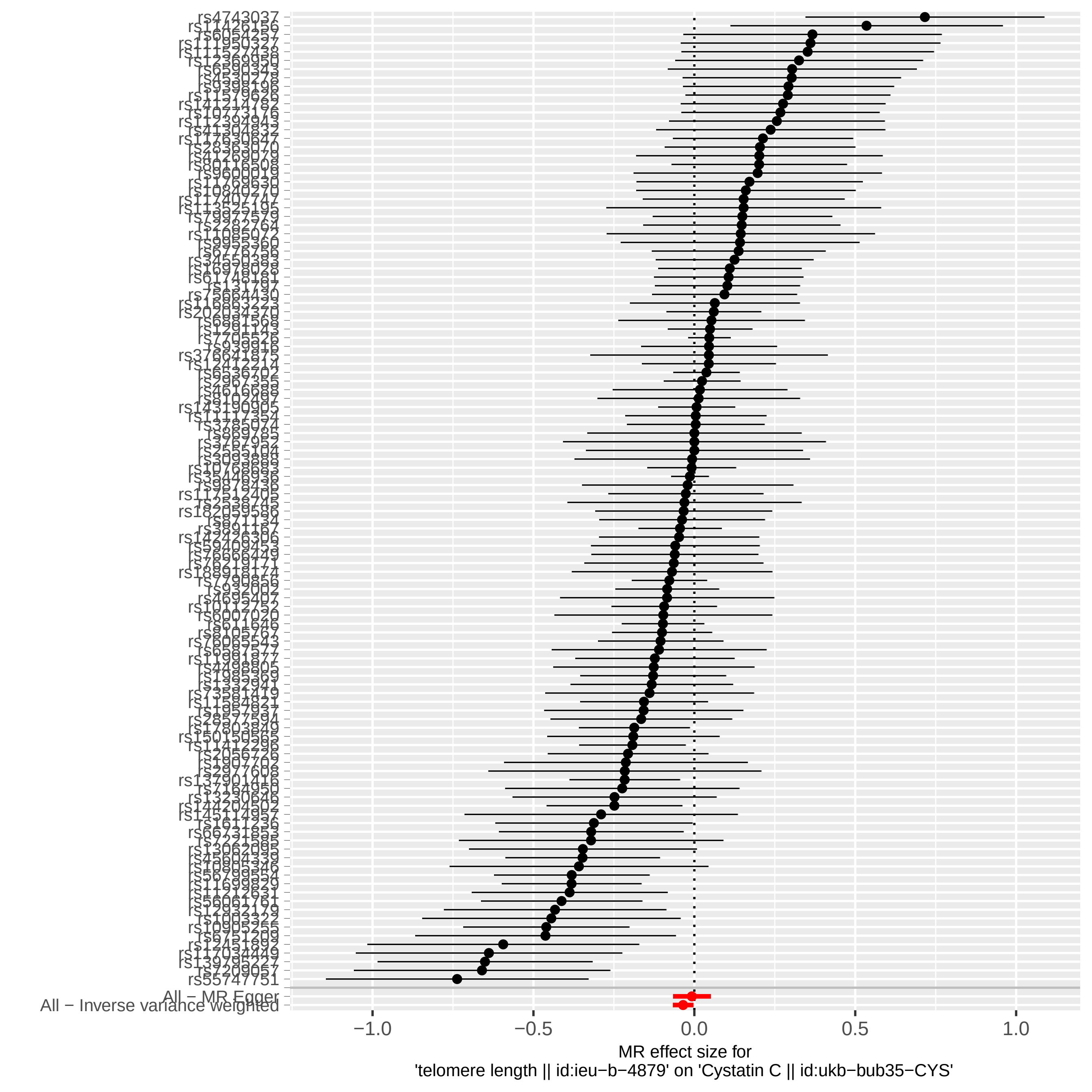
rs4743037
rs55747751
All − MR Egger
All − Inverse variance weighted
−1.0
−0.5
0.0
0.5
1.0
MR effect size for
'telomere length || id:ieu−b−4879' on 'Cystatin C || id:ukb−bub35−CYS'

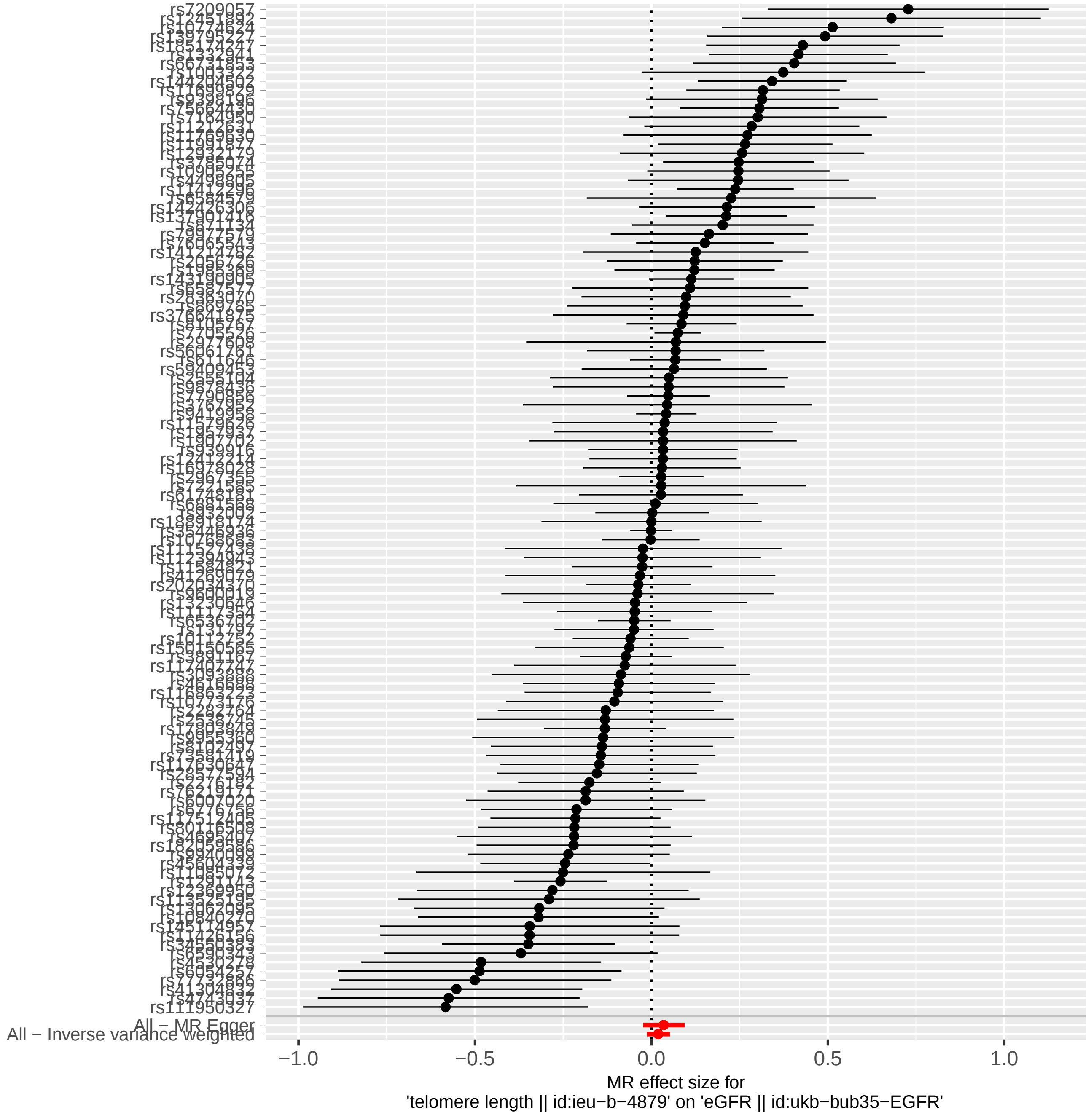
rs7209057
rs111950327
All − MR Egger
All − Inverse variance weighted
−1.0
−0.5
0.0
0.5
1.0
MR effect size for
'telomere length || id:ieu-b-4879' on 'eGFR || id:ukb-bub35-EGFR'

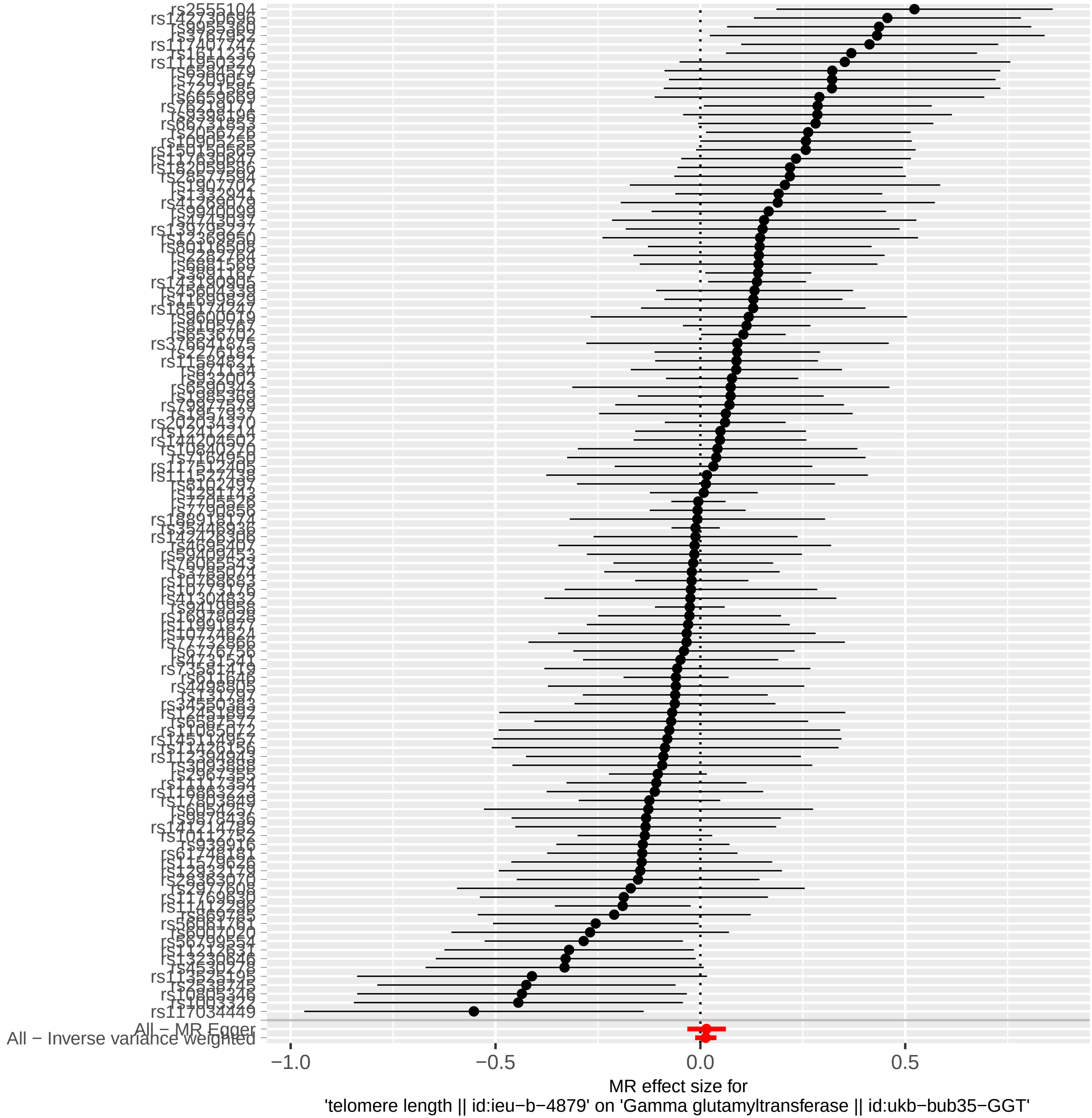

rs2555104
rs117034449
All − MR Egger
All − Inverse variance weighted
−1.0
−0.5
0.0
0.5
MR effect size for
'telomere length || id:ieu−b−4879' on 'Gamma glutamyltransferase || id:ukb−bub35−GGT'

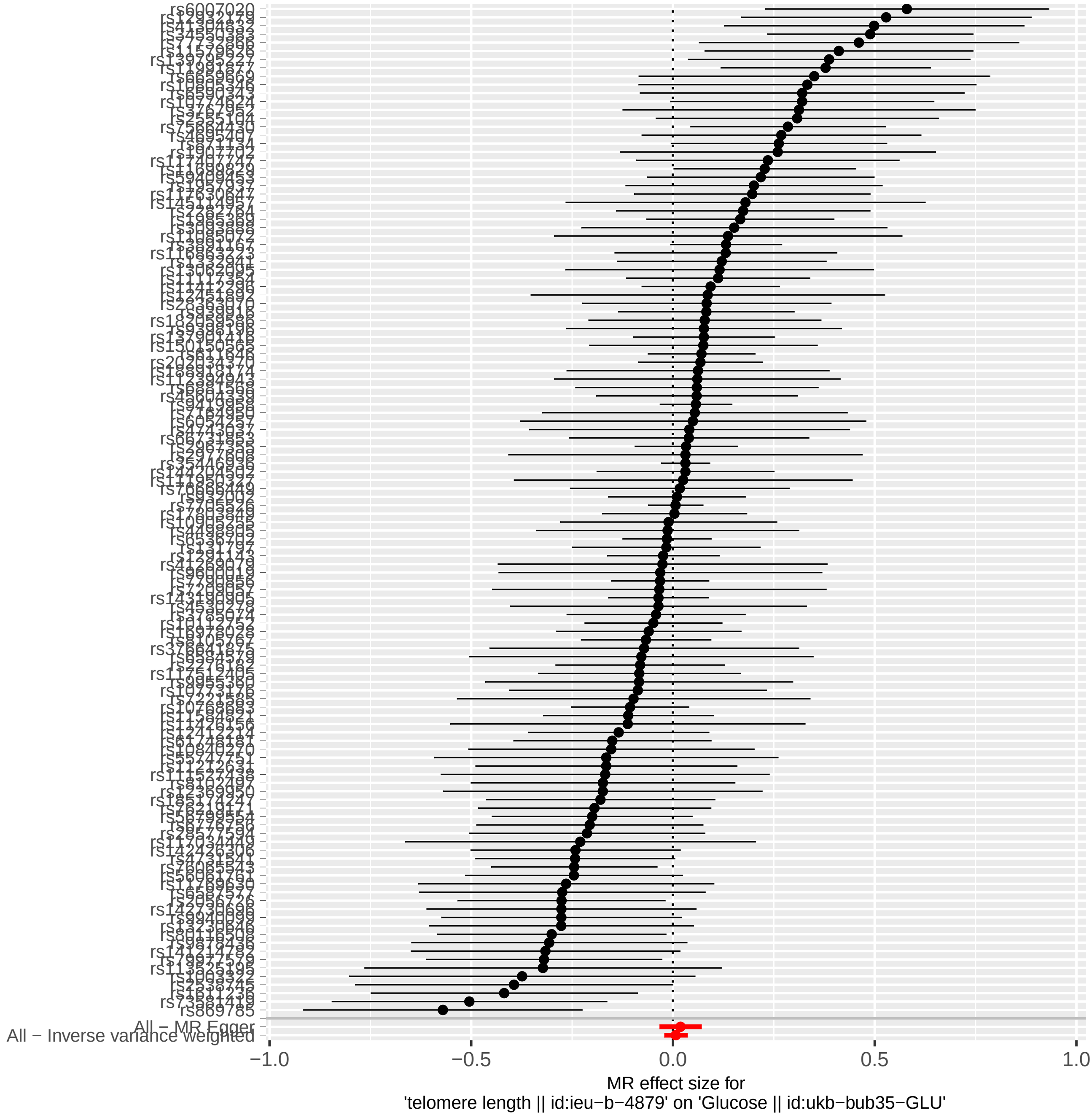

rs6007020
rs869785
All − MR Egger
All − Inverse variance weighted
−1.0
−0.5
0.0
0.5
1.0
MR effect size for
'telomere length || id:ieu−b−4879' on 'Glucose || id:ukb−bub35−GLU'

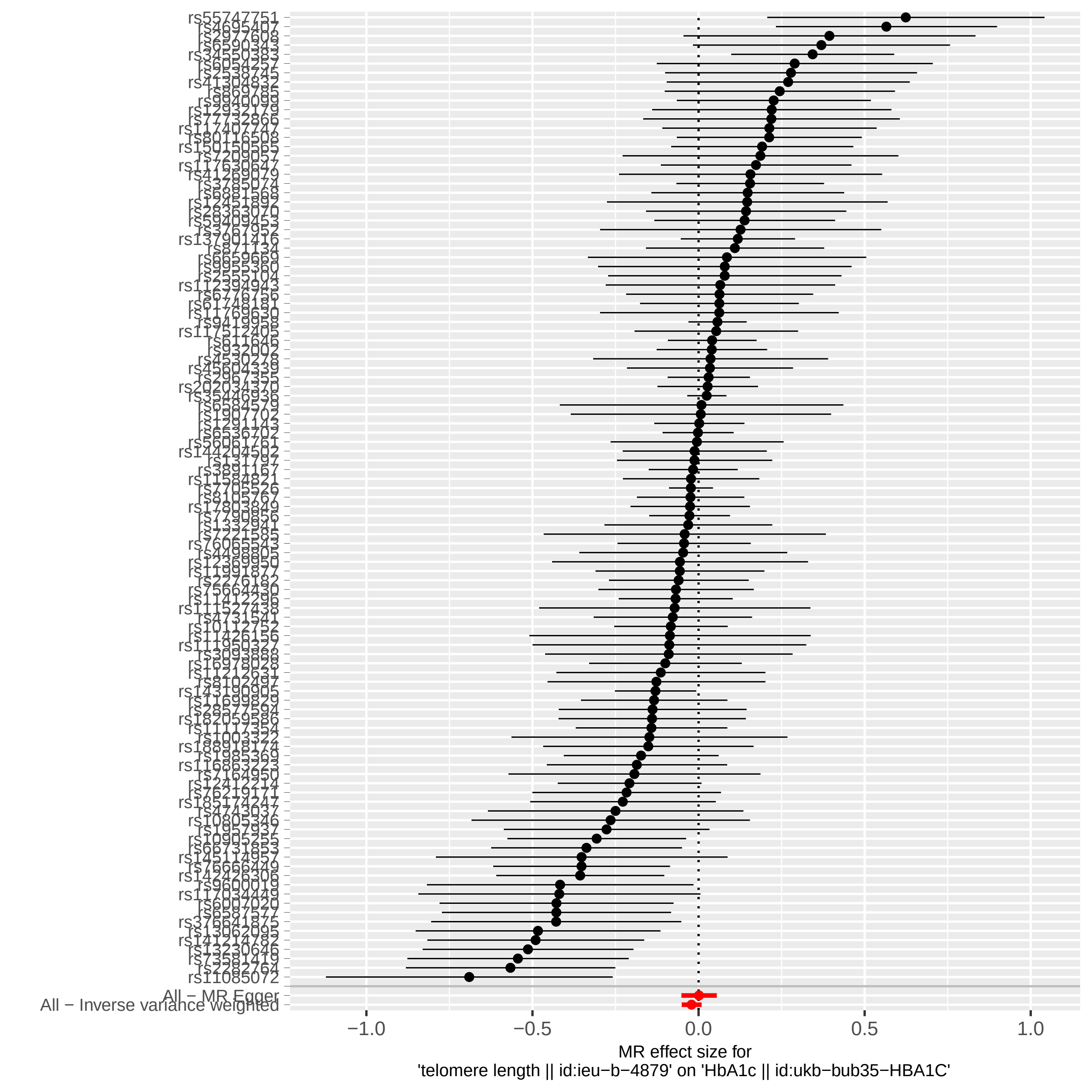
rs55747751
rs11085072
All − MR Egger
All − Inverse variance weighted
−1.0
−0.5
0.0
0.5
1.0
MR effect size for
'telomere length || id:ieu−b−4879' on 'HbA1c || id:ukb−bub35−HBA1C'

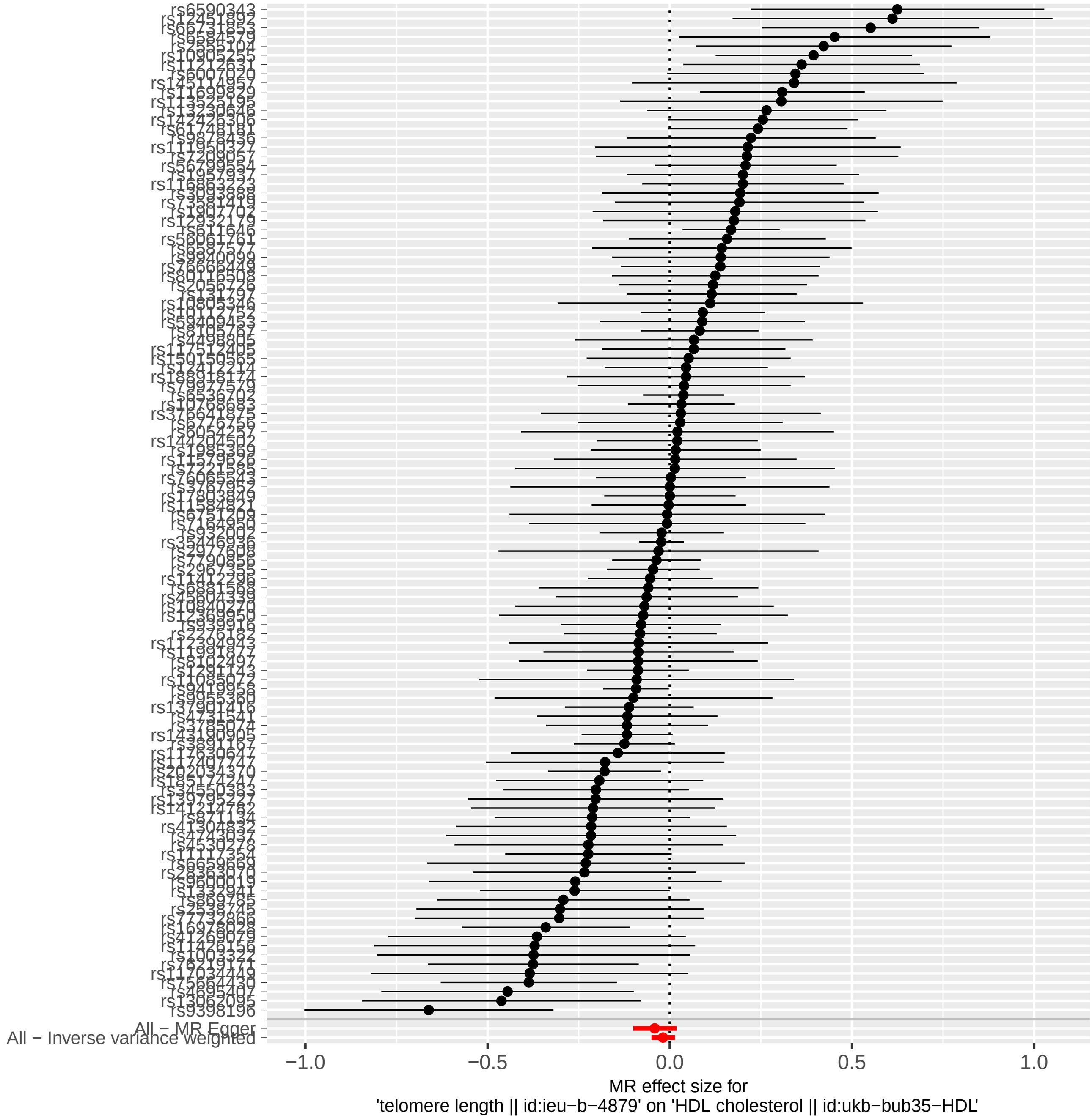
rs6590343
rs9398196
All − MR Egger
All − Inverse variance weighted
−1.0
−0.5
0.0
0.5
1.0
MR effect size for
'telomere length || id:ieu−b−4879' on 'HDL cholesterol || id:ukb−bub35−HDL'

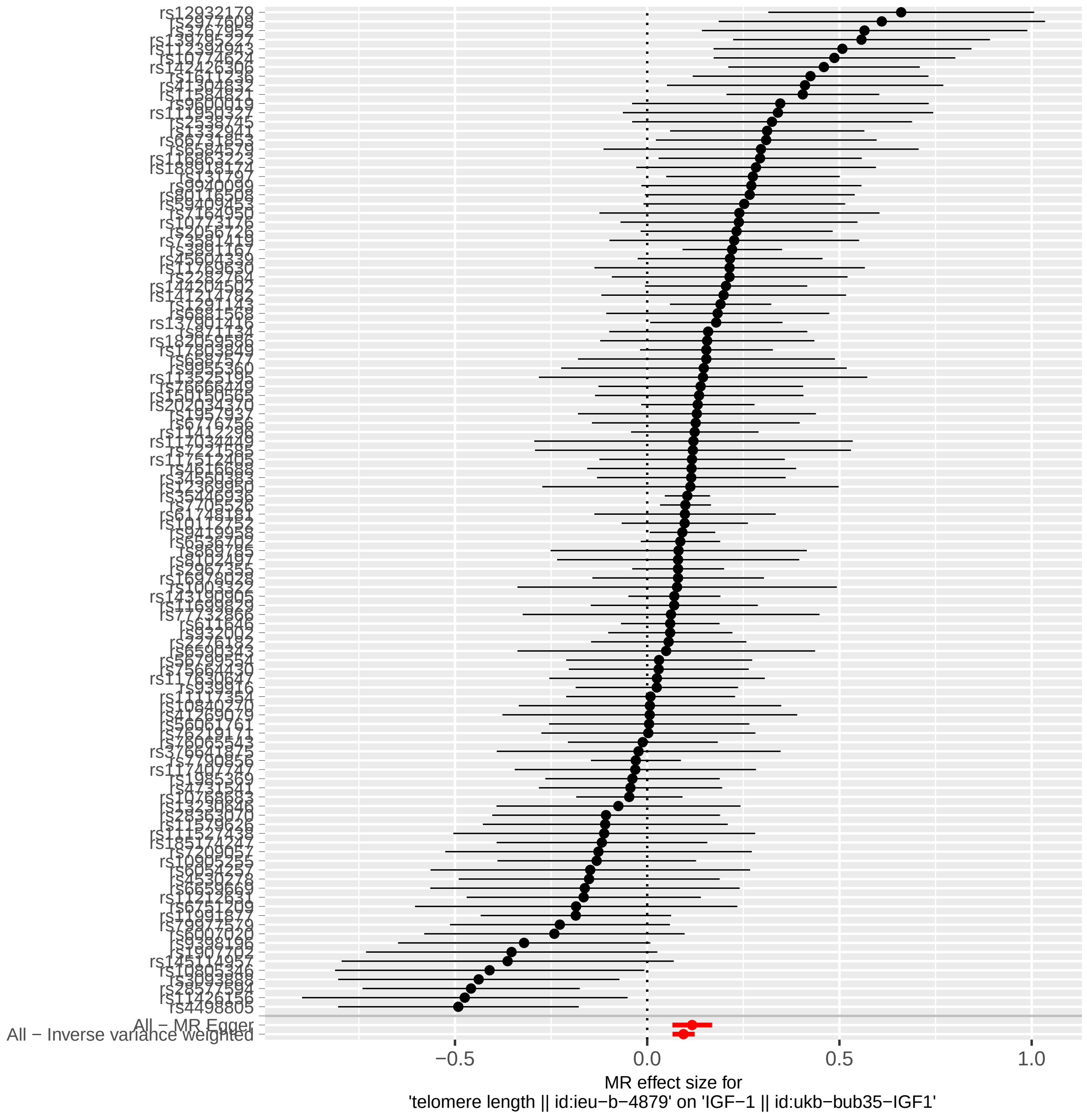

rs12932179
rs4498805
All − MR Egger
All − Inverse variance weighted
−0.5
0.0
0.5
1.0
MR effect size for
'telomere length || id:ieu−b−4879' on 'IGF−1 || id:ukb−bub35−IGF1'

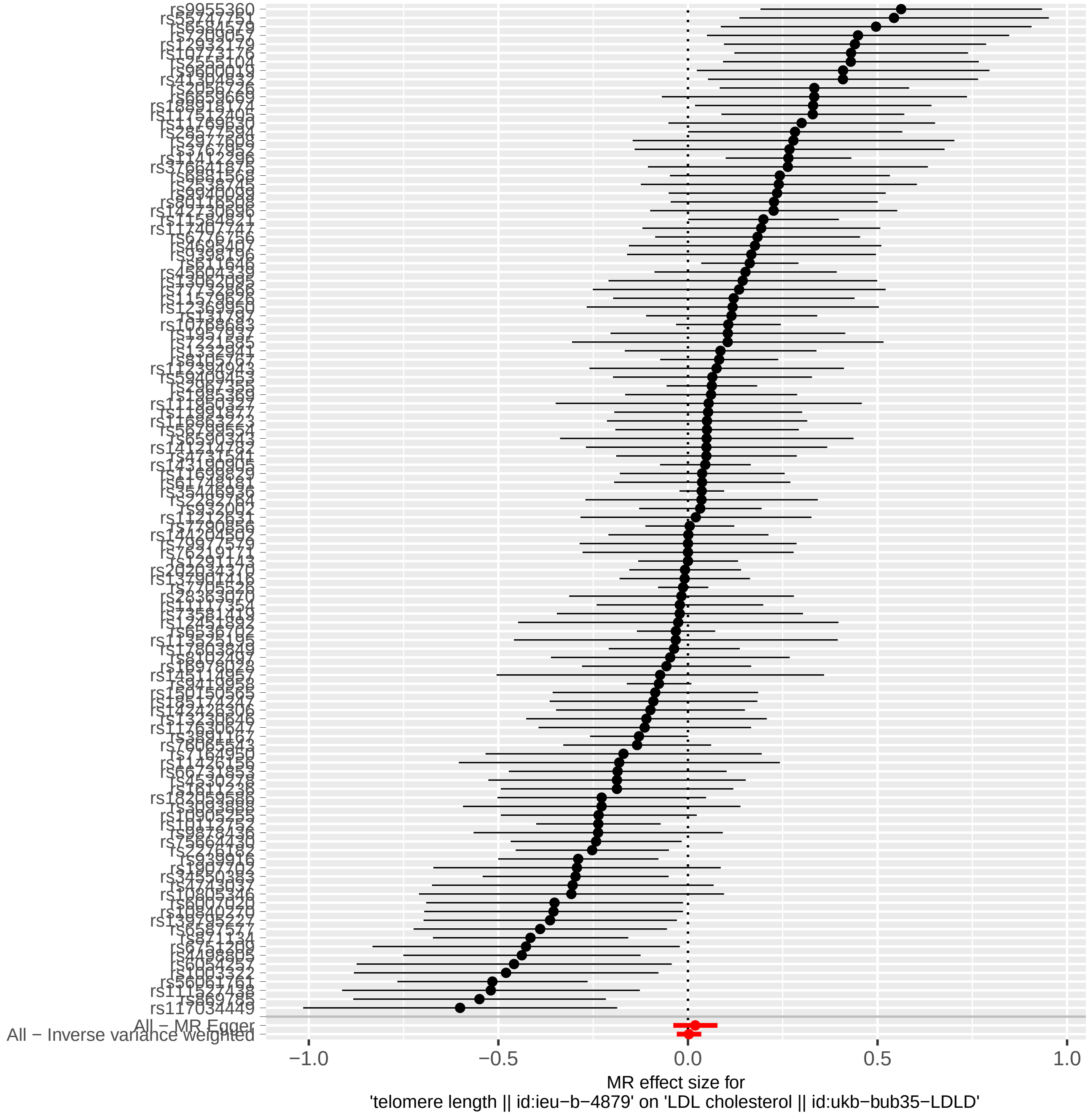

rs9955360
rs11703449
All − MR Egger
All − Inverse variance weighted
−1.0
−0.5
0.0
0.5
1.0
MR effect size for
'telomere length || id:ieu−b−4879' on 'LDL cholesterol || id:ukb−bub35−LDLD'

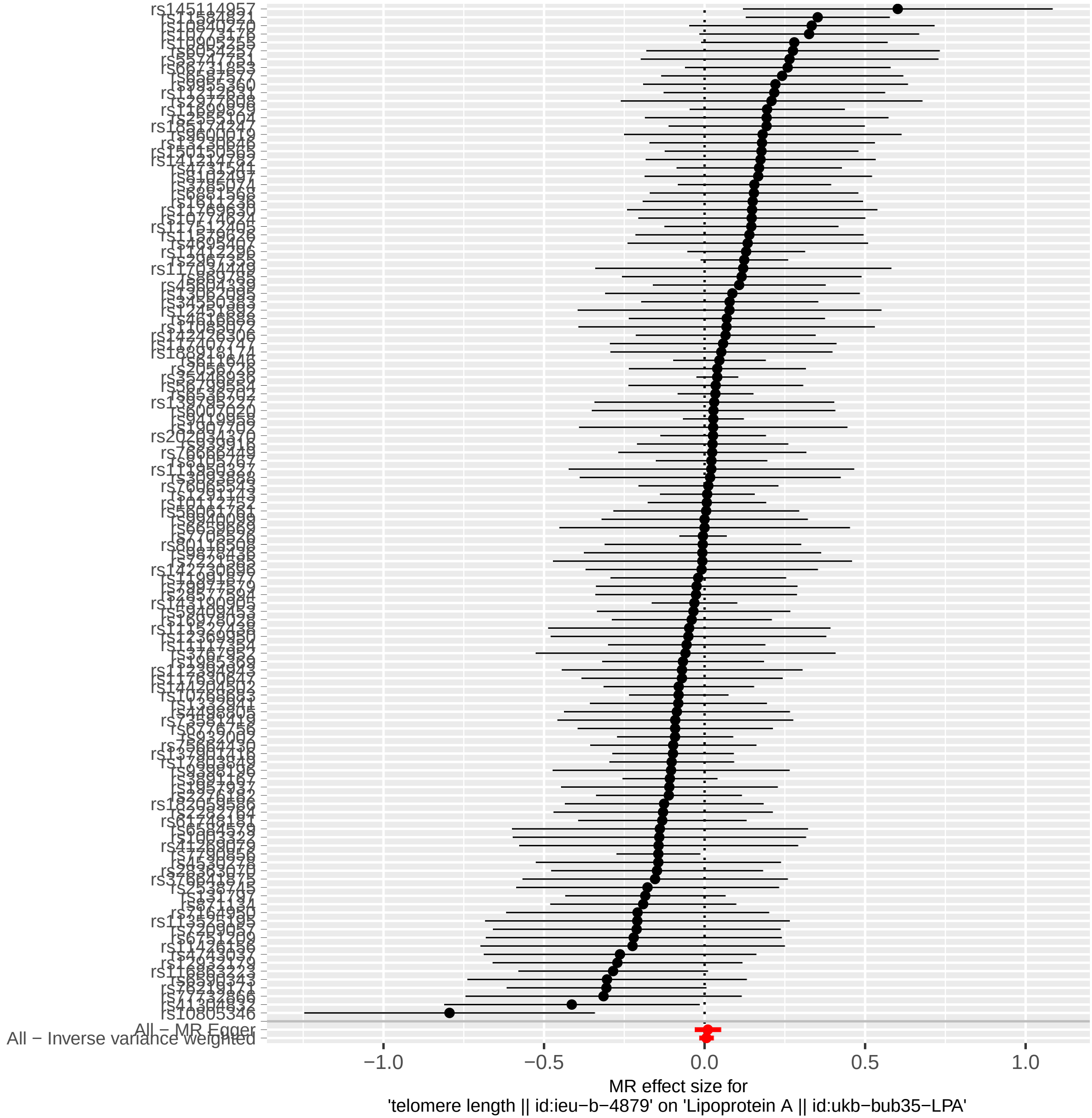
rs145114957
rs10805346
All − MR Egger
All − Inverse variance weighted
−1.0
−0.5
0.0
0.5
1.0
MR effect size for
'telomere length || id:ieu−b−4879' on 'Lipoprotein A || id:ukb−bub35−LPA'

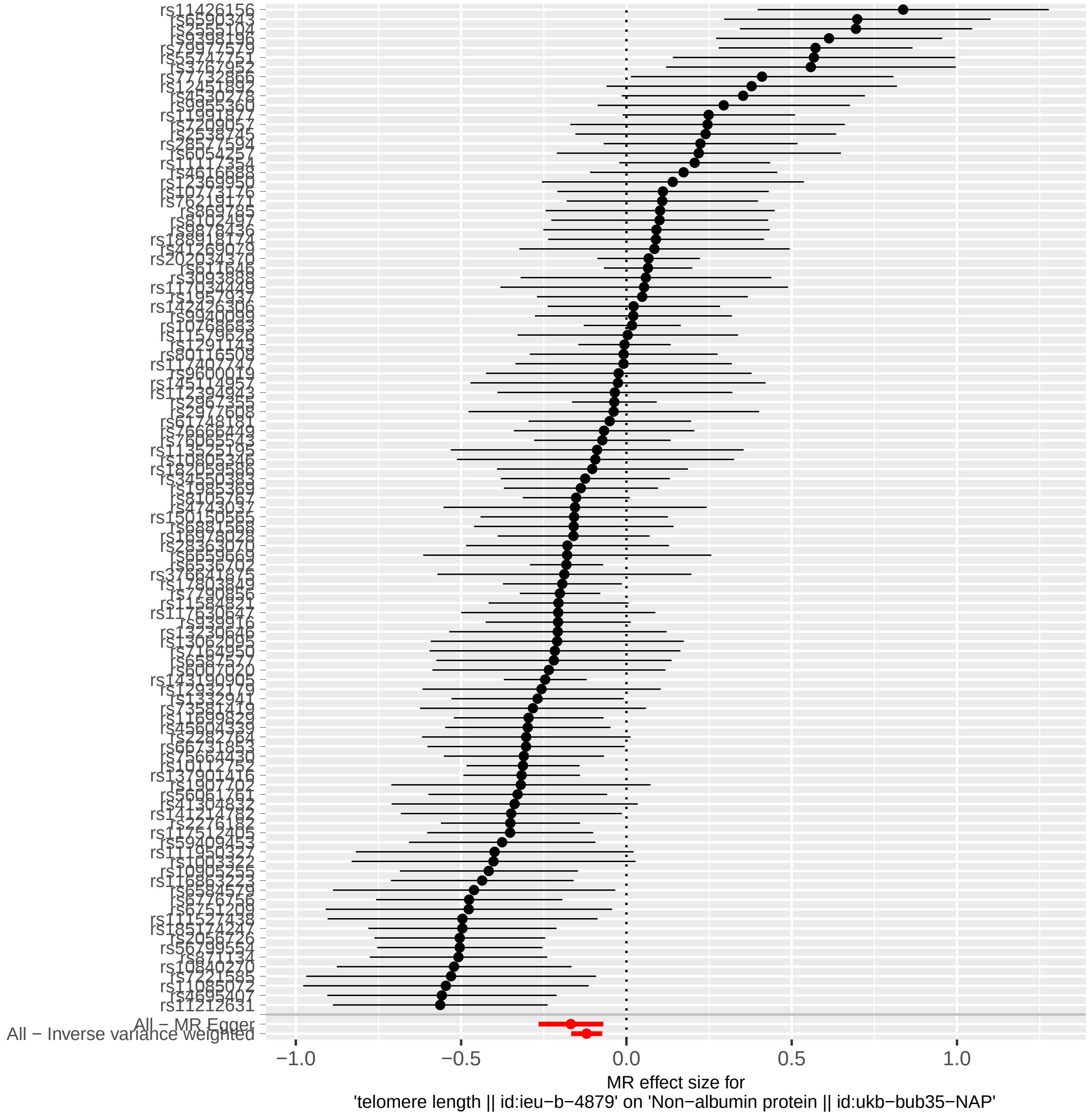

rs11426156
rs11212631
All − MR Egger
All − Inverse variance weighted
−1.0
−0.5
0.0
0.5
1.0
MR effect size for
'telomere length || id:ieu−b−4879' on 'Non−albumin protein || id:ukb−bub35−NAP'

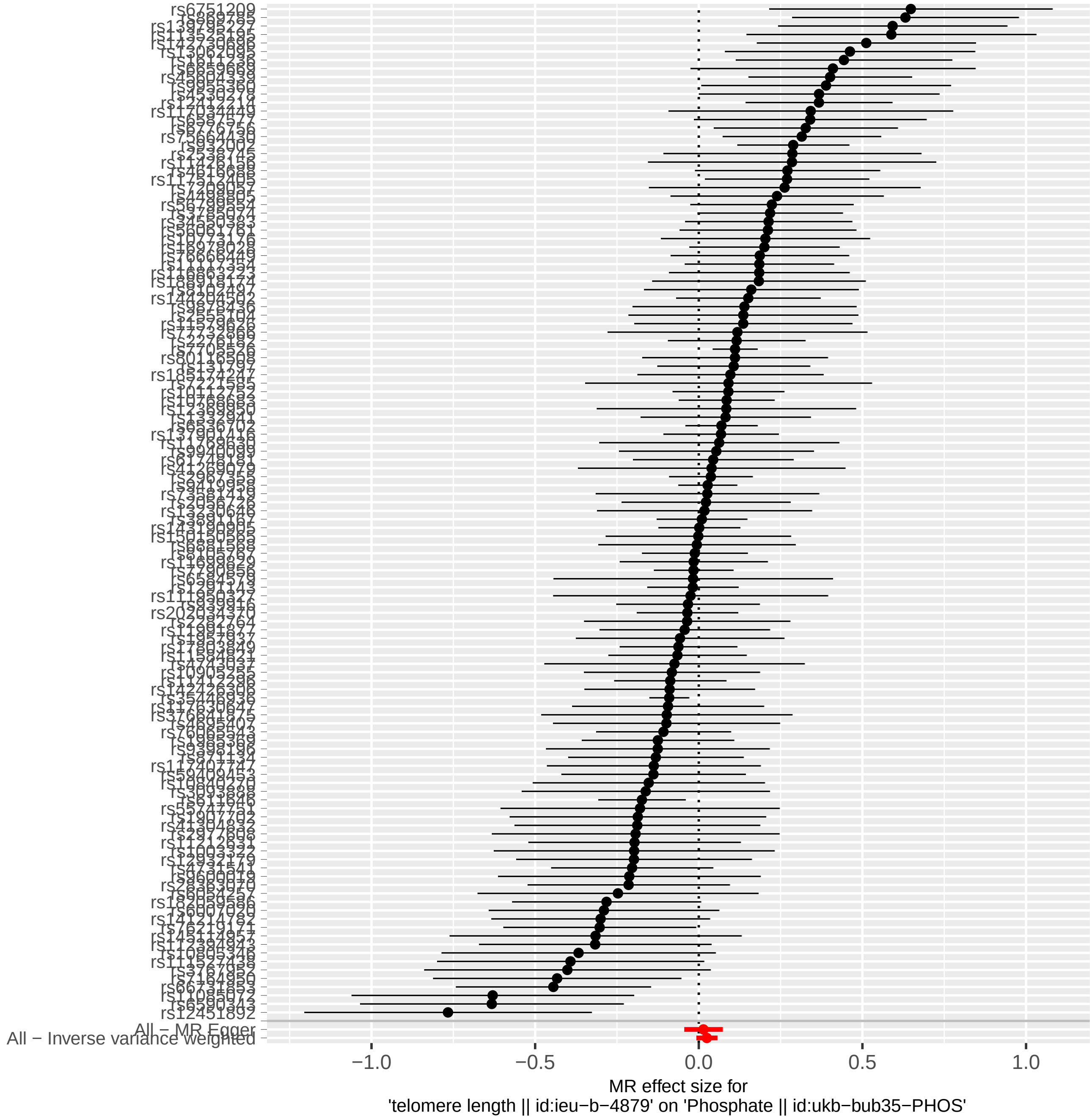

rs6751209
rs12451892
All − MR Egger
All − Inverse variance weighted
−1.0
−0.5
0.0
0.5
1.0
MR effect size for
'telomere length || id:ieu−b−4879' on 'Phosphate || id:ukb−bub35−PHOS'

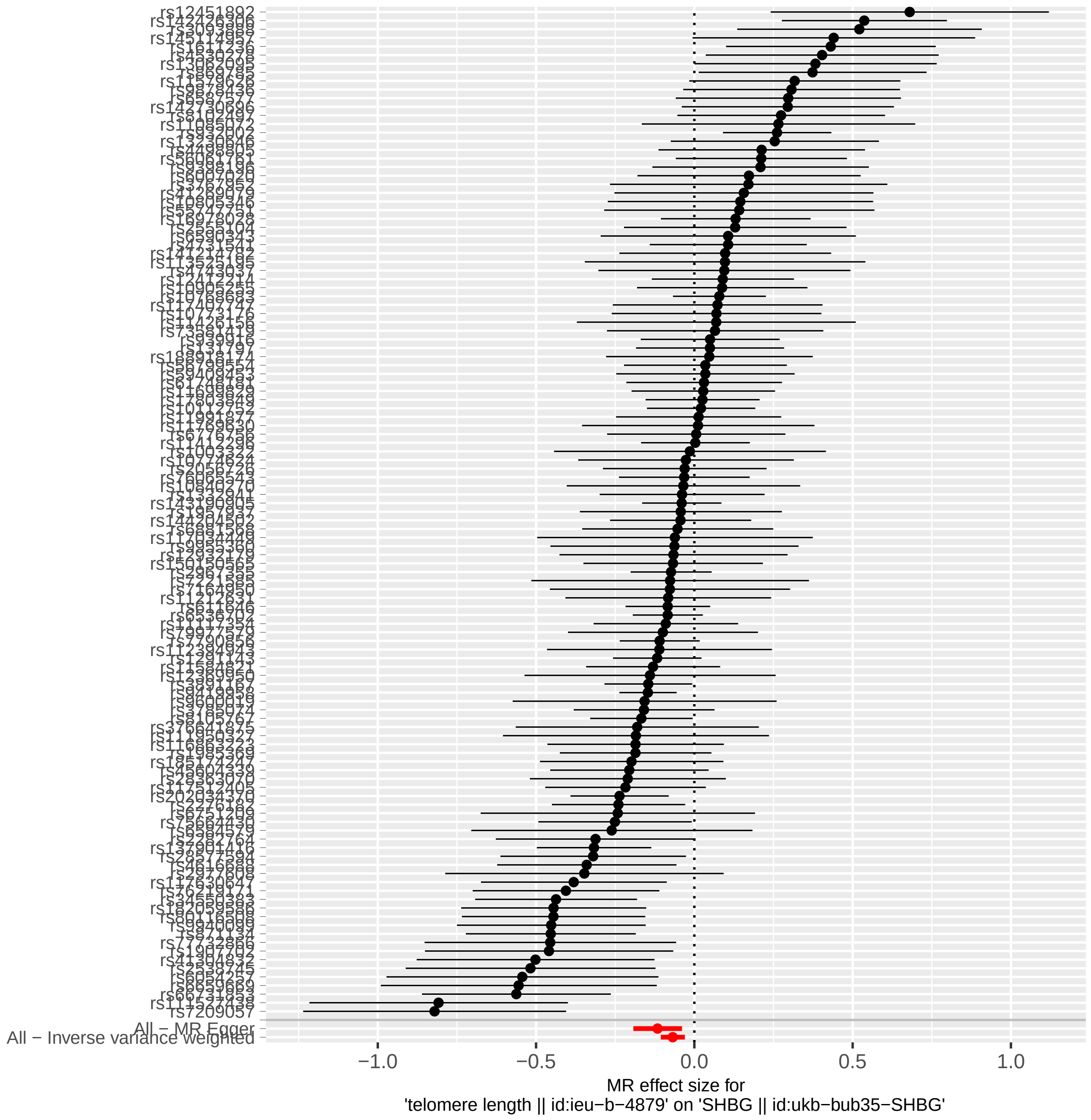

rs12451892
rs7209057
All − MR Egger
All − Inverse variance weighted
−1.0
−0.5
0.0
0.5
1.0
MR effect size for
'telomere length || id:ieu−b−4879' on 'SHBG || id:ukb−bub35−SHBG'

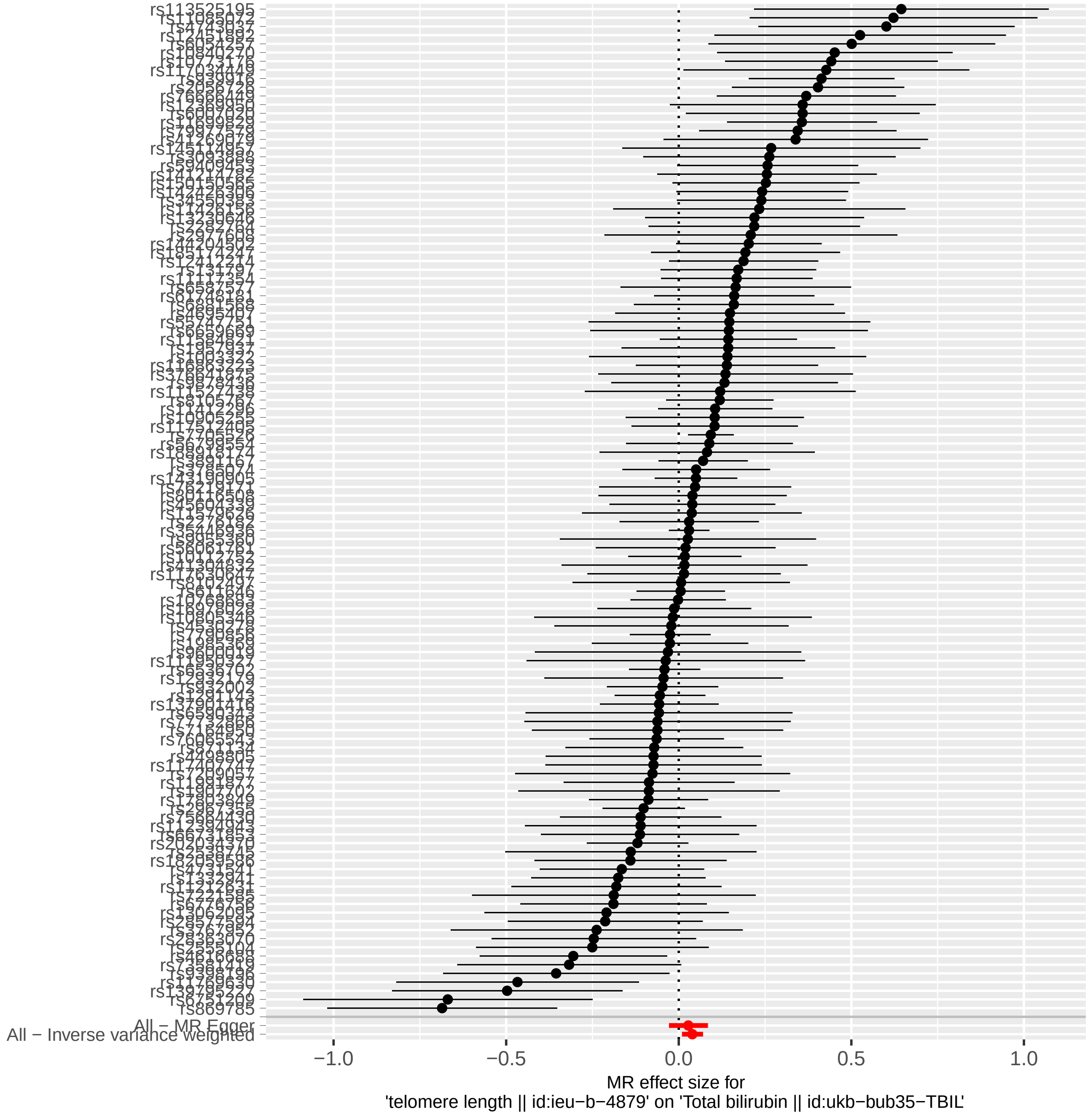
rs113525195
rs869785
All − MR Egger
All − Inverse variance weighted
−1.0
−0.5
0.0
0.5
1.0
MR effect size for
'telomere length || id:ieu−b−4879' on 'Total bilirubin || id:ukb−bub35−TBIL'

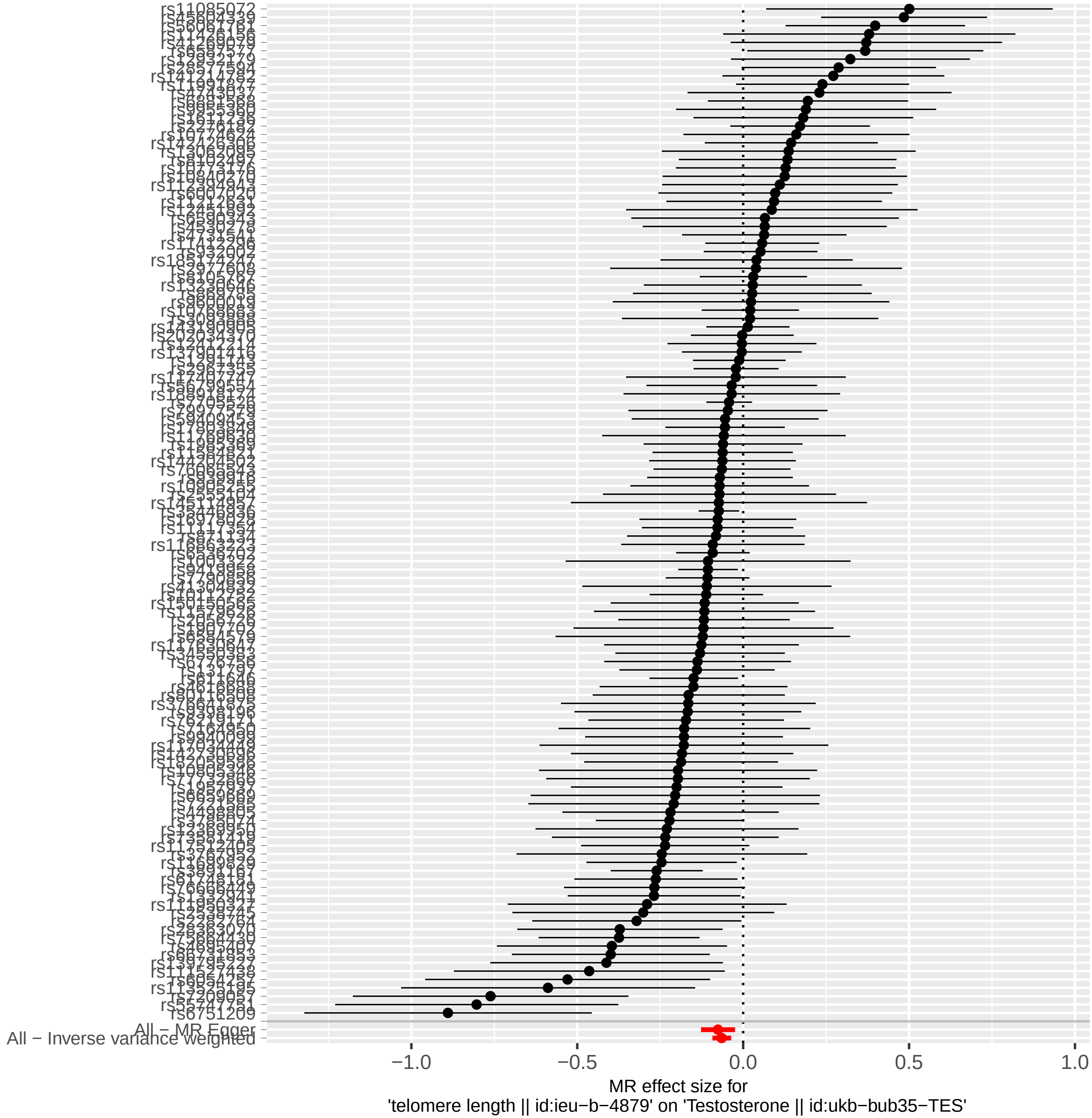

rs11085072
rs6751209
All − MR Egger
All − Inverse variance weighted
−1.0
−0.5
0.0
0.5
1.0
MR effect size for
'telomere length || id:ieu−b−4879' on 'Testosterone || id:ukb−bub35−TES'

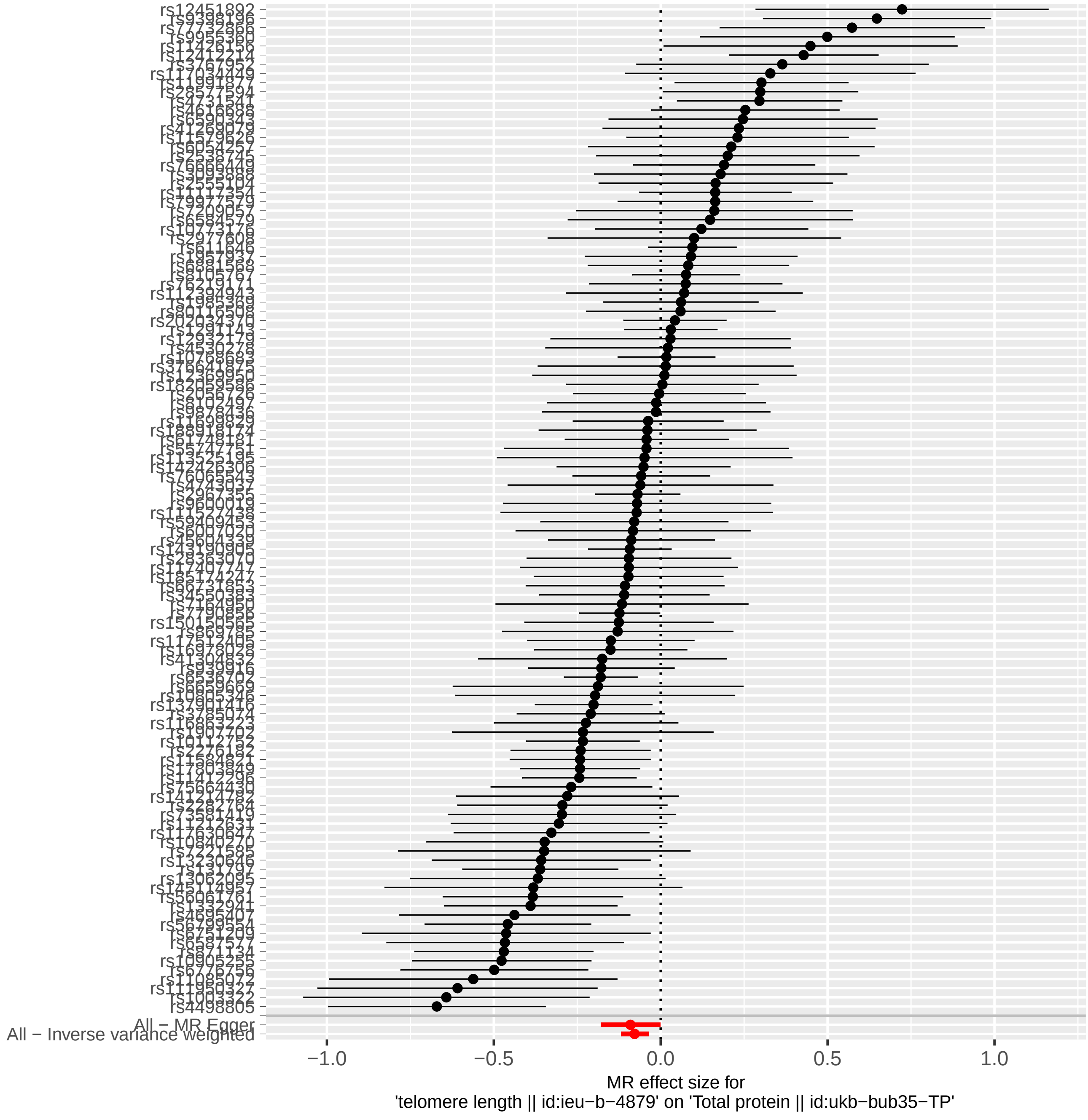

rs12451892
rs4498805
All − MR Egger
All − Inverse variance weighted
−1.0
−0.5
0.0
0.5
1.0
MR effect size for
'telomere length || id:ieu−b−4879' on 'Total protein || id:ukb−bub35−TP'

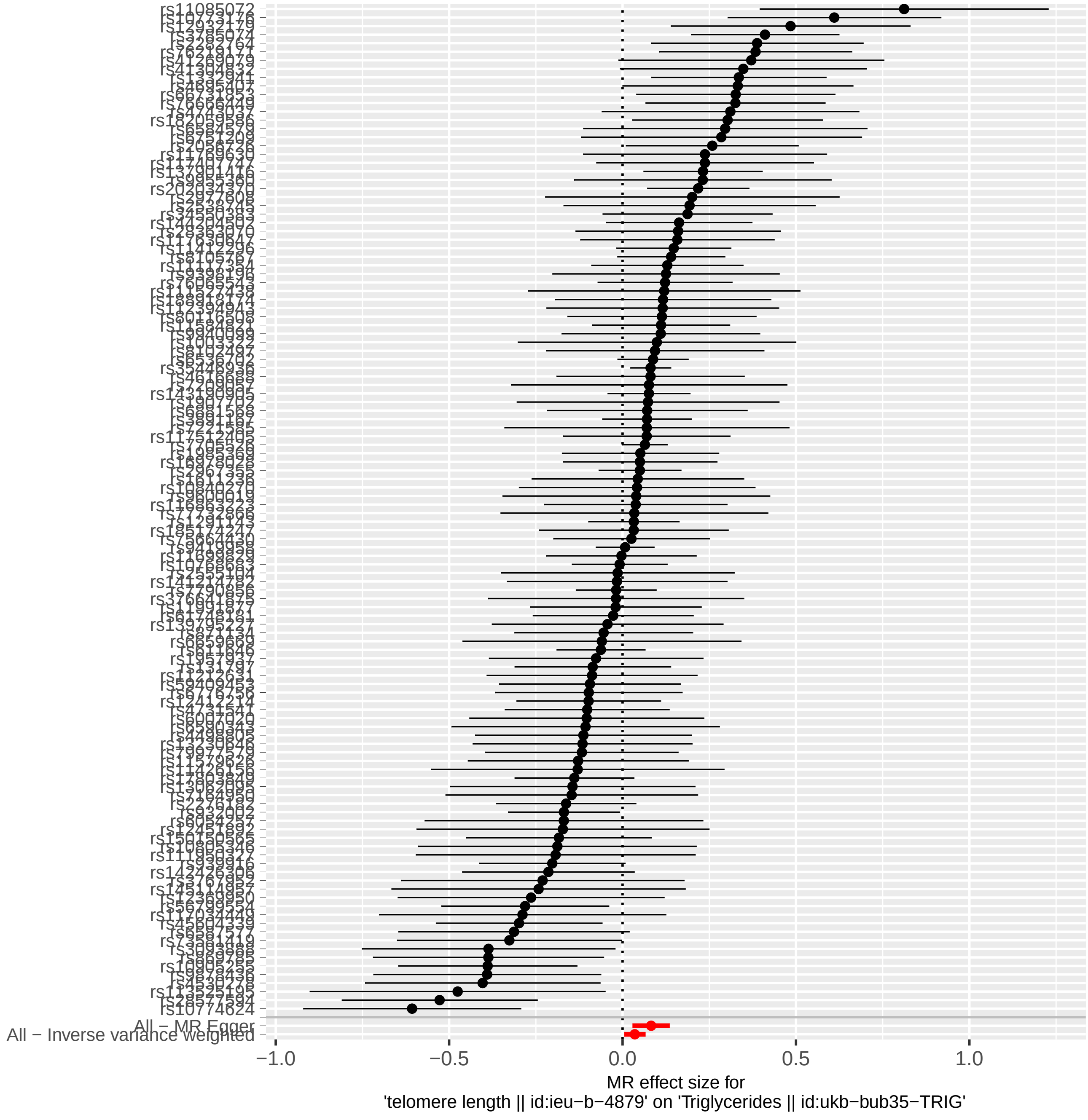
rs11085072
rs10774624
All − MR Egger
All − Inverse variance weighted
−1.0
−0.5
0.0
0.5
1.0
MR effect size for
'telomere length || id:ieu−b−4879' on 'Triglycerides || id:ukb−bub35−TRIG'

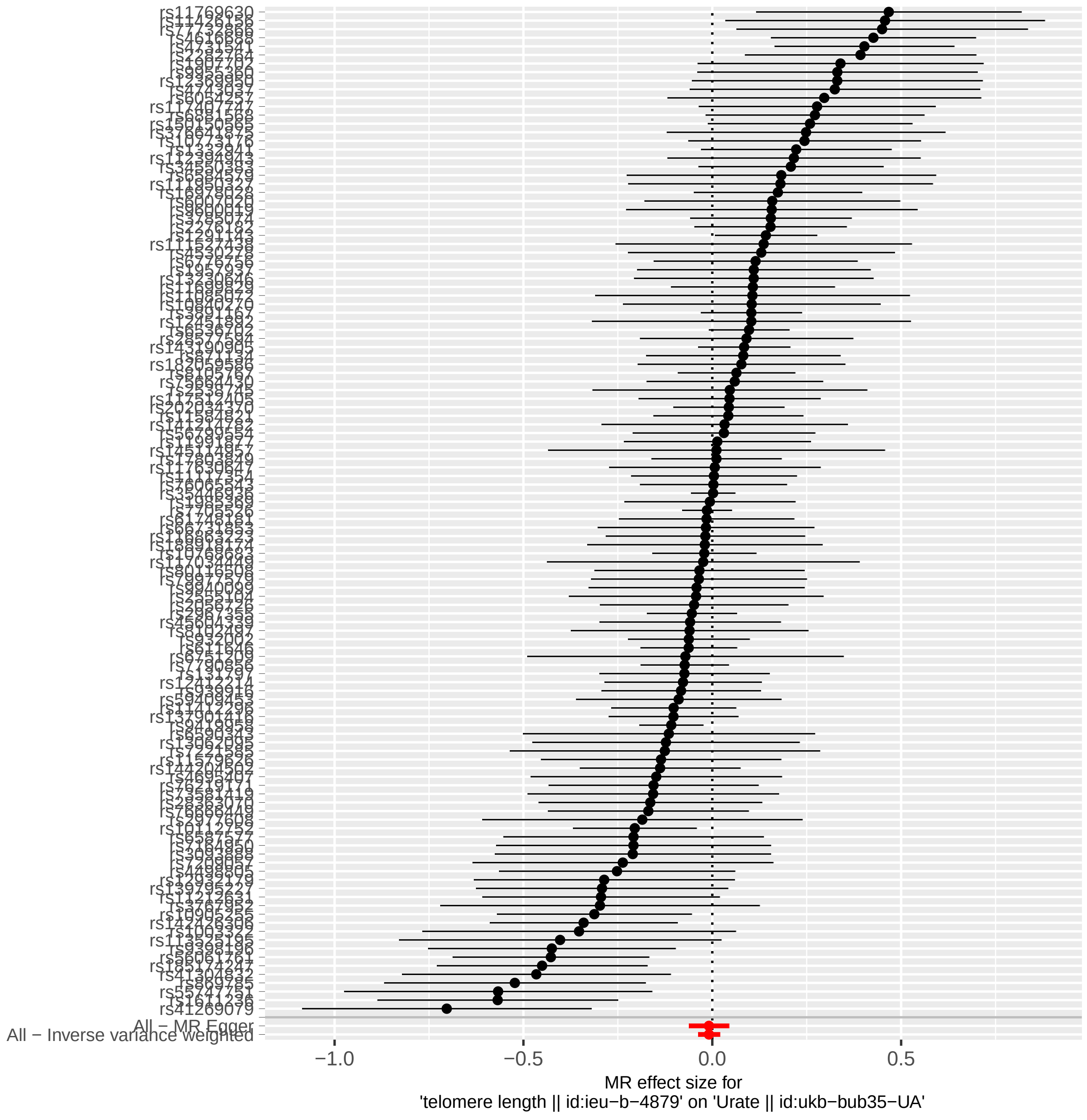

rs11769630
rs41269079
All − MR Egger
All − Inverse variance weighted
−1.0
−0.5
0.0
0.5
MR effect size for
'telomere length || id:ieu−b−4879' on 'Urate || id:ukb−bub35−UA'

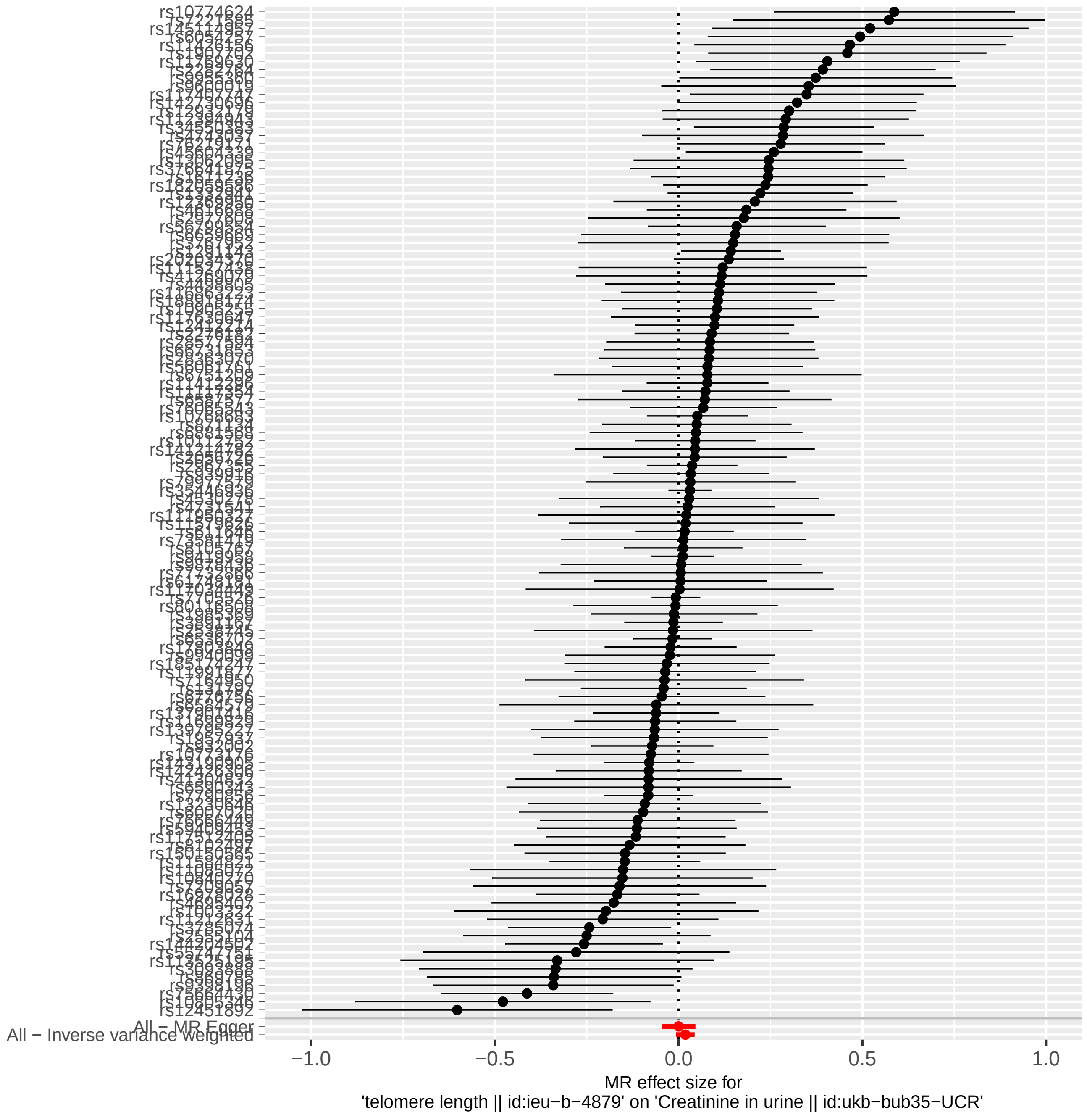
rs10774624
rs12451892
All − MR Egger
All − Inverse variance weighted
−1.0
−0.5
0.0
0.5
1.0
MR effect size for
'telomere length || id:ieu−b−4879' on 'Creatinine in urine || id:ukb−bub35−UCR'

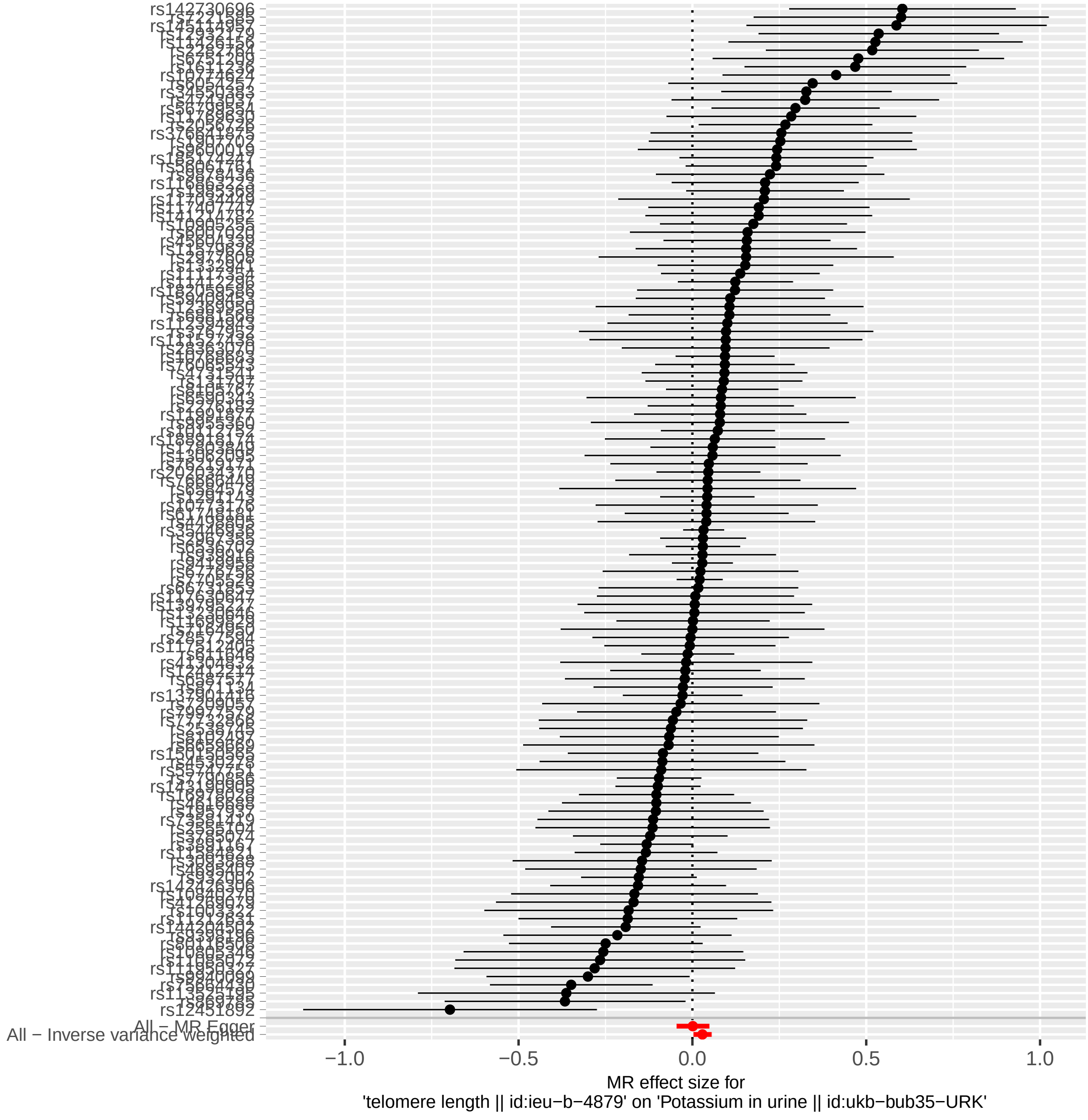
rs142730696
rs12451892
All − MR Egger
All − Inverse variance weighted
−1.0
−0.5
0.0
0.5
1.0
MR effect size for
'telomere length || id:ieu−b−4879' on 'Potassium in urine || id:ukb−bub35−URK'

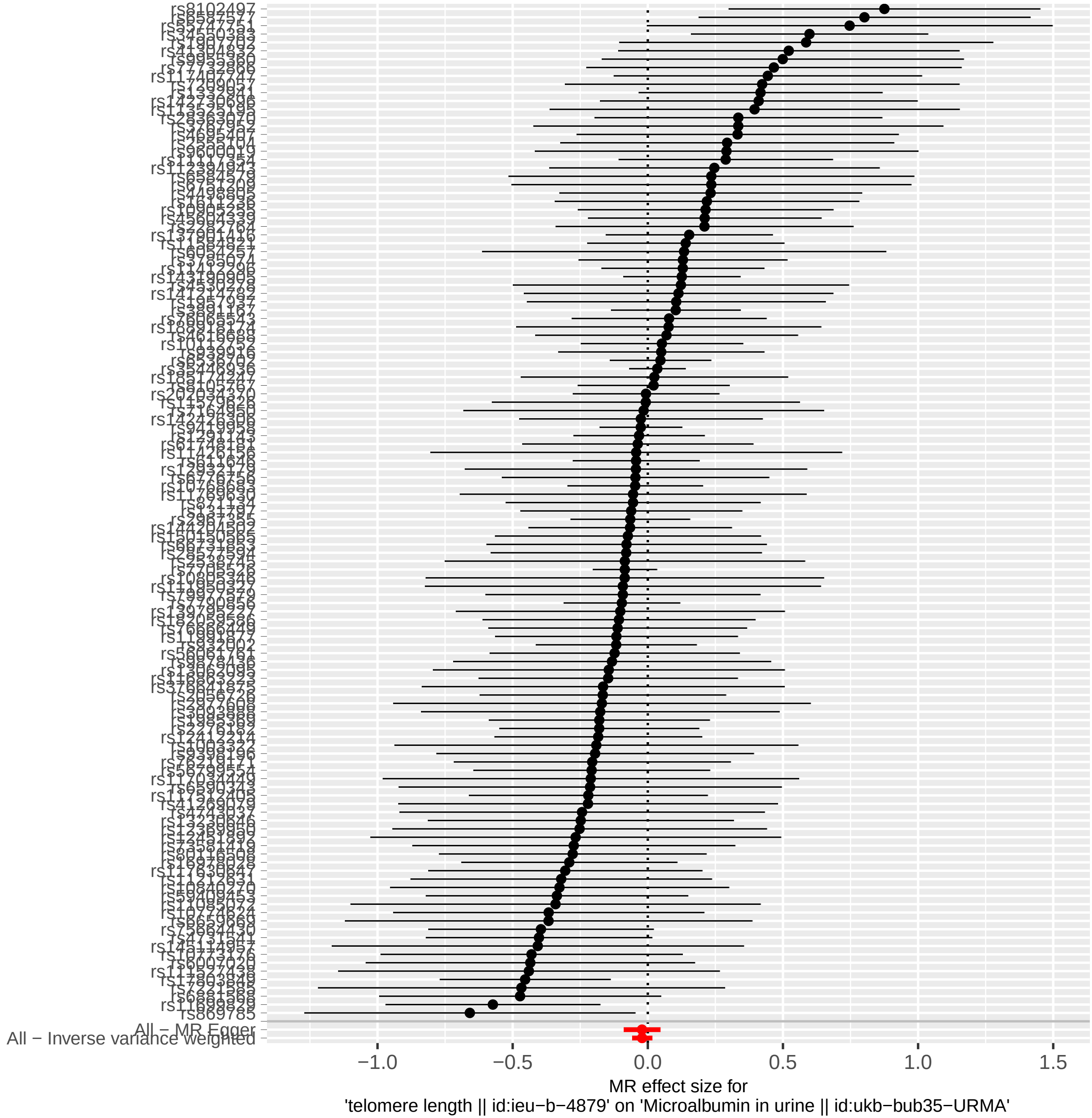
rs8102497
rs869785
All − MR Egger
All − Inverse variance weighted
−1.0
−0.5
0.0
0.5
1.0
1.5
MR effect size for
'telomere length || id:ieu−b−4879' on 'Microalbumin in urine || id:ukb−bub35−URMA'

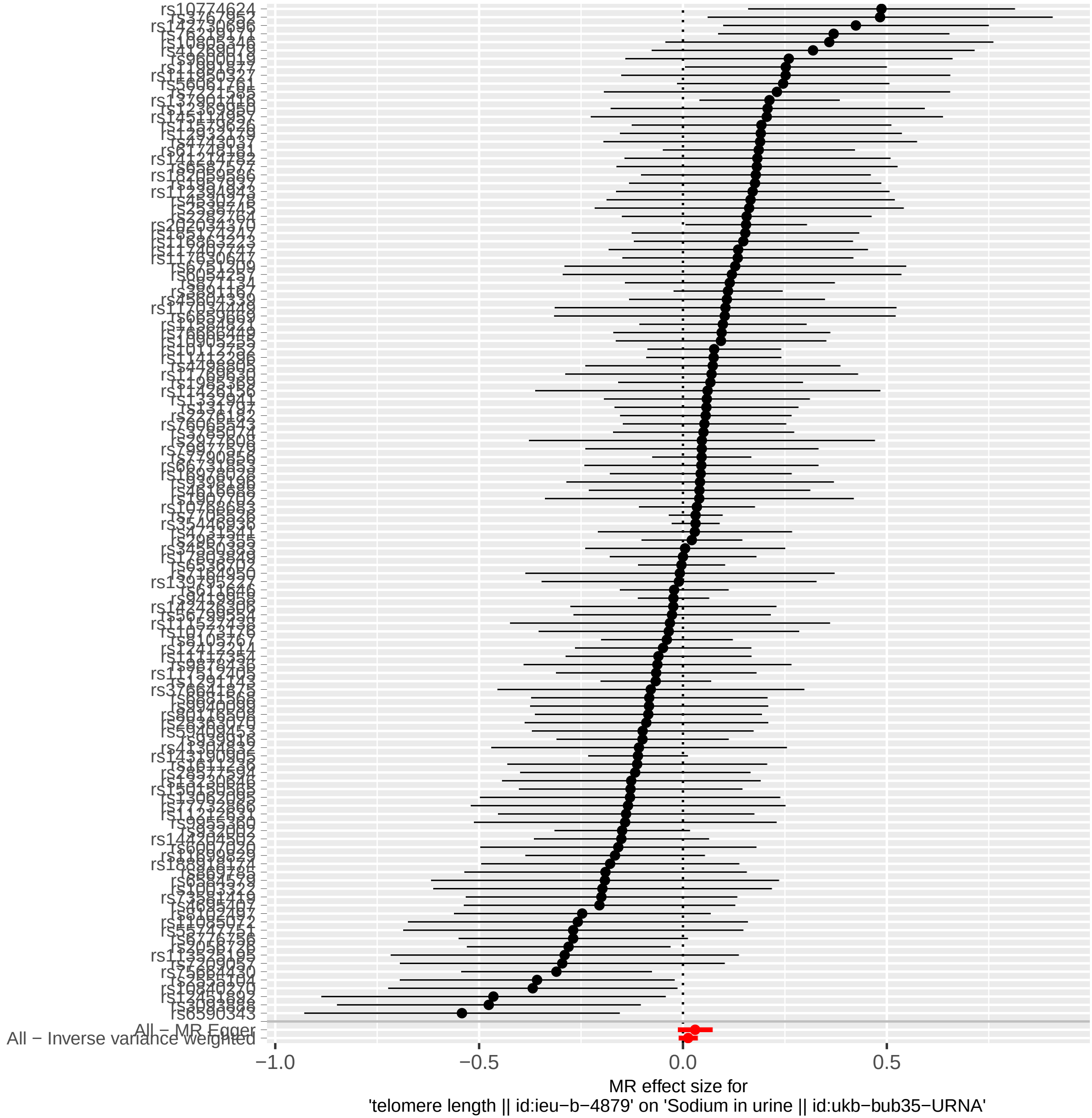
rs10774624
rs6590343
All − MR Egger
All − Inverse variance weighted
−1.0
−0.5
0.0
0.5
MR effect size for
'telomere length || id:ieu−b−4879' on 'Sodium in urine || id:ukb−bub35−URNA'

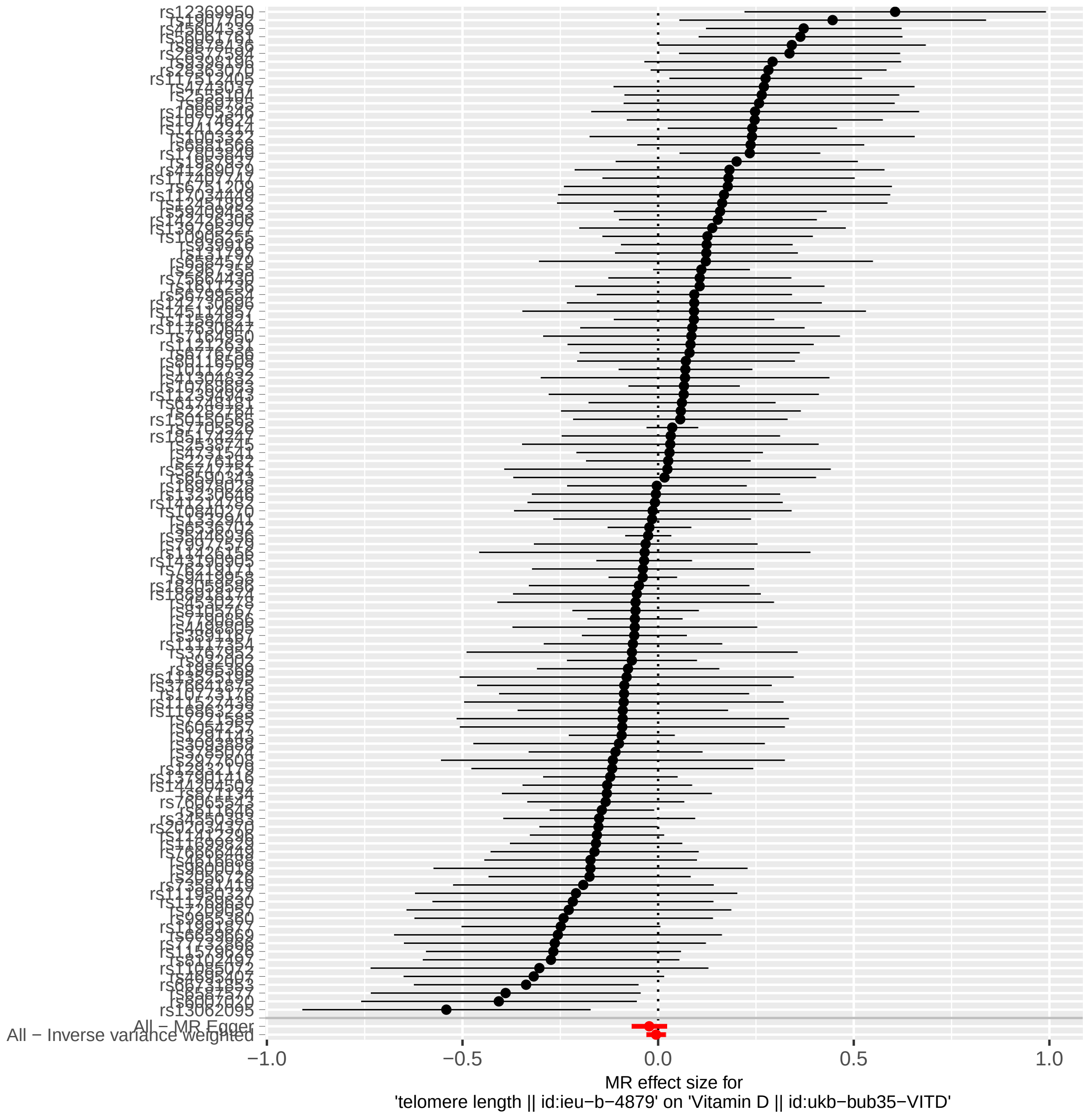

rs12369950
rs13062095
All − MR Egger
All − Inverse variance weighted
−1.0
−0.5
0.0
0.5
1.0
MR effect size for
'telomere length || id:ieu−b−4879' on 'Vitamin D || id:ukb−bub35−VITD'

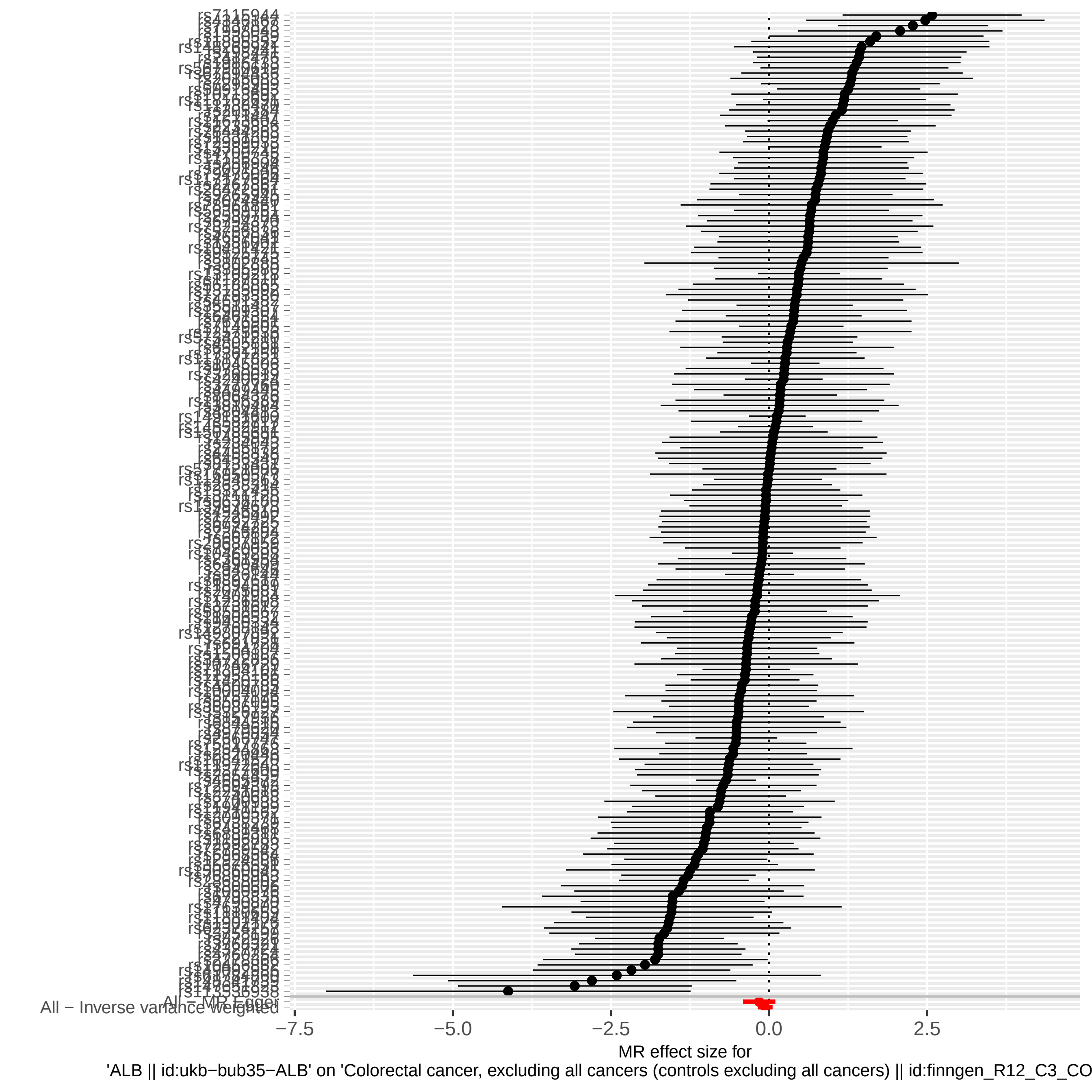

rs7115944
rs113536938
All − MR Egger
All − Inverse variance weighted
−7.5
−5.0
−2.5
0.0
2.5
MR effect size for
'ALB || id:ukb−bub35−ALB' on 'Colorectal cancer, excluding all cancers (controls excluding all cancers) || id:finngen_R12_C3_CO

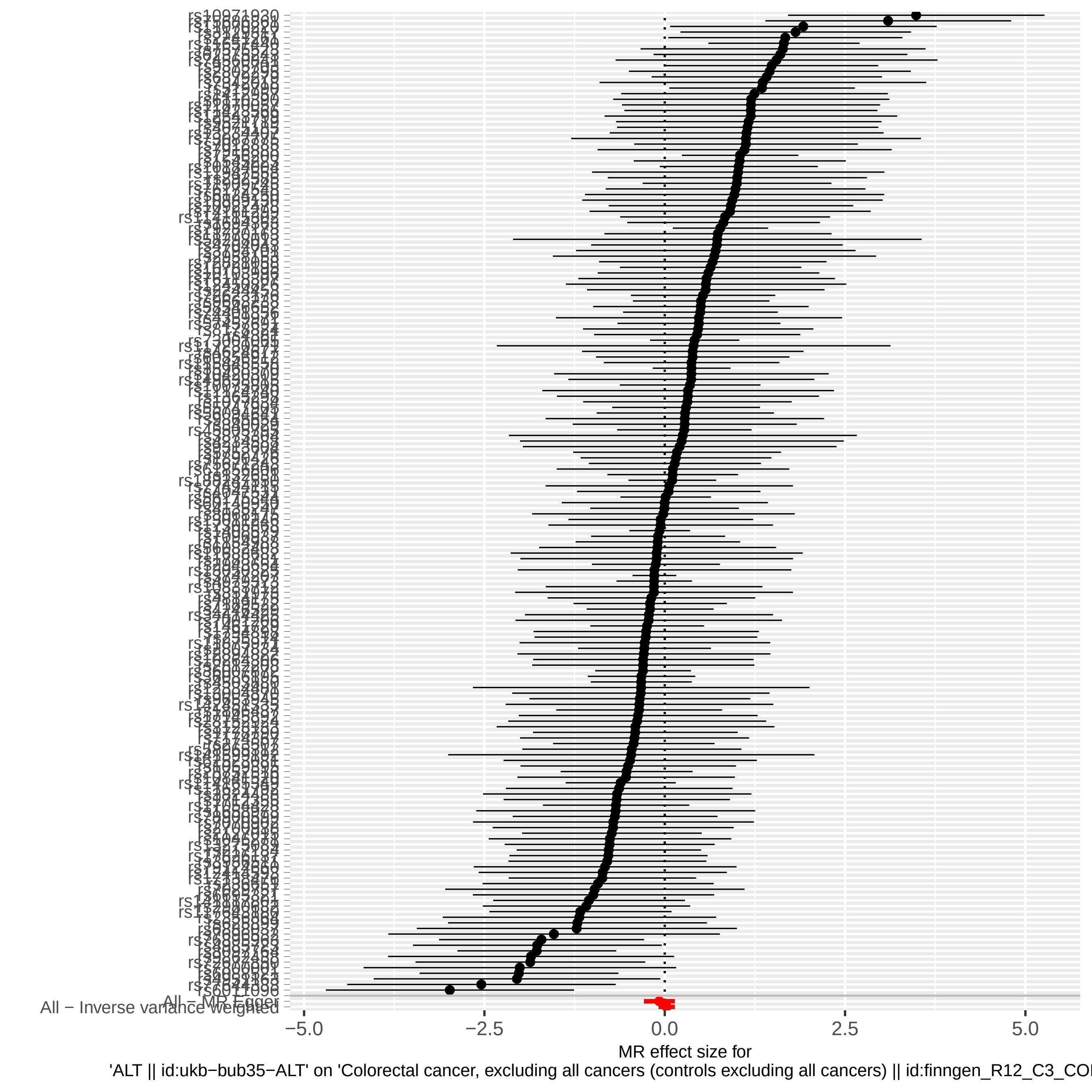
All − MR Egger
All − Inverse variance weighted
−5.0
−2.5
0.0
2.5
5.0
MR effect size for
'ALT || id:ukb−bub35−ALT' on 'Colorectal cancer, excluding all cancers (controls excluding all cancers) || id:finngen_R12_C3_CO

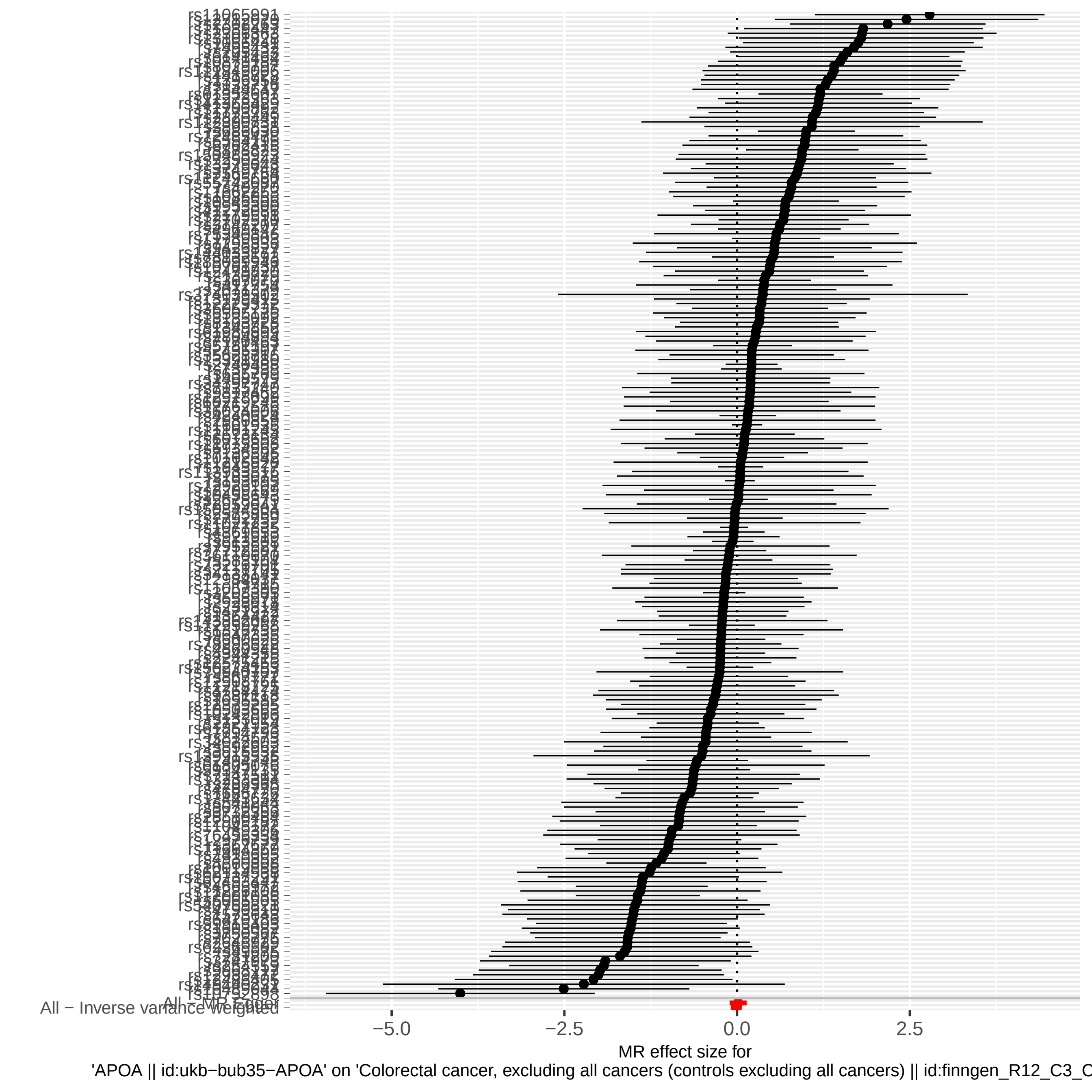
rs11065091
All − MR Egger
All − Inverse variance weighted
−5.0
−2.5
0.0
2.5
MR effect size for
'APOA || id:ukb−bub35−APOA' on 'Colorectal cancer, excluding all cancers (controls excluding all cancers) || id:finngen_R12_C3_C

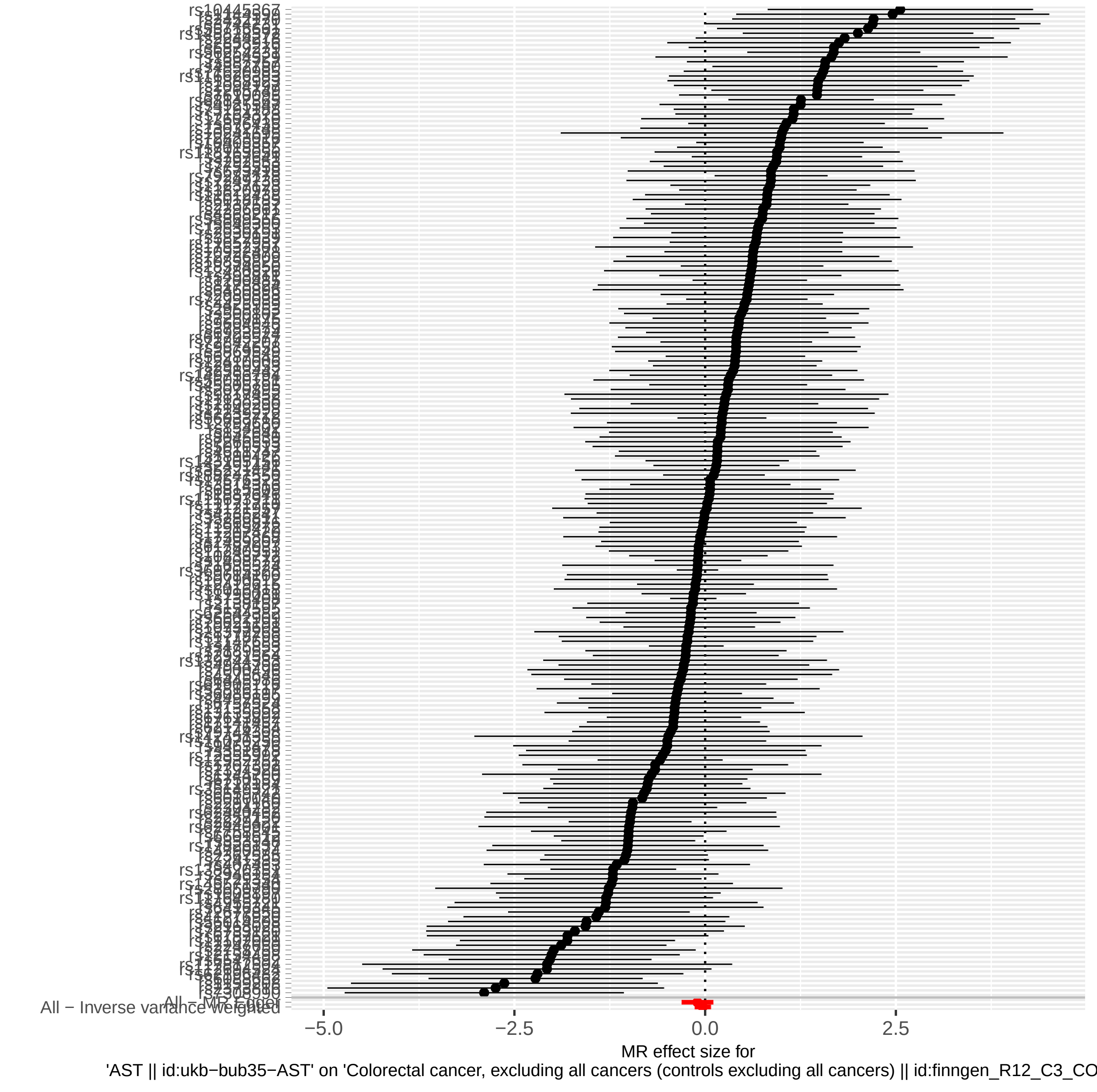
rs10445367
All − MR Egger
All − Inverse variance weighted
−5.0
−2.5
0.0
2.5
MR effect size for
'AST || id:ukb−bub35−AST' on 'Colorectal cancer, excluding all cancers (controls excluding all cancers) || id:finngen_R12_C3_CO

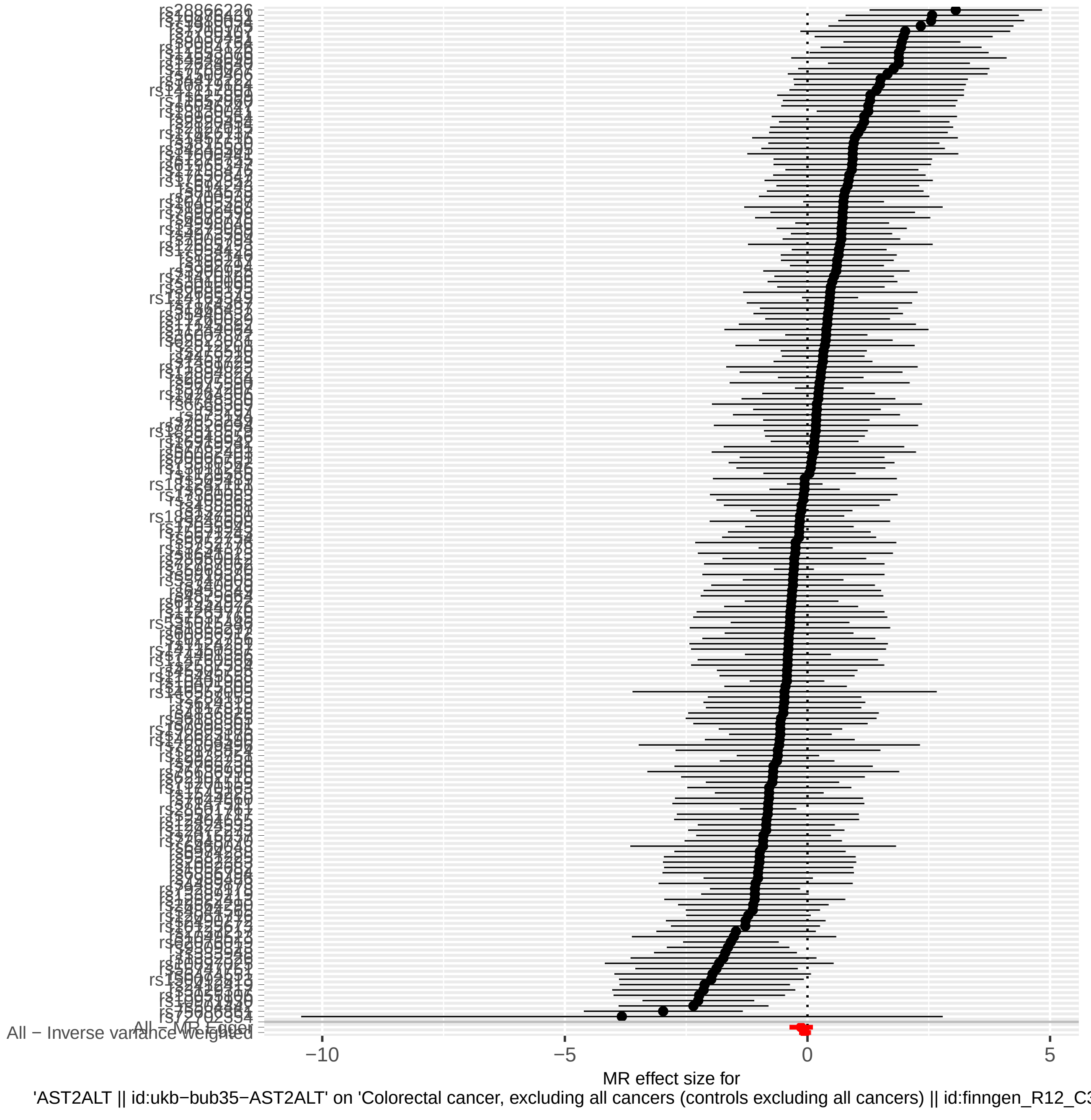

rs28866226
rs72902354
All − MR Egger
All − Inverse variance weighted
−10
−5
0
5
MR effect size for
'AST2ALT || id:ukb−bub35−AST2ALT' on 'Colorectal cancer, excluding all cancers (controls excluding all cancers) || id:finngen_R12_C

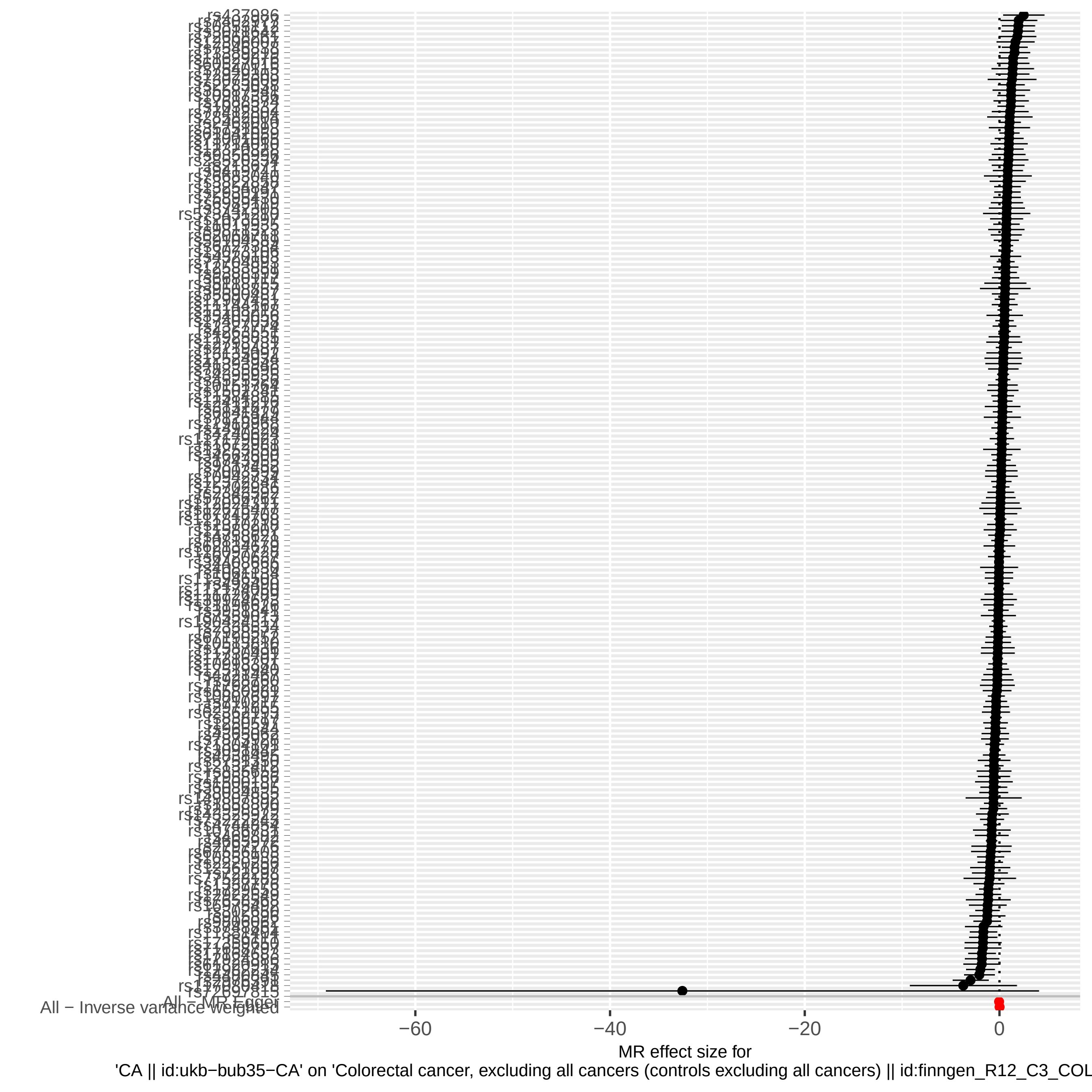

rs437086
rs72697815
All − MR Egger
All − Inverse variance weighted
−60
−40
−20
0
MR effect size for
'CA || id:ukb−bub35−CA' on 'Colorectal cancer, excluding all cancers (controls excluding all cancers) || id:finngen_R12_C3_COL

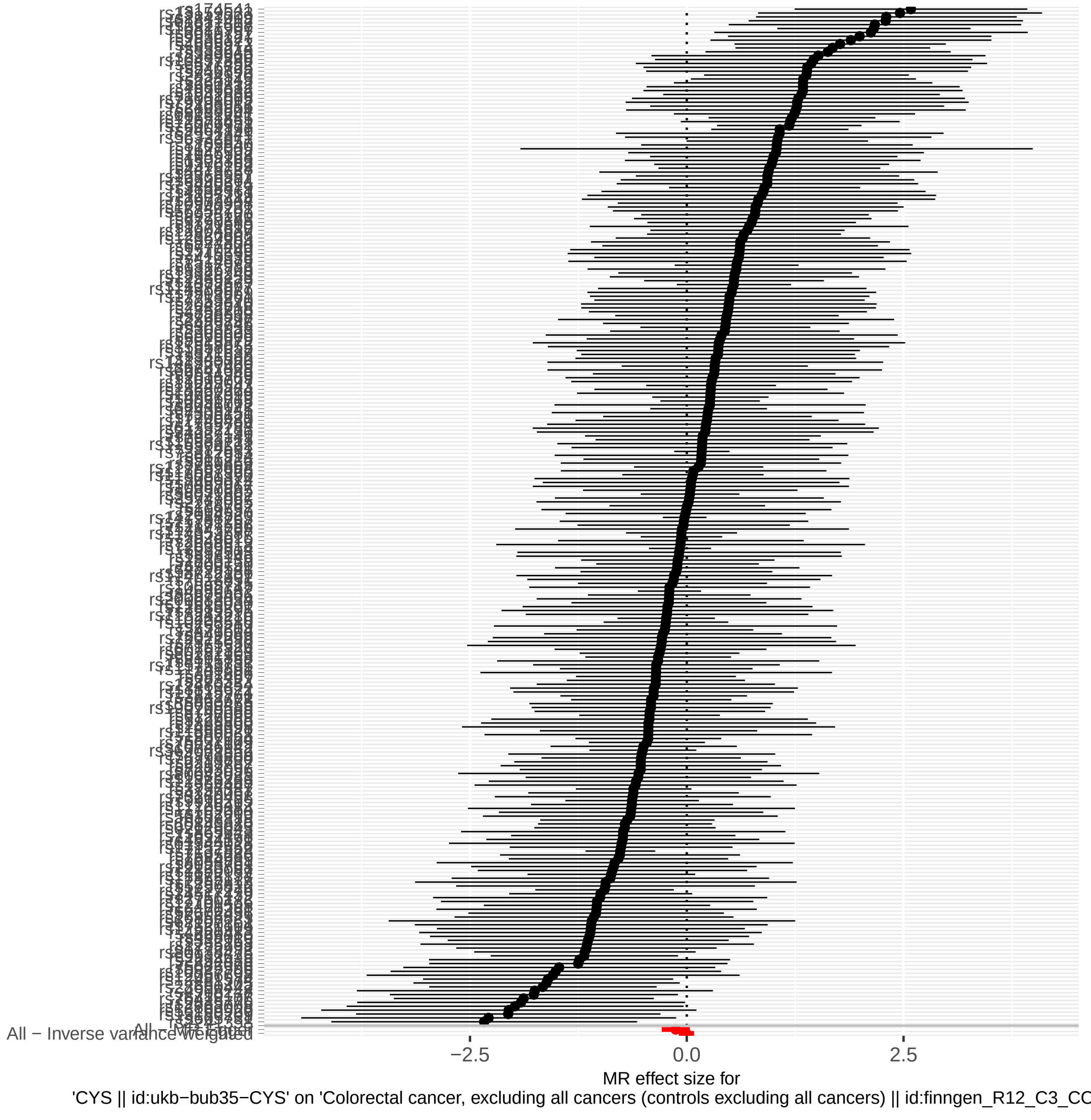

All − Inverse variance weighted
−2.5
0.0
2.5
MR effect size for
'CYS || id:ukb−bub35−CYS' on 'Colorectal cancer, excluding all cancers (controls excluding all cancers) || id:finngen_R12_C3_CO

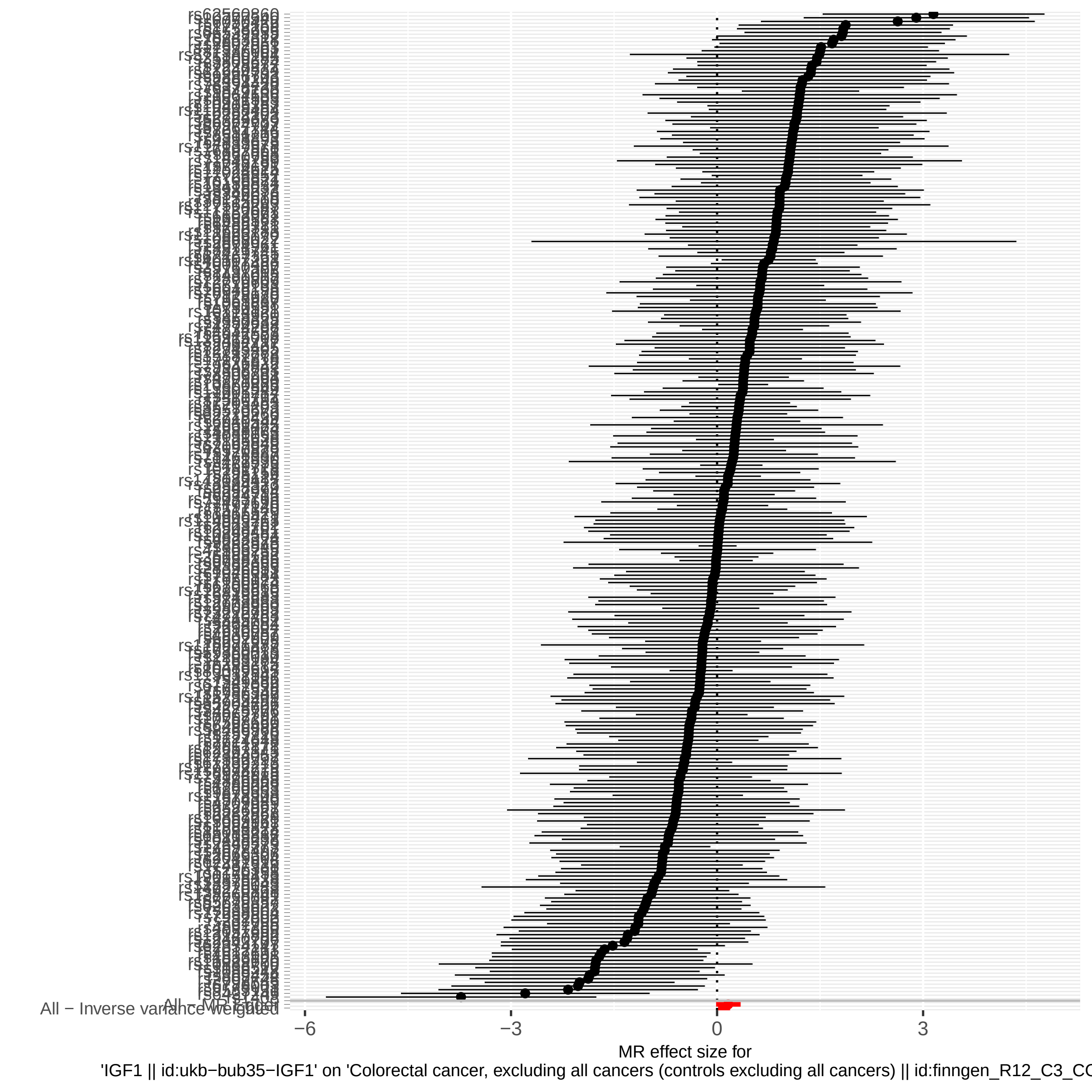

All − Inverse variance weighted
−6
−3
0
3
MR effect size for
'IGF1 || id:ukb−bub35−IGF1' on 'Colorectal cancer, excluding all cancers (controls excluding all cancers) || id:finngen_R12_C3_CO

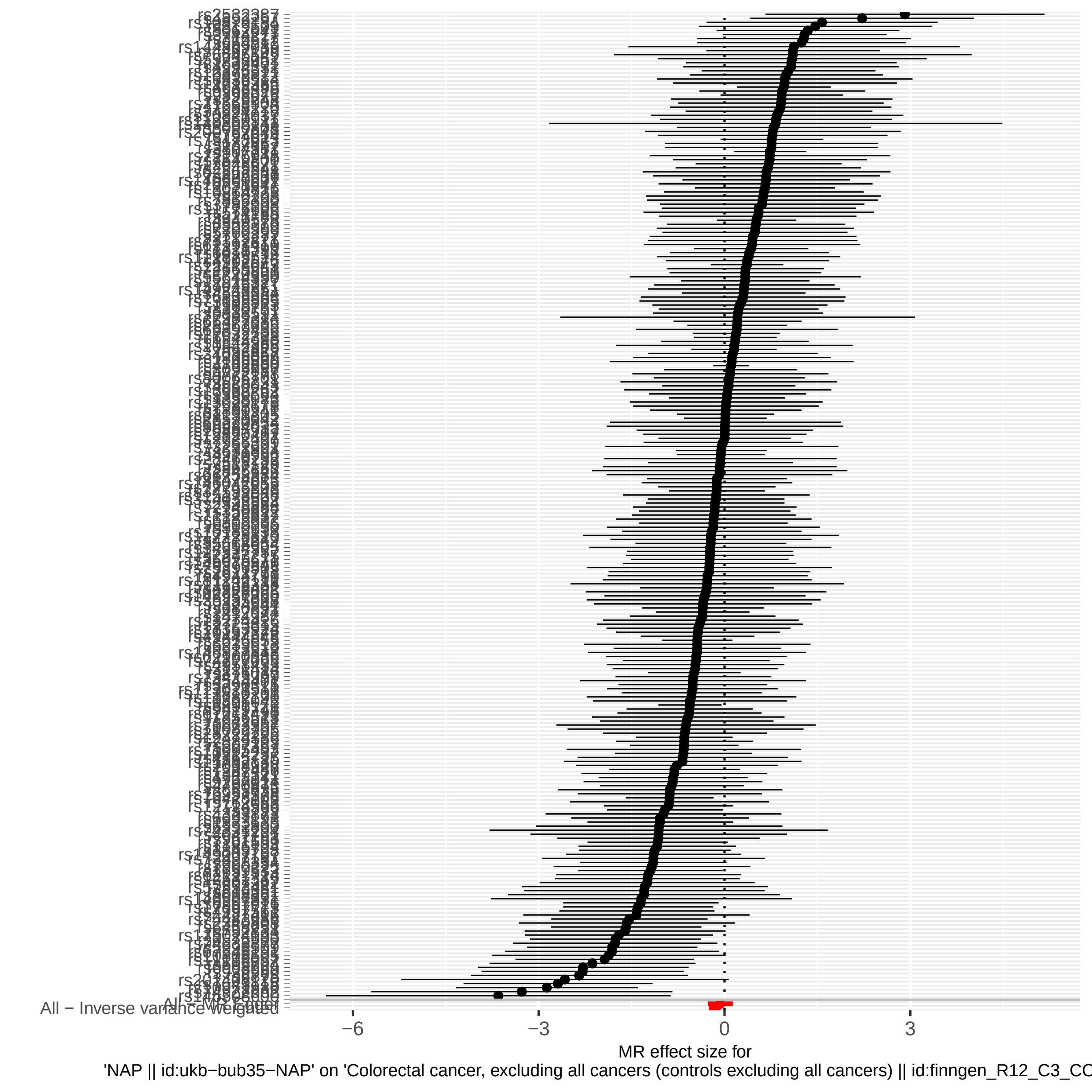

All − MR Egger
All − Inverse variance weighted
−6
−3
0
3
MR effect size for
'NAP || id:ukb−bub35−NAP' on 'Colorectal cancer, excluding all cancers (controls excluding all cancers) || id:finngen_R12_C3_CO

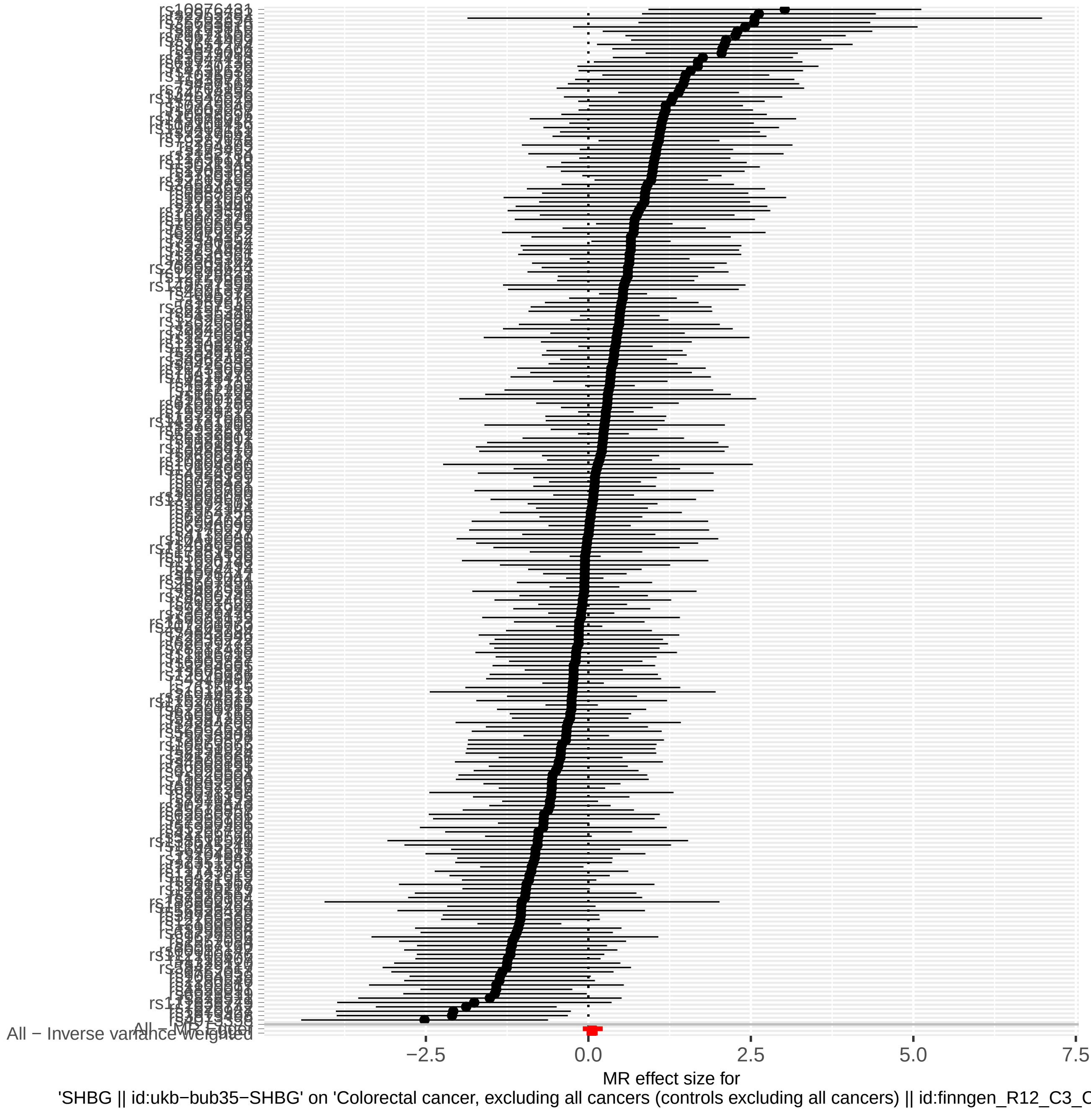

rs10876431
All − MR Egger
All − Inverse variance weighted
−2.5
0.0
2.5
5.0
7.5
MR effect size for
'SHBG || id:ukb−bub35−SHBG' on 'Colorectal cancer, excluding all cancers (controls excluding all cancers) || id:finngen_R12_C3_C

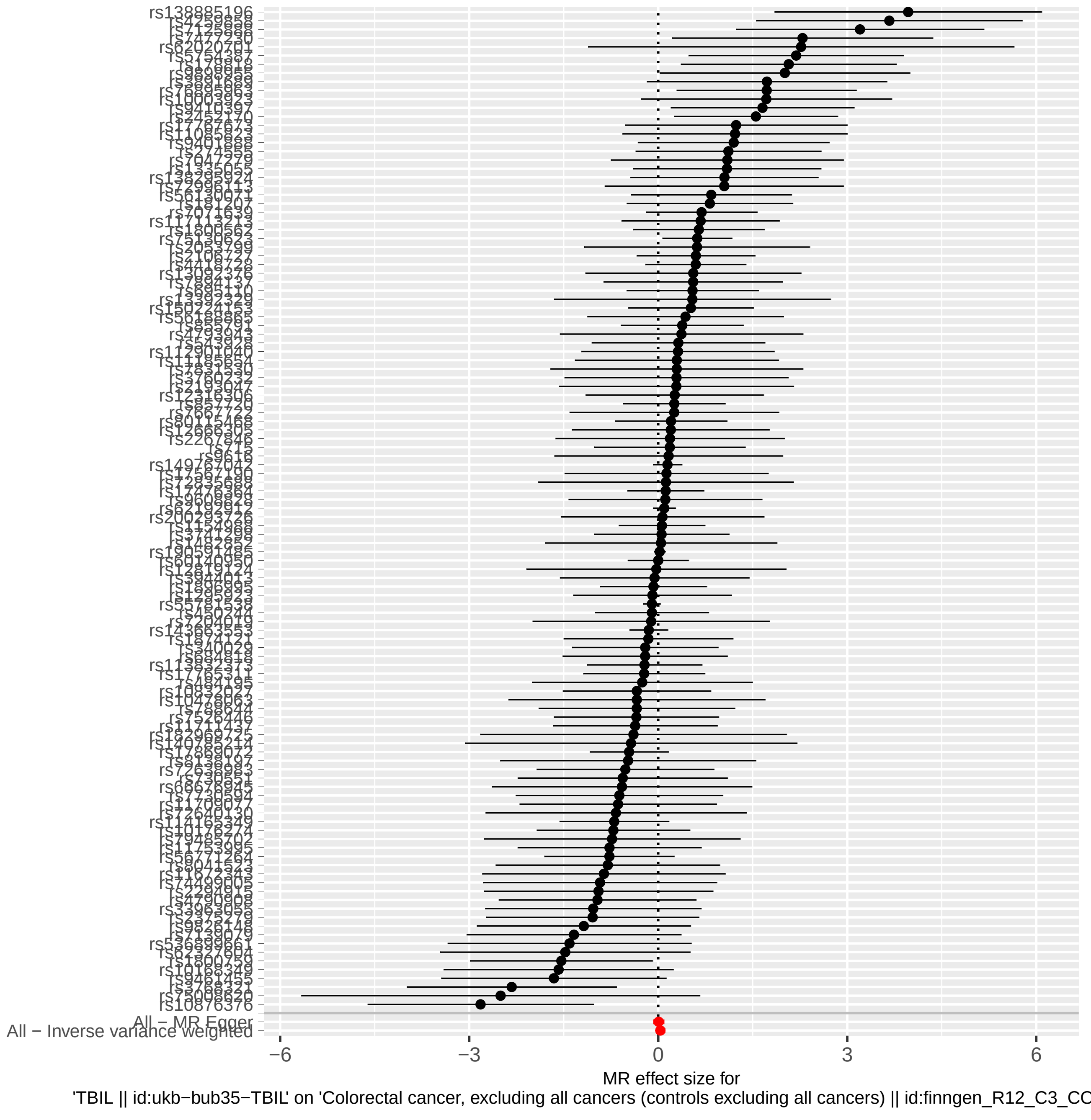

rs138885196
rs10876376
All − MR Egger
All − Inverse variance weighted
−6
−3
0
3
6
MR effect size for
'TBIL || id:ukb−bub35−TBIL' on 'Colorectal cancer, excluding all cancers (controls excluding all cancers) || id:finngen_R12_C3_CO

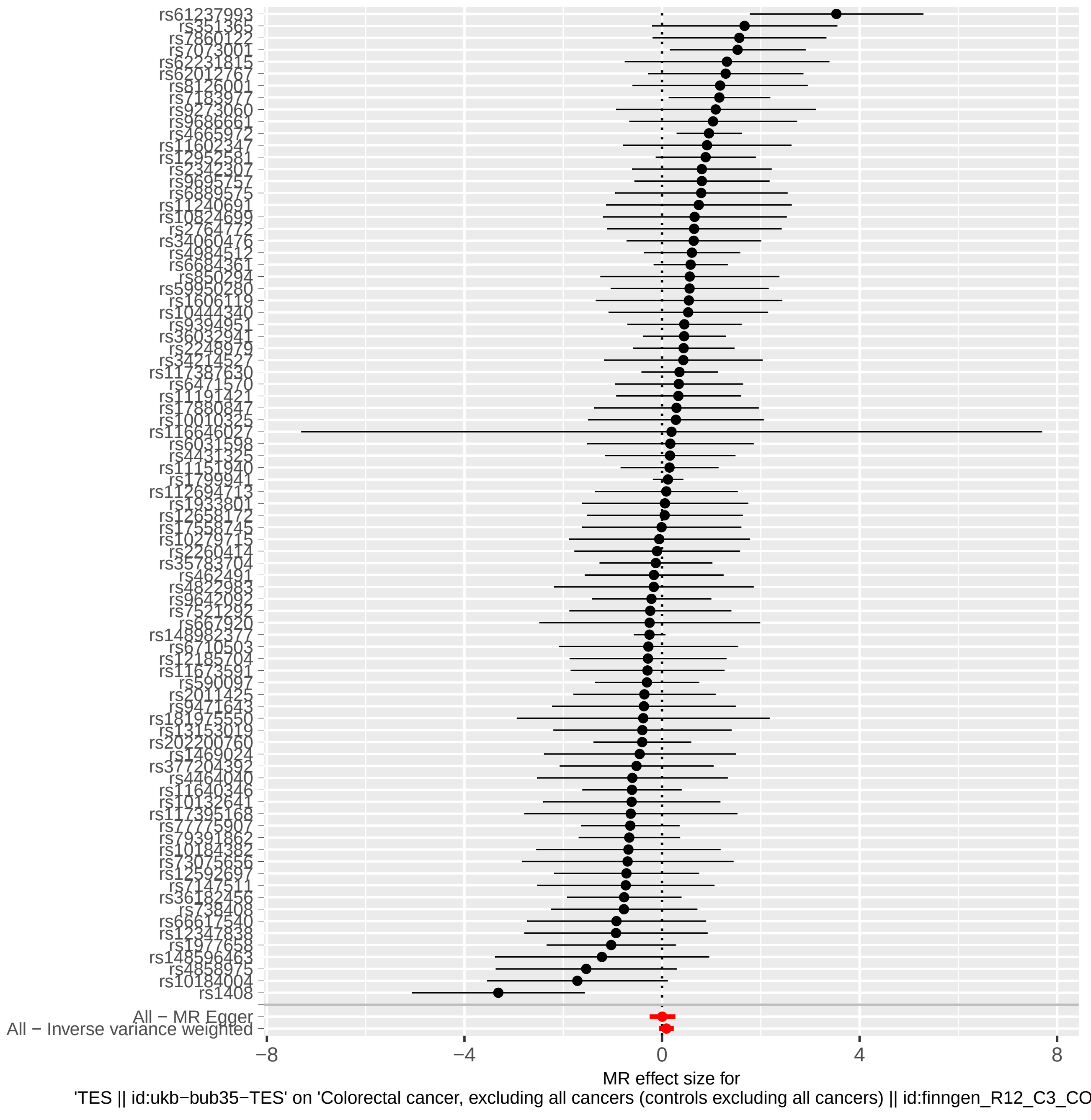

rs61237993
rs351365
rs7860122
rs7073001
rs62231815
rs62012767
rs8126001
rs7183977
rs9273060
rs9686661
rs4665972
rs11602347
rs12952581
rs2342307
rs9695757
rs6889575
rs11240691
rs10824699
rs2764772
rs34060476
rs4984512
rs6684361
rs850294
rs59950280
rs1606119
rs10444340
rs9394951
rs36032941
rs2248979
rs34214527
rs117387630
rs6471570
rs11191421
rs17880847
rs10010325
rs116646027
rs6031598
rs4431325
rs11151940
rs1799941
rs112694713
rs1933801
rs12658172
rs17558745
rs10279715
rs2260414
rs35783704
rs462491
rs4822983
rs9642092
rs7521292
rs667920
rs148982377
rs6710503
rs12185704
rs11673591
rs590097
rs2011425
rs9471643
rs181975550
rs13153019
rs202200760
rs1469024
rs377204392
rs4464040
rs11640346
rs10132641
rs117395168
rs7775907
rs79391862
rs10184382
rs73075656
rs12592697
rs7147511
rs36182456
rs738408
rs66617540
rs12347838
rs1977658
rs148596463
rs4858975
rs10184004
rs1408
All − MR Egger
All − Inverse variance weighted
−8
−4
0
4
8
MR effect size for
'TES || id:ukb−bub35−TES' on 'Colorectal cancer, excluding all cancers (controls excluding all cancers) || id:finngen_R12_C3_CO

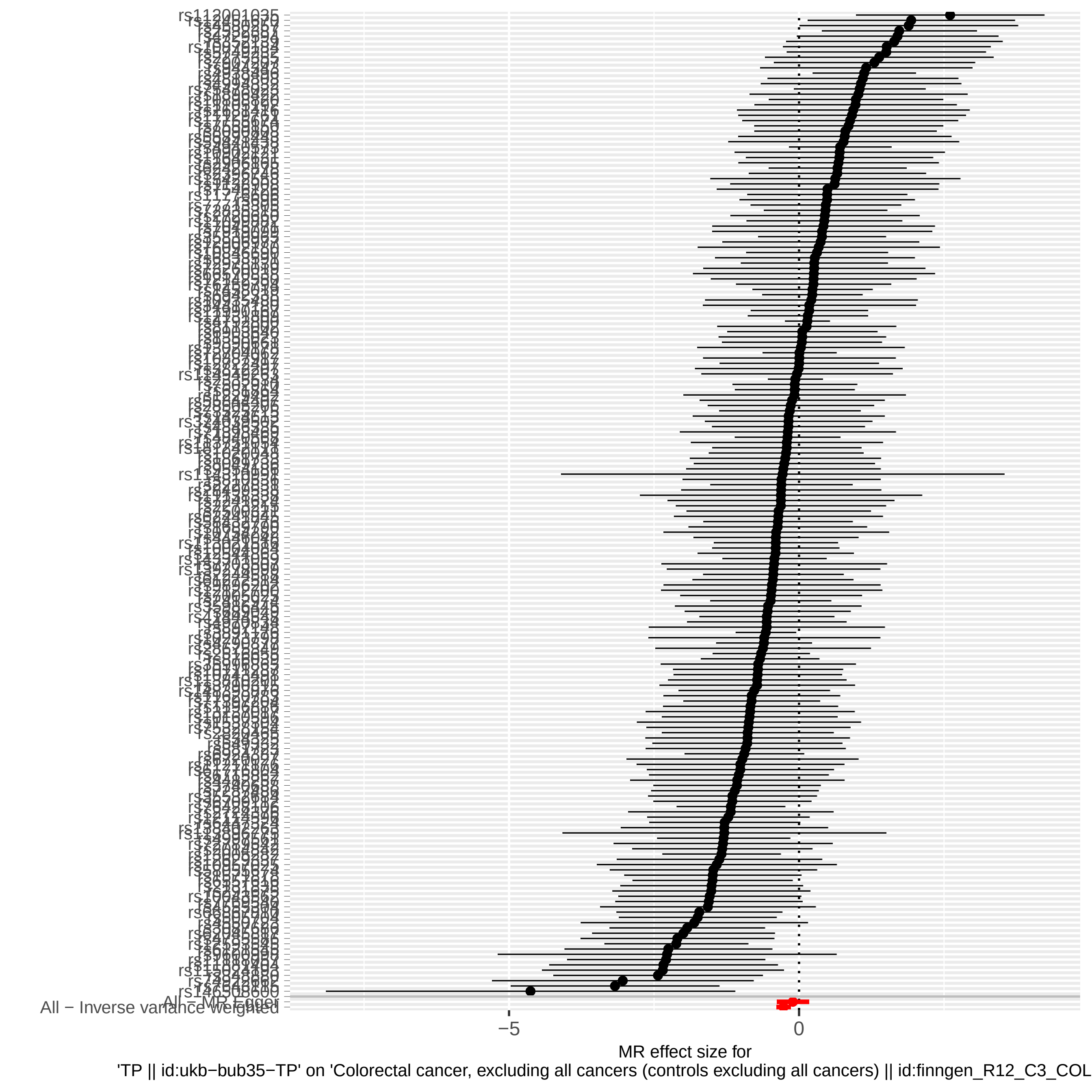
rs112001035
rs146508600
All − MR Egger
All − Inverse variance weighted
−5
0
MR effect size for
'TP || id:ukb−bub35−TP' on 'Colorectal cancer, excluding all cancers (controls excluding all cancers) || id:finngen_R12_C3_COL

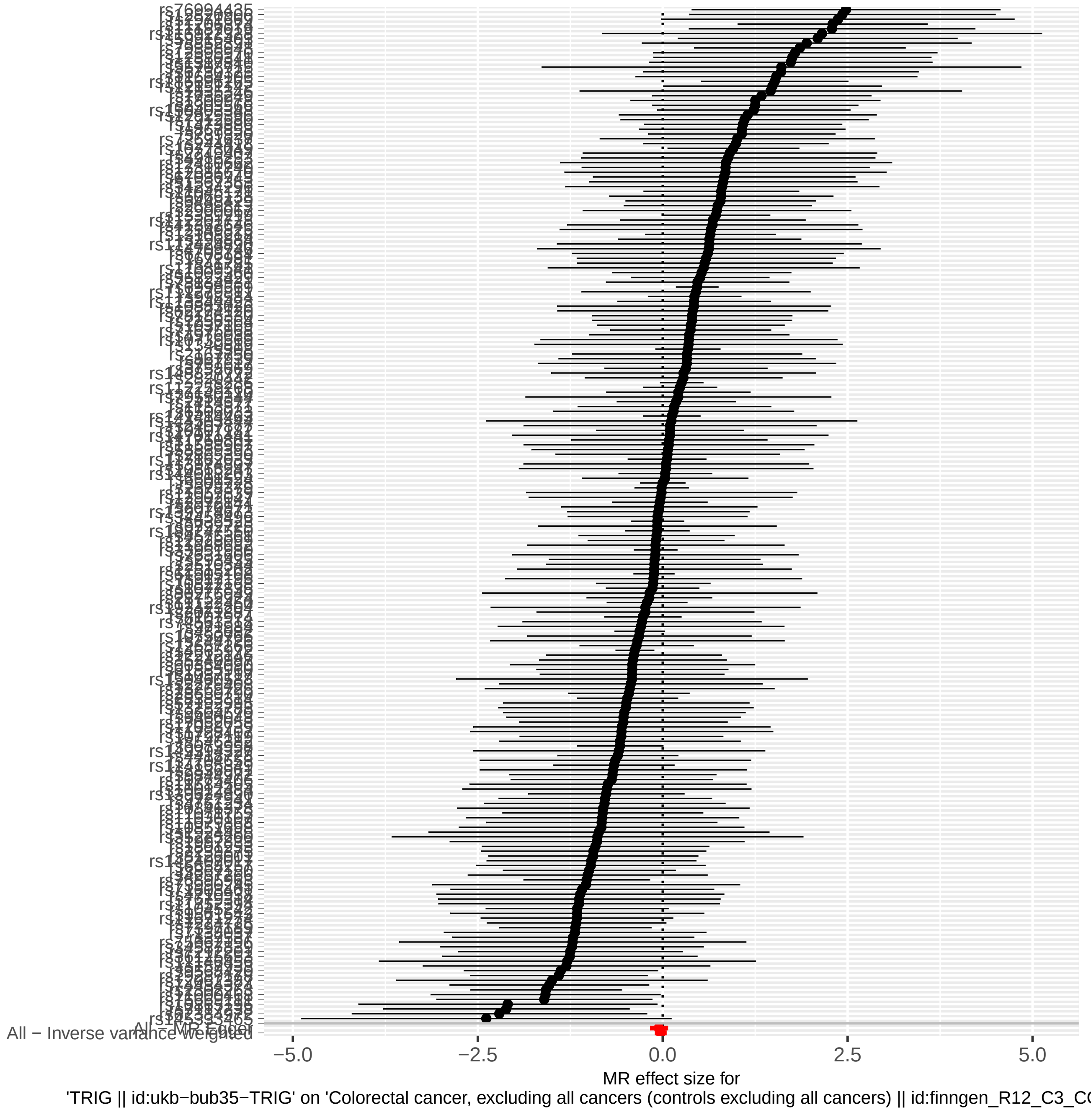

rs76994435
All − MR Egger
All − Inverse variance weighted
−5.0
−2.5
0.0
2.5
5.0
MR effect size for
'TRIG || id:ukb−bub35−TRIG' on 'Colorectal cancer, excluding all cancers (controls excluding all cancers) || id:finngen_R12_C3_C

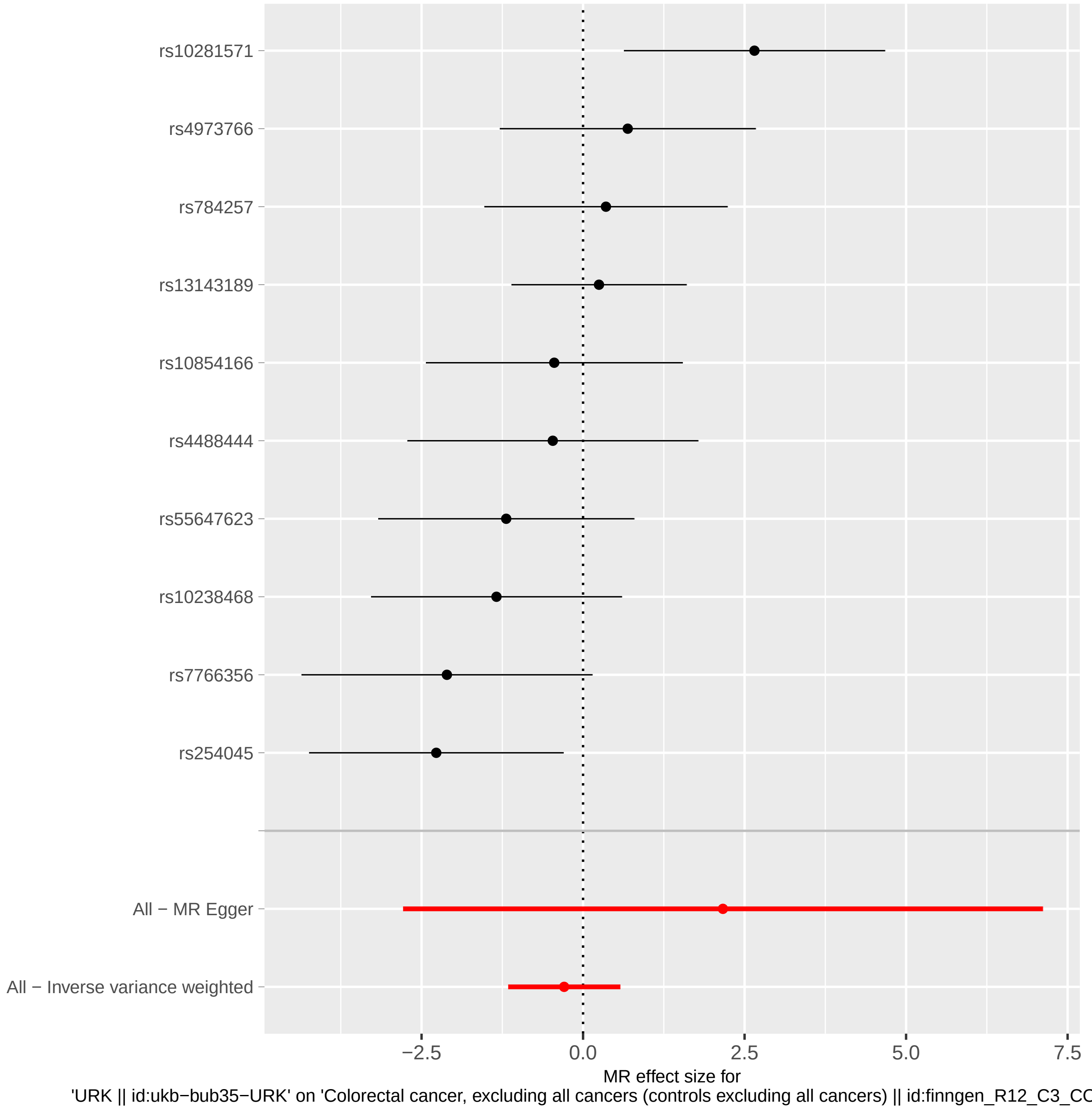

rs10281571
rs4973766
rs784257
rs13143189
rs10854166
rs4488444
rs55647623
rs10238468
rs7766356
rs254045
All − MR Egger
All − Inverse variance weighted
−2.5
0.0
2.5
5.0
7.5
MR effect size for
'URK || id:ukb−bub35−URK' on 'Colorectal cancer, excluding all cancers (controls excluding all cancers) || id:finngen_R12_C3_CO
